# Development of New Indole-Based *N*‑Heterocyclic Carbene Copper Complexes and Their Applications in Catalysis

**DOI:** 10.1021/acs.joc.5c00248

**Published:** 2025-05-28

**Authors:** Chun-Fa Lin, Ya-Hsuan Tseng, Hui-Yu Hsieh, Pei-Jei Hung, Yan-Wen Huang, Dong-Sheng Lee, Ta-Jung Lu

**Affiliations:** Department of Chemistry, 34916 National Chung Hsing University, Taichung 40227, Taiwan

## Abstract

This study disclosed the synthesis and characterization of a series of copper *N*-heterocyclic carbene (NHC) complexes incorporating an indole skeleton. Among these complexes, the Cu-NHC complex with a *p*-methoxyphenyl group on the indole ring exhibited stability in air for up to six months. In contrast, Cu-NHC complexes bearing alkyl groups on the indole ring were stable under a nitrogen atmosphere for one month. These newly developed Cu-NHC complexes proved effective as catalysts for hydrosilylation of carbonyls in 0.5 mol % loading and *N*-arylation of oxazolidinones and amides with aryl iodides in 8 mol % loading.

## Graphical abstract

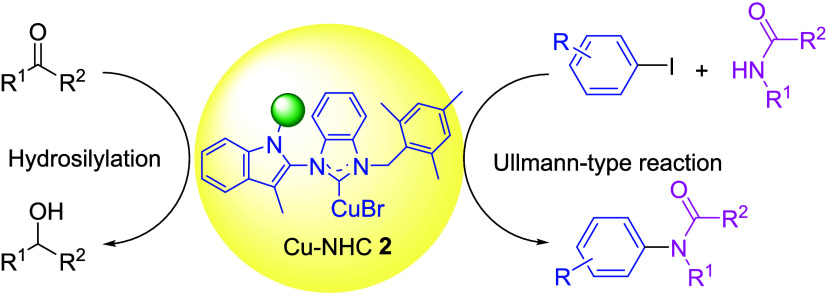

## Introduction

The robust σ-donating property of *N*-heterocyclic carbene (NHC) ligands plays a crucial role in forming strong bonds with various transition metals and thereby enhancing the stability of the resulting complexes. Consequently, NHC ligands have garnered tremendous attention in the scientific community. Over the past two decades, copper-NHC (Cu-NHC) complexes have been extensively studied and applied in catalysis,^1–7^ primarily due to the cost-effectiveness and availability of copper as a metal. Numerous neutral Cu–mono-NHC complexes^8–13^ [Cu­(NHC)­X] (X = halide, acetate, hydride, etc.) have been developed as efficient catalysts for a wide range of transformations, such as hydrosilylation of ketones,^14–21^ click chemistry,^22–26^ alkene boration.^27–30^ Cu­(I)-NHCs have also proven valuable as carbene transfer reagents, facilitating the preparation of transition metal-NHC complexes with metals like gold, palladium, nickel, ruthenium, rhodium, and chromium.^31–40^ Furthermore, studies on the biological activity of Cu-NHC complexes, particularly their potential as antitumor agents, have shown promising results,^41–43^ highlighting their potential therapeutic applications.

Building on this foundation, the indole scaffold has proven to be a versatile platform for ligand design, offering steric and electronic tunability along with high chemical stability. These features make it highly attractive for applications in metal-catalyzed reactions. Consequently, ligands incorporating indole moieties have recently attracted attention for their unique steric and electronic contributions to metal-catalyzed reactions.^44–64^ Among these, indole-substituted NHC ligands have been developed and demonstrated excellent catalytic activity in Pd-catalyzed carbon–carbon bond formation reactions.^60–64^ Notably, only one study has documented the synthesis of Cu­(I)-NHC complexes through the reaction of indole-substituted imidazolium salts with Cu_2_O.^57^ Despite their excellent performance in the Cu­(I)-catalyzed carboxylation of organoboronic esters, the synthesis of these Cu complexes remains a significant challenge. This limited investigation highlights a significant gap in the exploration of Cu-NHC complexes featuring indole-based ligands. Building on our ongoing research into indolyl NHC ligands, we present the synthesis, comprehensive characterization, and preliminary catalytic study of a series of Cu-NHC complexes.

## Results and Discussion

### Synthesis and Characterization of Cu­(I)-NHC Complexes

Following our previously reported methods,^61–63^ the synthesis of benzimidazolium salts incorporating indole derivatives was performed. According to the procedure reported by the Cazin group in 2013 for preparing [Cu­(X)­(NHC)] complexes,^65^ salts **1** were reacted with CuCl in the presence of 2.0 equiv K_2_CO_3_, using acetone as solvent at 60 °C (Scheme 1). The indole-substituted NHC–Cu complexes **2** were obtained in yields ranging from 64 to 99%. The EI-MS of complex **2a** revealed a [Cu­(Br)­(NHC)]^+^ ion peak at *m*/*e* = 629.3, indicating the replacement of chloride by bromide in acetone. Complexes **2** were also synthesized using CuBr with salts **1**, yielding 89–98%. All complexes **2** were generally stable in air and could be stored as solids for up to 4 weeks, except for **2a**, which remained stable for more than 6 months in the solid state. All complexes remain stable under a nitrogen atmosphere. However, upon exposure to air in solution, all complexes **2** exhibited instability, forming green Cu­(II) species. For instance, the decomposition of **2a** into urea **3** was observed in solution under air (Scheme 2), as confirmed by NMR spectroscopy.^66^ The methylene signal shifted upfield from 5.50 ppm in complex **2a** to 5.07 ppm in compound **3** (Figure 1). All complexes were soluble in DMF, DMSO, THF, dioxane, toluene, CH_2_Cl_2_, acetone, MeOH, EtOAc, and CH_3_CN, but insoluble in *n*-hexanes.

**Figure 1:**
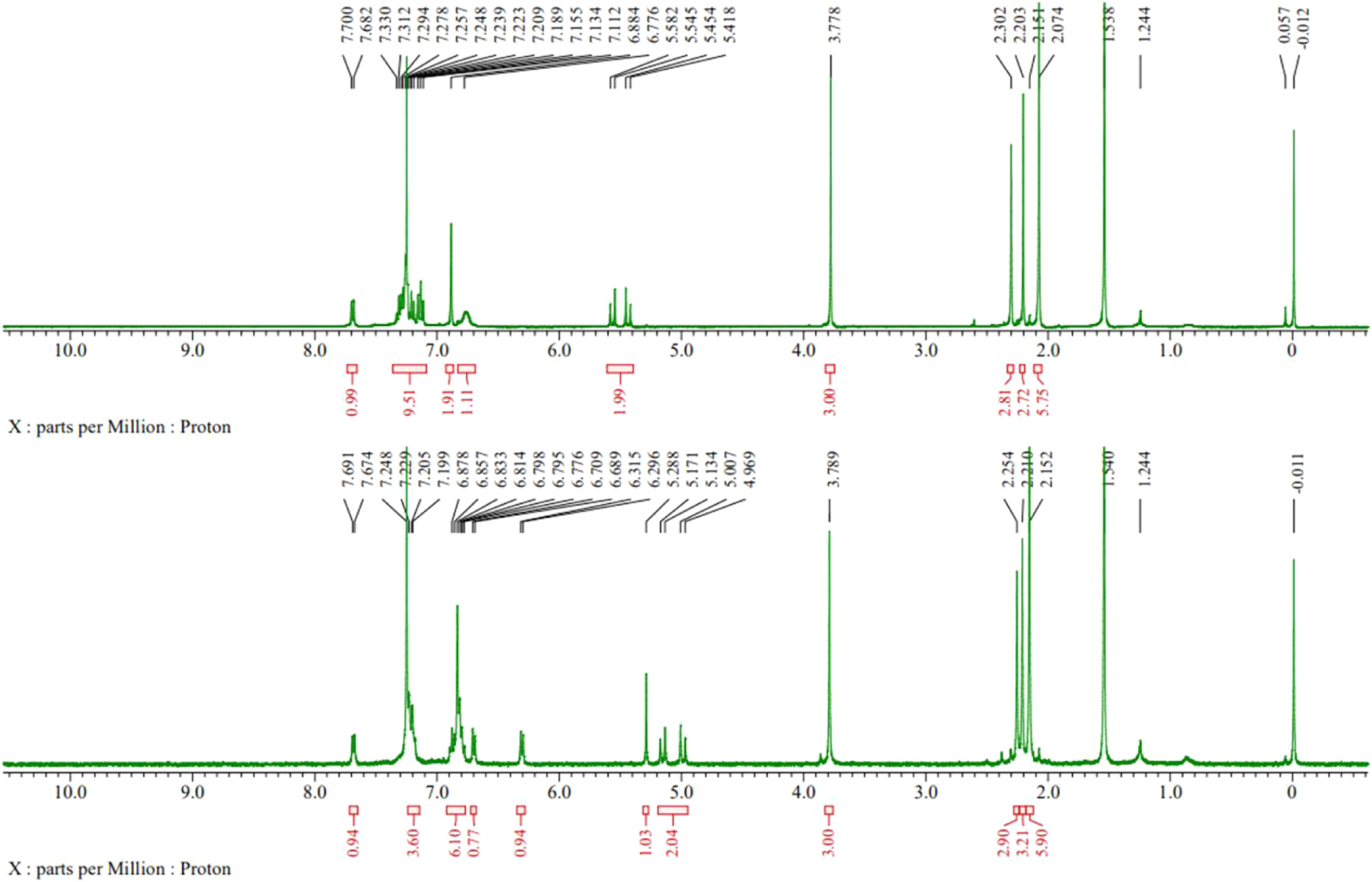
^1^H NMR spectra of [Cu­(Br)­(NHC)] **2a** (top) and **3** (bottom).

**Scheme 1:**
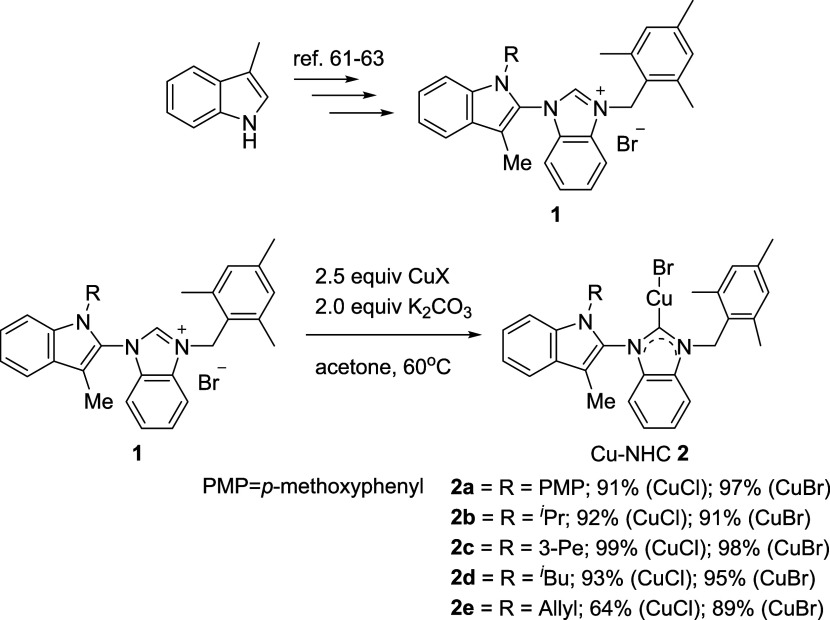
Synthetic Protocols to [Cu­(Br)­(NHC)] Complexes **2**

**Scheme 2:**
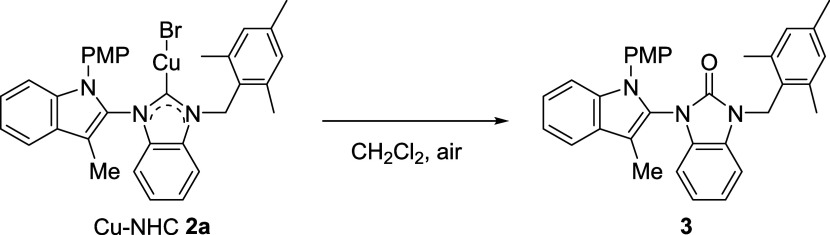
Decomposition of [Cu­(Br)­(NHC)] **2a**

All complexes **2** were initially characterized by NMR spectroscopy. The N–CH–N proton signals of salts **1** were observed as a singlet at δ 11.33–11.79 ppm but were absent in complexes **2**. The methylene signals of the benzimidazolium compounds **1** shifted upfield from 6.31–6.87 ppm to 5.28–5.79 ppm for Cu complexes **2**. Additionally, in their ^13^C­{^1^H} NMR spectra, the signals of the benzimidazolium salts **1** in CDCl_3_ shifted downfield from 140–145 ppm to 185–190 ppm.

A single crystal of complex **2a** for X-ray diffraction analysis was grown by evaporation of a dichloromethane/*n*-hexane solution under N_2_ (Figure 2). The bond distance of the Cu–C(23) bond is measured at 1.878(4) Å, indicating typical Cu–C coordinate bonds in the range of 1.87–2.00 Å.^67^ While the C(23)–Cu-Br angle in complex **2a** is equal to 174.61(12)°, indicating a near-linear geometry. Notably, the indole and the benzimidazole rings of **2a** do not lie on a coplane, making an angle of 64.8° [C(23)–N(2)–C(8)-N(1)]. Similarly, both the indole plane and anisole plane are not coplanar, as the dihedral angle is 57.5° [C(14)–C(9)–N(1)-C(8)] for **2a**. The angle between the mesityl plan and the benzimidazole plan is measured 83.7°.

**Figure 2:**
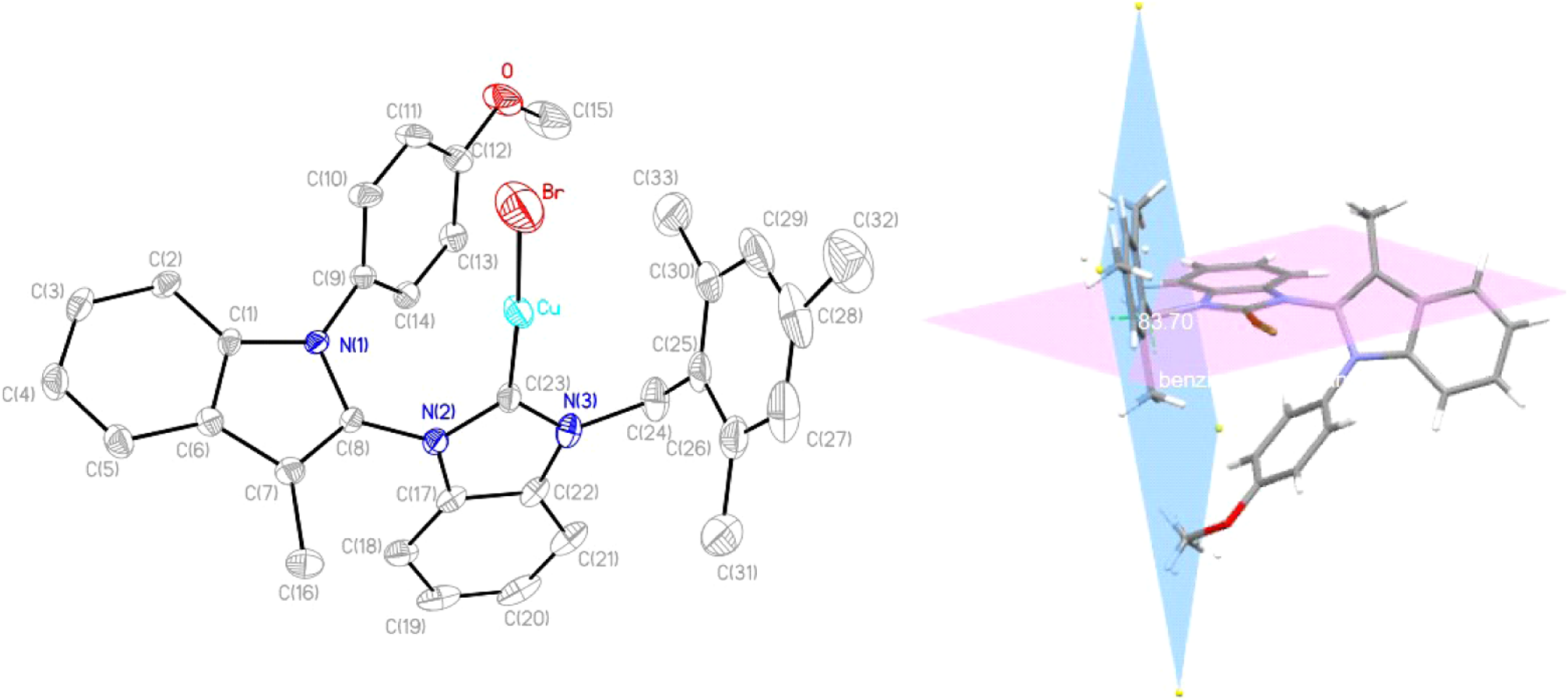
Crystal structure of **2a** (CCDC2402285) with thermal ellipsoids drawn at the 50% probability levels. Hydrogen atoms are omitted for clarity.

### Catalytic Activities of Cu­(I)-NHC Complexes **2** for Hydrosilylation Reaction of Carbonyl Compounds

The hydrosilylation of carbonyl compounds is a versatile method for evaluating the catalytic efficiency of Cu-NHC complexes.^3,14–16,68–76^ The resulting primary and secondary alcohols are significant intermediates in the synthesis of pharmaceuticals, agrochemicals, and fragrances. In this study, Cu-NHC complexes **2** were screened for the hydrosilylation of 1-(naphthalen-2-yl)­ethan-1-one (**4a**) (Table 1). At a catalyst loading of 1 mol %, all Cu-NHC complexes demonstrated activity, with **2a** (bearing a *p*-methoxyphenyl substituent) yielding **5a** at up to 82%.

**Table 1:**
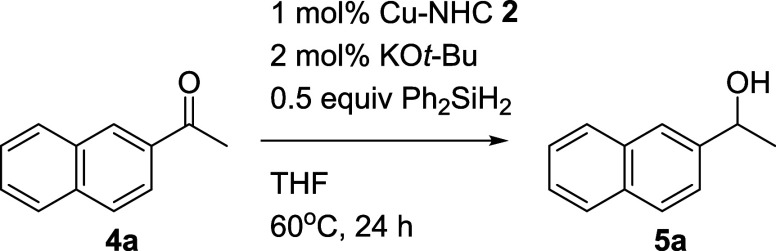

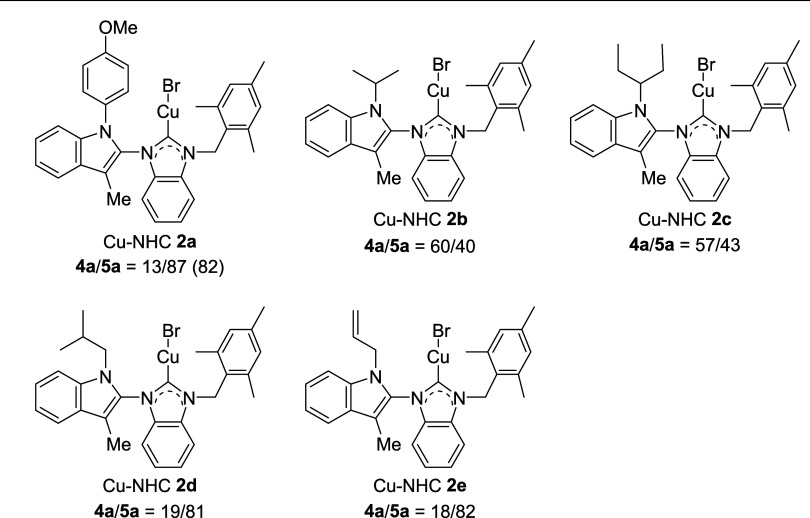
Performance of Cu-NHC **2** in the Hydrosilylation of **4a** ^a^,^b^

a The reaction was carried out on a 1 mmol scale of **4a**.

b The ratio was determined by 400 MHz NMR. Isolated yield of **5a** was shown in parentheses.

Optimization of the reaction conditions, such as base, silane, and solvent, was carried out using **2a** as the catalyst. Employing a design of experiments approach enabled us to assess a broad range of variables with minimal assays, yielding crucial insights into critical factors and optimal combinations for maximizing product yield. This study used the Taguchi L9 (3^3^) orthogonal array to determine optimum conditions (see Supporting Information, Table S1, entries 1–9). The optimum reaction conditions were KO^*t*^Bu (2 mol %) as a base, Ph_2_SiH_2_ (0.5 equiv) as a hydrogen source, and THF as a solvent. Further exploration of reaction parameters such as temperature, time, and catalyst loading provided a detailed understanding of their impact on the reaction outcome (entries 10–17). Finally, the hydrosilylation of **4a** was achieved at 40 °C within 4 h using 0.5 mol % of **2a** and 0.6 equiv of Ph_2_SiH_2_, yielding 98% of the target product (entry 16).

The optimized reaction conditions for Cu-NHC **2a**-catalyzed hydrosilylation were further explored with a range of carbonyl compounds (Table 2). Ketones with electron-deficient and electron-rich groups at the para positions on aromatic rings (**4b**–**4e**) yielded excellent results (90–98%). However, sterically hindered aromatic ketone **4g** exhibited poor reactivity under the optimum conditions, achieving only an 18% yield. When the temperature was increased to 60 °C and the reaction time extended to 24 h, the yield improved significantly to 82%. Heterocyclic aromatic ketones **4h** and **4i**, were subjected to the catalytic conditions, resulting in the corresponding alcohols **5h** and **5i** in 81 and 70% yields, respectively. Benzophenone **4j** was also reduced to **5j** in 97% yield. Aliphatic ketones (**4k**–**4n**) also gave the reduction products (**5k**–**5n**) with good yields (77–98%). Except for ketones, aldehydes (**4o**–**4t**) were also suitable for Cu-NHC **2a**-catalyzed hydrosilylation. The primary alcohols (**5o**–**5t**) were achieved in good to excellent yields (75–98%).

**Table 2:**
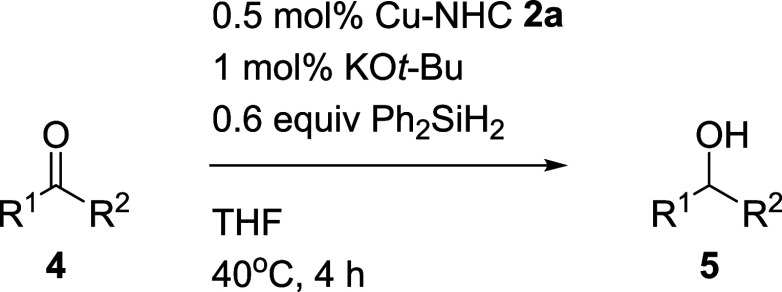

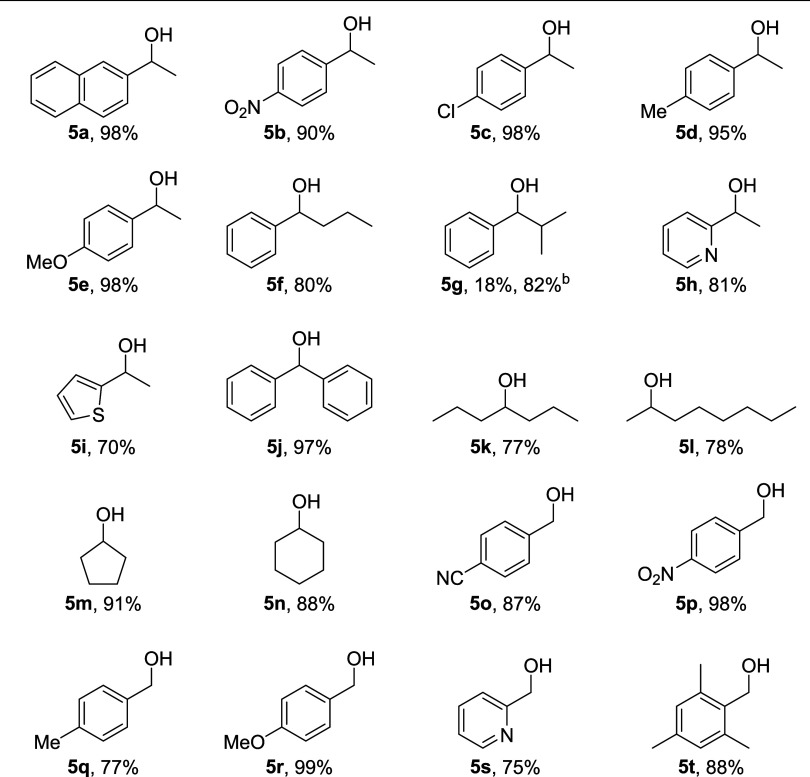
Scope of Carbonyl Compounds in the Cu-catalyzed Hydrosilylation^a^

a The reaction was carried out on a 1 mmol scale of **4a**. Isolated yield of **5** is shown.

b The reaction was stirred for 24 h at 60 °C.

### Catalytic Activities of Cu­(I)-NHC Complexes **2** for *N*-Arylation of Oxazolidin-2-One

*N*-Aryloxazolidinones have important applications in pharmaceuticals, such as Linezolid, Toloxatone, and Eperezolid (Figure 3). They are valuable tools in organic synthesis. Copper-catalyzed Ullmann-type reactions of iodoarenes and oxazolidinone serve as traditional methods for the synthesis of *N*-aryloxazolidinones.^76–82^ The Taguchi L9 (3^3^) orthogonal array was used to determine the optimum conditions. The optimum reaction conditions were K_2_CO_3_ (2 equiv) as a base, DMSO as a solvent, and 120 °C (Table S2). New developed Cu-NHC **2a**–**2d** were used as catalysts for the *N*-arylation of oxazolidinone (Table 3). The experimental results demonstrated that all of the complexes were suitable for this reaction, with complex **2a** indicating the best catalytic efficacy. Even at a reduced loading of 8 mol %, **2a** continued to display a conversion rate of 98% and a yield of 82%.

**Figure 3:**
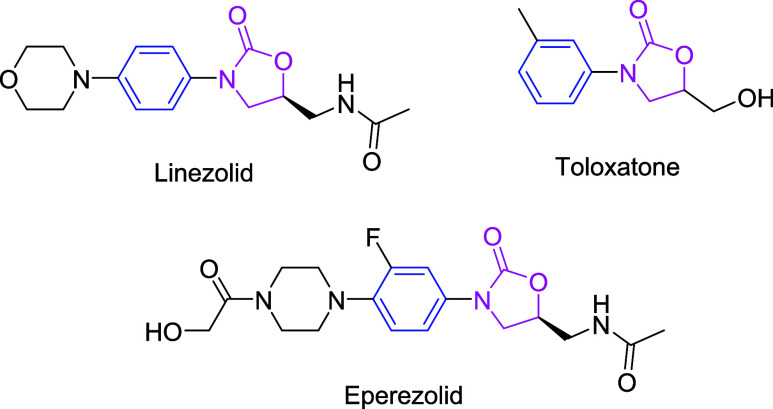
Structures of the bioactive *N*-aryloxazolidinones.

**Table 3:**
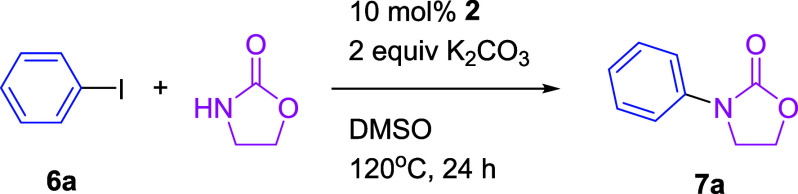

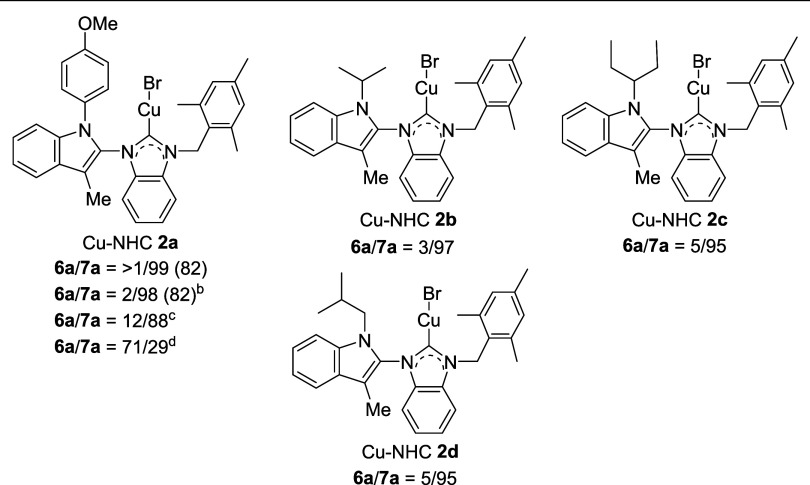
Performance of Cu-NHC **2a**–**2d** in the *N*-arylation of Oxazolidinone^a^

a The reaction was carried out on a 1 mmol scale of **6a**. The ratio was determined by 400 MHz NMR. Isolated yield of **7a** was shown in parentheses.

b Cu-NHC **2a** (8 mol %) was used.

c Cu-NHC **2a** (1 mol %) was used.

d Cu-NHC **2a** (0.1 mol %) was used.

A variety of iodoarenes were employed to investigate the reaction scope for the *N*-arylation of oxazolidinone (Table 4). Typically, iodoarenes featuring electron-deficient substrates (e.g., **6b**–**6f**), giving good to excellent yields (71–94%), exhibited faster reaction rates compared to electron-rich substrates (e.g., **6g**–**6k**), resulting in moderate to good yields (61–81%), under the optimized reaction conditions. The steric hindrance caused by *ortho*-substituted aryl iodides (e.g., **6h** and **6i**) resulted in decreased yields compared to *meta*- or *para*-substituted aryl iodides (**6g**, **6j**, and **6k**). It is worth noting that using **6b** as a reactant successfully minimized the halogen-exchange pathway in the Cu-NHC **2a**-catalyzed C–N bond coupling, with no iodo-substituted product detected. Similarly, the use of **6c** gave the same results. A series of functional groups, including halogen (**6b** and **6c**), nitro (**6d**), trifluoromethyl (**6e**), ketone (**6f**), and methoxy (**6g** and **6h**), were found to be well tolerated under the optimized reaction conditions. The *N*-arylation of heteroaryl substrates, such as 4-iodopyridine (**6l**) and 2-iodothiophene (**6m**), were also found to be successful, but the desired products **7l** and **7m** were obtained in poor yields (20 and 46%, respectively).

**Table 4:**
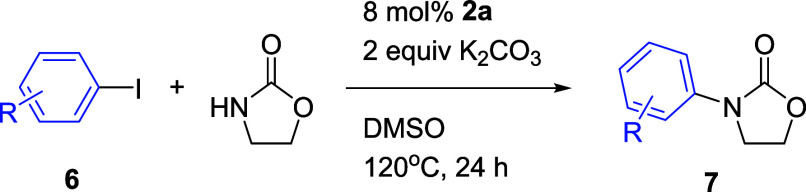

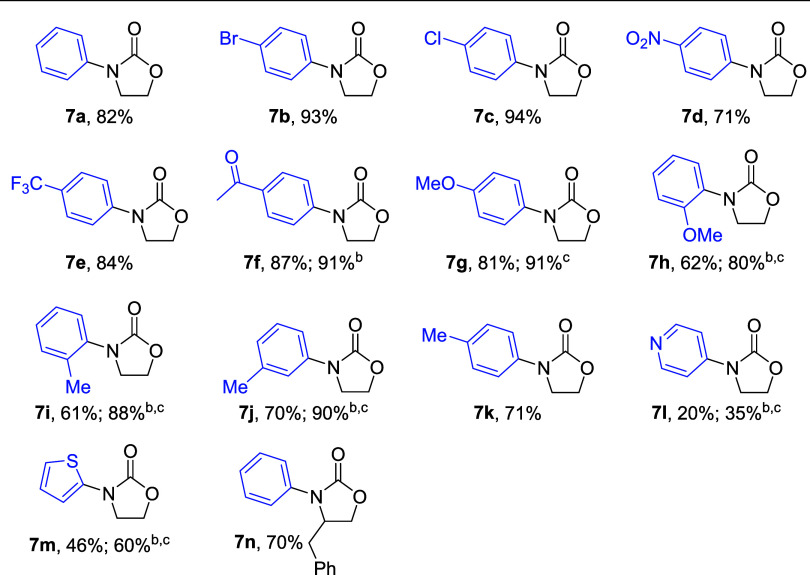
Scope of Iodoarenes in the *N*-arylation of Oxazolidinone with Cu-NHC **2a** ^a^

a **6** (1 mmol), oxazolidinone (1.3 mmol), Cu-NHC **2a** (8 mol %), K_2_CO_3_ (2 mmol), and DMSO (1.5 mL) were stirred for 24 h at 120 °C under N_2_.

b Cu-NHC **2a** (10 mol %) was used.

c The reaction was stirred for 24 h at 140 °C.

The developed catalytic system was also applied to the coupling of other nucleophiles, such as secondary or primary amides (Table 5). The Cu-catalyzed aryl amidation of cyclic secondary amides **8a** and **8b** with iodobenzene generally resulted in complete conversion under 10 mol % Cu-NHC **2a** at 140 °C. The aryl primary amides **8c**–**8e** coupled with iodobenzene, affording the corresponding products **9c**–**9e** in moderate to good yields. Notably, the primary amide **8d**, with a hydroxy group at the ortho position of the benzene ring, afforded **9d** in 90% yield under 10 mol % Cu-NHC **2a** at 140 °C. The *ortho*-substituted amide **8c** has been a difficult coupling partner for many previous catalytic systems. The use of Cu-NHC **2a** offers a promising solution to this problem. However, aliphatic amides **8f** and **8g** were found to be unsuitable for the Cu-catalyzed aryl amidation, resulting in poor yields of *N*-arylated products **9f** and **9g**. Good tolerance was observed when nicotinamide **8h** and isonicotinamide **8i** were used as coupling partners.

**Table 5:**
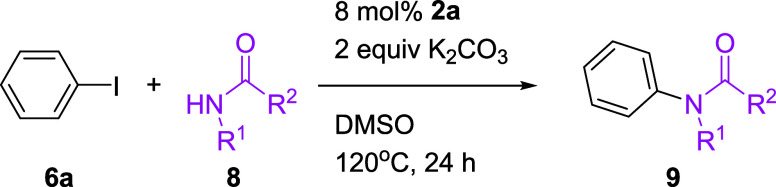

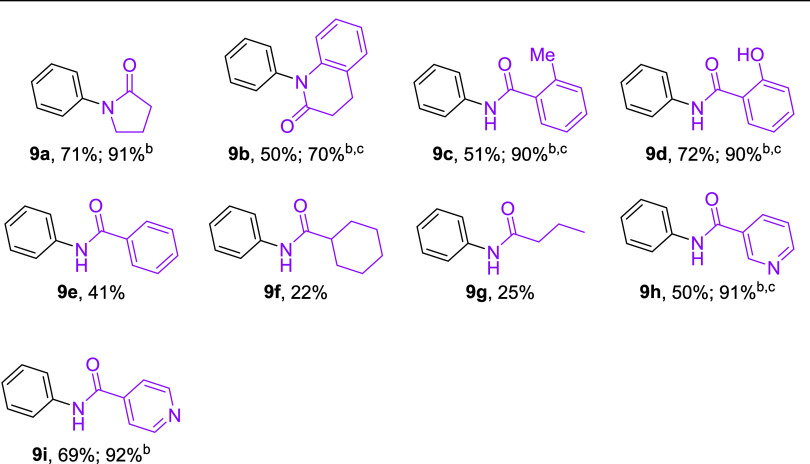
Coupling with Other Nitrogen Nucleophiles^a^

a **6a** (1 mmol), **8** (1.3 mmol), Cu-NHC **2a** (8 mol %), K_2_CO_3_ (2 mmol), and DMSO (1.5 mL) were stirred for 24 h at 120 °C under N_2_.

b The reaction was stirred for 24 h at 140 °C.

c Cu-NHC **2a** (10 mol %) was used.

## Conclusions

In summary, we have successfully accomplished the synthesis, spectroscopic characterization, and structural analysis of a series of Cu-NHC complexes. Based on crystal diffraction analysis, complex **2a** adopts a linear geometry. These Cu-NHC complexes exhibited stability in the solid state under a nitrogen atmosphere, with Cu-NHC **2a** remaining stable for up to 6 months even in the presence of air. The preparative procedure of these Cu-NHC complexes is straightforward, utilizing readily available starting materials. Furthermore, the catalytic potential of these Cu-NHC complexes was explored for two key transformations: Cu-catalyzed hydrosilylation of the carbonyl compounds and C–N coupling of iodoarenes with oxazolidinones and amides. Among these, complex **2a** exhibited the highest catalytic activity, showing good tolerance toward various functional groups and affording the desired products in good yields. The findings indicate that indole-substituted NHC–Cu complexes are promising catalysts for organic transformations, highlighting their potential application in various catalytic processes.

## Supplementary Material



## Data Availability

The data underlying this study are available in the published article and its Supporting Information.

## Notes

General experimental procedures, optimization of the reaction conditions, ^1^H and ^13^C NMR spectra of all the products (2, 3, 5, 7, and 9), and X-ray crystallographic analysis of Cu-NHC **2a** (PDF)
