# Supplementary material for: Development of New Indole-Based N‑Heterocyclic Carbene Copper Complexes and Their Applications in Catalysis
Source: J Org Chem. 2025 May 28;90(22):7236–45. doi: 10.1021/acs.joc.5c00248 (PMC12150340; doi:10.1021/acs.joc.5c00248)
Supplement: Supplementary file 1 [file jo5c00248_si_001.pdf]

Supporting Information

Development of New Indole-Based *N*-Heterocyclic Carbene Copper Complexes and Their Applications in Catalysis

Chun-Fa Lin, Ya-Hsuan Tseng, Hui-Yu Hsieh, Pei-Jei Hung, Yan-Wen Huang, Dong-Sheng Lee, and Ta-Jung Lu*

Department of Chemistry, National Chung-Hsing University, Taichung, 402, Taiwan

Fax: 886-4-22862547; E-Mail: dslee@mail.nchu.edu.tw

Table of Contents

1. Experimental Section …… S2
   1.1. General aspects …… S2
   1.2. Experimental procedures and spectral data
      1.2.1. Synthesis of Complexes **2** …… S2
      1.2.2. Application in the Cu-catalyzed hydrosilylation of carbonyls
         1.2.2.1. Table S1 …… S5
         1.2.2.2. General procedure for Table 2 …… S6
      1.2.3. Application in the Ullmann-type reaction
         1.2.3.1. Table S2 …… S10
         1.2.3.2 General procedure for Table 4 and Table 5 …… S11
2. References and notes …… S16
3. ^{1}H and ^{13}C-NMR Spectra of Compounds **2**, **3**, **5**, **7**, and **8** …… S17
4. X-ray crystallographic analysis …… S68

1. Experimental Section

1.1. General Aspects. Unless otherwise noted, commercially available materials were purchased from Alfa Aesar, Aldrich, and Acros, and were used without further purification. $CHCl_3$ was obtained by distillation over calcium hydride. Toluene and THF were distillated and used after treatment with sodium. Reactions were monitored with pre-coated silica gel 60 (F-254) plates. The purification of products was performed by column chromatography (silica gel, 0.040–0.063 μm) eluting with *n*-hexane/ethyl acetate. ^{1}H and ^{13}C NMR spectra were analyzed at an Agilent Mercury 400 spectrometer or JEOL 400 spectrometer. *J*-values are given in Hz. Chemical shifts (δ) were recorded from $CDCl_3$ (δ = 7.26 ppm) in the ^{1}H NMR spectra and the central peak of $CDCl_3$ (δ = 77.0 ppm) in the ^{13}C NMR spectra. Melting points were determined with a Thermo 1001D digital melting point apparatus and are uncorrected. High resolution mass spectra were obtained with a Finnigan/Thermo Quest MAT 95XL mass spectrometer using either electron impact (EI), matrix-assisted laser desorption/ionization Time-of-Flight (MALDI-TOF) method, or electrospray ionization (ESI) method. Suitable crystal of copper complex **2a** was mounted onto a glass fiber by using perfluoropolyether oil. Diffraction data was collected at 150 K using a Bruker APEX2 diffractometer. Salts **1** were prepared according to our previous reports.[1,2,3]

1.2. Experimental procedures and spectral data

1.2.1. Synthesis of Complexes 2

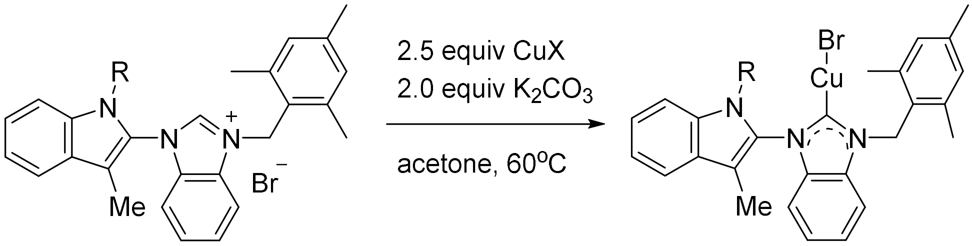

1 Cu-NHC **2**

All manipulations were carried out under nitrogen using dried solvent. To a Schleck tube was charged with **1** (0.26 mmol, 1.0 equiv), CuBr (0.63 mmol, 2.5 equiv), K_2CO_3 (0.51 mmol, 2.0 equiv), and acetone (4 mL) . After stirring at 60°C on an oil bath for 24 h, the reaction mixture was cooled to room temperature then filtered through silica. The filtrate was washed with dichloromethane (3 × 10 mL). The solvent was evaporated under reduced pressure to give the desired products.

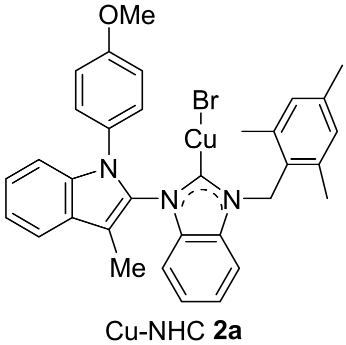

Cu-NHC **2a**

(1-(1-(4-Methoxyphenyl)-3-methyl-1H-indol-2-yl)-3-(2,4,6-trimethylbenzyl)-2,3-dihydro-1H-benzo[d]imidazol-2-yl)copper(I) bromide (2a). A mixture of **1a** (0.15 g, 0.26 mmol), CuBr (0.09 g, 0.63 mmol), K_2CO_3 (0.07 g, 0.51 mmol), and acetone (4 mL) were used to obtain **2a** as a white solid (0.16 g, 97%). Mp = 214.9–231.6°C. ^{1}H NMR (400 MHz, $CDCl_3$): δ 7.70 (d, *J* = 7.0 Hz, 1H), 7.34–7.26 (m, 6H), 7.24–7.20 (m, 2H), 7.17–7.11 (m, 2H), 6.89 (s, 2H), 6.79 (br, 1H), 5.56, 5.47 (ABq, *J* = 15.2 Hz, 2H), 3.79 (s, 3H), 2.31 (s, 3H), 2.21 (s, 3H), 2.08

(s, 6H); $^{13}C\{^1H\}$ NMR (101 MHz, $CDCl_3$): δ 187.3, 159.1, 139.2, 137.4, 136.5, 135.7, 132.6, 130.1, 128.2, 127.7, 126.5, 125.0, 124.5, 123.9, 120.5, 119.6, 114.5, 111.9, 111.8, 110.8, 109.1, 55.4, 48.2, 21.0, 20.3, 8.4; HRMS-EI (m/z) [M^+]: calcd for $C_{33}H_{31}BrCuN_3O$: 627.0947, found: 627.0938; Anal. calcd for $C_{33}H_{31}BrCuN_3O$: C, 63.01; H, 4.97; N, 6.68, found: C, 62.24; H, 4.96; N, 6.52.

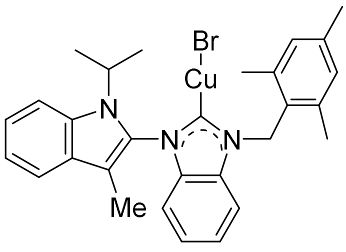
Cu-NHC **2b**

(1-(1-Isopropyl-3-methyl-1H-indol-2-yl)-3-(2,4,6-trimethylbenzyl)-2,3-dihydro-1H-benzo[d]imidazol-2-yl)copper(I) bromide (2b). A mixture of **1b** (0.15 g, 0.30 mmol), CuBr (0.11 g, 0.77 mmol), K_2CO_3 (0.08 g, 0.58 mmol) and acetone (4 mL) were used to obtain **2b** as a white solid (0.16 g, 91%). Mp = 225.9–238.5°C. ^1H NMR (400 MHz, $CDCl_3$): δ 7.65 (d, J = 8.0 Hz, 1H), 7.57 (d, J = 8.4 Hz, 1H), 7.35–7.29 (m, 3H), 7.28–7.24 (m, 1H), 7.20 (t, J = 7.4 Hz, 1H), 7.08–7.06 (m, 1H), 6.98 (s, 2H), 5.72 (s, 2H), 3.98 (septet, J = 7.0 Hz, 1H), 2.35 (s, 6H), 2.33 (s, 3H), 2.11 (s, 3H), 1.58 (d, J = 7.0 Hz, 3H), 1.49 (d, J = 7.0 Hz, 3H); $^{13}C\{^1H\}$ NMR (101 MHz, $CDCl_3$): δ 188.0, 139.4, 137.4, 135.9, 133.2, 132.8, 130.3, 127.4, 126.7, 126.6, 125.1, 124.8, 122.9, 120.0, 119.5, 112.0, 108.5, 48.5, 47.1, 21.9, 21.0, 20.5, 8.1; HRMS-EI (m/z) [M^+]: calcd for $C_{29}H_{31}BrCuN_3$: 563.0997, found: 563.1003; Anal. calcd for $C_{29}H_{31}BrCuN_3$: C, 61.65; H, 5.53; N, 7.44, found: C, 60.61; H, 5.39; N, 7.14.

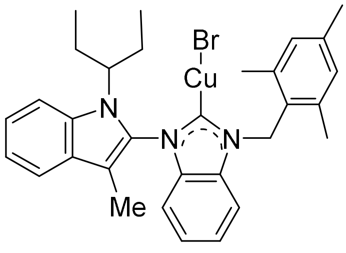
Cu-NHC **2c**

(1-(3-Methyl-1-(pentan-3-yl)-1H-indol-2-yl)-3-(2,4,6-trimethylbenzyl)-2,3-dihydro-1H-benzo[d]imidazol-2-yl)copper(II) bromide (2c). A mixture of **1c** (0.15 g, 0.28 mmol), CuBr (0.10 g, 0.70 mmol), K_2CO_3 (0.08 g, 0.58 mmol), and acetone (4 mL) were used to obtain **2c** as a white solid (0.16 g, 98%). Mp = 208.9–222.1°C. ^1H NMR (400 MHz, $CDCl_3$): δ 7.65 (d, J = 7.8 Hz, 1H), 7.51 (d, J = 8.6 Hz, 1H), 7.34–7.30 (m, 2H), 7.28–7.22 (m, 2H), 7.17 (t, J = 8.0 Hz, 1H), 7.10–7.07 (m, 1H), 6.97 (s, 2H), 5.78, 5.73 (ABq, J = 14.4 Hz, 2H), 3.34 (br, 1H), 2.35 (s, 6H), 2.33 (s, 3H), 2.12–2.02 (m, 2H), 2.07 (s, 3H), 1.92–1.84 (m, 2H), 0.87 (t, J = 7.0 Hz, 3H), 0.56 (t, J = 7.0 Hz, 3H); $^{13}C\{^1H\}$ NMR (101 MHz, $CDCl_3$): δ 188.1, 139.2, 137.3, 135.7, 133.4, 132.8, 130.3, 127.8, 127.0, 126.6, 124.8, 124.7, 122.8, 119.8, 119.4, 112.2, 111.9, 108.2, 59.8, 48.4, 27.4, 26.0, 21.0, 20.5, 12.6, 11.9, 8.1; HRMS-EI (m/z) [M^+]: calcd for $C_{31}H_{35}BrCuN_3$: 591.1310, found: 591.1316; Anal. calcd for $C_{31}H_{35}BrCuN_3$: C, 62.67; H, 6.11; N, 7.07, found: C, 60.18; H, 5.50; N, 6.74.

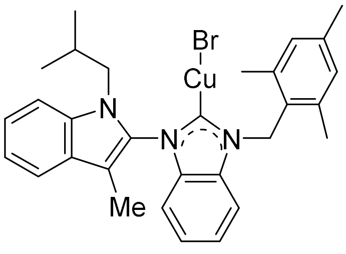
Cu-NHC **2d**

(1-(1-Isobutyl-3-methyl-1H-indol-2-yl)-3-(2,4,6-trimethylbenzyl)-2,3-dihydro-1H-benzo[d]imidazol-2-yl)copper(II) bromide (2d). A mixture of **1d** (0.15 g, 0.29 mmol), CuBr (0.10 g, 0.70 mmol), K_2CO_3 (0.08 g, 0.58 mmol), and acetone (4 mL) were used to obtain **2d** as a white solid (0.16 g, 95%). Mp = 217.6–221.1°C. ^1H NMR (400 MHz, $CDCl_3$): δ 7.64 (d, J = 8.0 Hz, 1H), 7.38–7.31 (m, 4H), 7.25–7.18 (m, 2H), 7.09–7.07 (m, 1H), 6.99 (s, 2H), 5.72, 5.66 (ABq, J = 14.4 Hz, 2H), 3.91 (dd, J = 14.8, 7.6 Hz, 1H), 3.55 (dd, J = 14.8, 7.6

Hz, 1H), 2.34 (s, 9H), 2.12 (s, 3H), 1.95 (septet, *J* = 7.6 Hz, 1H), 0.73 (d, *J* = 7.6 Hz, 3H), 0.64 (d, *J* = 7.6 Hz, 3H); $^{13}C\{^{1}H\}$ NMR (101 MHz, $CDCl_3$): δ 188.0, 139.1, 137.3, 135.4, 135.0, 132.7, 130.2, 127.5, 126.6, 126.4, 125.0, 124.7, 123.0, 119.6, 112.0, 111.9, 110.3, 108.2, 50.8, 48.3, 29.2, 21.0, 20.4, 20.2, 8.3; HRMS-EI (m/z) [M^+]: calcd for $C_{30}H_{33}BrCuN_3$: 577.1154, found: 577.1159. Anal. calcd for $C_{30}H_{33}BrCuN_3$: C, 62.12; H,5.91; N, 7.24, found: C, 60.64; H, 5.76; N, 6.88.

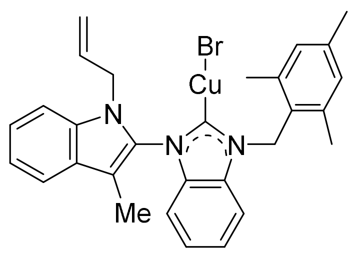

Cu-NHC **2e**

(1-(1-Allyl-3-methyl-1H-indol-2-yl)-3-(2,4,6-trimethylbenzyl)-2,3-dihydro-1H-benzo[d]imidazol-2-yl)copper(II) bromide (2e). A mixture of **1e** (0.15 g, 0.30 mmol), CuBr (0.10 g, 0.70 mmol), K_2CO_3 (0.08 g, 0.58 mmol), and acetone (4 mL) were used to obtain **2e** as a white solid (0.15 g, 89%). Mp = 215.2–218.7°C. ^{1}H NMR (400 MHz, $CDCl_3$): δ 7.66 (d, *J* = 7.8 Hz, 1H), 7.36–7.30 (m, 4H), 7.24–7.20 (m, 2H), 7.12–7.08 (m, 1H), 6.97 (s, 2H), 5.75–5.65 (m, 1H), 5.70 (s, 2H), 5.04 (d, *J* = 10.0 Hz, 1H), 4.80 (d, *J* = 10.0 Hz, 1H), 4.61 (d, *J* = 17.6 Hz, 1H), 4.30 (d, *J* = 17.6 Hz, 1H), 2.34 (s, 9H), 2.14 (s, 3H); $^{13}C\{^{1}H\}$ NMR (101 MHz, $CDCl_3$): δ 188.4, 139.1, 137.3, 135.3, 134.8, 132.8, 131.5, 130.2, 127.2, 126.6, 124.9, 124.7, 123.4, 119.9, 119.7, 116.4, 111.9, 110.1, 108.6, 48.3, 45.2, 21.0, 20.5, 8.2; HRMS-EI (m/z) [M^+]: calcd for $C_{29}H_{29}BrCuN_3$: 561.0841, found: 561.0850; Anal. calcd for $C_{29}H_{29}BrCuN_3$: C, 61.76; H, 5.36; N, 7.45, found: C, 60.21; H, 5.30; N, 6.98.

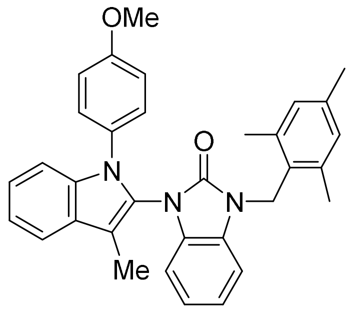

3

1-(1-(4-Methoxyphenyl)-3-methyl-1H-indol-2-yl)-3-(2,4,6-trimethylbenzyl)-1,3-dihydro-2H-benzo[d]imidazol-2-one (3). The compound **2a** decomposed to obtain **3** as a brown solid. Mp = 189.6–196.2°C. ^{1}H NMR (400 MHz, $CDCl_3$): δ 7.69 (d, *J* = 7.0 Hz, 1H), 7.26–7.21 (m, 5H), 6.89–6.81 (m, 4H), 6.84 (s, 2H), 6.71 (d, *J* = 8.0 Hz, 1H), 6.32 (d, *J* = 8.0 Hz, 1H), 5.14, 5.01 (ABq, *J* = 15.2 Hz, 2H), 3.80 (s, 3H), 2.27 (s, 3H), 2.22 (s, 3H), 2.16 (s, 6H); $^{13}C\{^{1}H\}$ NMR (101 MHz, $CDCl_3$): δ 158.8, 153.4, 137.5, 137.3, 136.5, 130.1, 129.5, 129.0, 128.7, 128.0, 127.0, 125.4, 123.2, 122.0, 121.3, 119.9, 119.3, 114.4, 110.5, 109.7, 108.9, 108.4, 55.3, 40.8, 20.8, 20.0, 8.2; HRMS-EI (m/z) [M^+]: calcd for $C_{33}H_{31}N_3O_2$: 501.2416, found: 501.2424.

1.2.2. Application in the Cu-catalyzed hydrosilylation of carbonyls

1.2.2.1. Table S1

Table S1. Screening optimization of reaction conditions using a Taguchi L9 array. [a]

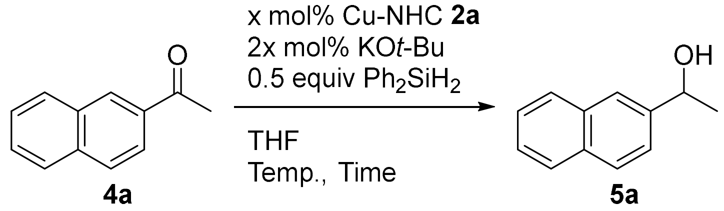

Entry	**2a** (mol%)	Base (mol%)	Silane (equiv)	Solvent	Temp. (°C)	Time (h)	NMR ratio[b] **4a**:**5a**
1	1.0	KOH (2.0)	Ph_2SiH_2 (0.5)	Toluene	60	24	19:81 (77)
2	1.0	K_2CO_3 (2.0)	Et_3SiH (1.0)	Toluene	60	24	99:1
3	1.0	KO^{t}Bu (2.0)	$PhMe_2SiH$ (1.0)	Toluene	60	24	90:10
4	1.0	KOH (2.0)	Et_3SiH (1.0)	THF	60	24	95:5
5	1.0	K_2CO_3 (2.0)	$PhMe_2SiH$ (1.0)	THF	60	24	92:8
6	1.0	KO^{t}Bu (2.0)	Ph_2SiH_2 (0.5)	THF	60	24	13:87 (82)
7	1.0	KOH (2.0)	$PhMe_2SiH$ (1.0)	$CHCl_3$	60	24	99:1
8	1.0	K_2CO_3 (2.0)	Ph_2SiH_2 (0.5)	$CHCl_3$	60	24	89:11
9	1.0	KO^{t}Bu (2.0)	Et_3SiH (1.0)	$CHCl_3$	60	24	99:1
10	1.0	KO^{t}Bu (2.0)	Ph_2SiH_2 (0.6)	THF	60	24	> 1:99 (95)
11	1.0	KO^{t}Bu (2.0)	Ph_2SiH_2 (0.6)	THF	40	24	> 1:99 (97)
12	1.0	KO^{t}Bu (2.0)	Ph_2SiH_2 (0.6)	THF	rt	24	9:91
13	1.0	KO^{t}Bu (2.0)	Ph_2SiH_2 (0.6)	THF	40	4	> 1:99 (95)
14	1.0	KO^{t}Bu (2.0)	Ph_2SiH_2 (0.6)	THF	40	2	7:93
15	1.0	KO^{t}Bu (2.0)	Ph_2SiH_2 (0.6)	THF	40	1	53:47
16	0.5	KO^{t}Bu (1.0)	Ph_2SiH_2 (0.6)	THF	40	4	> 1:99 (98)
17	0.25	KO^{t}Bu (0.5)	Ph_2SiH_2 (0.6)	THF	40	4	64:36
18	–	KO^{t}Bu (1.0)	Ph_2SiH_2 (0.6)	THF	40	4	> 99:1

[a] The reaction was carried out on a 1 mmol scale of **4a**. [b] The ratio was determined by 400 MHz NMR. Isolated yield of **5a** was shown in parentheses.

1.2.2.2. General procedure for Table 2

All manipulations were carried out under N_2. To a Schleck tube was charged with ketone or aldehyde (1.0 mmol, 1.0 equiv), **2a** (0.005 mmol, 0.5 mol%), KO^{t}Bu (0.01 mmol, 1 mol%), diphenylsilane (0.6 mmol, 0.6 equiv), and THF (3 mL). After stirring at 40°C on an oil bath for 4 h, the reaction mixture was cooled to room temperature. The resulting solution was added TBAF (1 mL, 1 M) and stirred for 1 h. The resulting mixture was added to H_2O (10 mL). The aqueous layer was extracted with EtOAc (10 mL × 3). The combined organic layers were washed with water (15 mL x 3), dried over anhydrous Na_2SO_4, and then filtered through celite. The solvent was evaporated under reduced pressure. The residue was purified by column chromatography on a 200–400 mesh silica gel using 10% ethyl acetate in *n*-hexane as the eluent to get the final product **5**.

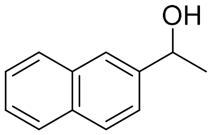

5a

1-(Naphthalen-2-yl)ethan-1-ol (5a).[4] A mixture of **4a** (0.17 g, 1.00 mmol), **2a** (3.10 mg, 0.005 mmol), KO^{t}Bu (1.12 mg, 0.01 mmol), and THF (3 mL) were used. 5a (0.17 g, 98%) was obtained by column chromatography (SiO_2; *n*-hexane:EtOAc; 95:5) as white solid. ^{1}H NMR (400 MHz, $CDCl_3$): δ 7.86–7.82 (m, 4H), 7.53–7.45 (m, 3H), 5.08 (q, *J* = 6.8 Hz, 1H), 1.93 (s, 1H, OH), 1.58 (d, *J* = 6.8 Hz, 3H); $^{13}C\{^1H\}$ NMR (101 MHz, $CDCl_3$): δ 143.0, 133.0, 132.5, 127.8, 127.6, 127.3, 125.7, 125.3, 123.6, 123.5, 69.8, 24.8. Spectroscopic data consistent with literature.[4]

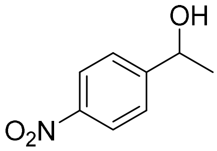

5b

1-(4-Nitrophenyl)ethan-1-ol (5b).[5] A mixture of **4b** (0.17 g, 1.00 mmol), **2a** (3.10 mg, 0.005 mmol), KO^{t}Bu (1.12 mg, 0.01 mmol), and THF (3 mL) were used. **5a** (0.15 g, 90%) was obtained by column chromatography (SiO_2; *n*-hexane:EtOAc; 70:30) as brown liquid. ^{1}H NMR (400 MHz, $CDCl_3$): δ 8.21 (d, *J* = 8.6 Hz, 2H), 7.55 (d, *J* = 8.6 Hz, 2H), 5.03 (q, *J* = 6.6 Hz, 1H), 2.00 (s, 1H, OH), 1.53 (d, *J* = 6.6 Hz, 3H); $^{13}C\{^1H\}$ NMR (101 MHz, $CDCl_3$): δ 153.2, 146.7, 126.0, 123.4, 69.0, 25.1. Spectroscopic data consistent with literature.[5]

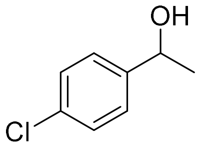

5c

1-(4-Chlorophenyl)ethan-1-ol (5c).[5] A mixture of **4c** (0.15 g, 1.00 mmol), **2a** (3.10 mg, 0.005 mmol), KO^{t}Bu (1.12 mg, 0.01 mmol), and THF (3 mL) were used. **5c** (0.15 g, 98%) was obtained by column chromatography (SiO_2; *n*-hexane:EtOAc; 95:5) as colorless liquid. ^{1}H NMR (400 MHz, $CDCl_3$): δ 7.32 (s, 4H), 4.89 (q, *J* = 6.4 Hz, 1H), 1.82 (s, 1H, OH), 1.48 (d, *J* = 6.4 Hz, 3H); $^{13}C\{^1H\}$ NMR (101 MHz, $CDCl_3$): δ 144.1, 132.8, 128.3, 126.6, 69.4, 25.0. Spectroscopic data consistent with literature.[5]

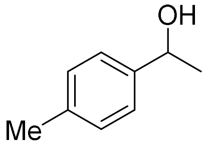

5d

1-(p-Tolyl)ethan-1-ol (5d).[6] A mixture of **4d** (0.13 g, 1.00 mmol), **2a** (3.10 mg, 0.005 mmol), KO^{t}Bu (1.12 mg, 0.01 mmol), and THF (3 mL) were used. **5d** (0.13 g, 95%) was obtained by column chromatography (SiO_2; *n*-hexane:EtOAc; 95:5) as colorless liquid. ^{1}H NMR (400 MHz, $CDCl_3$): δ 7.27 (d, *J* = 8.4 Hz, 2H), 7.16 (d, *J* = 8.4 Hz, 2H), 4.87 (q, *J* = 6.4 Hz, 1H), 2.34 (s, 3H), 1.75 (s, 1H, OH), 1.49 (d, *J* = 6.4 Hz, 3H); $^{13}C\{^1H\}$ NMR (101 MHz, $CDCl_3$): δ 142.8, 136.6, 128.9, 125.2, 69.7, 24.8, 20.8. Spectroscopic data consistent

with literature.[6]

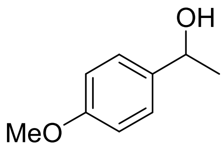
5e

1-(4-Methoxyphenyl)ethan-1-ol (5e).[5] A mixture of **4e** (0.15 g, 1.00 mmol), **2a** (3.10 mg, 0.005 mmol), KO^{t}Bu (1.12 mg, 0.01 mmol), and THF (3 mL) were used. **5e** (0.15 g, 98%) was obtained by column chromatography (SiO_2; *n*-hexane:EtOAc; 95:5) as yellow liquid. ^{1}H NMR (400 MHz, $CDCl_3$): δ 7.31 (d, *J* = 8.4 Hz, 2H), 6.89 (d, *J* = 8.4 Hz, 2H), 4.86 (q, *J* = 6.4 Hz, 1H), 3.80 (s, 3H), 1.74 (s, 1H, OH), 1.48 (d, *J* = 6.4 Hz, 3H); $^{13}C\{^1H\}$ NMR (101 MHz, $CDCl_3$): δ 158.7, 137.9, 126.5, 113.6, 69.6, 55.1, 24.8. Spectroscopic data consistent with literature.[5]

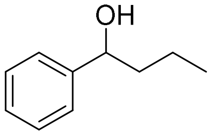
5f

1-Phenylbutan-1-ol (5f).[6] A mixture of **4f** (0.15 g, 1.00 mmol), **2a** (3.10 mg, 0.005 mmol), KO^{t}Bu (1.12 mg, 0.01 mmol), and THF (3 mL) were used. **5f** (0.12 g, 80%) was obtained by column chromatography (SiO_2; *n*-hexane:EtOAc; 95:5) as colorless liquid. ^{1}H NMR (400 MHz, $CDCl_3$): δ 7.37–7.32 (m, 4H), 7.30–7.25 (m, 1H), 4.68 (t, *J* = 7.6 Hz, 1H), 1.84 (s, 1H, OH), 1.82–1.64 (m, 2H),1.48–1.26 (m, 2H), 0.93 (t, *J* = 7.4 Hz, 3H); $^{13}C\{^1H\}$ NMR (101 MHz, $CDCl_3$): δ 144.9, 128.2, 127.2, 125.8, 74.2, 41.1, 18.9, 13.9. Spectroscopic data consistent with literature.[6]

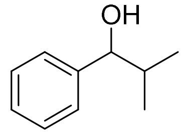
5g

2-Methyl-1-phenylpropan-1-ol (5g).[6] A mixture of **4g** (0.15 g, 1.00 mmol), **2a** (3.10 mg, 0.005 mmol), KO^{t}Bu (1.12 mg, 0.01 mmol), and THF (3 mL) were used. **5g** (0.12 g, 82%) was obtained by column chromatography (SiO_2; *n*-hexane:EtOAc; 95:5) as colorless liquid. ^{1}H NMR (400 MHz, $CDCl_3$): δ 7.36–7.25 (m, 5H), 4.36 (d, *J* = 6.8 Hz, 1H), 1.96 (octet, *J* = 6.8 Hz, 1H), 1.84 (s, 1H, OH), 1.0 (d, *J* = 6.8 Hz, 3H), 0.8 (d, *J* = 6.8 Hz, 3H), $^{13}C\{^1H\}$ NMR (101 MHz, $CDCl_3$): δ 143.5, 128.0, 127.2, 126.5, 79.8, 35.1, 18.8, 18.2. Spectroscopic data consistent with literature.[6]

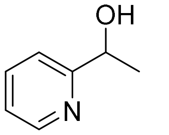
5h

1-(Pyridin-2-yl)ethan-1-ol (5h).[5] A mixture of **4h** (0.12 g, 1.00 mmol), **2a** (3.10 mg, 0.005 mmol), KO^{t}Bu (1.12 mg, 0.01 mmol), and THF (3 mL) were used. **5h** (0.10 g, 81%) was obtained by column chromatography (SiO_2; *n*-hexane:EtOAc; 70:30) as yellow liquid. ^{1}H NMR (400 MHz, $CDCl_3$): δ 8.54 (d, *J* = 4.0 Hz, 1H), 7.70 (td, *J* = 7.6, 1.6 Hz, 1H), 7.28 (d, *J* = 7.6 Hz, 1H), 7.22–7.18 (m, 1H), 4.90 (q, *J* = 6.4 Hz, 1H), 1.51 (d, *J* = 6.4 Hz, 3H); $^{13}C\{^1H\}$ NMR (101 MHz, $CDCl_3$): δ 163.3, 147.8, 136.8, 122.1, 119.7, 69.0, 23.9. Spectroscopic data consistent with literature.[5]

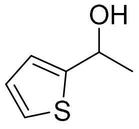
5i

1-(Thiophen-2-yl)ethan-1-ol (5i).[7] A mixture of **4i** (0.13 g, 1.00 mmol), **2a** (3.10 mg, 0.005 mmol), KO^{t}Bu (1.12 mg, 0.01 mmol), and THF (3 mL) were used. **5i** (0.09 g, 70%) was obtained by column chromatography (SiO_2; *n*-hexane:EtOAc; 80:20) as yellow liquid. ^{1}H NMR (400 MHz, $CDCl_3$): δ 7.24 (dd, *J* = 4.8, 1.6 Hz 1H), 7.01–6.94 (m, 2H), 5.17–5.11 (m, 1H), 1.61 (d, *J* = 6.4 Hz, 3H); $^{13}C\{^1H\}$ NMR (101 MHz, $CDCl_3$): δ 149.7, 126.4, 124.1, 123.0, 65.9, 25.0. Spectroscopic data consistent with literature.[7]

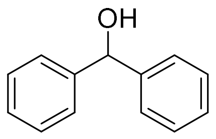

5j

Diphenylmethanol (5j).[5] A mixture of **4j** (0.18 g, 1.00 mmol), **2a** (3.10 mg, 0.005 mmol), KO^{t}Bu (1.12 mg, 0.01 mmol), and THF (3 mL) were used. **5j** (0.18 g, 97%) was obtained by column chromatography (SiO_2; *n*-hexane:EtOAc; 95:5) as white solid. ^{1}H NMR (400 MHz, $CDCl_3$): δ 7.39–7.31 (m, 8H), 7.28–7.24 (m, 2H), 5.85 (s, 1H), 2.21 (s, 1H, OH); ^{13}C{^{1}H} NMR (101 MHz, $CDCl_3$): δ 143.6, 128.2, 127.2, 126.4, 75.8. Spectroscopic data consistent with literature.[5]

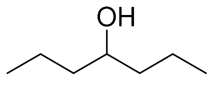

5k

4-Heptanol (5k).[8] A mixture of **4k** (0.11 g, 1.00 mmol), **2a** (3.10 mg, 0.005 mmol), KO^{t}Bu (1.12 mg, 0.01 mmol), and THF (3 mL) were used. **5k** (0.089 g, 77%) was obtained by column chromatography (SiO_2; *n*-hexane:EtOAc; 95:5) as colorless liquid. ^{1}H NMR (400 MHz, $CDCl_3$): δ 3.62 (br, 1H), 1.50–1.32 (m, 8H), 1.29 (s, 1H, OH), 0.93 (t, J = 7.0 Hz, 6H); ^{13}C{^{1}H} NMR (101 MHz, $CDCl_3$): δ 71.3, 39.6, 18.7, 14.0. Spectroscopic data consistent with literature.[8]

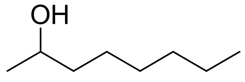

5l

2-Octanol (5l).[9] A mixture of **4l** (0.13 g, 1.00 mmol), **2a** (3.10 mg, 0.005 mmol), KO^{t}Bu (1.12 mg, 0.01 mmol), and THF (3 mL) were used. **5l** (0.10 g, 78%) was obtained by column chromatography (SiO_2; *n*-hexane:EtOAc; 95:5) as colorless liquid. ^{1}H NMR (400 MHz, $CDCl_3$): δ 3.79 (br, 1H), 1.48–1.39 (m, 2H), 1.29–1.24 (br, 8H), 1.19 (d, J = 6.0 Hz, 3H), 0.89 (t, J = 6.8 Hz, 3H); ^{13}C{^{1}H} NMR (101 MHz, $CDCl_3$): 68.1, 39.3, 31.7, 29.2, 25.6, 23.3, 22.5, 14.0. Spectroscopic data consistent with literature.[9]

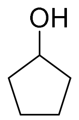

5m

Cyclopentanol (5m).[6] A mixture of **4m** (0.084 g, 1.00 mmol), **2a** (3.10 mg, 0.005 mmol), KO^{t}Bu (1.12 mg, 0.01 mmol), and THF (3 mL) were used. **5m** (0.078 g, 91%) was obtained by column chromatography (SiO_2; *n*-hexane:EtOAc; 95:5) as colorless liquid. ^{1}H NMR (400 MHz, $CDCl_3$): δ 4.35–4.33 (br, 1H), 1.80–1.70 (m, 4H), 1.60–1.50 (m, 4H), 1.34 (br, 1H, OH); ^{13}C{^{1}H} NMR (101 MHz, $CDCl_3$): δ 73.7, 35.3, 23.1. Spectroscopic data consistent with literature.[6]

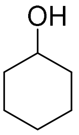

5n

Cyclohexanol (5n).[6] A mixture of **4n** (0.098 g, 1.00 mmol), **2a** (3.10 mg, 0.005 mmol), KO^{t}Bu (1.12 mg, 0.01 mmol), and THF (3 mL) were used. **5n** (0.088 g, 88%) was obtained by column chromatography (SiO_2; *n*-hexane:EtOAc; 95:5) as colorless liquid. ^{1}H NMR (400 MHz, $CDCl_3$): δ 4.35–4.33 (br, 1H), 1.80–1.70 (m, 4H), 1.60–1.50 (m, 4H), 1.34 (br, 1H, OH); ^{13}C{^{1}H} NMR (101 MHz, $CDCl_3$): δ 73.7, 35.3, 23.1. Spectroscopic data consistent with literature.[6]

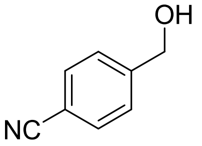

5o

4-(Hydroxymethyl)benzonitrile (5o).[6] A mixture of **4o** (0.13 g, 1.00 mmol), **2a** (3.10 mg, 0.005 mmol), KO^{t}Bu (1.12 mg, 0.01 mmol), and THF (3 mL) were used. **5o** (0.12 g, 87%) was obtained by column chromatography (SiO_2; *n*-hexane:EtOAc; 95:5) as colorless liquid. ^{1}H NMR (400 MHz, $CDCl_3$): δ 4.35–4.33 (br, 1H), 1.80–1.70 (m, 4H), 1.60–1.50 (m, 4H), 1.34 (br, 1H, OH); ^{13}C{^{1}H} NMR (101 MHz, $CDCl_3$): δ 73.7, 35.3, 23.1. Spectroscopic data consistent with literature.[6]

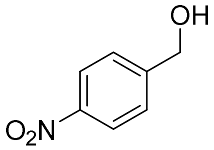

5p

(4-Nitrophenyl)methanol (5p).[6] A mixture of **4p** (0.15 g, 1.00 mmol), **2a** (3.10 mg, 0.005 mmol), KO^tBu (1.12 mg, 0.01 mmol), and THF (3 mL) were used. **5p** (0.15 g, 98%) was obtained by column chromatography (SiO_2; *n*-hexane:EtOAc; 95:5) as colorless liquid. ^1H NMR (400 MHz, $CDCl_3$): δ 4.35–4.33 (br, 1H), 1.80–1.70 (m, 4H), 1.60–1.50 (m, 4H), 1.34 (br, 1H, OH); $^{13}C\{^1H\}$ NMR (101 MHz, $CDCl_3$): δ 73.7, 35.3, 23.1. Spectroscopic data consistent with literature.[6]

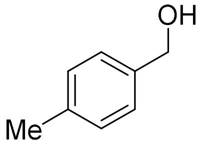

5q

(4-Methylphenyl)methanol (5q).[6] A mixture of **4q** (0.12 g, 1.00 mmol), **2a** (3.10 mg, 0.005 mmol), KO^tBu (1.12 mg, 0.01 mmol), and THF (3 mL) were used. **5q** (0.094 g, 77%) was obtained by column chromatography (SiO_2; *n*-hexane:EtOAc; 95:5) as colorless liquid. ^1H NMR (400 MHz, $CDCl_3$): δ 4.35–4.33 (br, 1H), 1.80–1.70 (m, 4H), 1.60–1.50 (m, 4H), 1.34 (br, 1H, OH); $^{13}C\{^1H\}$ NMR (101 MHz, $CDCl_3$): δ 73.7, 35.3, 23.1. Spectroscopic data consistent with literature.[6]

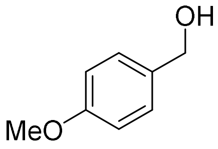

5r

(4-Methoxyphenyl)methanol (5r).[7] A mixture of **4r** (0.14 g, 1.00 mmol), **2a** (3.10 mg, 0.005 mmol), KO^tBu (1.12 mg, 0.01 mmol), and THF (3 mL) were used. **5r** (0.14 g, 99%) was obtained by column chromatography (SiO_2; *n*-hexane:EtOAc; 95:5) as yellow liquid. ^1H NMR (400 MHz, $CDCl_3$): δ 7.30 (d, J = 8.6 Hz, 2H), 6.90 (d, J = 8.6 Hz, 2H), 4.62 (s, 2H), 3.81 (s, 3H); $^{13}C\{^1H\}$ NMR (101 MHz, $CDCl_3$): δ 158.8, 133.0, 128.4, 113.6, 64.4, 55.1. Spectroscopic data consistent with literature.[7]

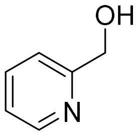

5s

Pyridin-2-ylmethanol (5s).[11] A mixture of **4s** (0.11 g, 1.00 mmol), **2a** (3.10 mg, 0.005 mmol), KO^tBu (1.12 mg, 0.01 mmol), and THF (3 mL) were used. **5s** (0.082 g, 75%) was obtained by column chromatography (SiO_2; *n*-hexane:EtOAc; 95:5) as yellow liquid. ^1H NMR (400 MHz, $CDCl_3$): δ 8.56 (d, J = 7.6 Hz, 1H), 7.69 (td, J = 7.6, 1.6 Hz, 1H), 7.25 (d, J = 7.6 Hz, 1H), 7.20 (t, J = 7.6, 1H), 4.77 (s, 2H); $^{13}C\{^1H\}$ NMR (101 MHz, $CDCl_3$): δ 159.7, 148.2, 136.8, 122.1, 120.7, 64.1. Spectroscopic data consistent with literature.[11]

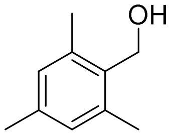

5t

Mesitylmethanol (5t).[12] A mixture of **4t** (0.15 g, 1.00 mmol), **2a** (3.10 mg, 0.005 mmol), KO^tBu (1.12 mg, 0.01 mmol), and THF (3 mL) were used. **5t** (0.13 g, 88%) was obtained by column chromatography (SiO_2; *n*-hexane:EtOAc; 95:5) as white solid. ^1H NMR (400 MHz, $CDCl_3$): δ 6.87 (s, 2H), 4.71 (s, 2H), 2.39 (s, 6H), 2.27 (s, 3H); $^{13}C\{^1H\}$ NMR (101 MHz, $CDCl_3$): δ 137.6, 137.3, 133.7, 129.1, 59.0, 20.9, 19.3. Spectroscopic data consistent with literature.[12]

1.2.3. Application in the Ullmann-type reaction

1.2.3.1. Table S2

Table S2. Screening optimization of reaction conditions using a Taguchi L9 array. [a]

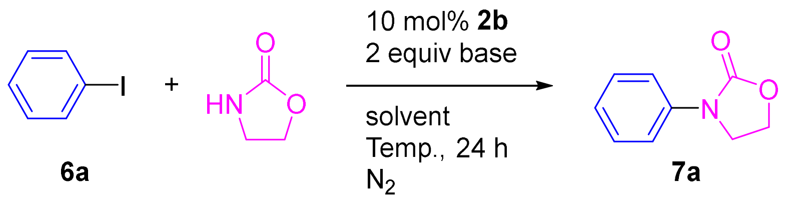

Entry	Base	Solvent	Temp. (°C)	Time (h)	NMR ratio[b] **6a**:**7a**
1	K_3PO_4	DMSO	120	24	4:96 (80)
2	K_2CO_3	Toluene	120	24	44:56
3	KO*t*Bu	Dioxane	120	24	> 99:1
4	K_3PO_4	Toluene	90	24	90:10
5	K_2CO_3	Dioxane	90	24	85:15
6	KO*t*Bu	DMSO	90	24	95:5
7	K_3PO_4	Dioxane	70	24	> 99:1
8	K_2CO_3	DMSO	70	24	60:40
9	KO*t*Bu	Toluene	70	24	> 99:1

[a] The reaction was carried out on a 1 mmol scale of **6a**. [b] The ratio was determined by 400 MHz NMR. Isolated yield of **7a** was shown in parentheses.

1.2.3.1. General procedure for Table 4 and Table 5

All manipulations were carried out under N_2. To a Schleck tube was charged with aryl iodide (1.0 mmol, 1.0 equiv), amide or carbamate (1.3 mmol, 1.3 equiv), **2a** (0.08 mmol, 8 mol%), K_2CO_3 (2.0 mmol, 2.0 equiv), and DMSO (3 mL). After stirring at 120ºC on an oil bath for 24 h, the reaction mixture was quenched with H_2O (10 mL). The aqueous layer was extracted with EtOAc (10 mL × 3). The combined organic layers were washed with water (10 mL × 3) and brine (10 mL). The combined organic layers were dried over anhydrous Na_2SO_4, and then filtered through celite. The solvent was evaporated under reduced pressure and the corresponding crude product was purified by column chromatography on a 200–400 mesh silica gel using 50% ethyl acetate in *n*-hexane as the eluent to get the final product **7**.

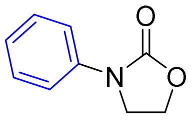

7a

3-Phenyloxazolidin-2-one (7a).[13] A mixture of **6a** (0.20 g, 1.00 mmol), oxazolidine-2-one (0.11 g 1.30 mmol), **2a** (0.05 g, 0.08 mmol), K_2CO_3 (0.28 g, 2.00 mmol), and DMSO (3 mL) were used. **7a** (0.13 g, 82%) was obtained by column chromatography (SiO_2; *n*-hexane:EtOAc; 1:1) as brown solid. ^{1}H NMR (400 MHz, $CDCl_3$): δ 7.55 (d, J = 7.6 Hz, 2H), 7.39 (t, J = 7.6 Hz, 2H), 7.16 (t, J = 7.6 Hz, 1H), 4.50 (t, J = 7.6 Hz, 2H), 4.07 (t, J = 7.6 Hz, 2H); $^{13}C\{^1H\}$ NMR (101 MHz, $CDCl_3$): δ 155.0, 137.9, 128.6, 123.6, 117.8, 61.0, 44.7. Spectroscopic data consistent with literature.[13]

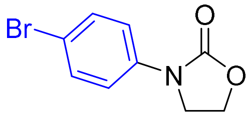

7b

3-(4-Bromophenyl)oxazolidin-2-one (7b).[13] A mixture of **6b** (0.28 g, 1.00 mmol), oxazolidine-2-one (0.11 g 1.30 mmol), **2a** (0.05 g, 0.08 mmol), K_2CO_3 (0.28 g, 2.00 mmol), and DMSO (3 mL) were used. **7b** (0.23 g, 93%) was obtained by column chromatography (SiO_2; *n*-hexane:EtOAc; 3:1) as brown solid. ^{1}H NMR (400 MHz, $CDCl_3$): δ 7.51–7.44 (m, 4H), 4.50 (t, J = 8.0 Hz, 2H), 4.05 (t, J = 8.0 Hz, 2H); $^{13}C\{^1H\}$ NMR (101 MHz, $CDCl_3$): δ 154.9, 137.2, 131.8, 119.5, 116.6, 61.2, 44.8. Spectroscopic data consistent with literature.[13]

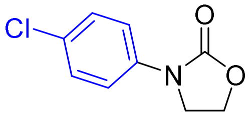

7c

3-(4-Chlorophenyl)oxazolidin-2-one (7c).[13] A mixture of **6c** (0.24 g, 1.00 mmol), oxazolidine-2-one (0.11 g 1.30 mmol), **2a** (0.05 g, 0.08 mmol), K_2CO_3 (0.28 g, 2.00 mmol), and DMSO (3 mL) were used. **7c** (0.19 g, 94%) was obtained by column chromatography (SiO_2; *n*-hexane:EtOAc; 1:1) as brown solid. ^{1}H NMR (400 MHz, $CDCl_3$): δ 7.50 (d, J = 9.2 Hz, 2H), 7.34 (d, J = 9.2 Hz, 2H), 4.50 (t, J = 7.6 Hz, 2H), 4.05 (t, J = 7.6 Hz, 2H); $^{13}C\{^1H\}$ NMR (101 MHz, $CDCl_3$): δ 154.9, 136.6, 128.7, 119.0, 61.1, 44.7. Spectroscopic data consistent with literature.[13]

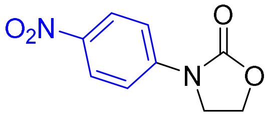

7d

3-(4-Nitrophenyl)oxazolidin-2-one (7d).[13] A mixture of **6d** (0.25 g, 1.00 mmol), oxazolidine-2-one (0.11 g 1.30 mmol), **2a** (0.05 g, 0.08 mmol), K_2CO_3 (0.28 g, 2.00 mmol), and DMSO (3 mL) were used. **7d** (0.15 g, 71%) was obtained by column chromatography (SiO_2; *n*-hexane:EtOAc; 1:2) as yellow solid. ^{1}H NMR (400 MHz, $CDCl_3$): δ 8.28 (d, J = 9.2 Hz, 2H), 7.74 (d, J = 9.2 Hz, 2H), 4.57 (t, J = 8.8 Hz, 2H), 4.15 (t, J = 8.8 Hz, 2H); $^{13}C\{^1H\}$ NMR (101 MHz, $CDCl_3$): δ 154.5, 143.7, 143.3,

124.9, 117.4, 61.1, 44.7. Spectroscopic data consistent with literature.[13]

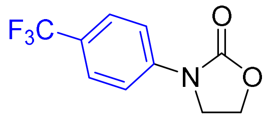

7e

3-(4-(Trifluoromethyl)phenyl)oxazolidin-2-one (7e).[14] A mixture of **6e** (0.27 g, 1.00 mmol), oxazolidine-2-one (0.11 g 1.30 mmol), **2a** (0.05 g, 0.08 mmol), K_2CO_3 (0.28 g, 2.00 mmol), and DMSO (3 mL) were used. **7e** (0.19 g, 84%) was obtained by column chromatography (SiO_2; *n*-hexane:EtOAc; 3:1) as white solid. ^{1}H NMR (400 MHz, $CDCl_3$): δ 7.68 (d, *J* = 8.8 Hz, 2H), 7.64 (d, *J* = 8.8 Hz, 2H), 4.54 (t, *J* = 8.0 Hz, 2H), 4.10 (t, *J* = 8.0 Hz, 2H); ^{13}C{^{1}H} NMR (101 MHz, $CDCl_3$): δ 154.9, 141.2, 126.1 (q, $J_{C–F}$ = 3.4 Hz), 125.3 (q, $J_{C–F}$ = 33.0 Hz), 121.2 (d, $J_{C–F}$ = 270.2 Hz), 117.5, 61.3, 44.8; ^{19}F NMR (376 MHz, $CDCl_3$): δ -63.1. Spectroscopic data consistent with literature.[14]

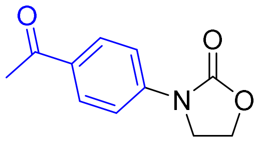

7f

3-(4-Acetylphenyl)oxazolidin-2-one (7f).[13] A mixture of **6f** (0.25 g, 1.00 mmol), oxazolidine-2-one (0.11 g 1.30 mmol), **2a** (0.05 g, 0.08 mmol), K_2CO_3 (0.28 g, 2.00 mmol), and DMSO (3 mL) were used. **7f** (0.19 g, 91%) was obtained by column chromatography (SiO_2; *n*-hexane:EtOAc; 1:1) as white solid. ^{1}H NMR (400 MHz, $CDCl_3$): δ 8.00 (d, *J* = 8.8 Hz, 2H), 7.66 (d, *J* = 8.8 Hz, 2H), 4.54 (t, *J* = 8.0 Hz, 2H), 4.12 (t, *J* = 8.0 Hz, 2H), 2.60 (s, 3H); ^{13}C{^{1}H} NMR (101 MHz, $CDCl_3$): δ 196.8, 154.7, 142.2, 132.2, 129.4, 117.0, 61.3, 44.7, 26.3. Spectroscopic data consistent with literature.[13]

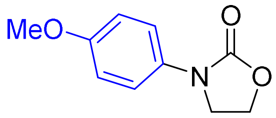

7g

3-(4-Methoxyphenyl)oxazolidin-2-one (7g).[13] A mixture of **6g** (0.23 g, 1.00 mmol), oxazolidine-2-one (0.11 g 1.30 mmol), **2a** (0.05 g, 0.08 mmol), K_2CO_3 (0.28 g, 2.00 mmol), and DMSO (3 mL) were used. **7g** (0.18 g, 91%) was obtained by column chromatography (SiO_2; *n*-hexane:EtOAc; 1:1) as brown solid. ^{1}H NMR (400 MHz, $CDCl_3$): δ 7.44 (d, *J* = 9.2 Hz, 2H), 6.94–6.90 (d, *J* = 9.2 Hz, 2H), 4.47 (t, *J* = 8.8 Hz, 2H), 4.03 (t, *J* = 8.8 Hz, 2H), 3.81 (s, 3H); ^{13}C{^{1}H} NMR (101 MHz, $CDCl_3$): δ 155.9, 155.3, 131.2, 119.9, 113.9, 61.1, 55.2, 45.3. Spectroscopic data consistent with literature.[13]

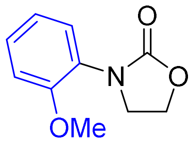

7h

3-(2-Methoxyphenyl)oxazolidin-2-one (7h).[15] A mixture of **6h** (0.23 g, 1.00 mmol), oxazolidine-2-one (0.11 g 1.30 mmol), **2a** (0.05 g, 0.08 mmol), K_2CO_3 (0.28 g, 2.00 mmol), and DMSO (3 mL) were used. **7h** (0.15 g, 80%) was obtained by column chromatography (SiO_2; *n*-hexane:EtOAc; 1:1) as brown liquid. ^{1}H NMR (400 MHz, $CDCl_3$): δ 7.37 (dd, *J* = 8.0, 1.6 Hz, 1H), 7.29 (td, *J* = 8.0, 1.6 Hz, 1H), 7.01–6.95 (m, 2H), 4.49 (t, *J* = 8.8 Hz, 2H), 3.99 (t, *J* = 8.8 Hz, 2H), 3.87 (s, 3H); ^{13}C{^{1}H} NMR (101 MHz, $CDCl_3$): δ 157.2, 154.6, 128.6, 128.1, 125.7, 120.6, 111.7, 62.2, 55.3, 46.7. Spectroscopic data consistent with literature.[15]

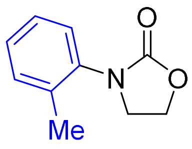

7i

3-(o-Tolyl)oxazolidin-2-one (7i).[15] A mixture of **6i** (0.22 g, 1.00 mmol), oxazolidine-2-one (0.11 g 1.30 mmol), **2a** (0.05 g, 0.08 mmol), K_2CO_3 (0.28 g, 2.00 mmol), and DMSO (3 mL) were used. **7i** (0.16 g, 88%) was obtained by column chromatography (SiO_2; *n*-hexane:EtOAc; 1:1) as yellow liquid. ^{1}H NMR (400 MHz, $CDCl_3$): δ 7.29–7.26 (m, 2H), 7.24 (dd, *J* = 4.8, 1.2 Hz, 2H), 4.53 (t, *J* = 8.0 Hz, 2H), 3.96 (t, *J* = 8.0

Hz, 2H), 2.32 (s, 3H); $^{13}C\{^1H\}$ NMR (101 MHz, $CDCl_3$): δ 156.6, 135.8, 135.6, 131.1, 127.9, 126.7, 126.2, 62.2, 47.6, 17.6. Spectroscopic data consistent with literature.[15]

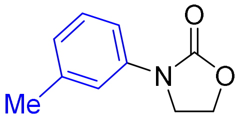

7j

3-(m-Tolyl)oxazolidin-2-one (7j).[14] A mixture of **6j** (0.22 g, 1.00 mmol), oxazolidine-2-one (0.11 g 1.30 mmol), **2a** (0.05 g, 0.08 mmol), K_2CO_3 (0.28 g, 2.00 mmol), and DMSO (3 mL) were used. **7j** (0.16 g, 90%) was obtained by column chromatography (SiO_2; *n*-hexane:EtOAc; 1:1) as brown solid. ^1H NMR (400 MHz, $CDCl_3$): δ 7.40 (s, 1H), 7.32–7.26 (m, 2H), 6.97 (d, J = 7.2 Hz, 1H), 4.48 (t, J = 7.2 Hz, 2H), 4.06 (t, J = 7.2 Hz, 2H), 2.38 (s, 3H); $^{13}C\{^1H\}$ NMR (101 MHz, $CDCl_3$): δ 155.1, 138.7, 138.0, 128.6, 124.6, 118.7, 115.1, 61.1, 45.0, 21.4. Spectroscopic data consistent with literature.[14]

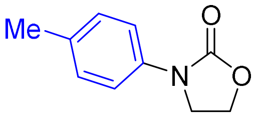

7k

3-(p-Tolyl)oxazolidin-2-one (7k).[13] A mixture of **6k** (0.22 g, 1.00 mmol), oxazolidine-2-one (0.11 g 1.30 mmol), **2a** (0.05 g, 0.08 mmol), K_2CO_3 (0.28 g, 2.00 mmol), and DMSO (3 mL) were used. **7k** (0.13 g, 71%) was obtained by column chromatography (SiO_2; *n*-hexane:EtOAc; 1:1) as white solid. ^1H NMR (400 MHz, $CDCl_3$): δ 7.43–7.40 (d, J = 8.4 Hz, 2H), 7.18 (d, J = 8.4 Hz, 2H), 4.48 (t, J = 8.4 Hz, 2H), 4.05 (t, J = 8.4 Hz, 2H), 2.33 (s, 3H); $^{13}C\{^1H\}$ NMR (101 MHz, $CDCl_3$): δ 155.2, 135.5, 133.4, 129.3, 118.0, 61.1, 45.0, 20.5. Spectroscopic data consistent with literature.[13]

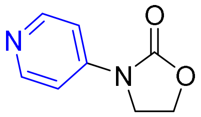

7l

3-(Pyridin-4-yl)oxazolidin-2-one (7l).[14] A mixture of **6l** (0.20 g, 1.00 mmol), oxazolidine-2-one (0.11 g 1.30 mmol), **2a** (0.05 g, 0.08 mmol), K_2CO_3 (0.28 g, 2.00 mmol), and DMSO (3 mL) were used. **7l** (0.06 g, 35%) was obtained by column chromatography (SiO_2; *n*-hexane:EtOAc; 1:2) as white solid. ^1H NMR (400 MHz, $CDCl_3$): δ 8.56 (d, J = 5.0 Hz, 2H), 7.50 (dd, J = 5.0, 1.6 Hz, 2H), 4.55 (t, J = 8.0 Hz, 2H), 4.07 (t, J = 8.0 Hz, 2H); $^{13}C\{^1H\}$ NMR (101 MHz, $CDCl_3$): δ 154.4, 150.4, 144.8, 111.6, 61.4, 43.8. Spectroscopic data consistent with literature.[14]

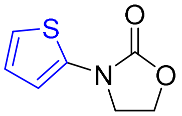

7m

3-(Thiophen-2-yl)oxazolidin-2-one (7m).[14] A mixture of **6m** (0.21 g, 1.00 mmol), oxazolidine-2-one (0.11 g 1.30 mmol), **2a** (0.05 g, 0.08 mmol), K_2CO_3 (0.28 g, 2.00 mmol), and DMSO (3 mL) were used. **7m** (0.10 g, 60%) was obtained by column chromatography (SiO_2; *n*-hexane:EtOAc; 1:1) as brown solid. ^1H NMR (400 MHz, $CDCl_3$): δ 6.94 (dd, J = 5.6, 1.2 Hz, 1H), 6.88 (t, J = 5.6 Hz, 1H), 6.51 (dd, J = 5.6, 1.2 Hz, 1H), 4.56 (t, J = 8.0 Hz, 2H), 4.07 (t, J = 8.0 Hz, 2H); $^{13}C\{^1H\}$ NMR (101 MHz, $CDCl_3$): δ 154.5, 140.1, 124.5, 117.8, 110.7, 62.1, 45.8. Spectroscopic data consistent with literature.[14]

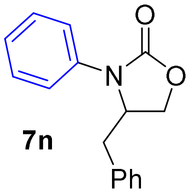

7n

4-Benzyl-3-phenyloxazolidin-2-one (7n).[14] A mixture of **6n** (0.20 g, 1.00 mmol), 4-benzyloxazolidine-2-one (0.23 g 1.30 mmol), **2a** (0.05 g, 0.08 mmol), K_2CO_3 (0.28 g, 2.00 mmol), and DMSO (3 mL) were used. **7n** (0.18 g, 70%) was obtained by column chromatography (SiO_2; *n*-hexane:EtOAc; 1:1) as white solid. ^1H NMR (400 MHz, $CDCl_3$): δ 7.56 (d, J = 7.6 Hz, 2H), 7.45 (t, J = 7.6 Hz, 2H), 7.34–7.21 (m, 4H), 7.13 (d, J = 7.6 Hz, 2H), 4.70–4.63 (m, 1H), 4.36 (t, J = 13.8 Hz, 1H), 4.21 (dd, J = 13.8, 3.4 Hz, 1H), 3.15

(dd, J = 13.8, 3.4 Hz, 1H), 2.78 (dd, J = 13.8, 3.4 Hz, 1H); $^{13}C\{^1H\}$ NMR (101 MHz, $CDCl_3$): δ 155.4, 136.5, 135.0, 129.1, 129.0, 128.7, 127.0, 125.0, 121.4, 65.8, 56.9, 37.4. Spectroscopic data consistent with literature.[14]

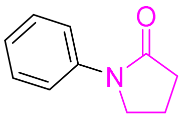

9a

1-Phenylpyrrolidin-2-one (9a).[16] A mixture of **6a** (0.20 g, 1.00 mmol), **8a** (0.11 g 1.30 mmol), **2a** (0.05 g, 0.08 mmol), K_2CO_3 (0.28 g, 2.00 mmol), and DMSO (3 mL) were used. **9a** (0.15 g, 91%) was obtained by column chromatography (SiO_2; *n*-hexane:EtOAc; 1:1) as brown solid. ^1H NMR (400 MHz, $CDCl_3$): δ 7.61 (dd, J = 8.8, 1.2 Hz, 2H), 7.37 (t, J = 8.8 Hz, 2H), 7.14 (t, J = 8.8 Hz, 1H), 3.88 (t, J = 8.0 Hz, 2H), 2.62 (t, J = 8.0 Hz, 2H), 2.17 (septet, J = 8.0 Hz, 2H); $^{13}C\{^1H\}$ NMR (101 MHz, $CDCl_3$): δ 174.0, 139.2, 128.6, 124.2, 119.7, 48.5, 32.6, 17.8. Spectroscopic data consistent with literature.[16]

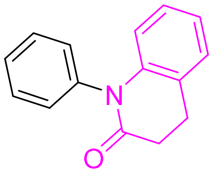

9b

1-Phenyl-3,4-dihydroquinolin-2(1H)-one (9b).[17] A mixture of **6a** (0.20 g, 1.00 mmol), **8b** (0.19 g 1.30 mmol), **2a** (0.05 g, 0.08 mmol), K_2CO_3 (0.28 g, 2.00 mmol), and DMSO (3 mL) were used. **9b** (0.16 g, 70%) was obtained by column chromatography (SiO_2; *n*-hexane:EtOAc; 1:1) as red solid. ^1H NMR (400 MHz, $CDCl_3$): δ 7.53 (t, J = 8.0 Hz, 2H), 7.42 (t, J = 7.2 Hz, 1H), 7.25 (d, J = 7.2 Hz, 1H), 7.22–7.20 (m, 2H), 7.04 (t, J = 7.2 Hz, 1H), 6.99 (t, J = 7.2 Hz, 1H), 6.36 (d, J = 8.0 Hz, 1H), 3.08 (t, J = 8.0 Hz, 2H), 2.83 (t, J = 8.0 Hz, 2H); $^{13}C\{^1H\}$ NMR (101 MHz, $CDCl_3$): δ 170.0, 141.4, 138.2, 129.6, 128.8, 127.9, 127.6, 126.9, 125.4, 122.8, 116.8, 32.0, 25.4. Spectroscopic data consistent with literature.[17]

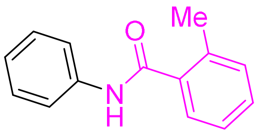

9c

2-Methyl-N-phenylbenzamide (9c).[18] A mixture of **6a** (0.20 g, 1.00 mmol), **8c** (0.18 g 1.30 mmol), **2a** (0.05 g, 0.08 mmol), K_2CO_3 (0.28 g, 2.00 mmol), and DMSO (3 mL) were used. **9c** (0.19 g, 90%) was obtained by column chromatography (SiO_2; *n*-hexane:EtOAc; 5:1) as yellow solid. ^1H NMR (400 MHz, $CDCl_3$): δ 7.63 (d, J = 7.2 Hz, 2H), 7.50 (d, J = 7.2 Hz, 1H), 7.44 (s, 1H), 7.38 (t, J = 7.2 Hz, 3H), 7.27 (t, J = 7.2 Hz, 2H), 7.16 (t, J = 7.2 Hz, 1H), 2.52 (s, 3H); $^{13}C\{^1H\}$ NMR (101 MHz, $CDCl_3$): δ 168.3, 138.0, 136.3, 136.1, 131.0, 130.0, 128.8, 126.6, 125.6, 124.3, 119.9, 19.6. Spectroscopic data consistent with literature.[18]

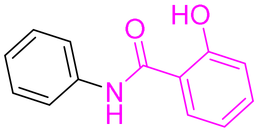

9d

2-Hydroxy-N-phenylbenzamide (9d).[19] A mixture of **6a** (0.20 g, 1.00 mmol), **8d** (0.20 g 1.30 mmol), **2a** (0.05 g, 0.08 mmol), K_2CO_3 (0.28 g, 2.00 mmol), and DMSO (3 mL) were used. **9d** (0.19 g, 90%) was obtained by column chromatography (SiO_2; *n*-hexane:EtOAc; 5:1) as yellow solid. ^1H NMR (400 MHz, $CDCl_3$): δ 11.99 (s, 1H, OH), 7.92 (s, 1H, NH), 7.59 (dd, J = 8.4, 1.6 Hz, 2H), 7.53 (dd, J = 8.2, 1.6 Hz, 1H), 7.46 (t, J = 8.4 Hz, 1H), 7.41 (t, J = 8.4 Hz, 2H), 7.21 (t, J = 8.2 Hz, 1H), 7.05 (dd, J = 8.2, 1.6 Hz, 1H), 6.94 (t, J = 8.2 Hz, 1H); $^{13}C\{^1H\}$ NMR (101 MHz, $CDCl_3$): δ 168.4, 161.6, 136.5, 134.6, 129.1, 125.6, 125.3, 121.3, 119.0, 118.7, 114.6. Spectroscopic data consistent with literature.[19]

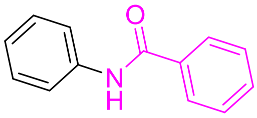

9e

N-Phenylbenzamide (9e).[18] A mixture of **6a** (0.20 g, 1.00 mmol), **8e** (0.16 g 1.30 mmol), **2a** (0.05 g, 0.08 mmol), K_2CO_3 (0.28 g, 2.00 mmol), and DMSO (3 mL) were used. **9e** (0.09 g, 41%) was obtained by column chromatography (SiO_2; *n*-hexane:EtOAc; 10:1) as red solid. ^{1}H NMR (400 MHz, $CDCl_3$): δ 7.88 (d, *J* = 8.0 Hz, 2H), 7.80 (s, 1H, N**H**), 7.65 (d, *J* = 8.0 Hz, 2H), 7.57 (t, *J* = 8.0 Hz, 1H), 7.51 (t, *J* = 8.0 Hz, 2H), 7.39 (t, *J* = 8.0 Hz, 2H), 7.17 (t, *J* = 8.0 Hz, 1H); ^{13}C{^{1}H} NMR (101 MHz, $CDCl_3$): δ 165.8, 137.9, 134.9, 131.8, 129.0, 128.7, 127.0, 124.5, 120.2. Spectroscopic data consistent with literature.[18]

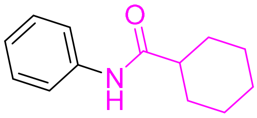

9f

N-Phenylcyclohexanecarboxamide (9f).[18] A mixture of **6a** (0.20 g, 1.00 mmol), **8f** (0.17 g 1.30 mmol), **2a** (0.05 g, 0.08 mmol), K_2CO_3 (0.28 g, 2.00 mmol), and DMSO (3 mL) were used. **9f** (0.04 g, 22%) was obtained by column chromatography (SiO_2; *n*-hexane:EtOAc; 70:30) as yellow solid. ^{1}H NMR (400 MHz, $CDCl_3$): δ 7.52 (d, *J* = 8.4 Hz, 2H), 7.31 (t, *J* = 8.4 Hz, 2H), 7.09 (t, *J* = 8.4 Hz, 1H), 2.22 (tt, *J* = 11.6, 3.2 Hz, 1H), 1.98–1.95 (m, 2H), 1.86–1.83 (m, 2H), 1.72–1.70 (m, 1H), 1.60–1.54 (m, 2H), 1.37–1.23 (m, 3H); ^{13}C{^{1}H} NMR (101 MHz, $CDCl_3$): δ 174.7, 138.0, 128.8, 123.9, 119.8, 46.3, 29.6, 25.6. Spectroscopic data consistent with literature.[18]

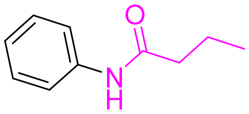

9g

N-Phenylbutyramide (9g).[18] A mixture of **6a** (0.20 g, 1.00 mmol), **8g** (0.11 g 1.30 mmol), **2a** (0.05 g, 0.08 mmol), K_2CO_3 (0.28 g, 2.00 mmol), and DMSO (3 mL) were used. **9g** (0.04 g, 25%) was obtained by column chromatography (SiO_2; *n*-hexane:EtOAc; 5:1) as purple solid. ^{1}H NMR (400 MHz, $CDCl_3$): δ 7.51 (d, *J* = 8.0 Hz, 2H), 7.32 (d, *J* = 8.0 Hz, 2H), 7.10 (t, *J* = 8.0 Hz, 1H), 2.34 (t, *J* = 7.2 Hz, 2H), 1.77 (sextet, *J* = 7.2 Hz, 2H), 1.02 (t, *J* = 7.2 Hz, 3H); ^{13}C{^{1}H} NMR (101 MHz, $CDCl_3$): δ 171.7, 138.0, 128.8, 124.1, 119.9, 39.5, 19.0, 13.7. Spectroscopic data consistent with literature.[18]

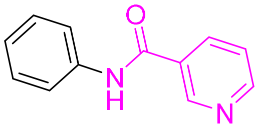

9h

N-Phenylnicotinamide (9h).[19] A mixture of **6a** (0.20 g, 1.00 mmol), **8h** (0.16 g 1.30 mmol), **2a** (0.05 g, 0.08 mmol), K_2CO_3 (0.28 g, 2.00 mmol), and DMSO (3 mL) were used. **9h** (0.18 g, 91%) was obtained by column chromatography (SiO_2; *n*-hexane:EtOAc; 1:1) as green solid. ^{1}H NMR (400 MHz, $CDCl_3$): δ 9.07 (s, 1H), 8.77 (d, 1H, *J* = 4.0 Hz), 8.21 (dt, *J* = 4.0, 1.6 Hz, 1H), 7.79 (s, 1H, NH), 7.62 (d, *J* = 8.0 Hz, 2H), 7.45 (dd, *J* = 4.0, 1.6 Hz, 1H), 7.39 (t, *J* = 8.0 Hz, 2H), 7.19 (t, *J* = 8.0 Hz, 1H); ^{13}C{^{1}H} NMR (101 MHz, $CDCl_3$): δ 164.3, 151.8, 148.0, 137.6, 135.4, 130.8, 128.8, 124.8, 123.4, 120.8. Spectroscopic data consistent with literature.[19]

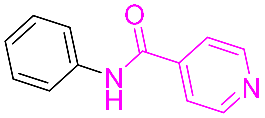

9i

N-Phenylisonicotinamide *(9i).*[20] A mixture of **6a** (0.20 g, 1.00 mmol), **8i** (0.16 g 1.30 mmol), **2a** (0.05 g, 0.08 mmol), K_2CO_3 (0.28 g, 2.00 mmol), and DMSO (3 mL) were used. **9i** (0.18 g, 92%) was obtained by column chromatography (SiO_2; *n*-hexane:EtOAc; 1:1) as yellow solid. ^{1}H NMR (400 MHz, $CDCl_3$): δ 8.84 (br, 2H), 7.81 (s, 1H, NH), 7.73 (d, *J* = 4.4 Hz, 2H), 7.65 (d, *J* = 8.0 Hz, 2H), 7.41 (t, *J* = 8.0 Hz, 2H), 7.21 (t, *J* = 8.0 Hz, 1H); ^{13}C{^{1}H} NMR (101 MHz, $CDCl_3$): δ 164.0, 150.5, 137.2, 134.3, 129.2, 127.5, 125.2, 120.5. Spectroscopic data consistent with literature.[20]

2. References and notes

1. Hsu, T.-Y.; Hsu, Y.-M.; Lai, Y.-T.; Lin, H.-P.; Wang, Y.-Y.; Lu, T.-J.; Lee, D.-S. *Asian J. Org. Chem.* **2022**, *11*, e202200397.
2. Lai, Y.-T.; Lin, H.-P.; Hsu, Y.-M.; Wang, Y.-Y.; Lee, D.-S.; Lu, T.-J. *Asian J. Org. Chem.* **2023**, *12*, e202200676.
3. Hsu, Y.-M.; Hua, Y.-W.; Wang, Y.-Y.; Lu, T.-J.; Lee. D.-S. *J. Chinese. Chem. Soc.* **2024**, *71*, 694.
4. Yang, T.-K.; Lee, D.-S. *Tetrahedron: Asymmetry* **1999**, *10*, 405.
5. Trose, M.; Lazreg, F. ; Chang, T.; Nahra, F.; Cordes, D. B.; Slawin, A. M. Z.; Cazin, C. S. J. *ACS Catal.* **2017**, *7*, 238.
6. Li, Z. W.; An, D. L.; Wei, Z. B.; Li, Y. Y.; Gao, J. X. *Tetrahedron Lett.* **2022**, *97*, 153798.
7. Sharma, A.; So, S.; Kim, J.-H.; MacMillan, S. N.; Baik, M.-H.; Trovitch, R. J. *Chem. Commun.* **2022**, *58*, 10793.
8. Kawamorita, S.; Hamasaka, G.; Ohmiya, H.; Hara, K.; Fukuoka, A.; Sawamura, M. *Org. Lett.* **2008**, *10*, 4697.
9. Fianu, G. D.; Schipper, K. C.; .Flowers II, R. A. *Catal. Sci. Tech.* **2017**, *7*, 3469.
10. McGeary, R. P. *Tetrahedron Lett.* **1998**, *39*, 3319.
11. Prasanth, C. P.; Ebbin, J.; Abhijith, A.; Nair, D. S.; Ibrahim, I.; Jevgenij R.; Bakthan S. *J. Org. Chem.* **2018**, *83*, 1431.
12. Gülcemal, S.; Gökçe, A. G.; Çetinkaya, B. Dalton Trans. 2013, 42, 7305.
13. Gupta, R.; Yadav, M.; Gaur, R.; Arora, G.; Sharma, R. K. *Green Chem.* **2017**, *19*, 3801.
14. Mahy, W.; Plucinski, P. K.; Frost, C. G. *Org. Lett.* **2014**, *16*, 5020.
15. Phillips, D. P.; Zhu, X.-F.; Lau, T L.; He, X; Yang, K; Liu, H. *Tetrahedron Lett.* **2009**, *50*, 7293.
16. Cristau, H.-J.; Cellier, P. P.; Spindler, J.-F.; Taillefer, M. *Chem. Eur. J.* **2004**, *10*, 5607.
17. Wu, L.; Hao, Y.; Liu, Y.; Wang, Q. *Org. Biomol. Chem.* **2019**, *17*, 6762.
18. Zhang, L.; Wang, W.; Wang, A.; Cui, Y.; Yang, X.; Huang, Y.; Liu, X.; Liu, W.; Son, J.-Y.; Oji, H.; Zhang, T. *Green Chem.* **2013**, *15*, 2680.
19. Ma, F.; Xie, X.; Zhang, L.; Peng, Z.; Ding, L.; Fu, L.; Zhang, Z. *J. Org. Chem.* **2012**, *77*, 5279.
20. Nguyen, T. T.; Duong, V. D.; Pham, T. N. N.; Duong, Q. T.; Nguyen, T. B. *Org. Biomol. Chem.* **2022**, *20*, 8054.

3. ^{1}H and ^{13}C{^{1}H}-NMR Spectra of Compounds 2, 5, 7, and 8

^{1}H NMR ($CDCl_3$, 400 MHz) spectrum of compound **2a**

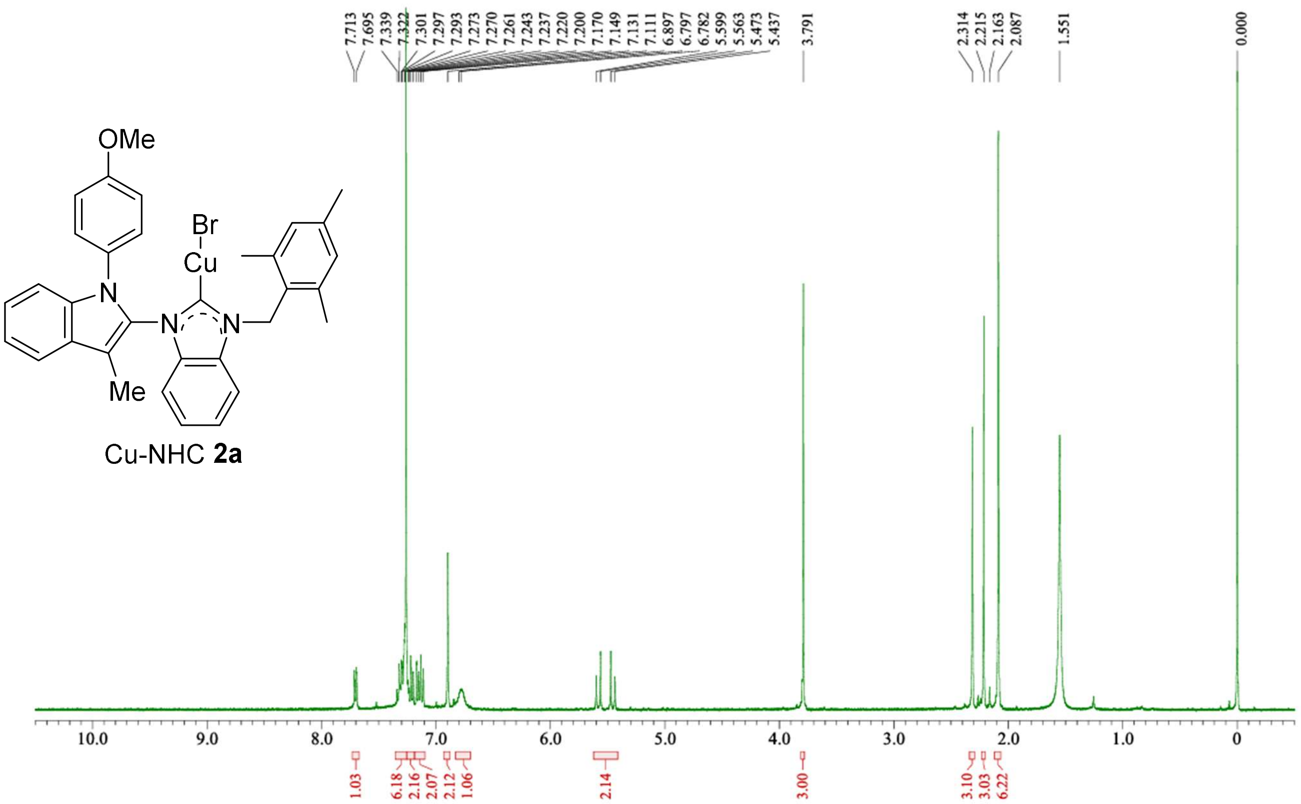

^{13}C{^{1}H} NMR ($CDCl_3$, 100 MHz) spectrum of compound **2a**

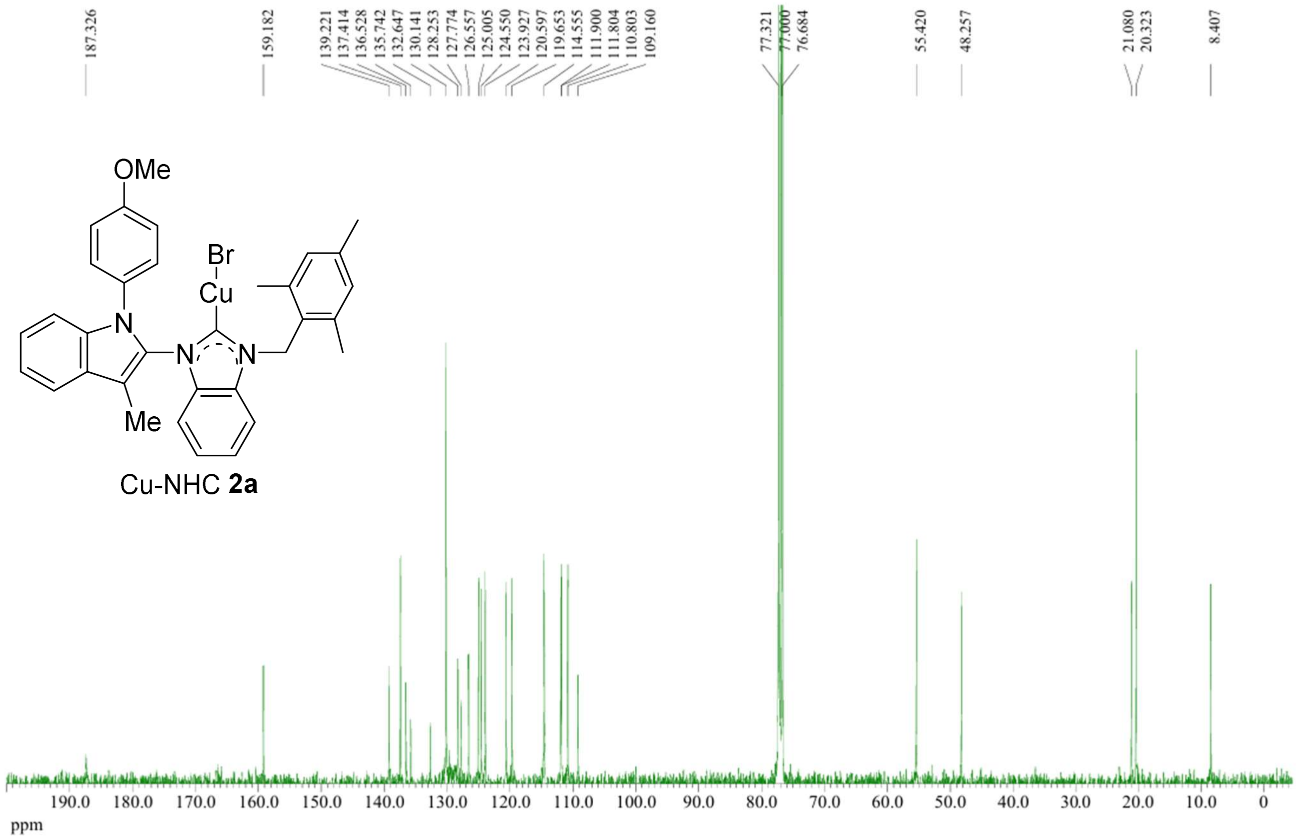

^{1}H NMR ($CDCl_3$, 400 MHz) spectrum of compound **2b**

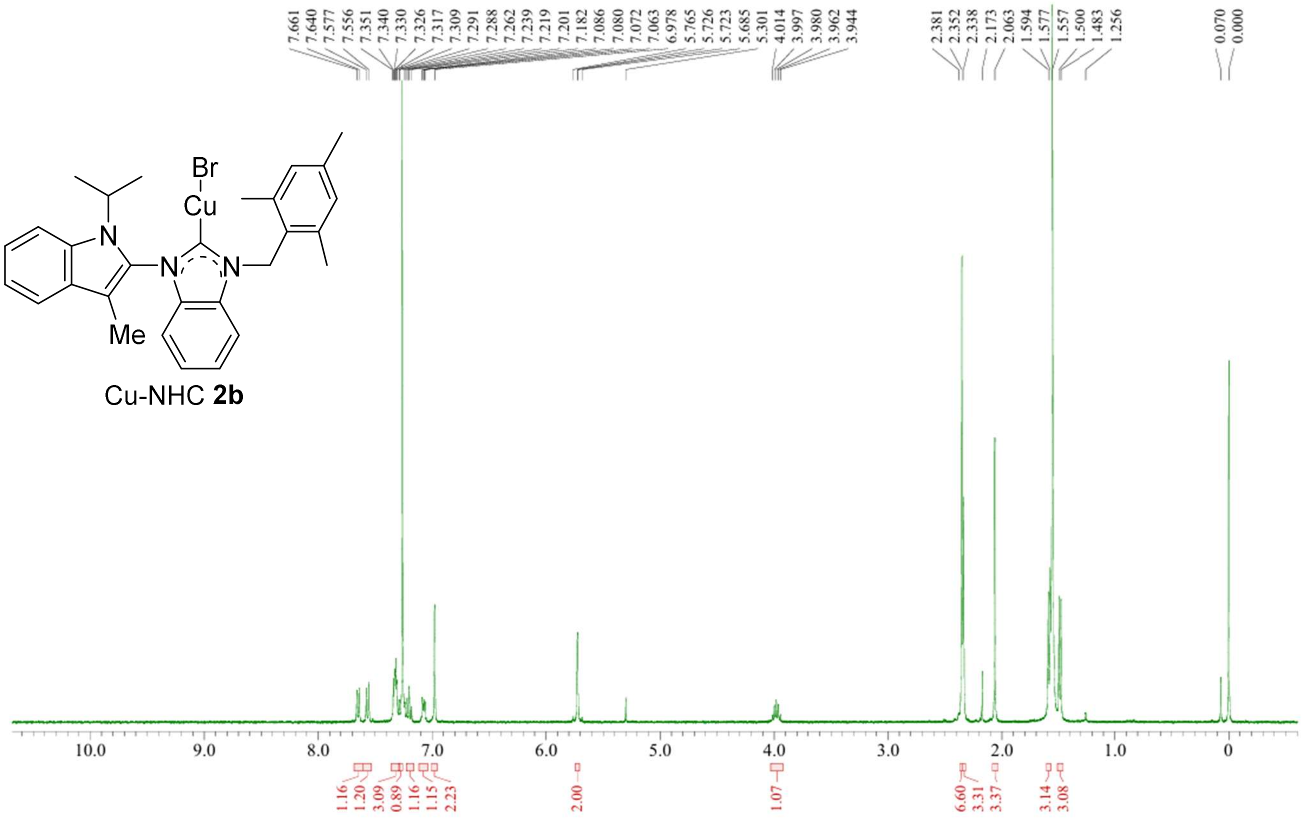

$^{13}C\{^{1}H\}$ NMR ($CDCl_3$, 100 MHz) spectrum of compound **2b**

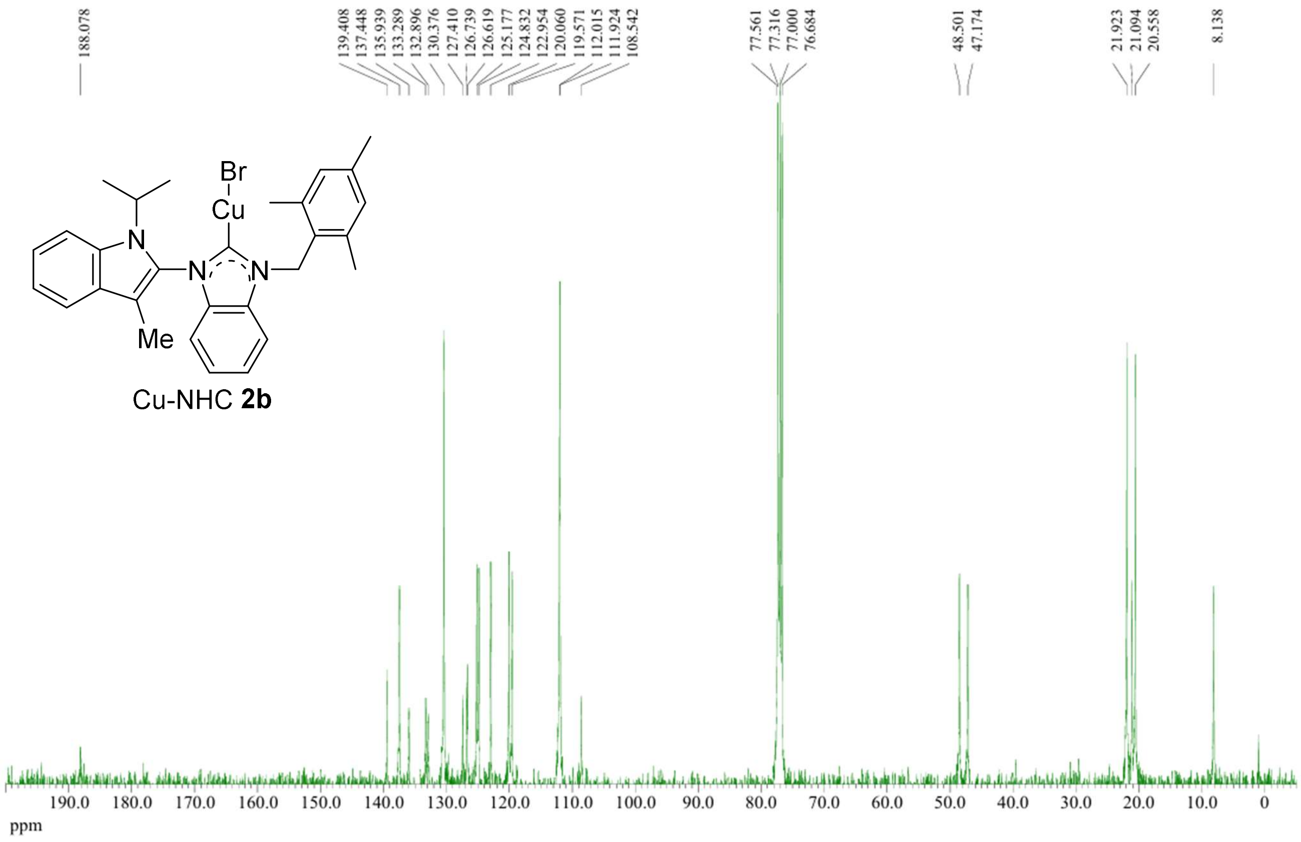

^{1}H NMR ($CDCl_3$, 400 MHz) spectrum of compound **2c**

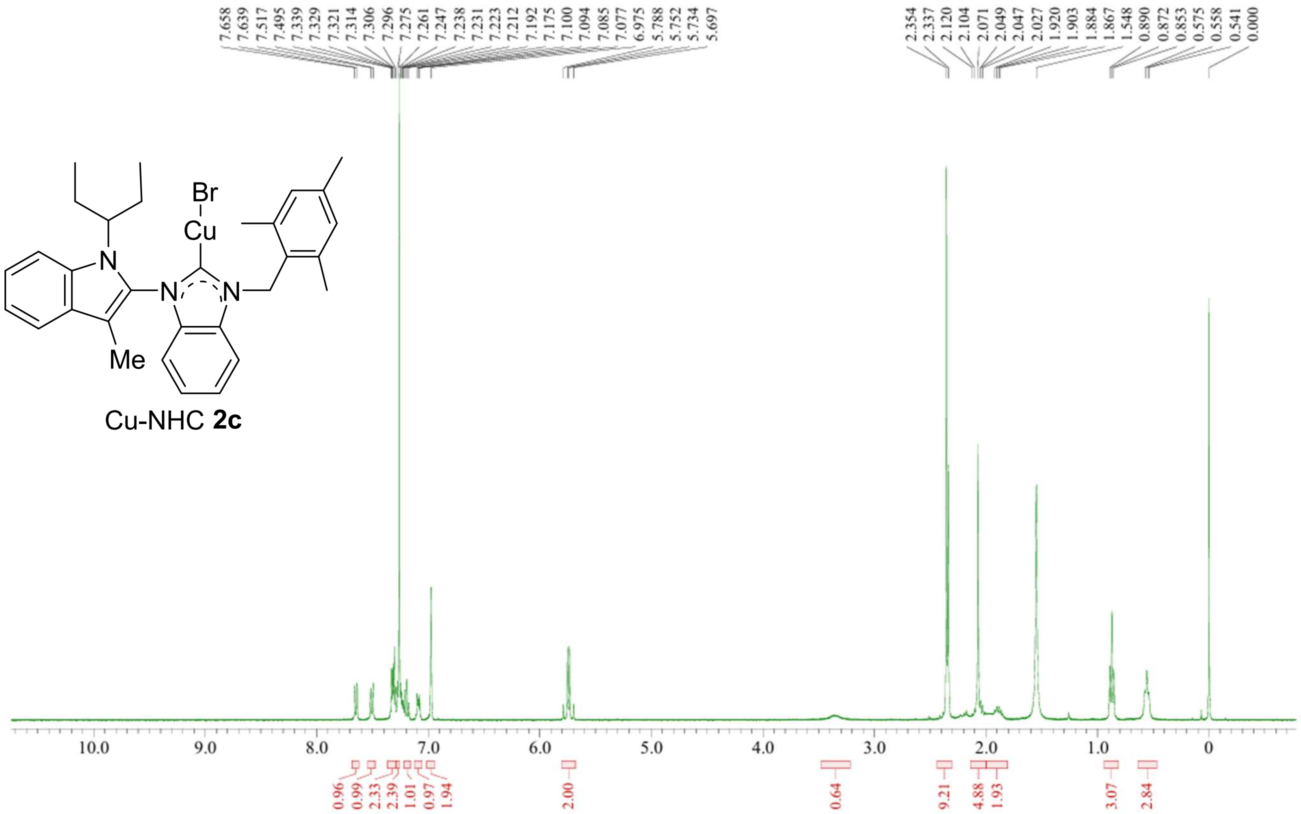

$^{13}C\{^{1}H\}$ NMR ($CDCl_3$, 100 MHz) spectrum of compound **2c**

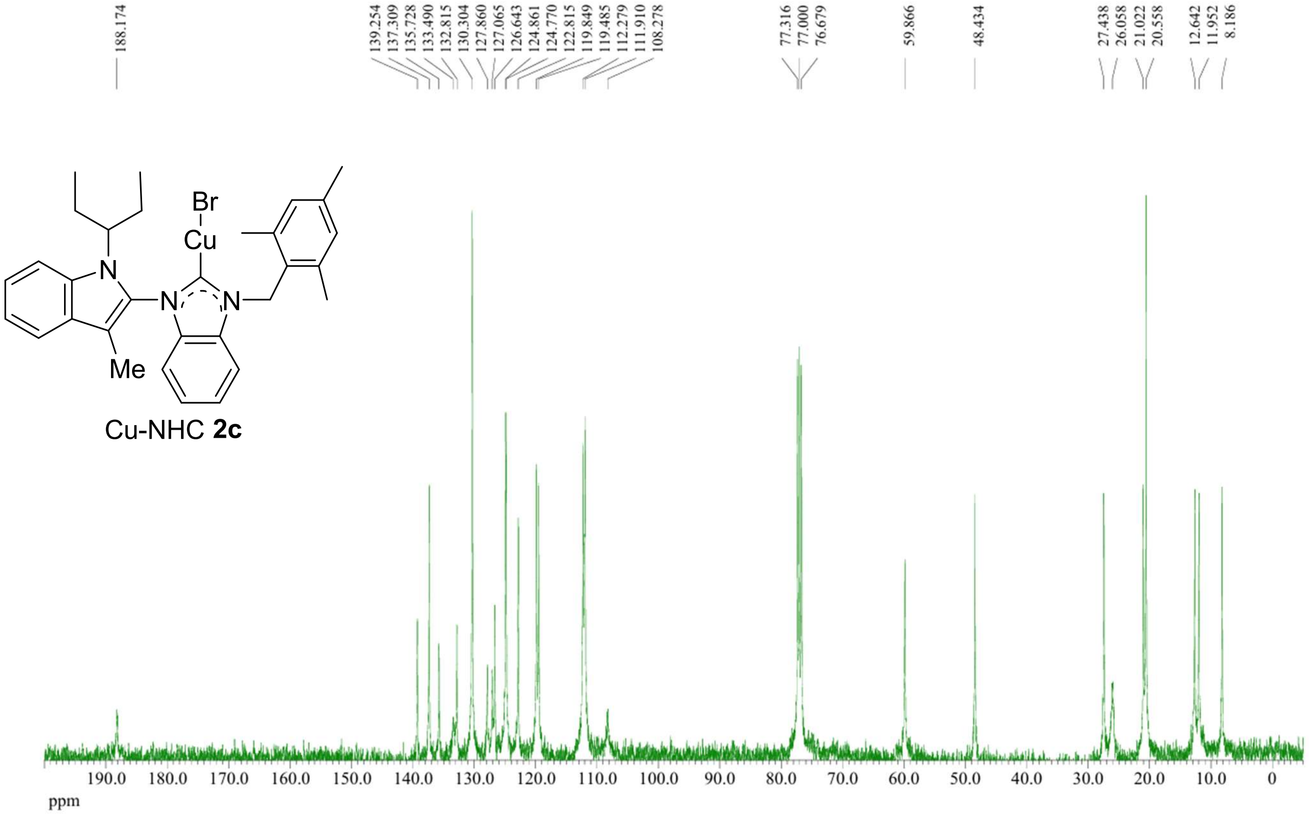

^{1}H NMR ($CDCl_3$, 400 MHz) spectrum of compound **2d**

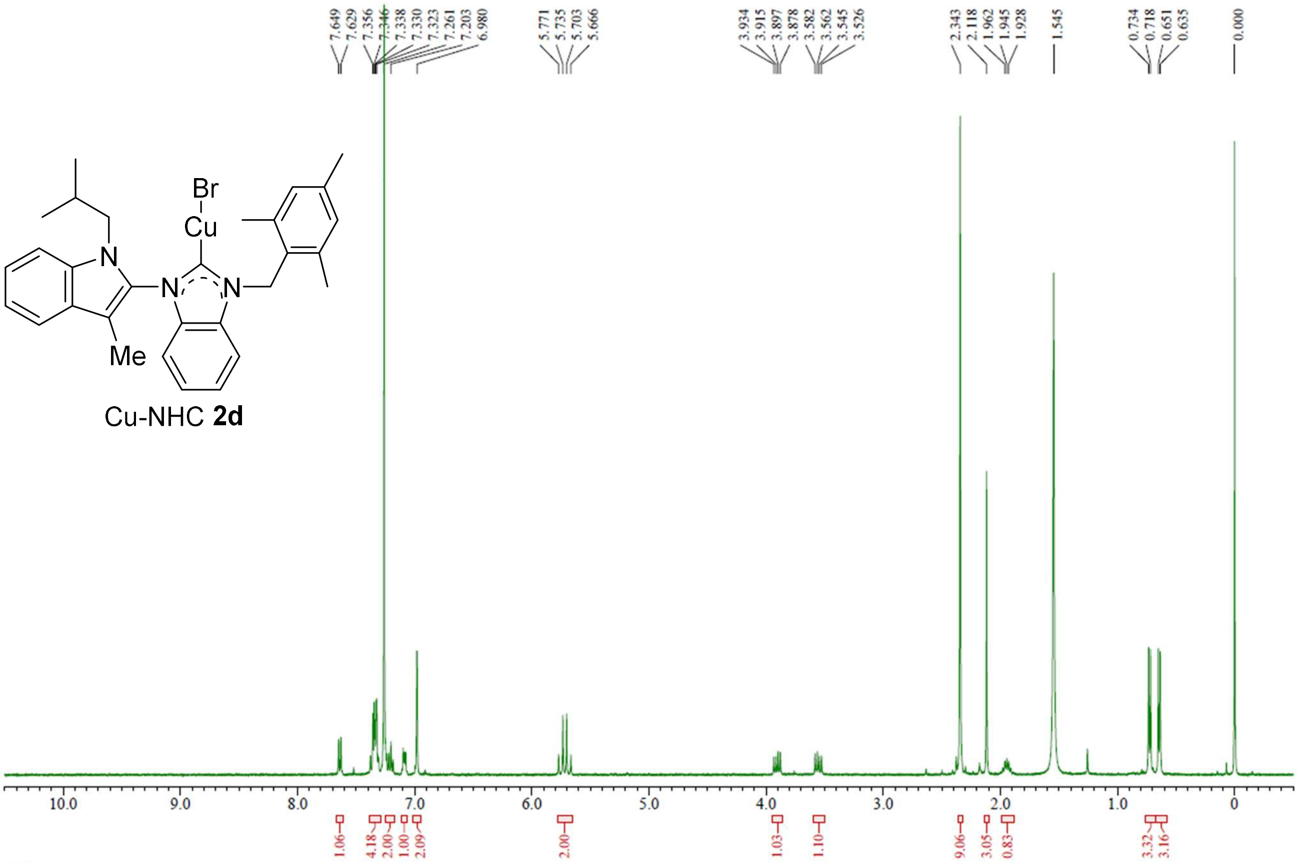

^{13}C{^{1}H} NMR ($CDCl_3$, 100 MHz) spectrum of compound **2d**

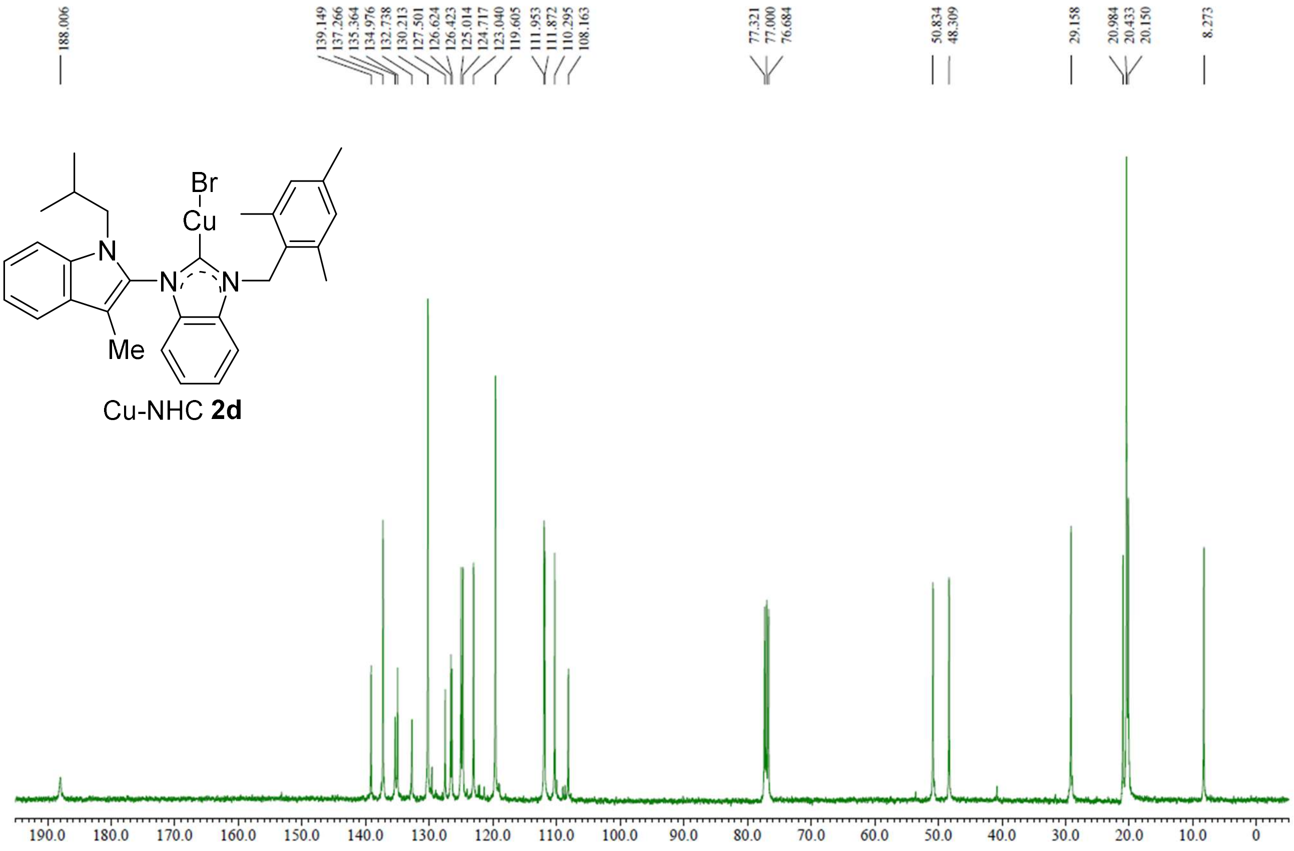

^{1}H NMR ($CDCl_3$, 400 MHz) spectrum of compound **2e**

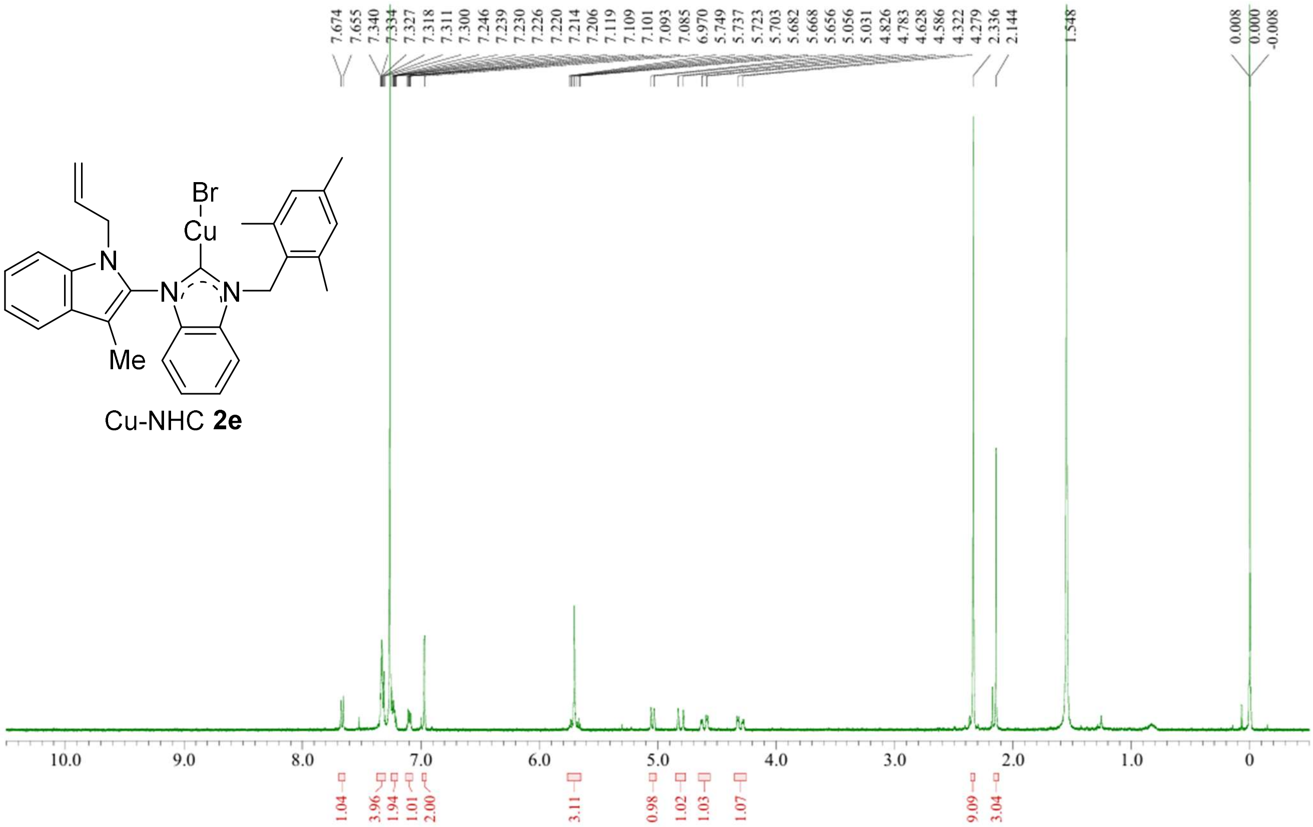

$^{13}C\{^{1}H\}$ NMR ($CDCl_3$, 100 MHz) spectrum of compound **2e**

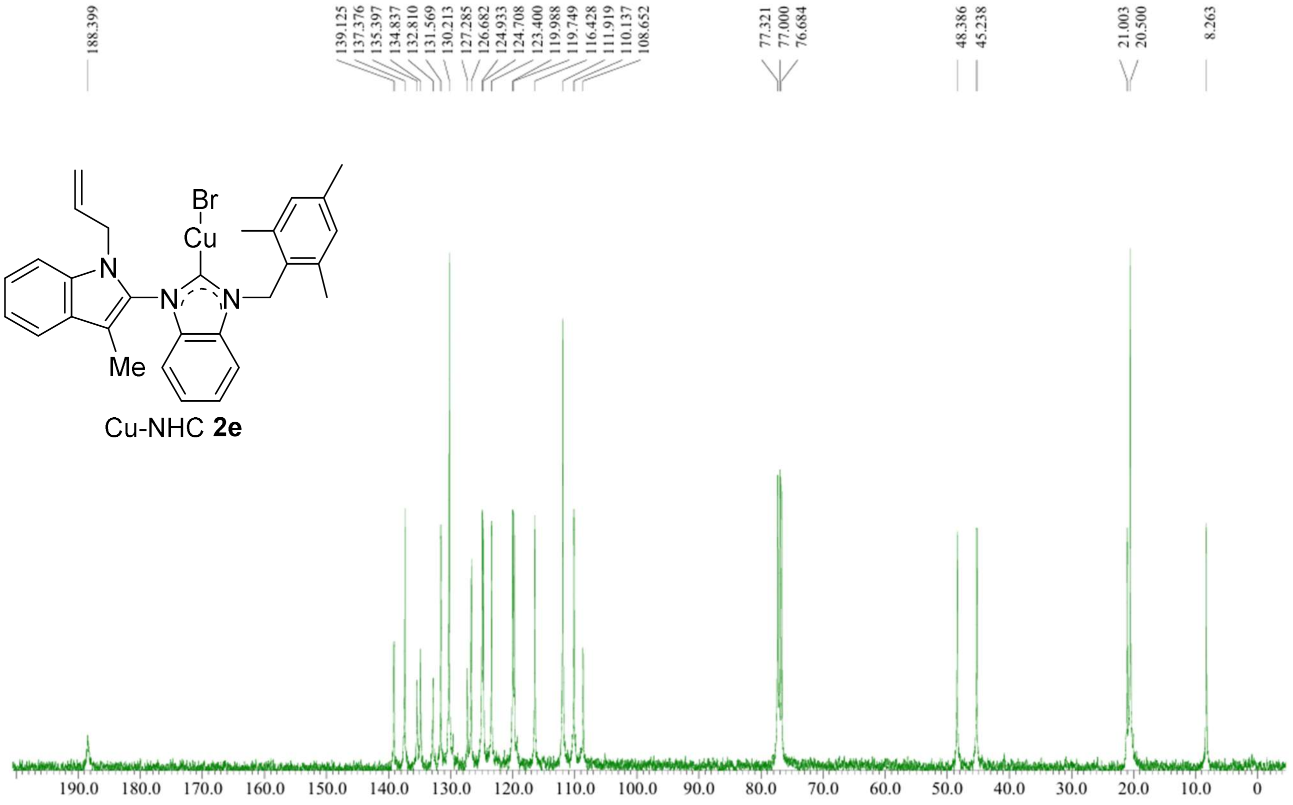

^{1}H NMR (CDCl3, 400 MHz) spectrum of compound **3**

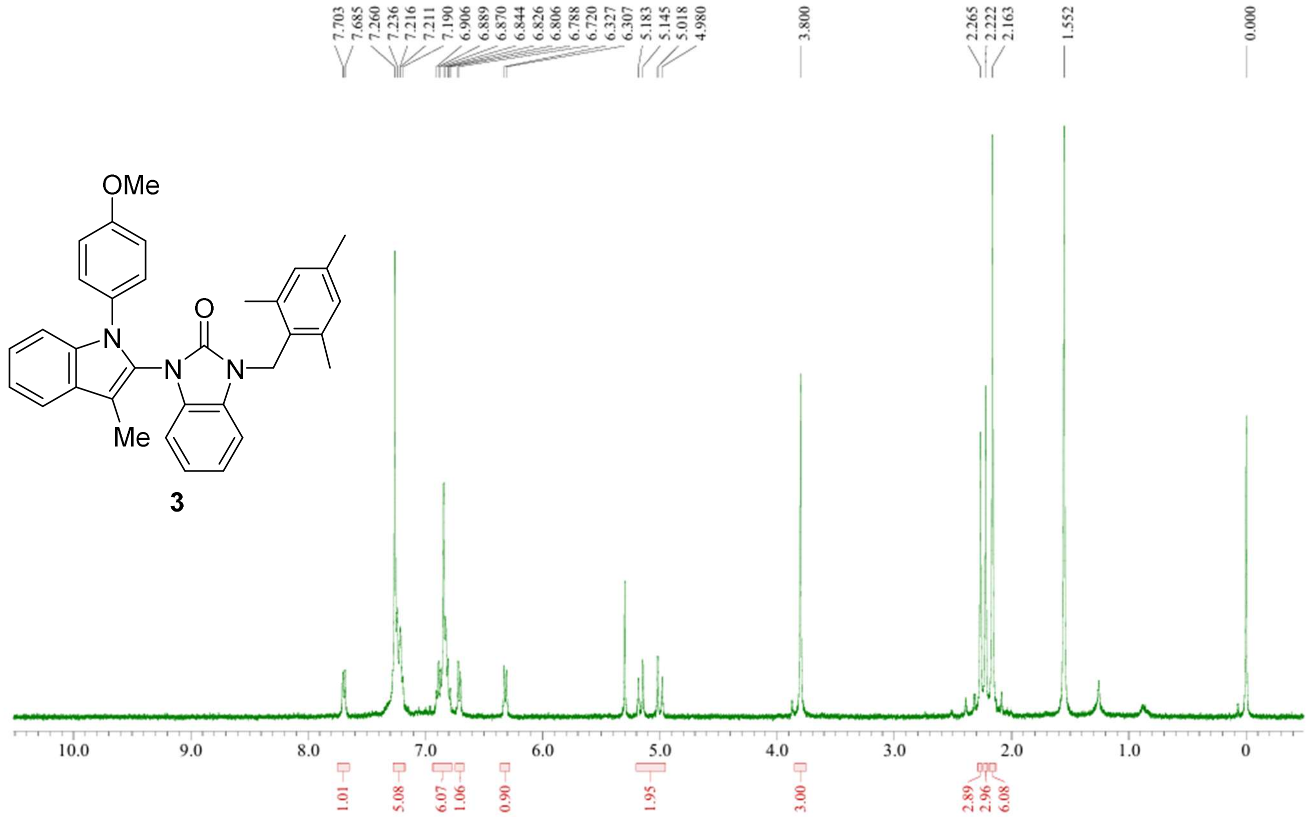

$^{13}C\{^{1}H\}$ NMR (CDCl3, 100 MHz) spectrum of compound **3**

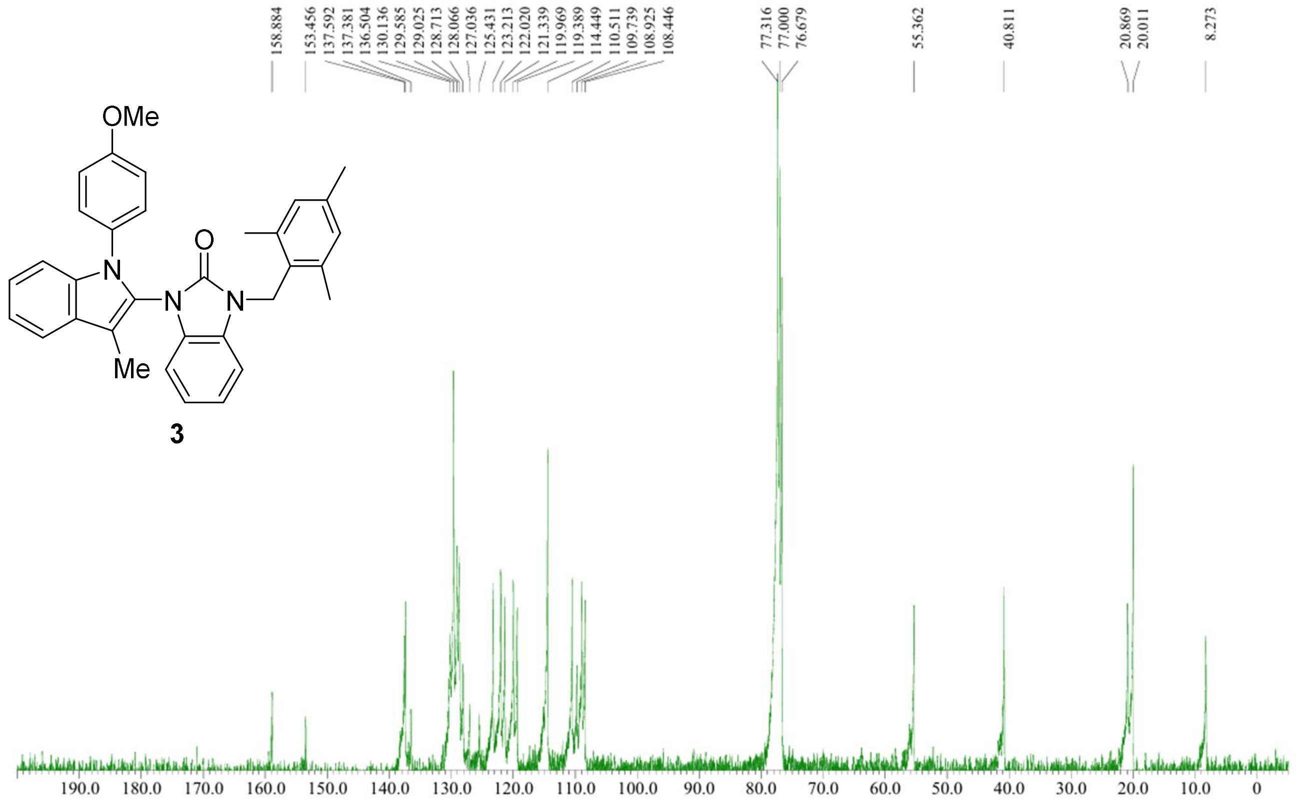

^{1}H NMR ($CDCl_3$, 400 MHz) spectrum of compound **5a**

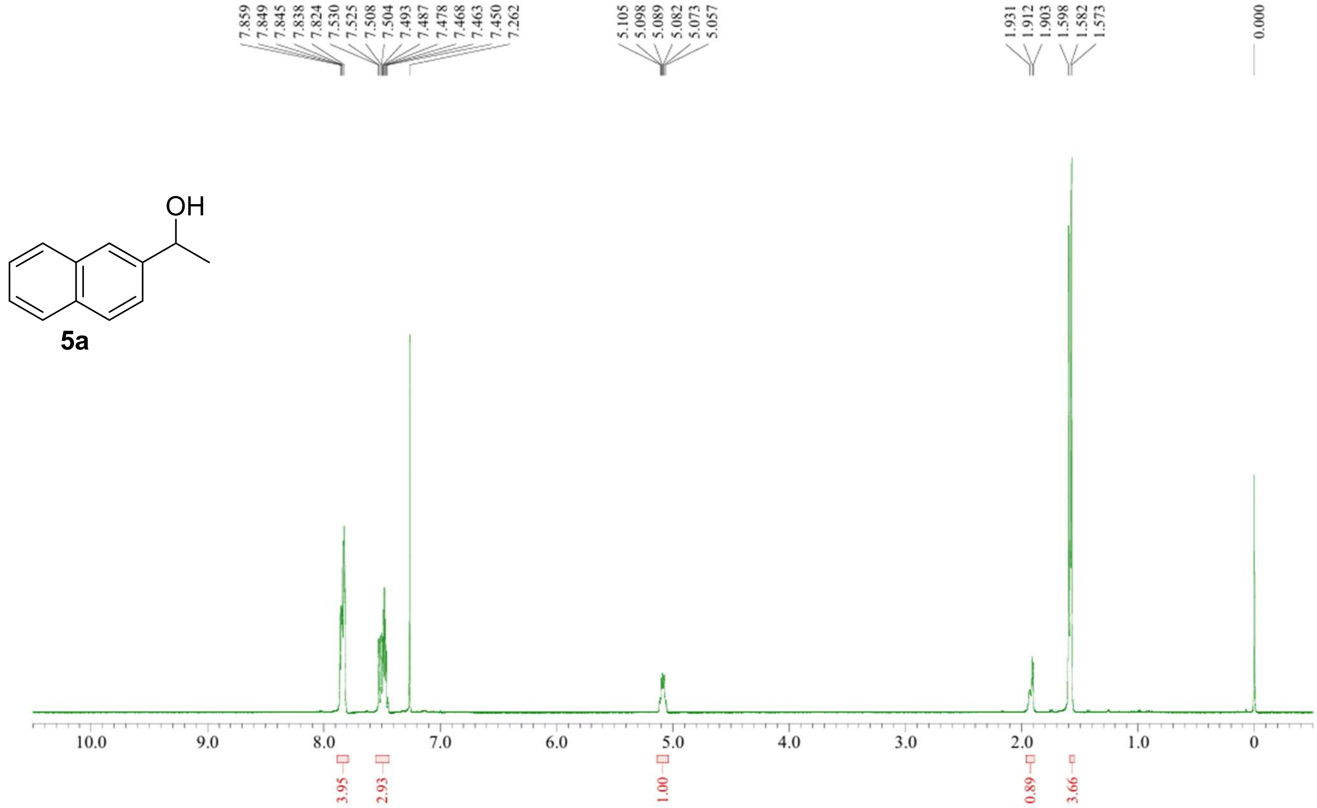

$^{13}C\{^{1}H\}$ NMR ($CDCl_3$, 100 MHz) spectrum of compound **5a**

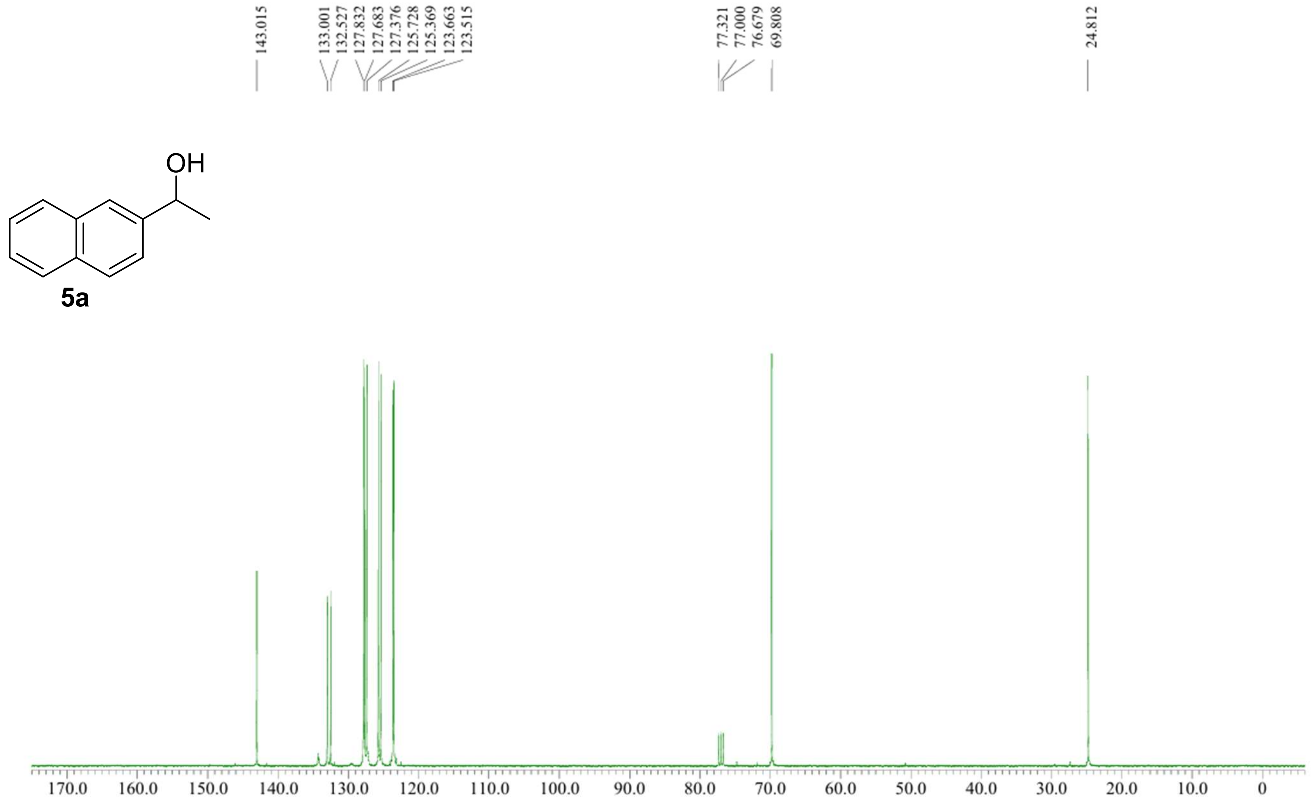

^{1}H NMR ($CDCl_3$, 400 MHz) spectrum of compound **5b**

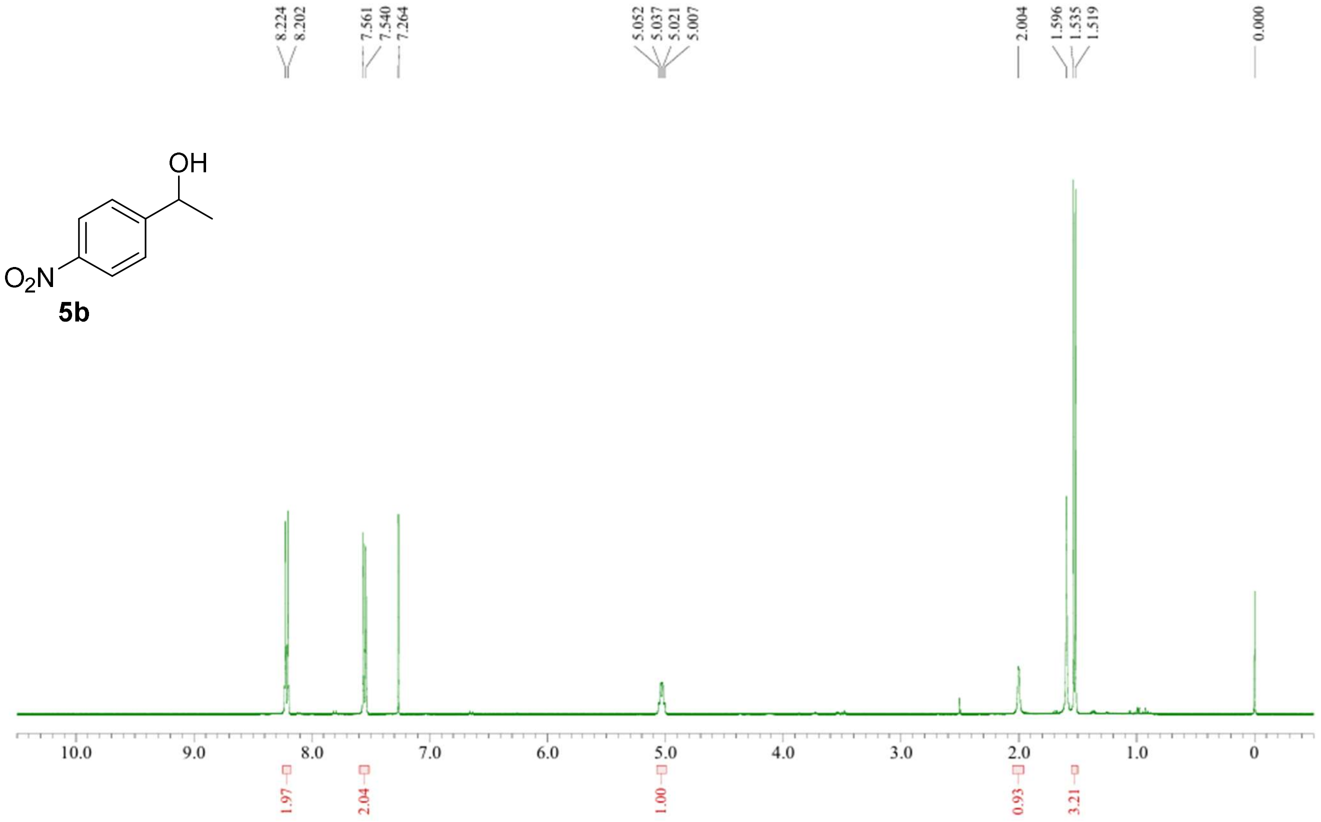

^{13}C{^{1}H} NMR ($CDCl_3$, 100 MHz) spectrum of compound **5b**

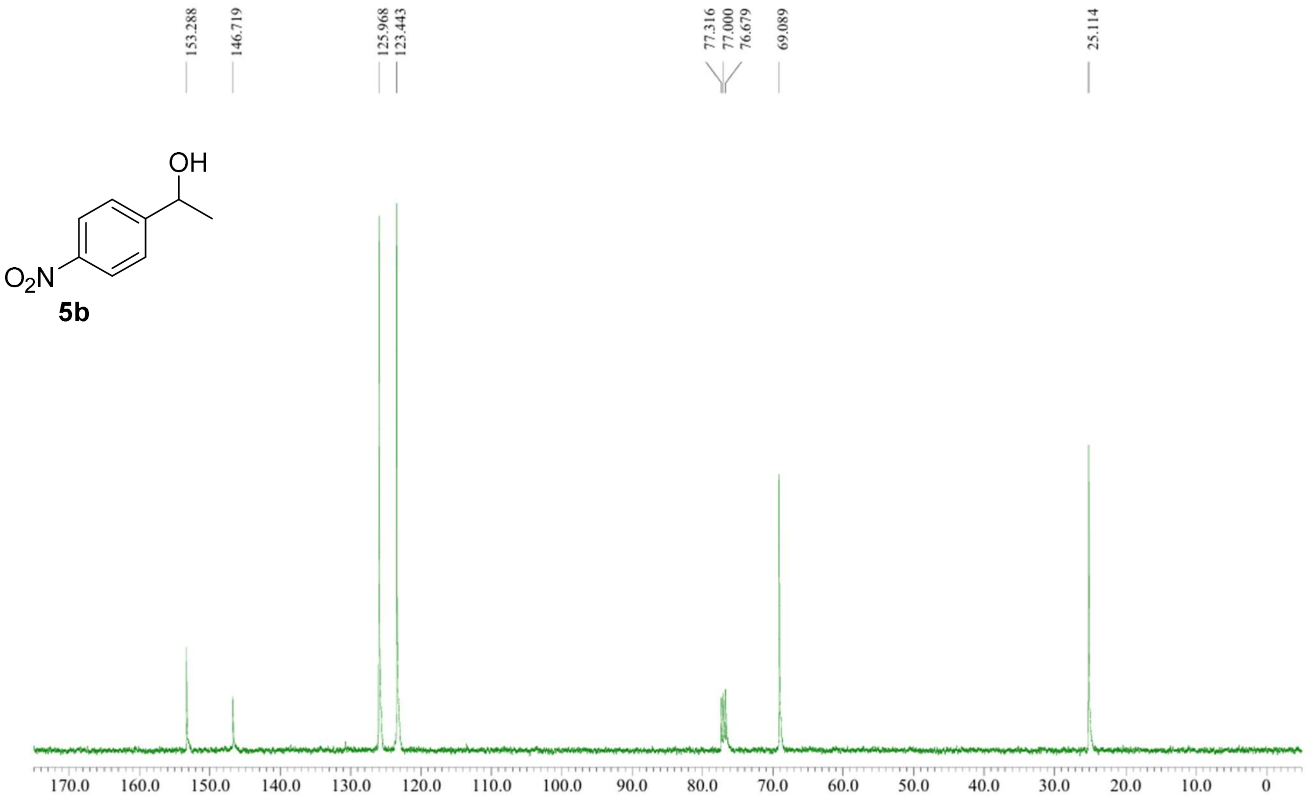

^{1}H NMR ($CDCl_3$, 400 MHz) spectrum of compound **5c**

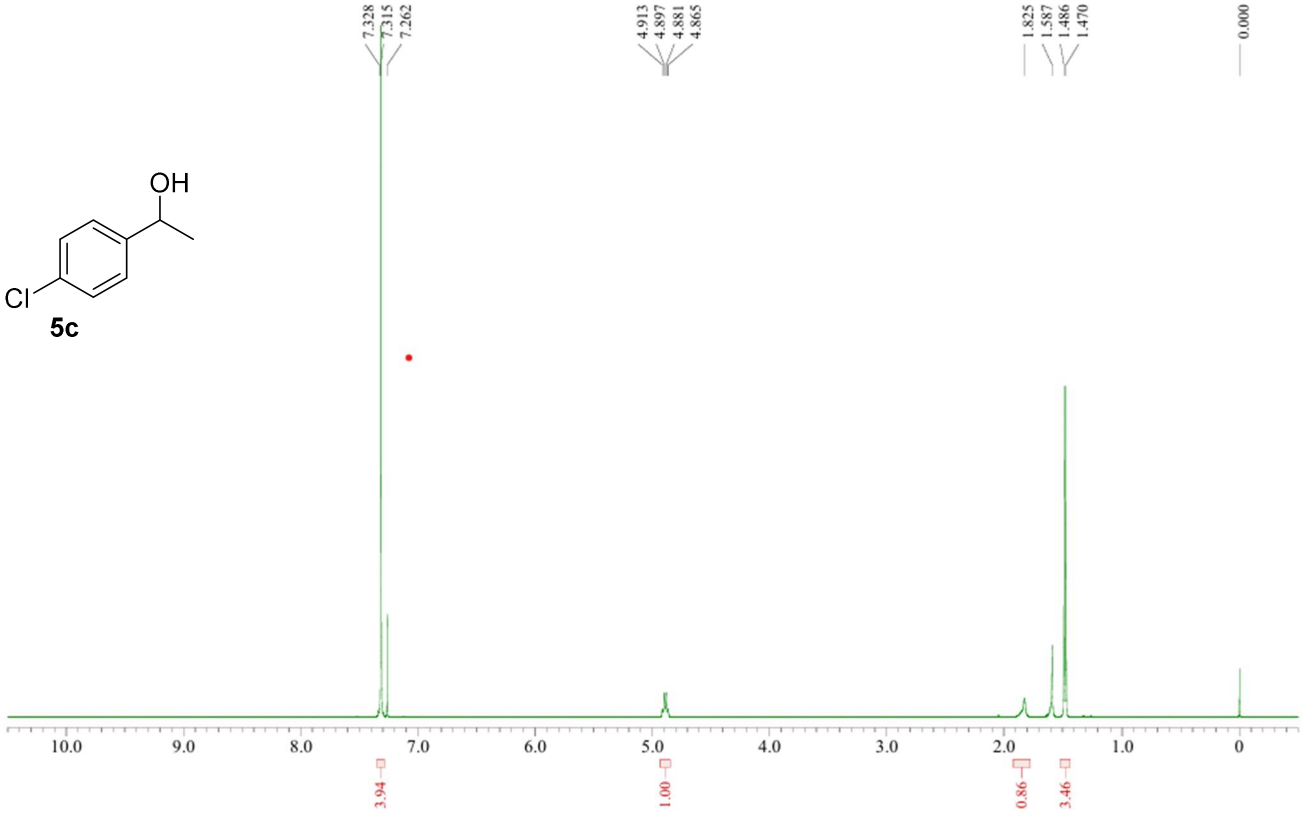

$^{13}C\{^1H\}$ NMR ($CDCl_3$, 100 MHz) spectrum of compound **5c**

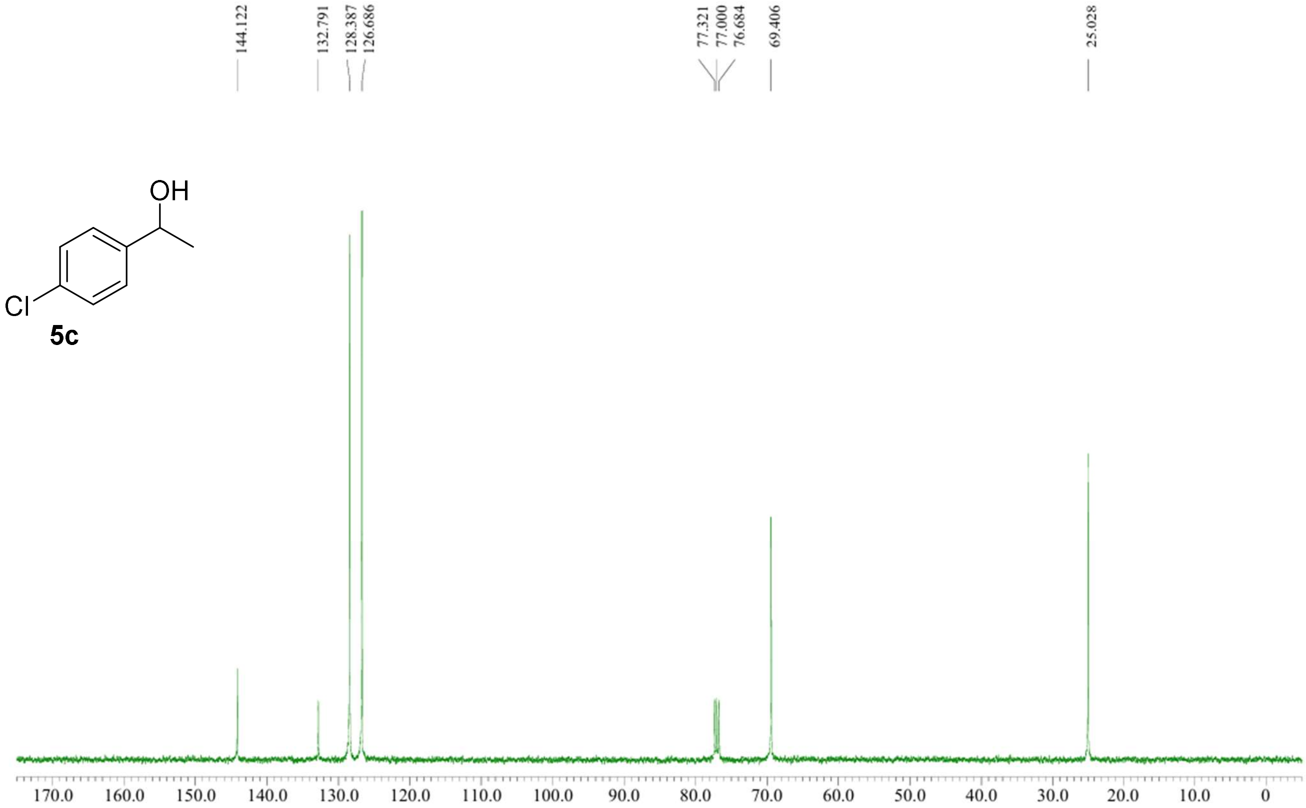

^{1}H NMR ($CDCl_3$, 400 MHz) spectrum of compound **5d**

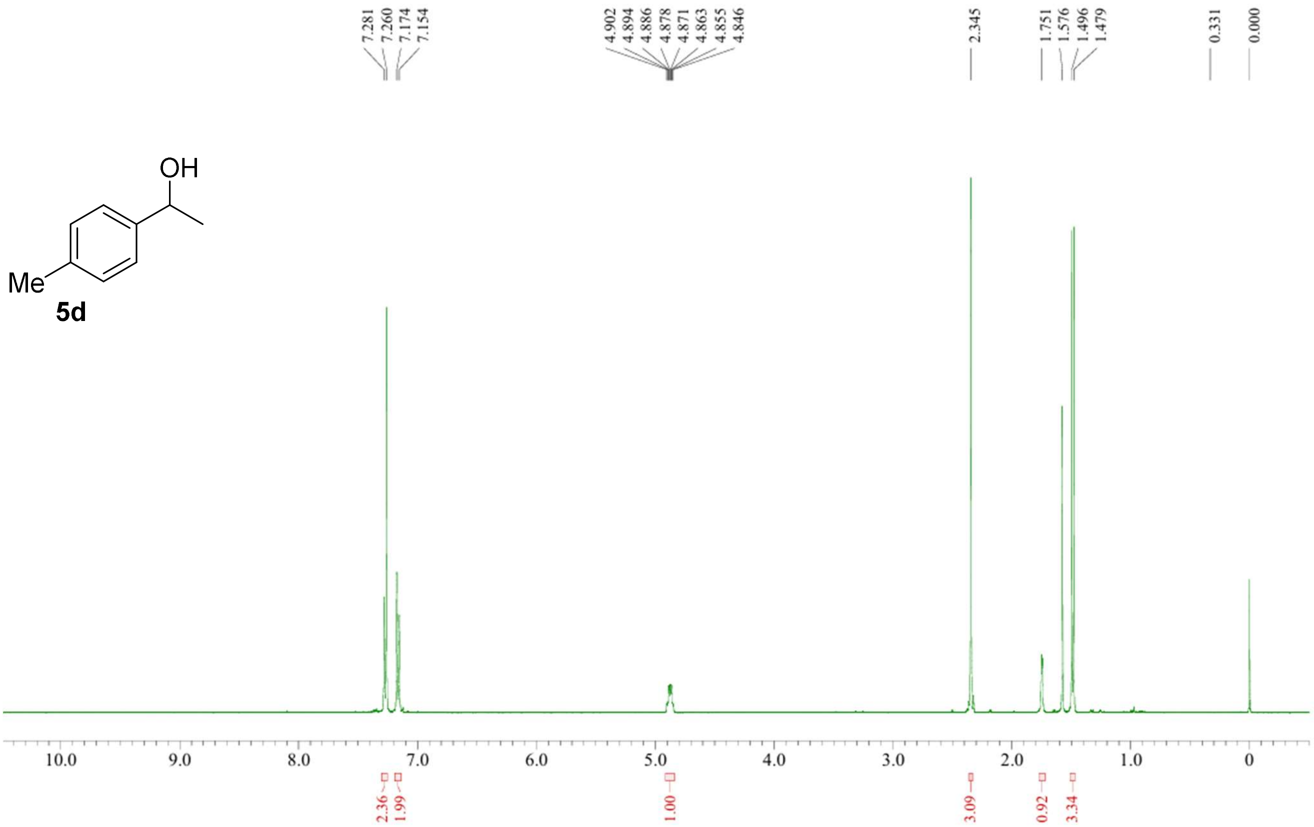

$^{13}C\{^{1}H\}$ NMR ($CDCl_3$, 100 MHz) spectrum of compound **5d**

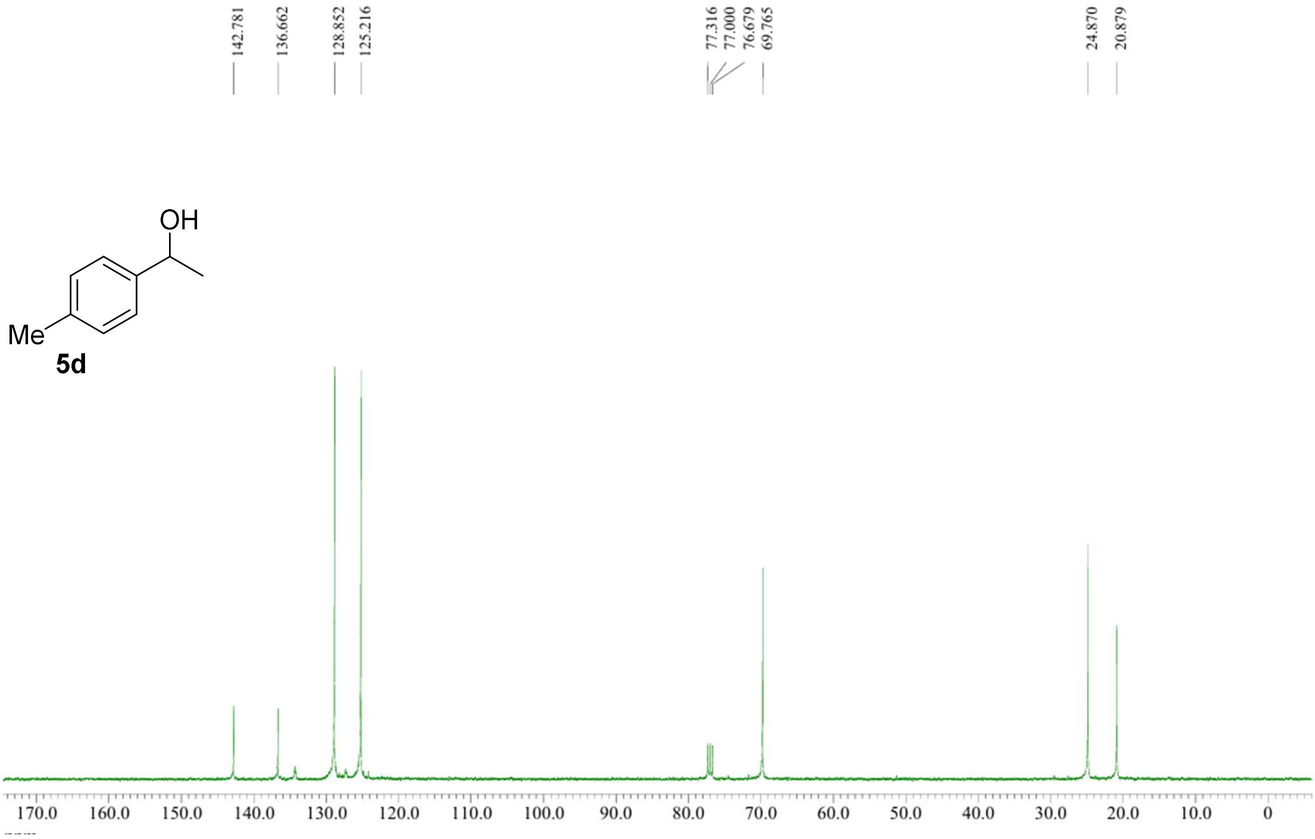

^{1}H NMR ($CDCl_3$, 400 MHz) spectrum of compound **5e**

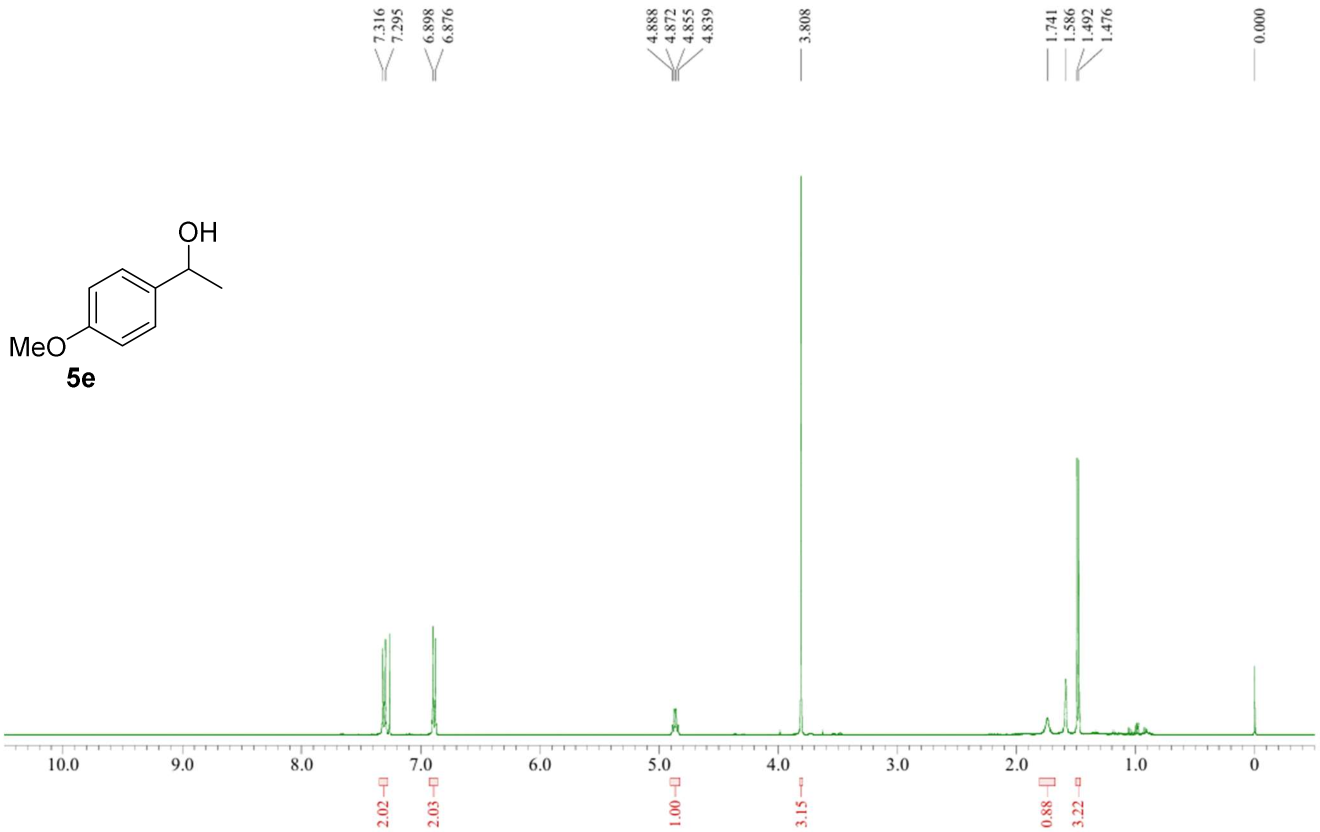

^{13}C{^{1}H} NMR ($CDCl_3$, 100 MHz) spectrum of compound **5e**

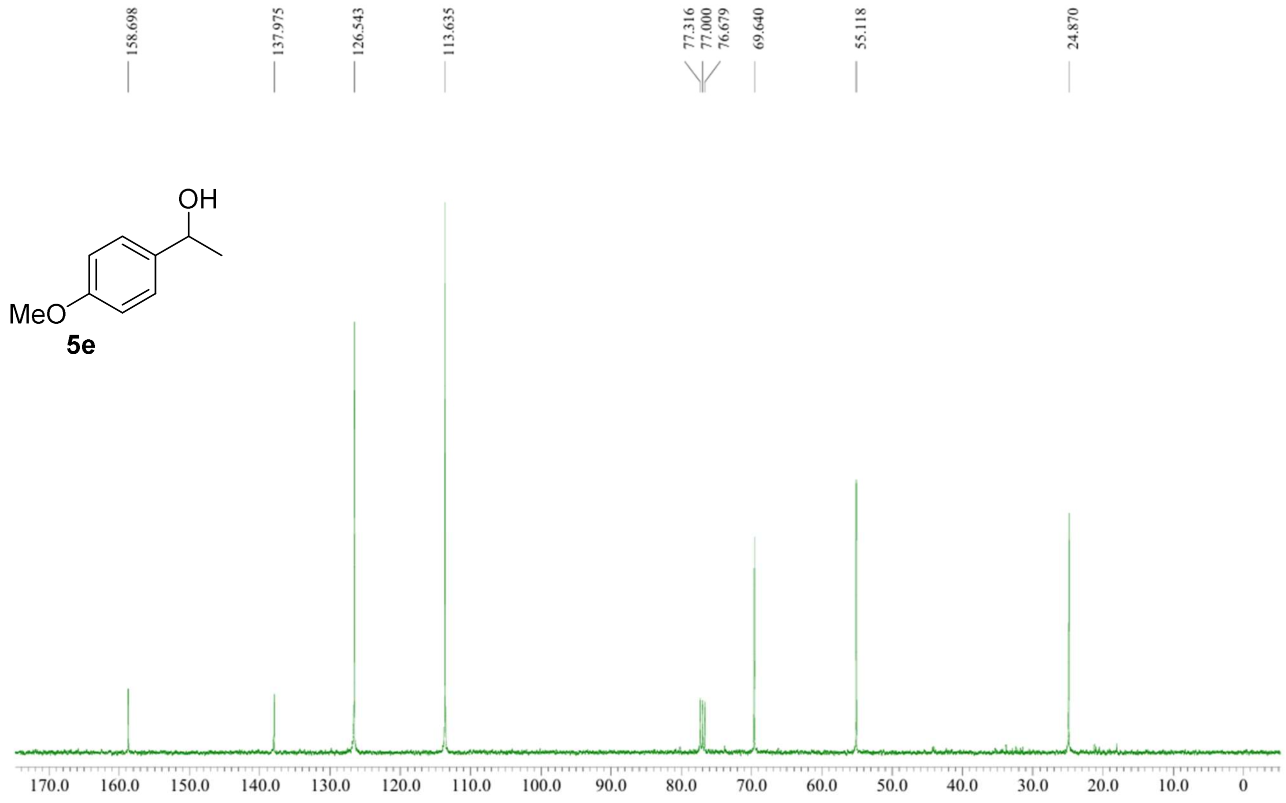

^{1}H NMR ($CDCl_3$, 400 MHz) spectrum of compound **5f**

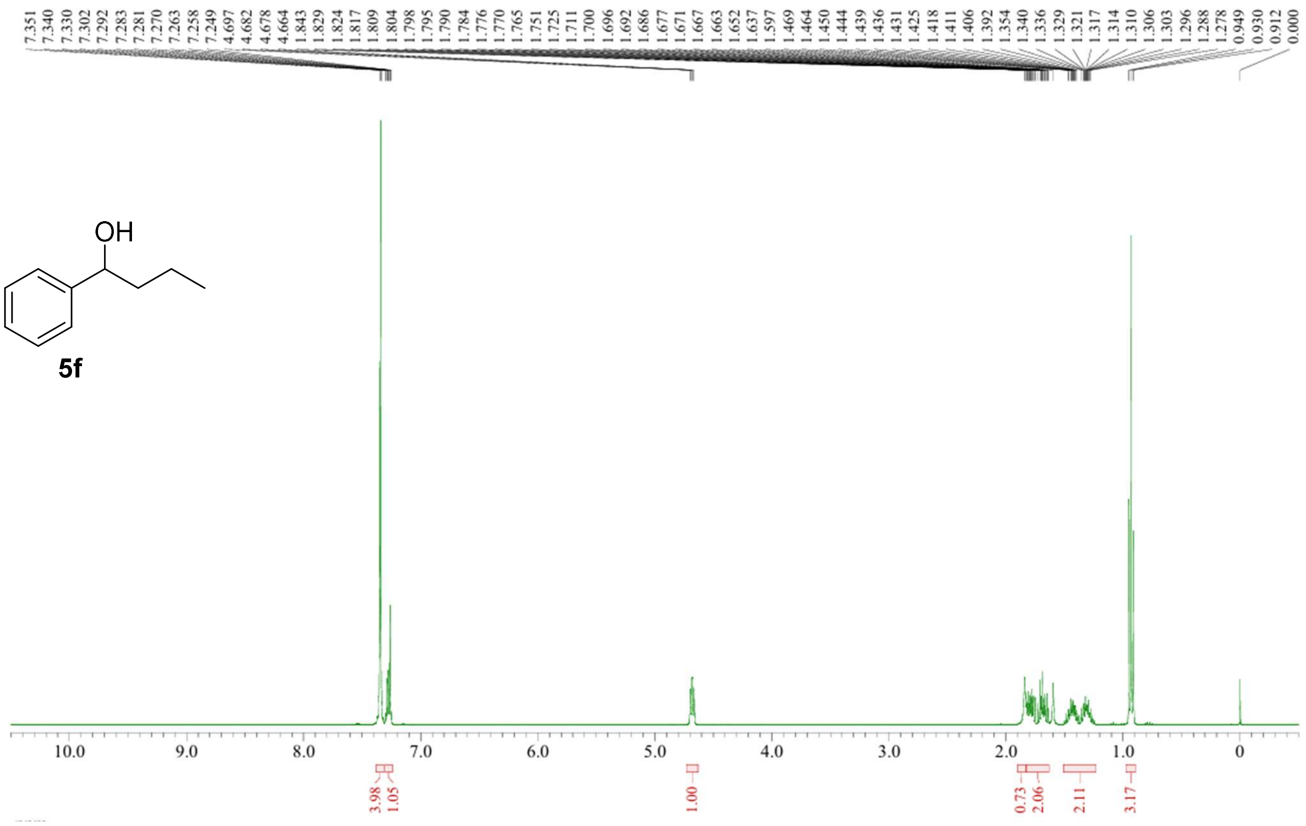

$^{13}C\{^{1}H\}$ NMR ($CDCl_3$, 100 MHz) spectrum of compound **5f**

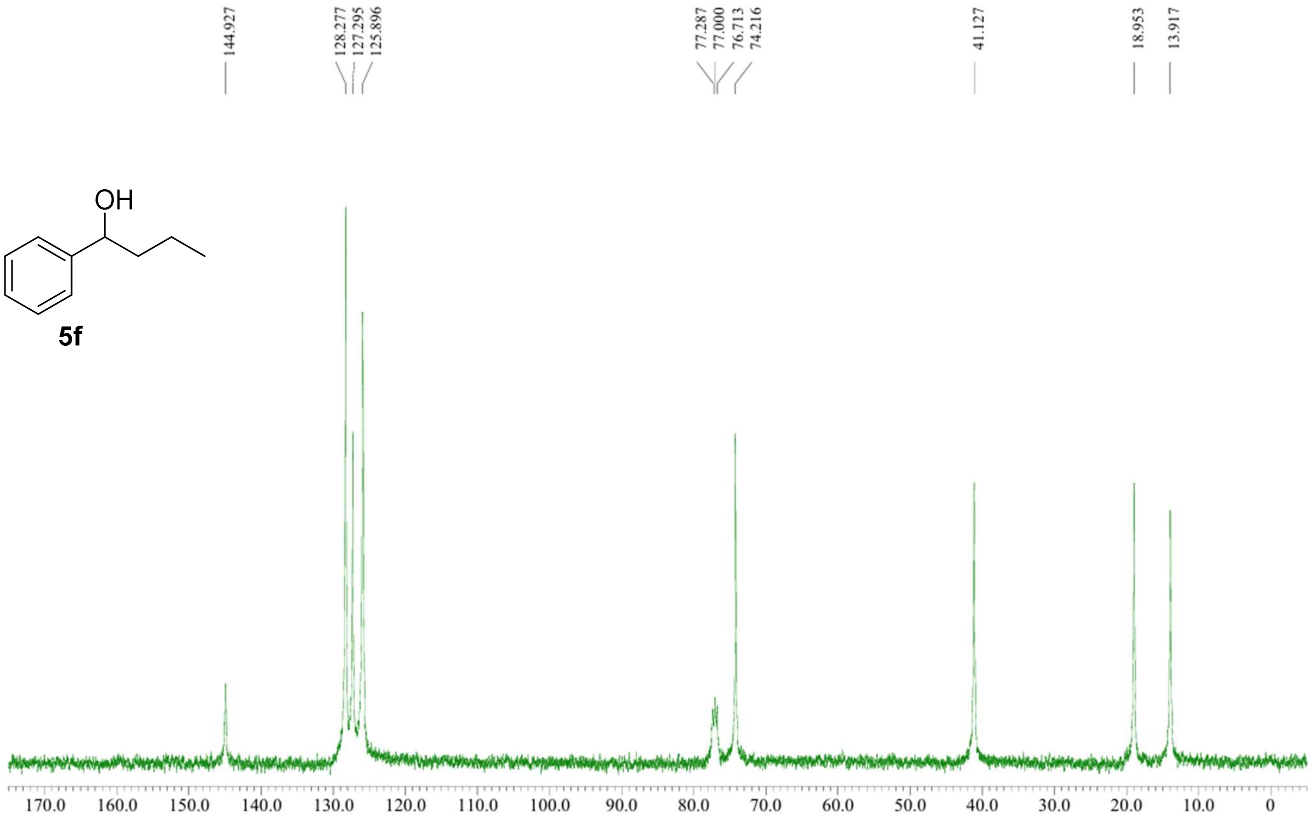

^{1}H NMR ($CDCl_3$, 400 MHz) spectrum of compound **5g**

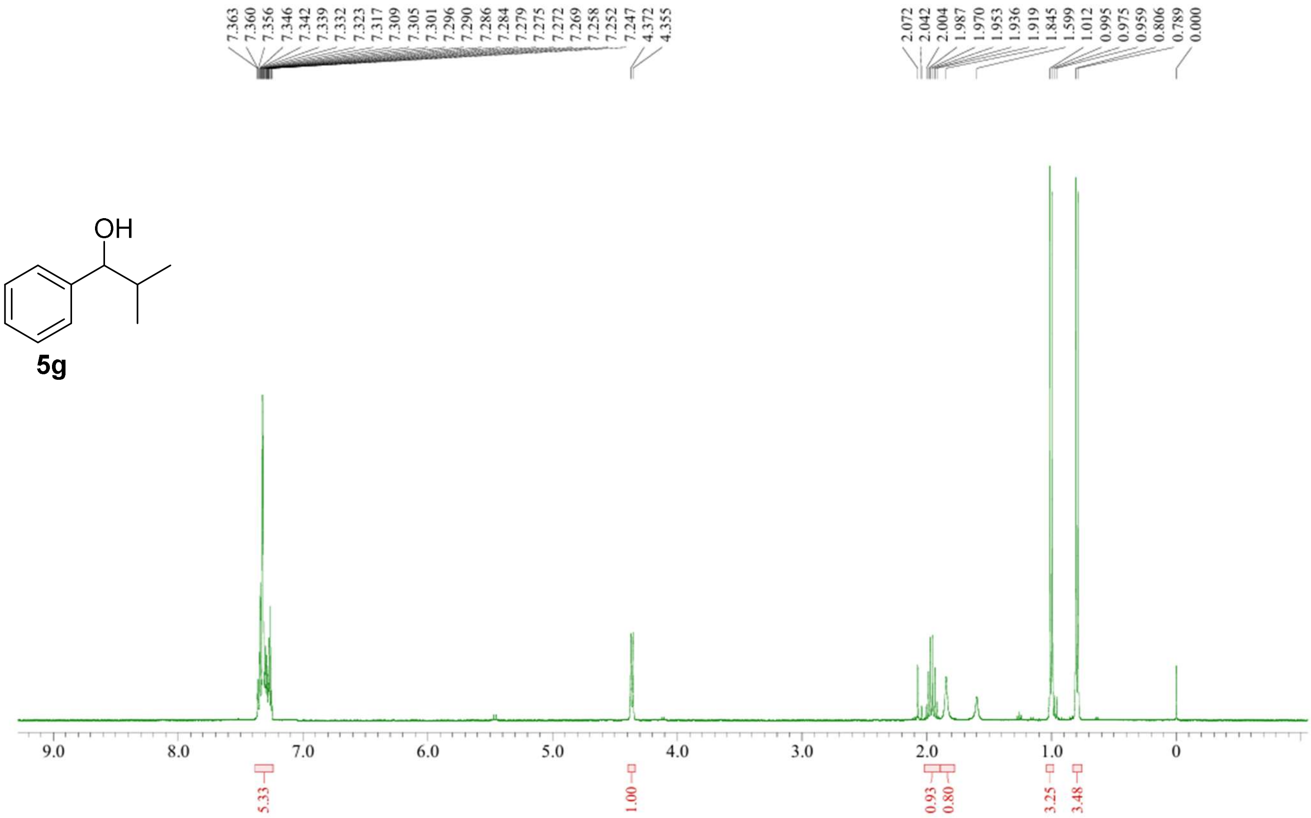

$^{13}C\{^{1}H\}$ NMR ($CDCl_3$, 100 MHz) spectrum of compound **5g**

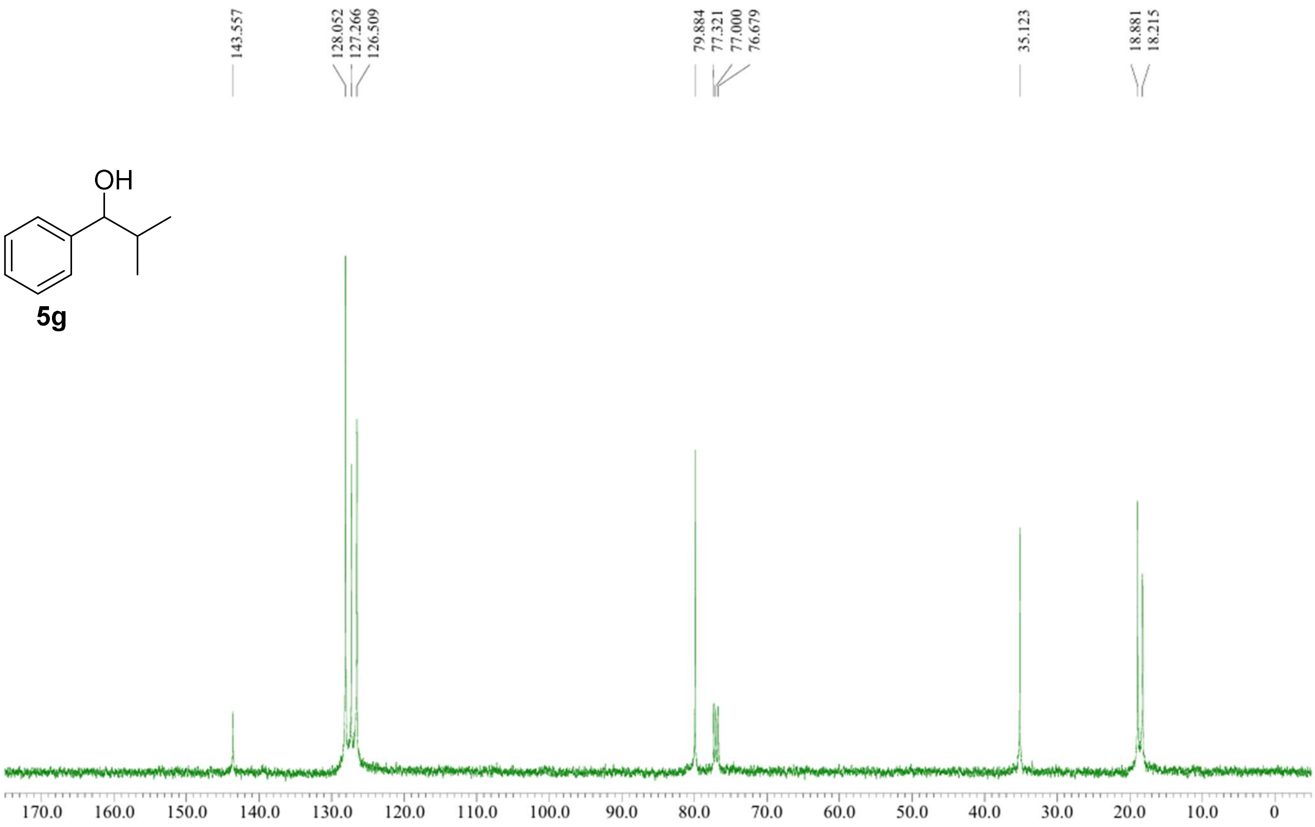

^{1}H NMR ($CDCl_3$, 400 MHz) spectrum of compound **5h**

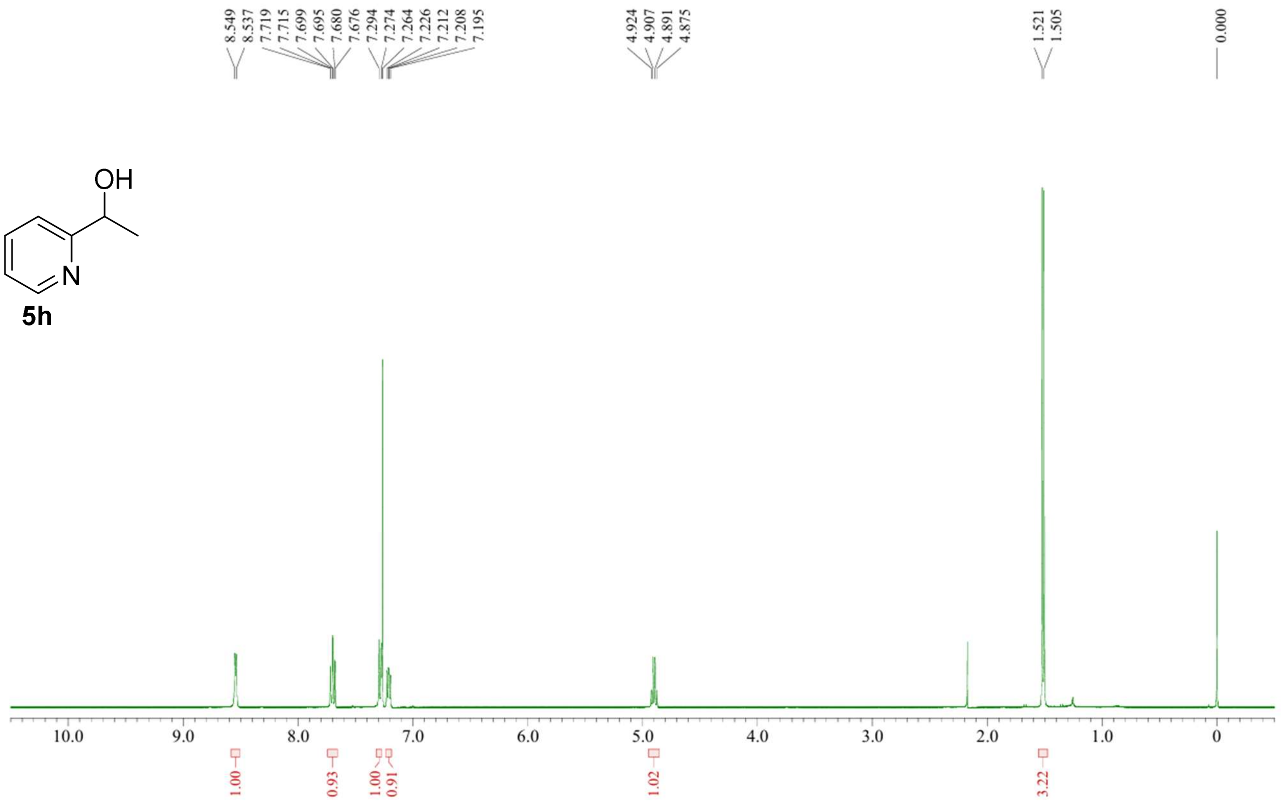

$^{13}C\{^{1}H\}$ NMR ($CDCl_3$, 100 MHz) spectrum of compound **5h**

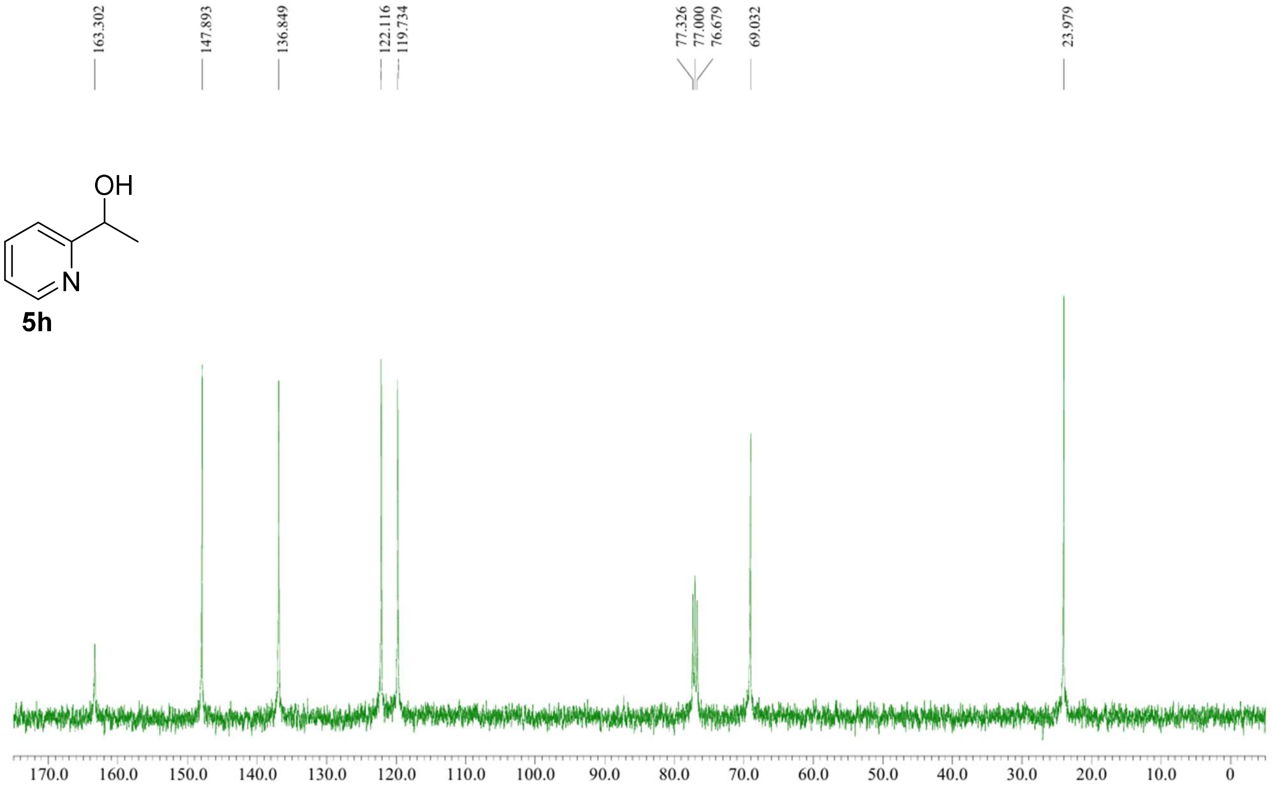

^{1}H NMR ($CDCl_3$, 400 MHz) spectrum of compound **5i**

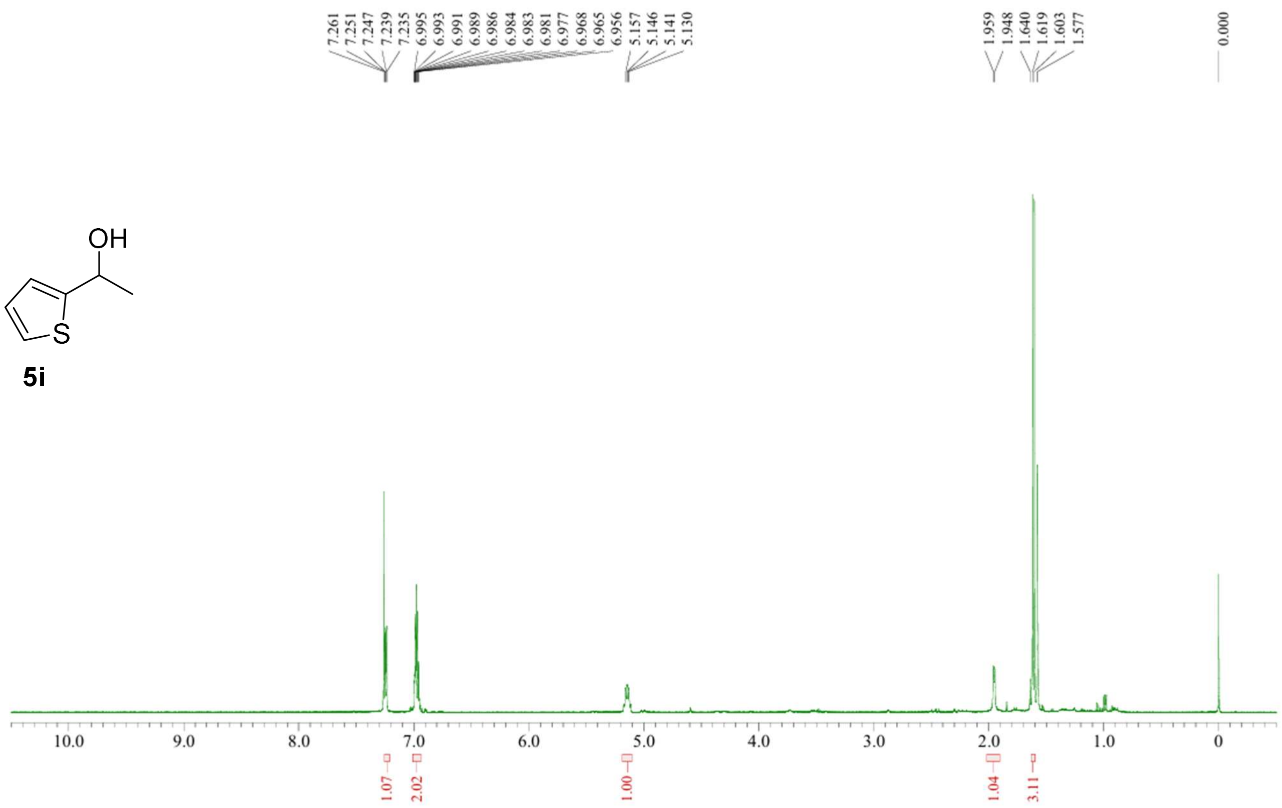

^{13}C{^{1}H} NMR ($CDCl_3$, 100 MHz) spectrum of compound **5i**

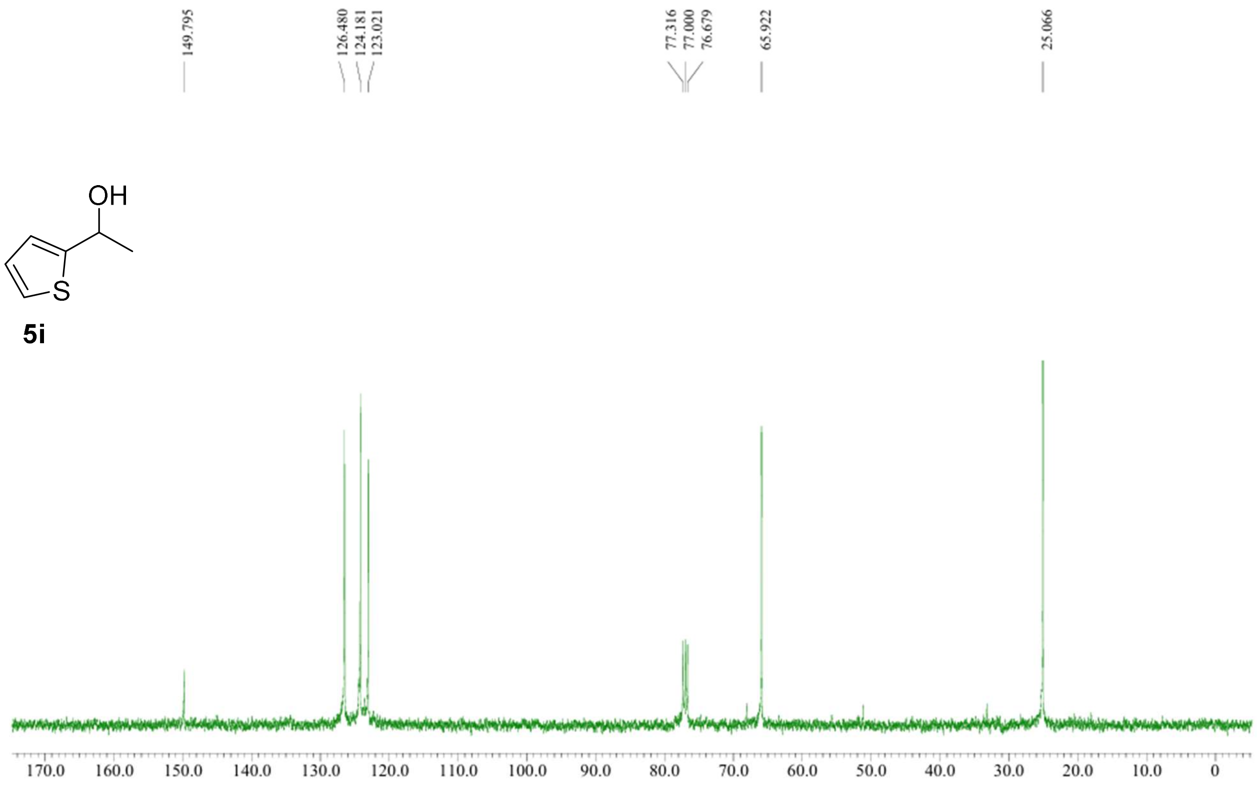

^{1}H NMR ($CDCl_3$, 400 MHz) spectrum of compound **5j**

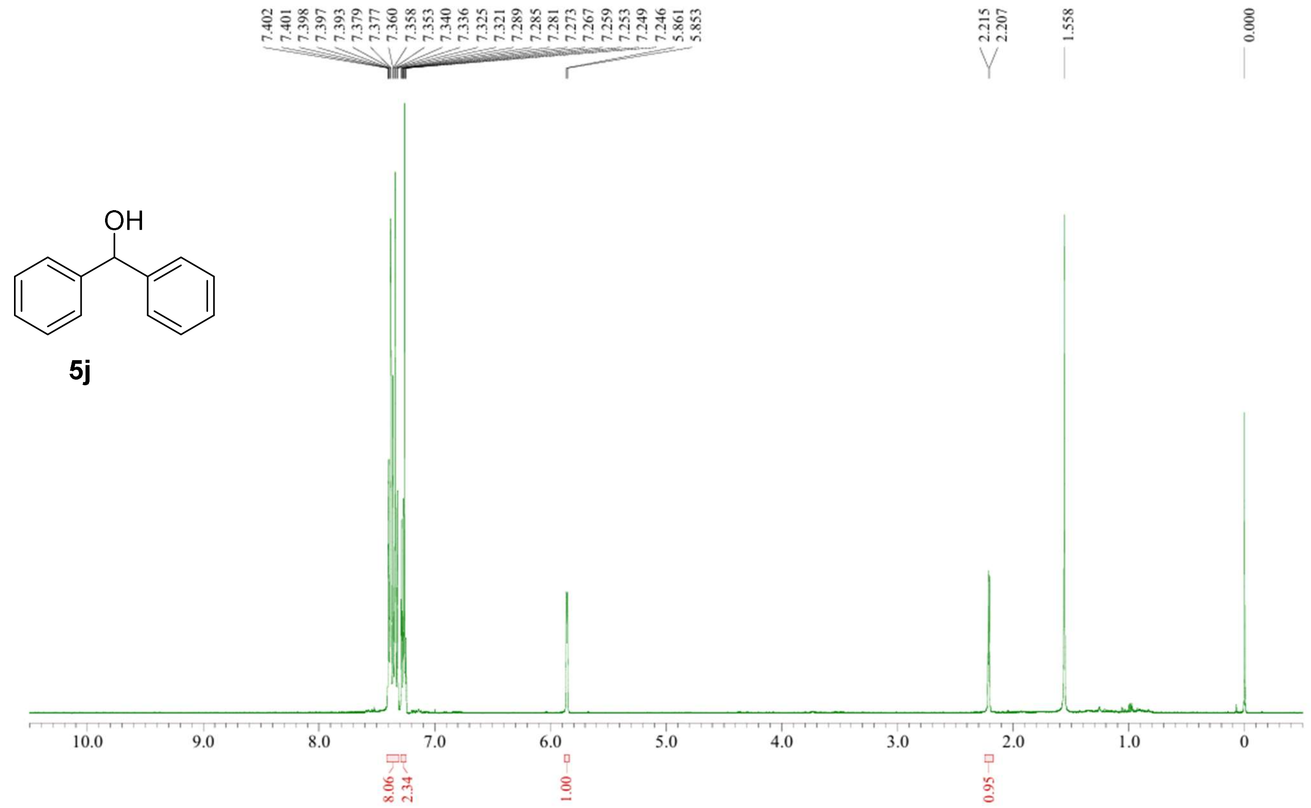

^{13}C{^{1}H} NMR ($CDCl_3$, 100 MHz) spectrum of compound **5j**

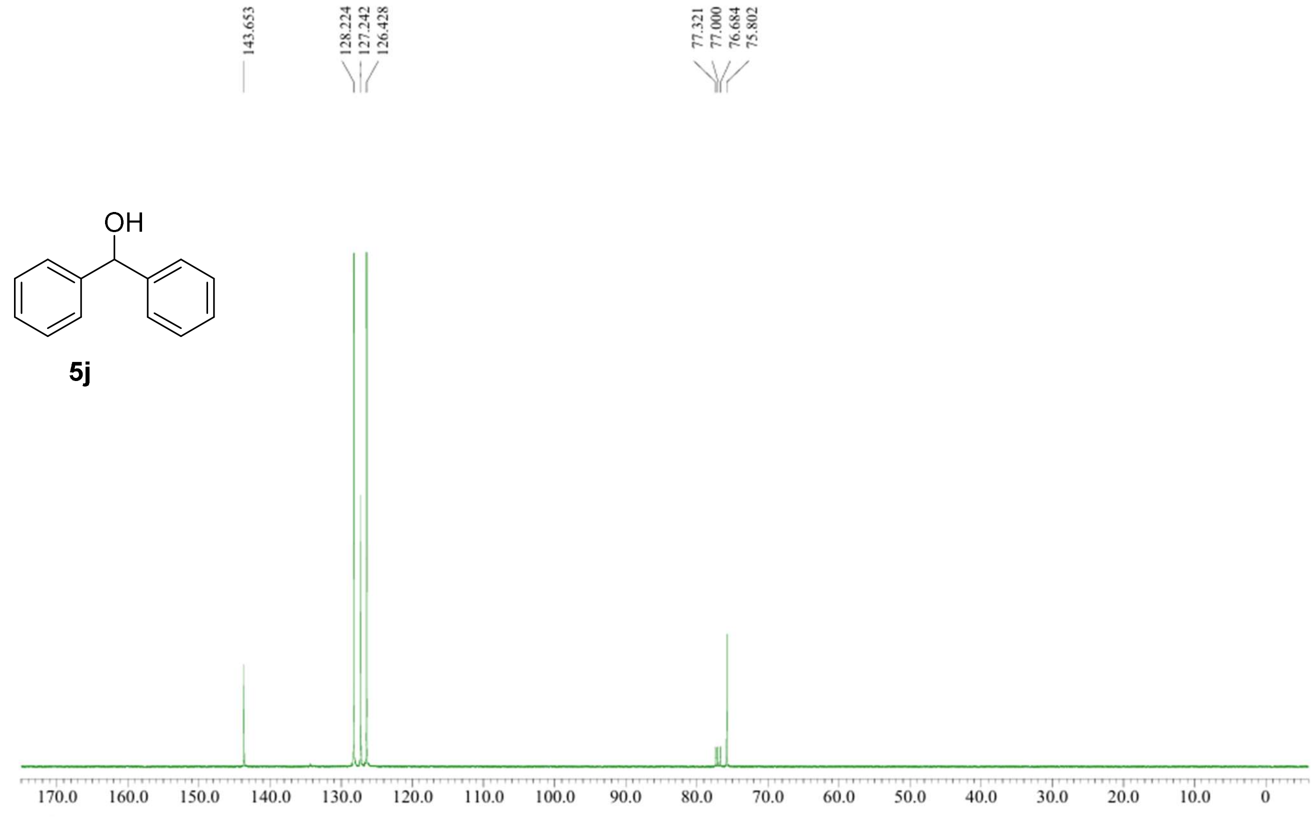

^{1}H NMR ($CDCl_3$, 400 MHz) spectrum of compound **5k**

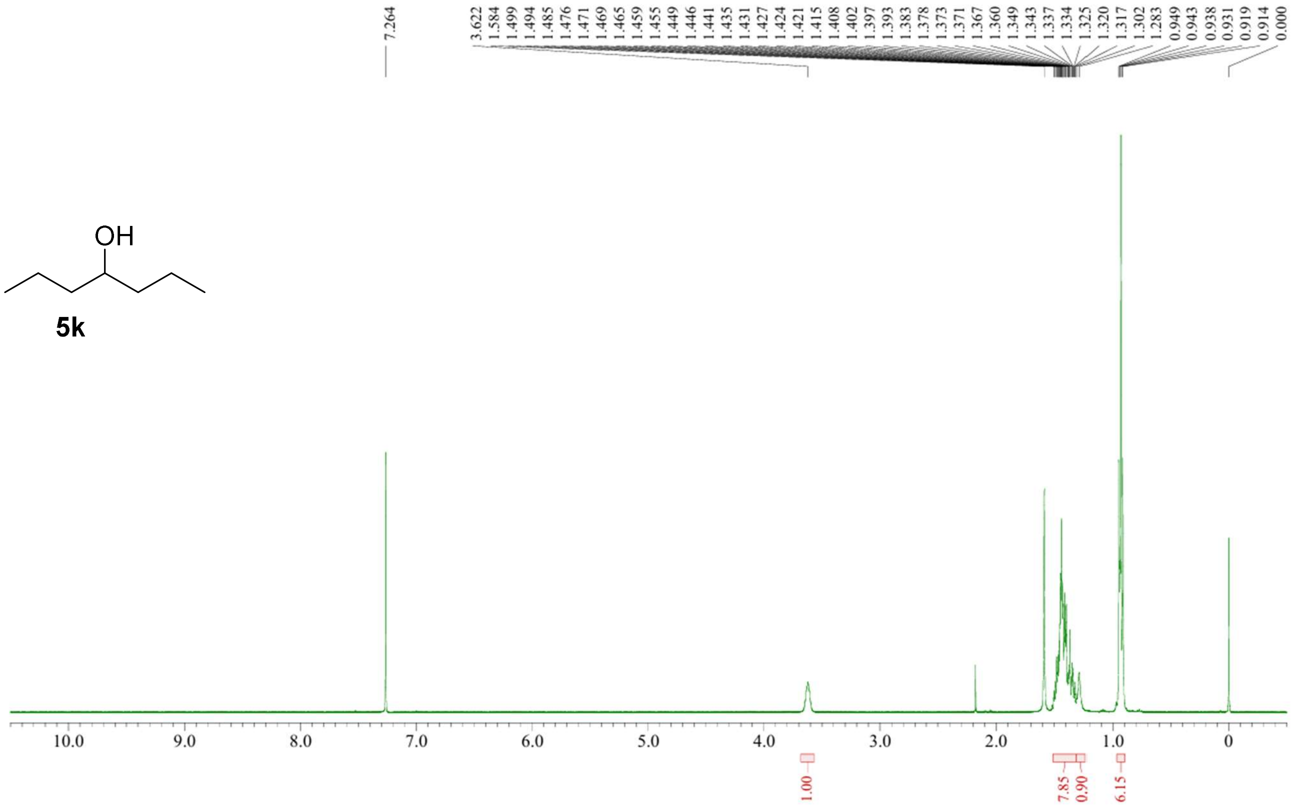

^{13}C{^{1}H} NMR ($CDCl_3$, 100 MHz) spectrum of compound **5k**

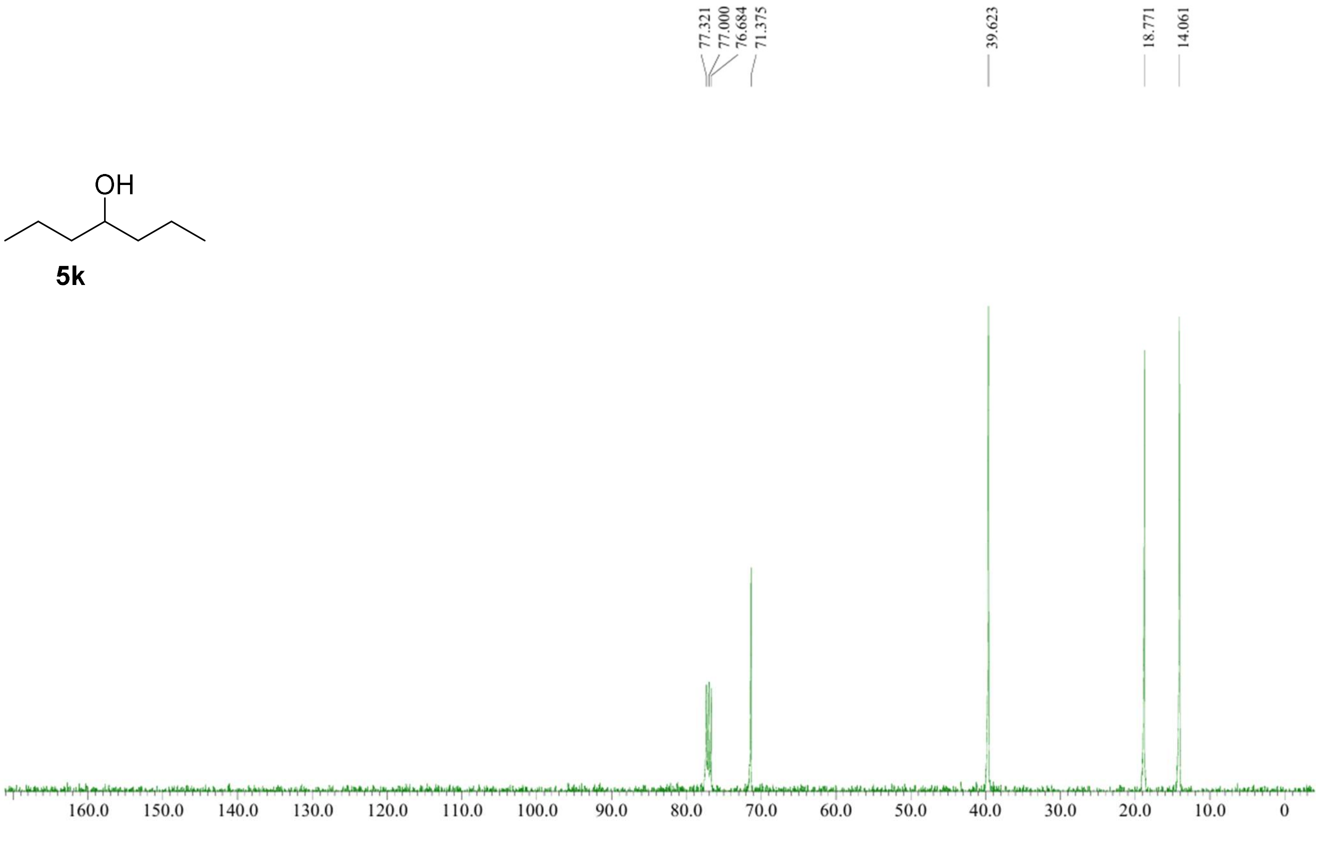

^{1}H NMR ($CDCl_3$, 400 MHz) spectrum of compound **5l**

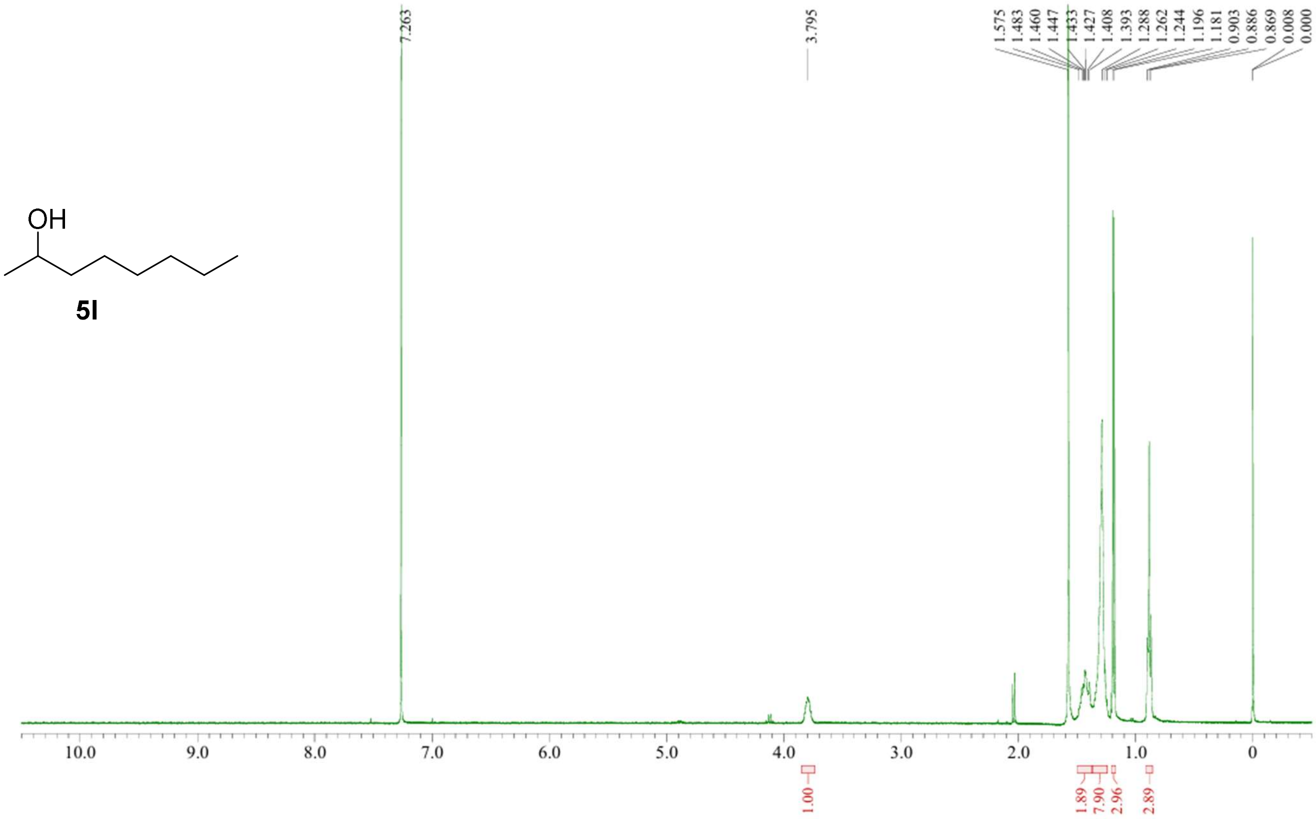

$^{13}C\{^{1}H\}$ NMR ($CDCl_3$, 100 MHz) spectrum of compound **5l**

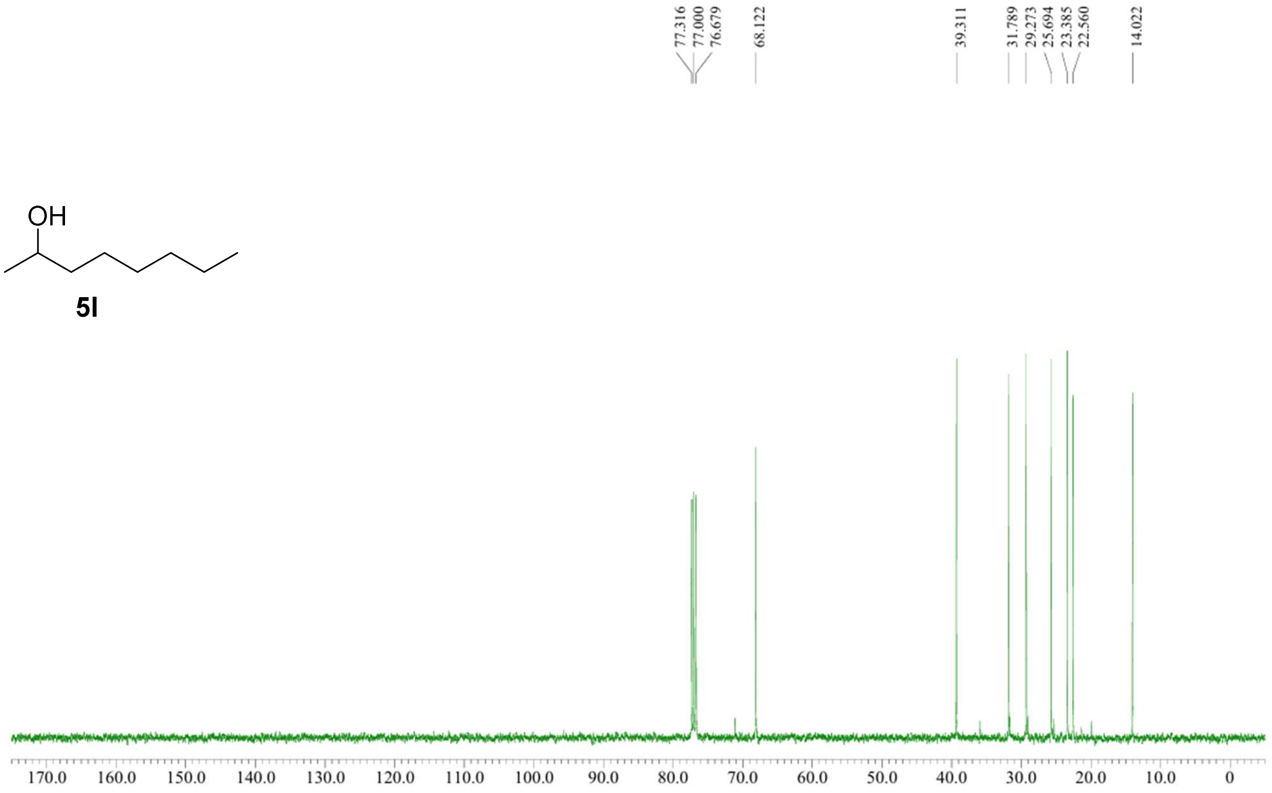

^{1}H NMR ($CDCl_3$, 400 MHz) spectrum of compound **5m**

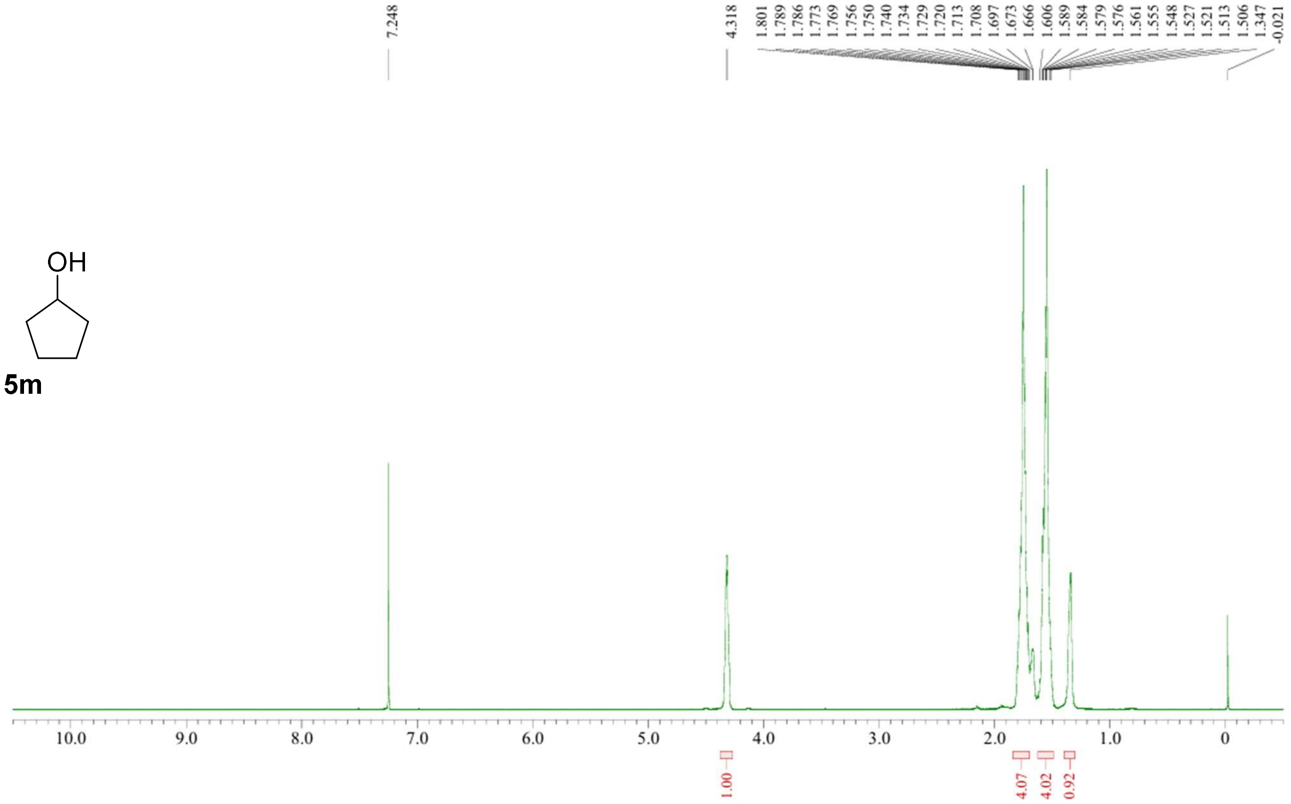

^{13}C{^{1}H} NMR ($CDCl_3$, 100 MHz) spectrum of compound **5m**

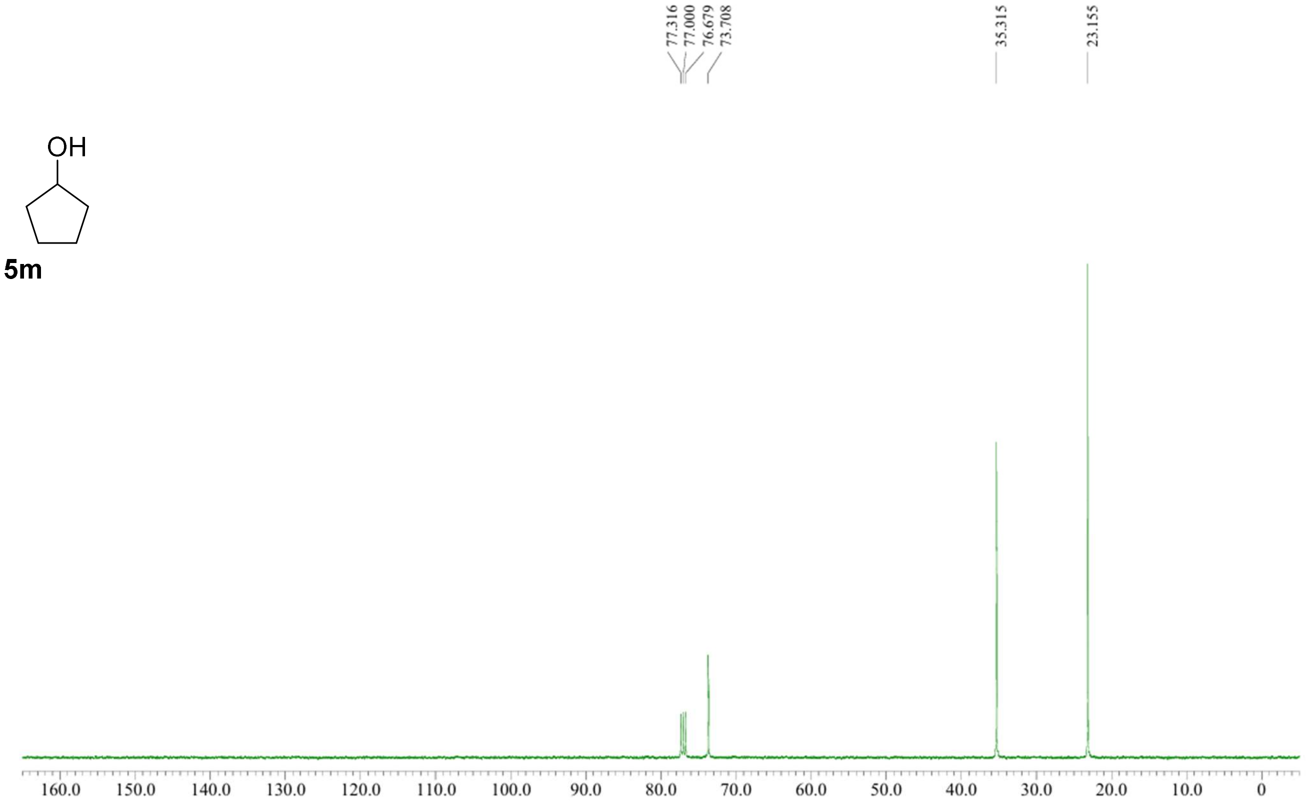

^{1}H NMR ($CDCl_3$, 400 MHz) spectrum of compound **5n**

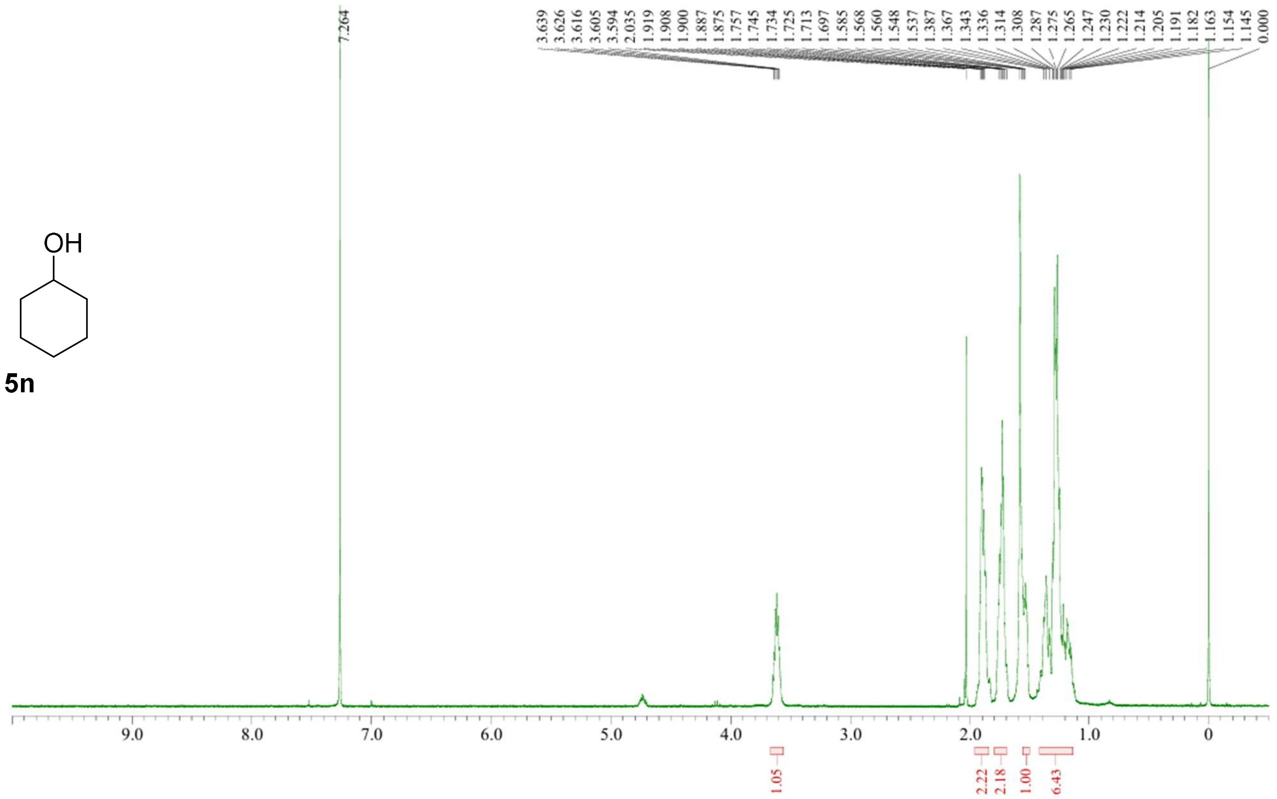

^{13}C{^{1}H} NMR ($CDCl_3$, 100 MHz) spectrum of compound **5n**

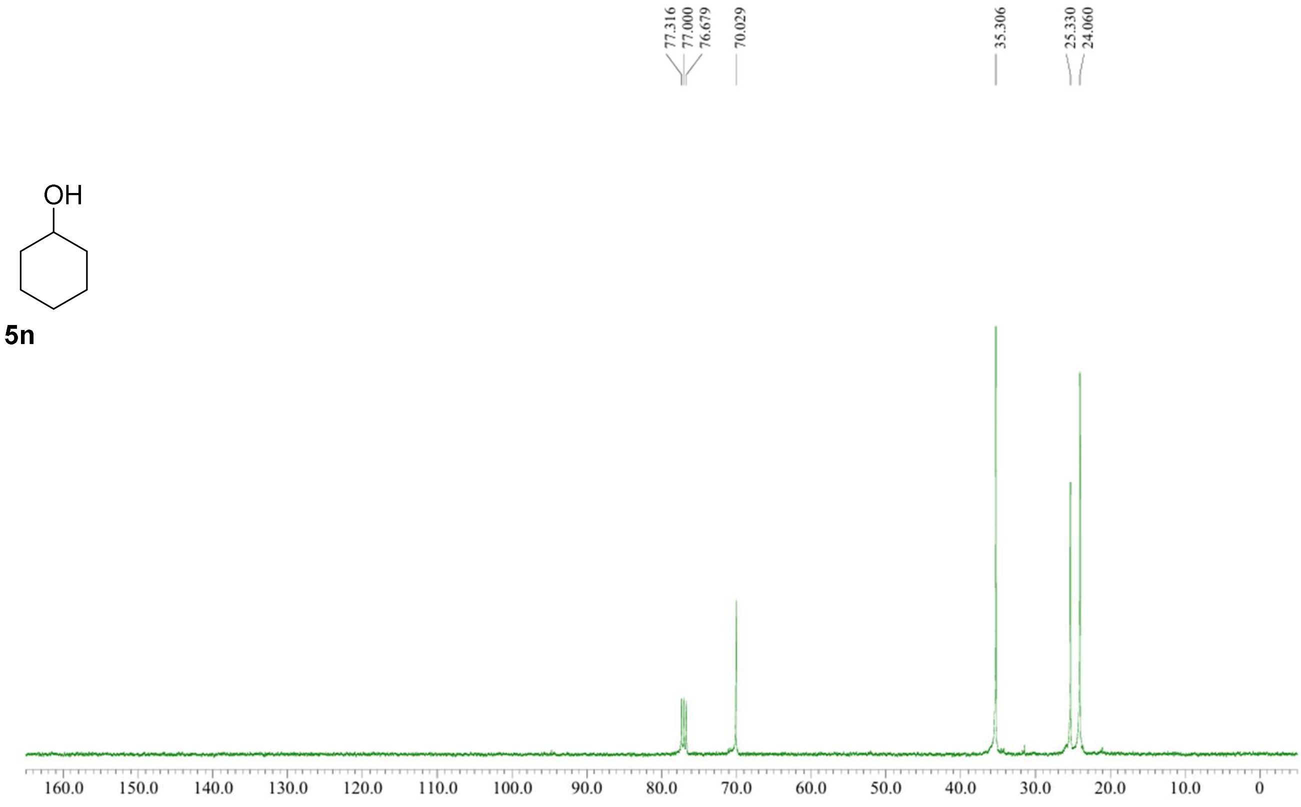

^{1}H NMR ($CDCl_3$, 400 MHz) spectrum of compound **5o**

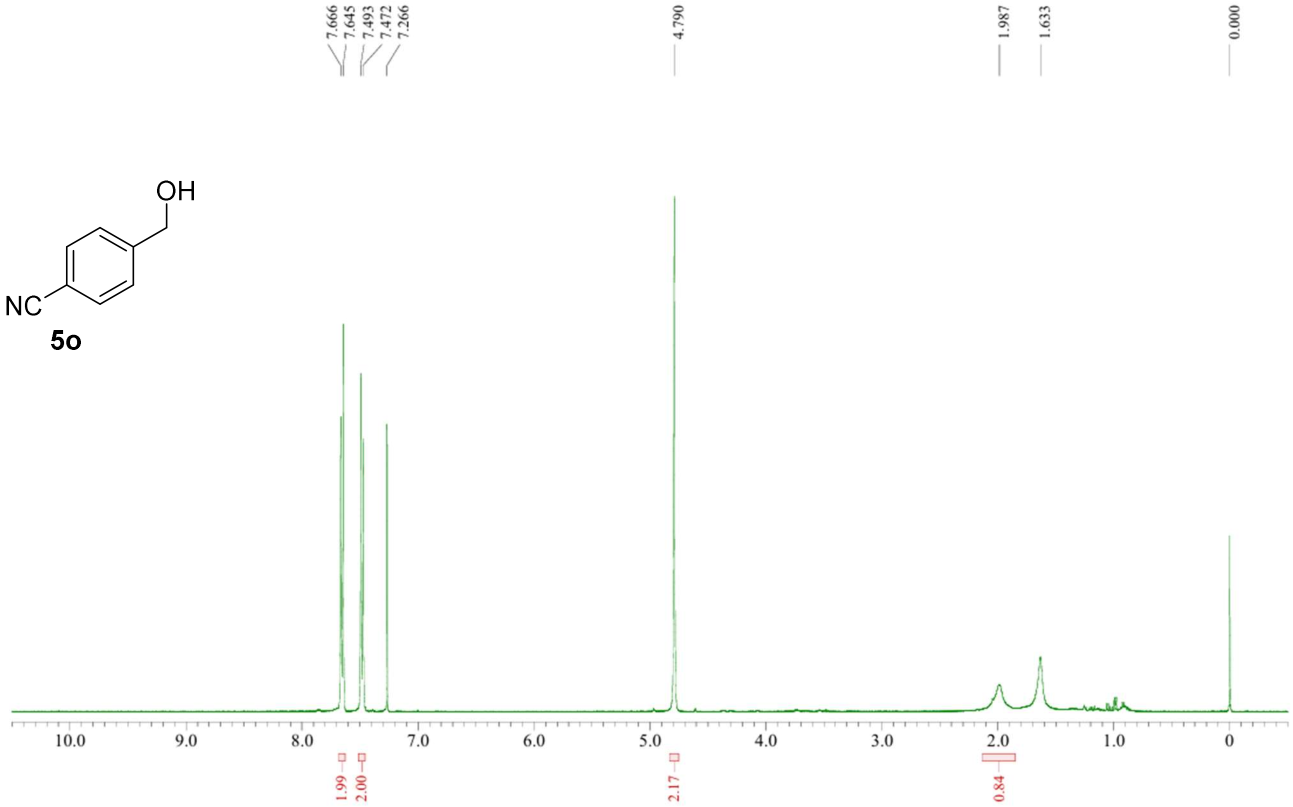

^{13}C{^{1}H} NMR ($CDCl_3$, 100 MHz) spectrum of compound **5o**

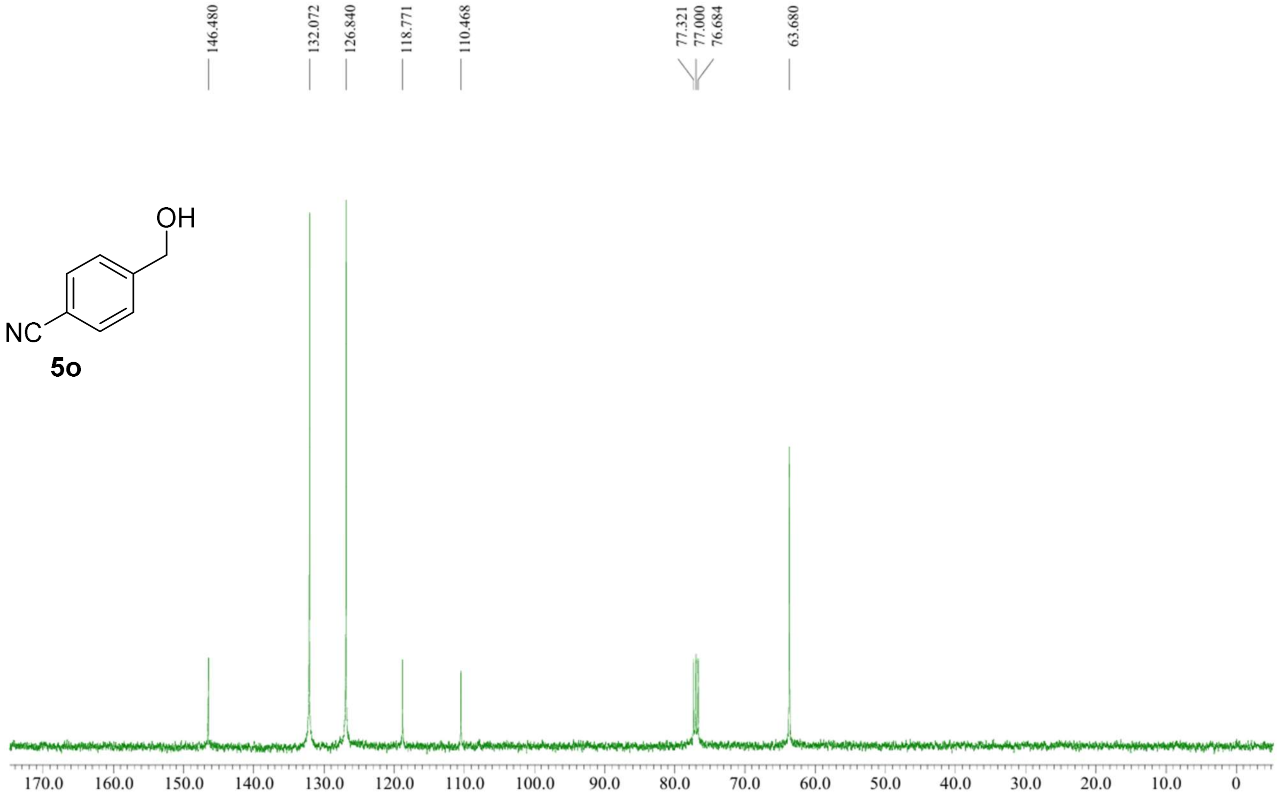

^{1}H NMR ($CDCl_3$, 400 MHz) spectrum of compound **5p**

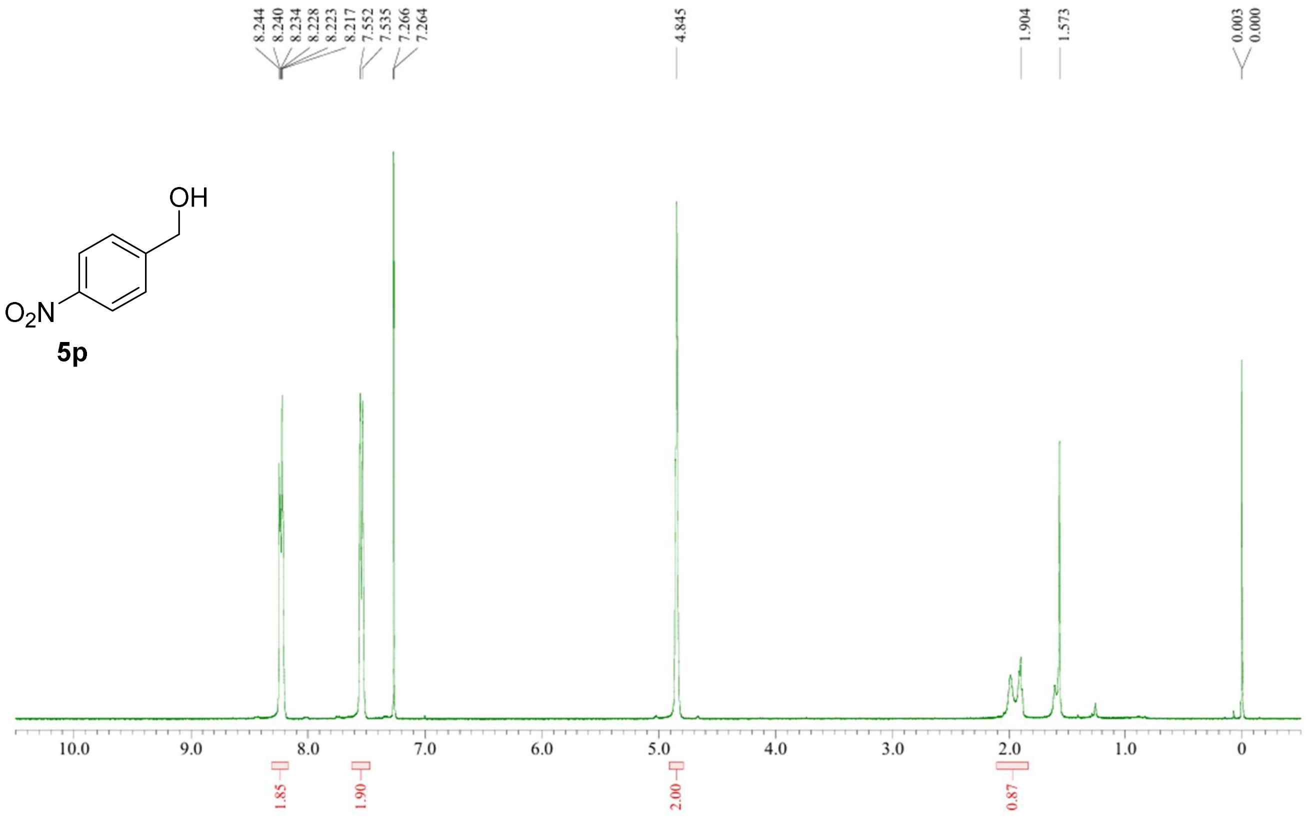

^{13}C{^{1}H} NMR ($CDCl_3$, 100 MHz) spectrum of compound **5p**

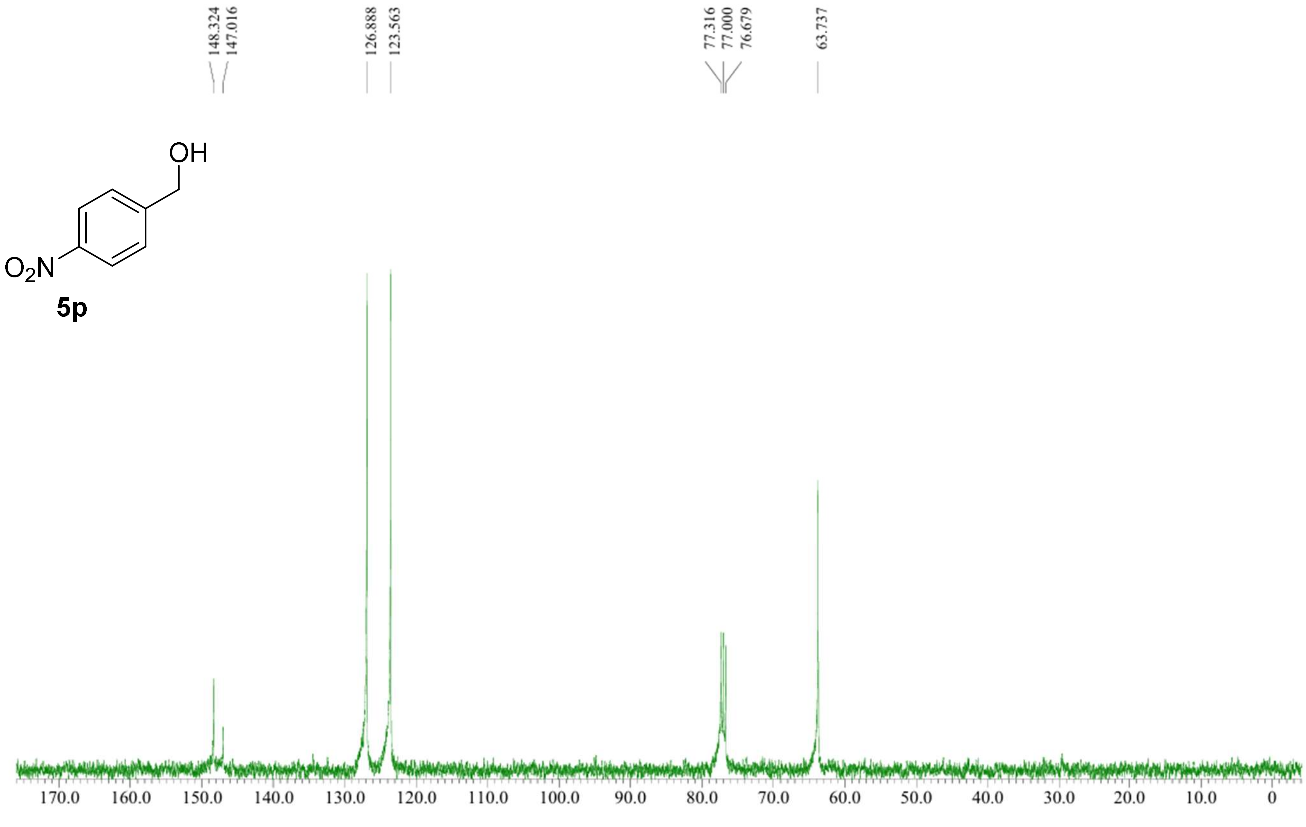

^{1}H NMR ($CDCl_3$, 400 MHz) spectrum of compound **5q**

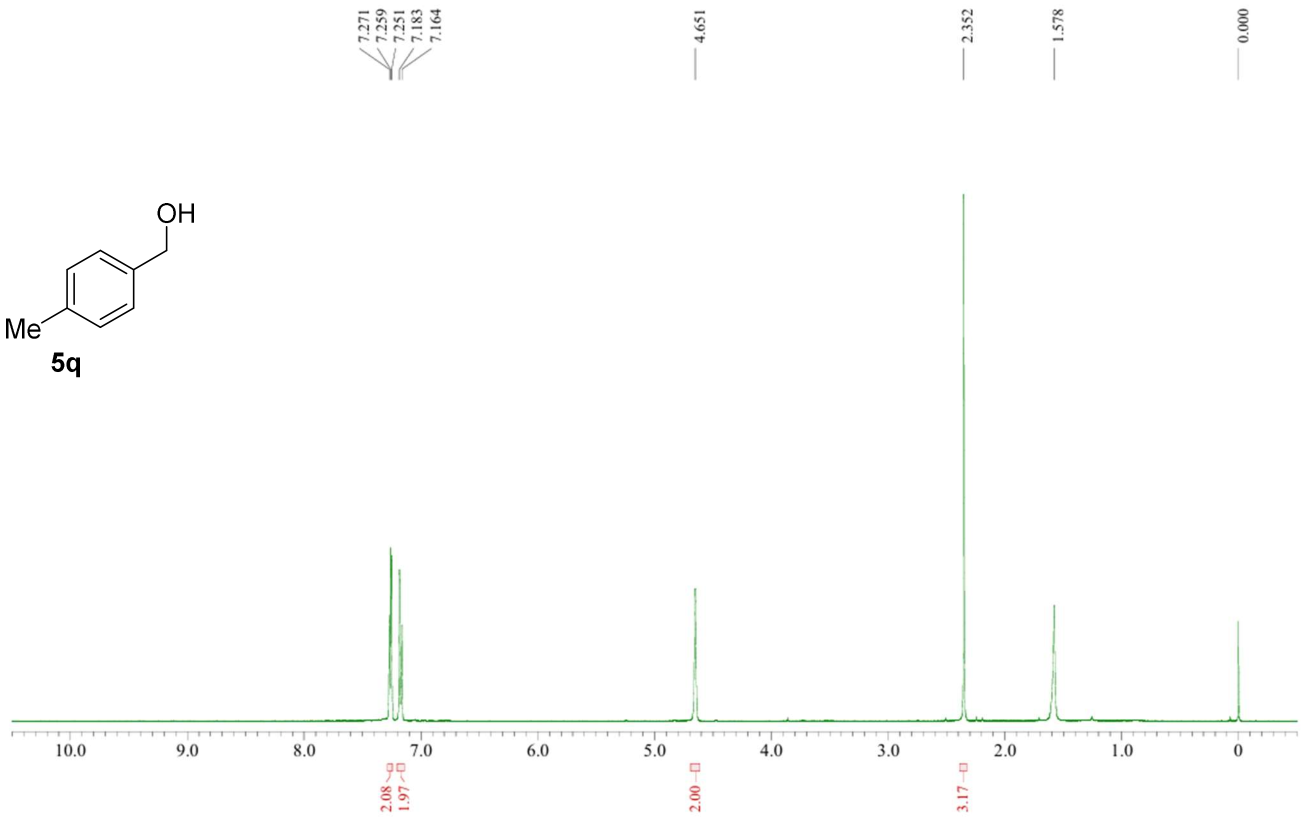

^{13}C{^{1}H} NMR ($CDCl_3$, 100 MHz) spectrum of compound **5q**

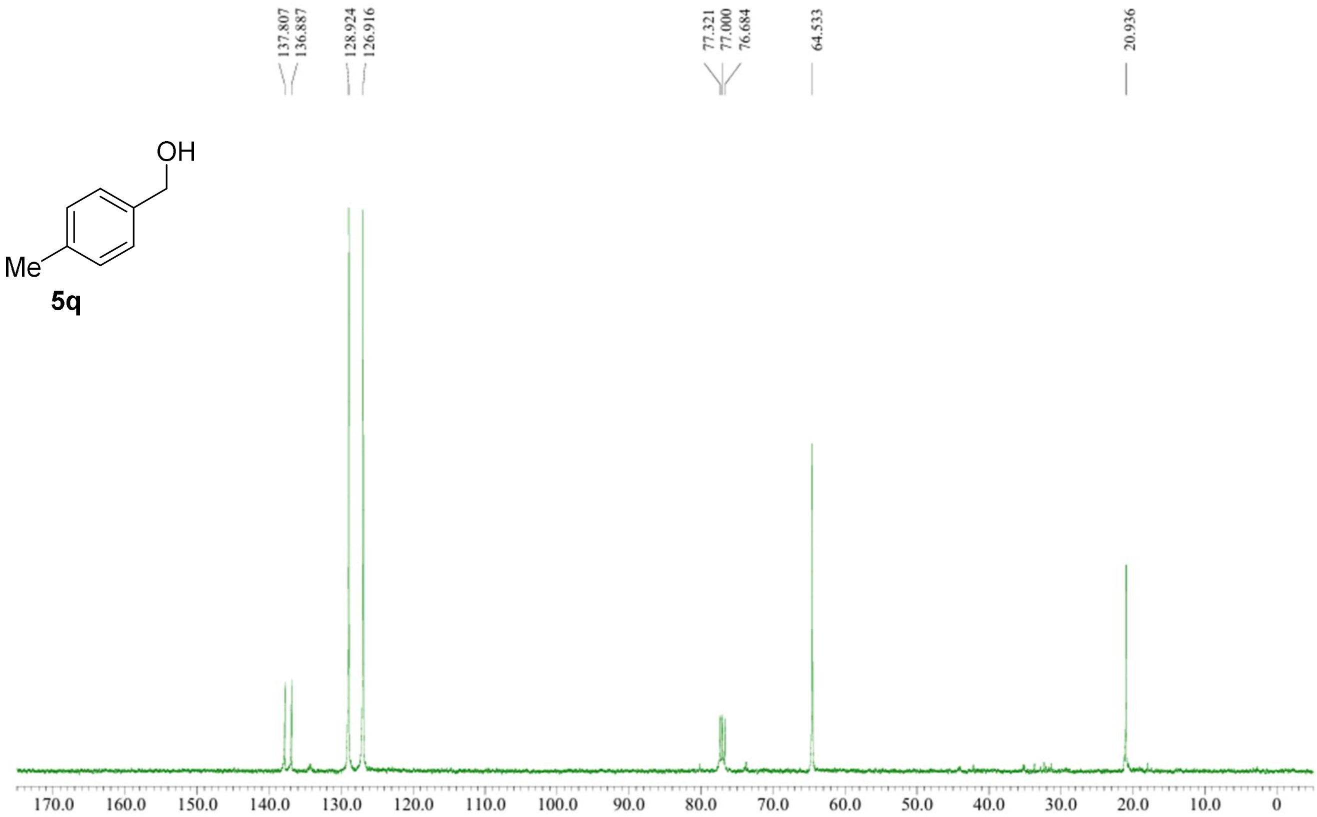

^{1}H NMR ($CDCl_3$, 400 MHz) spectrum of compound **5r**

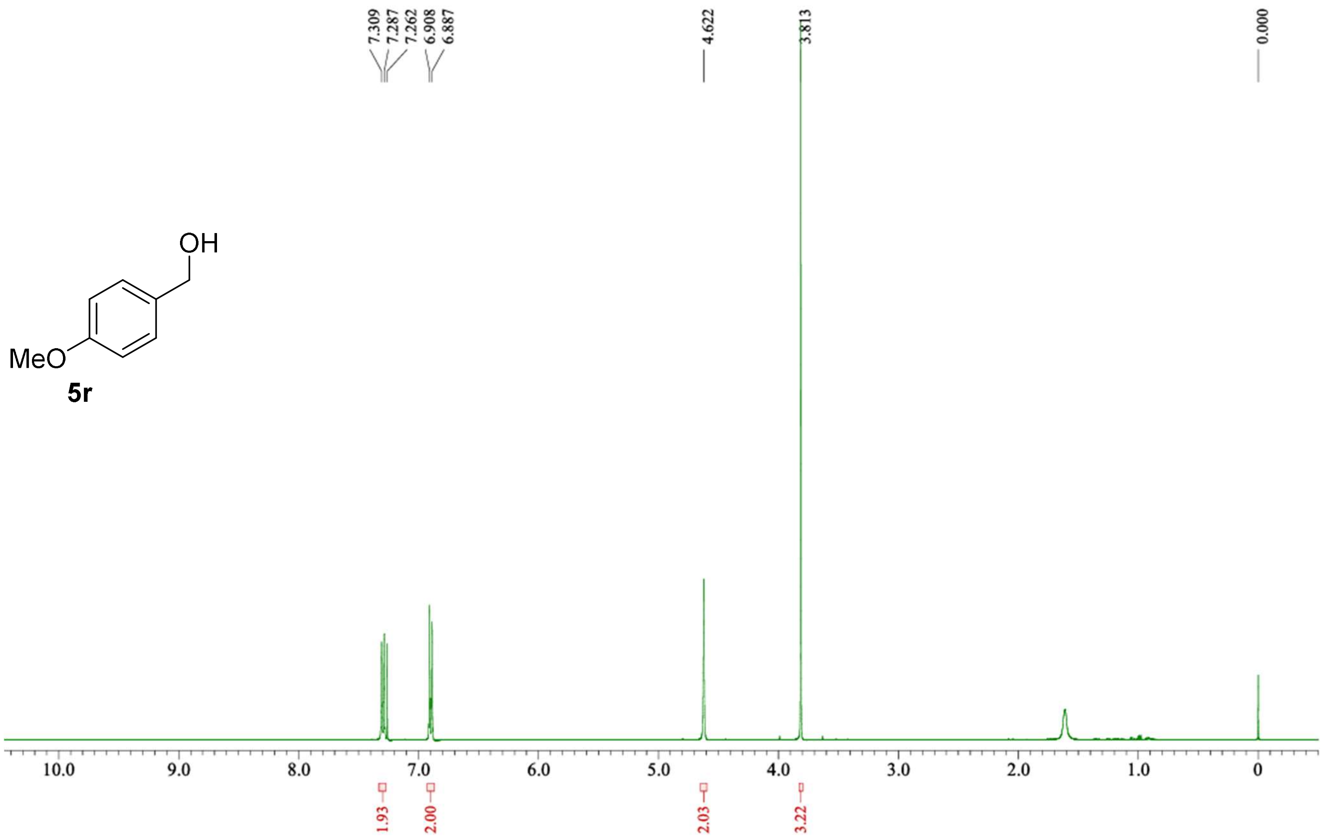

$^{13}C\{^{1}H\}$ NMR ($CDCl_3$, 100 MHz) spectrum of compound **5r**

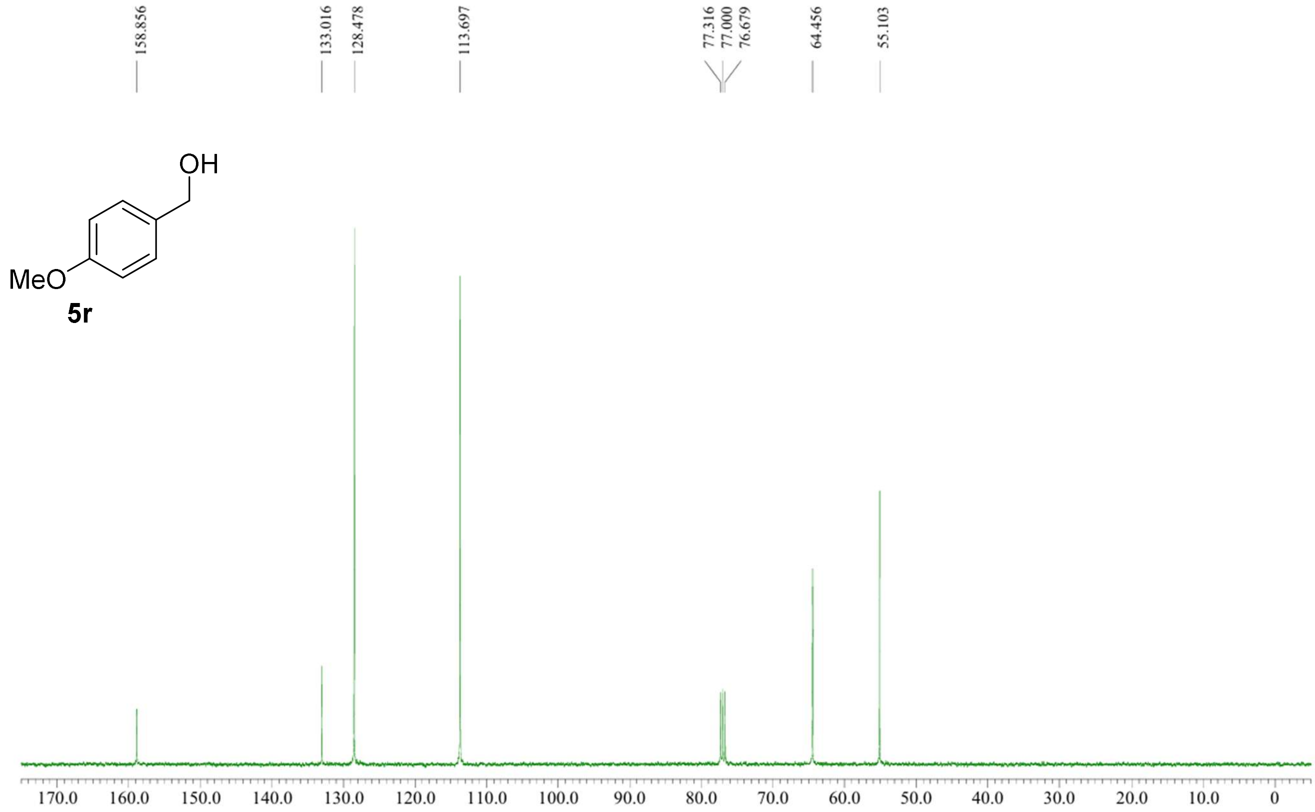

^{1}H NMR ($CDCl_3$, 400 MHz) spectrum of compound **5s**

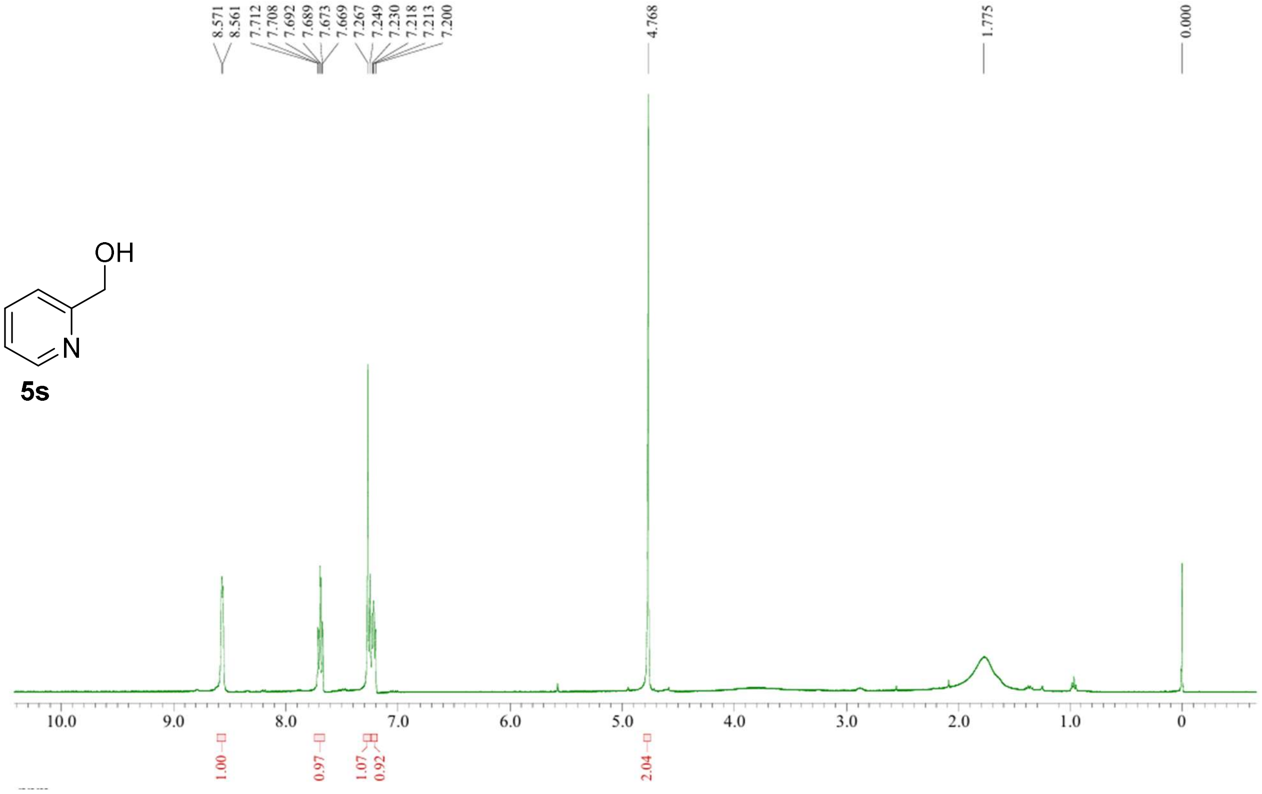

$^{13}C\{^{1}H\}$ NMR ($CDCl_3$, 100 MHz) spectrum of compound **5s**

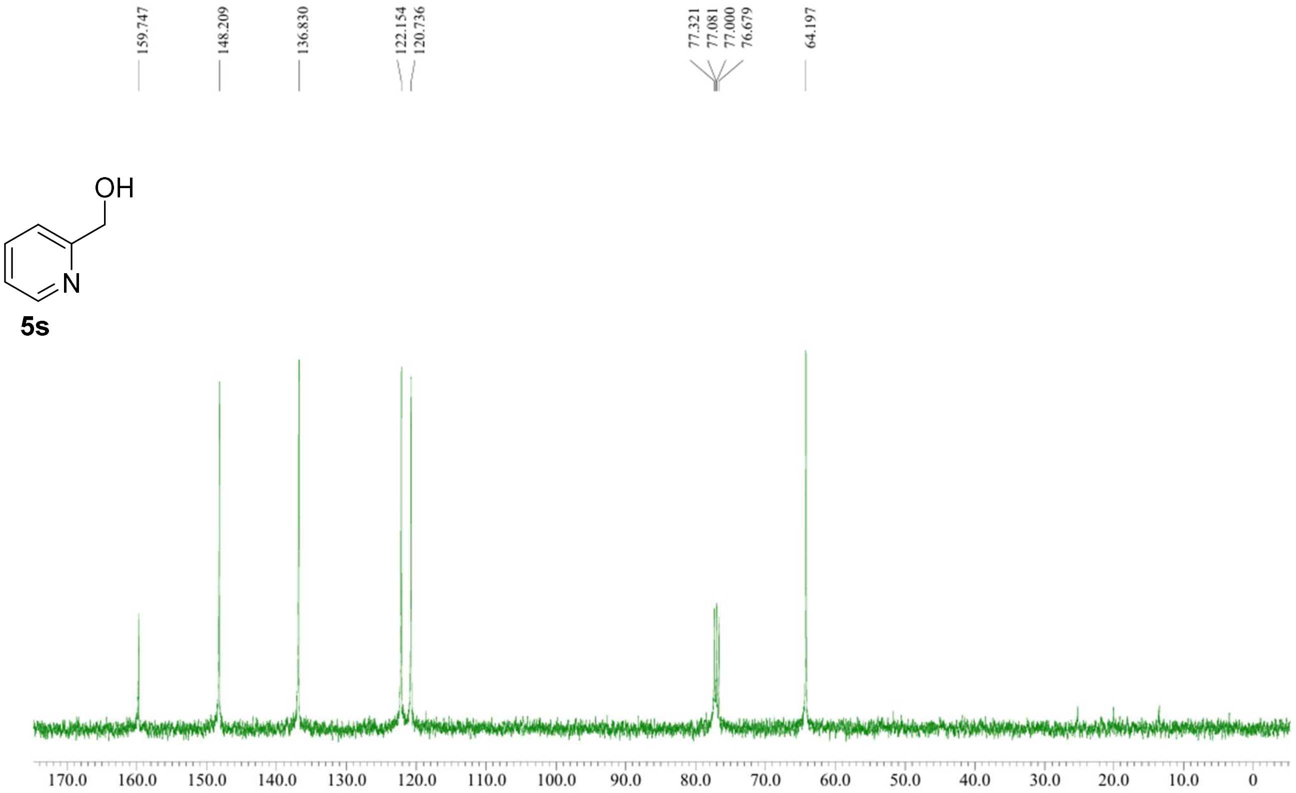

^{1}H NMR ($CDCl_3$, 400 MHz) spectrum of compound **5t**

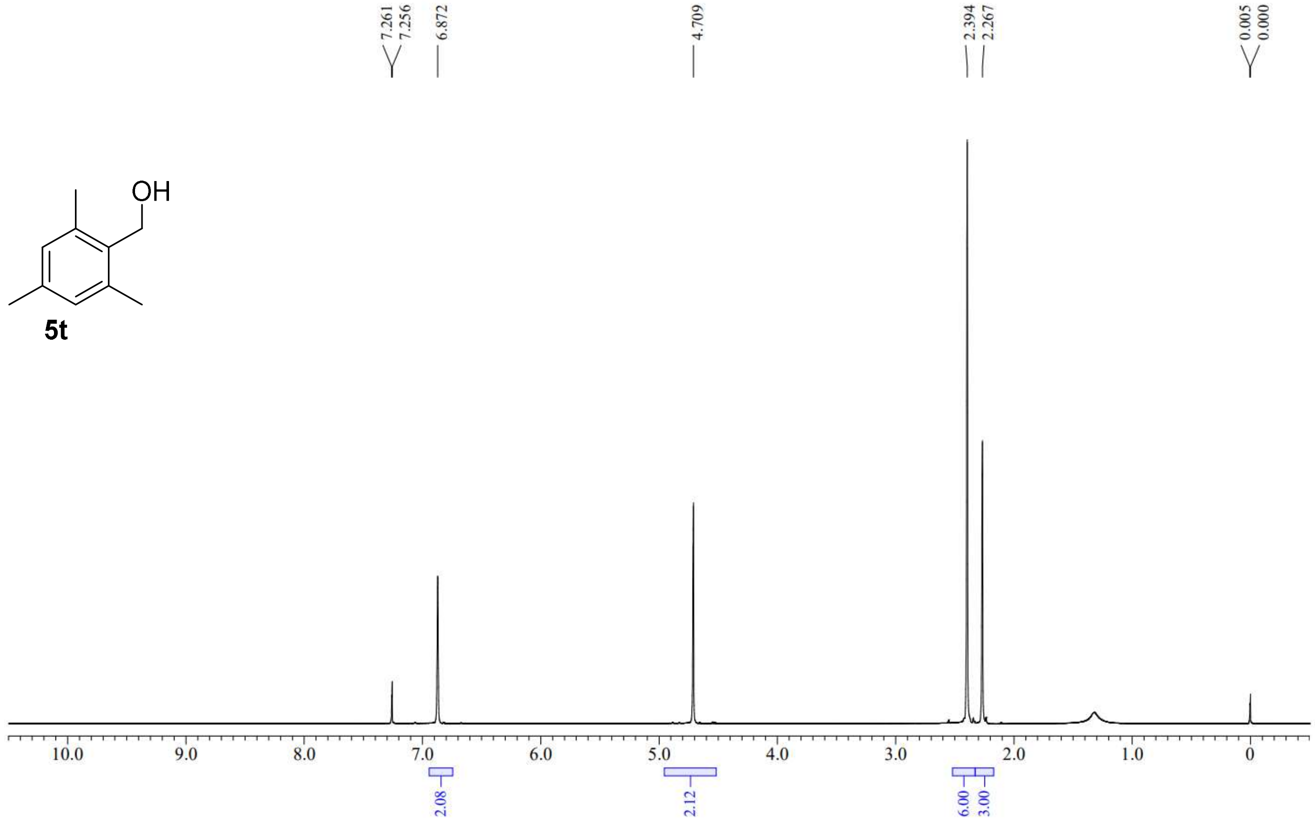

$^{13}C\{^{1}H\}$ NMR ($CDCl_3$, 100 MHz) spectrum of compound **5t**

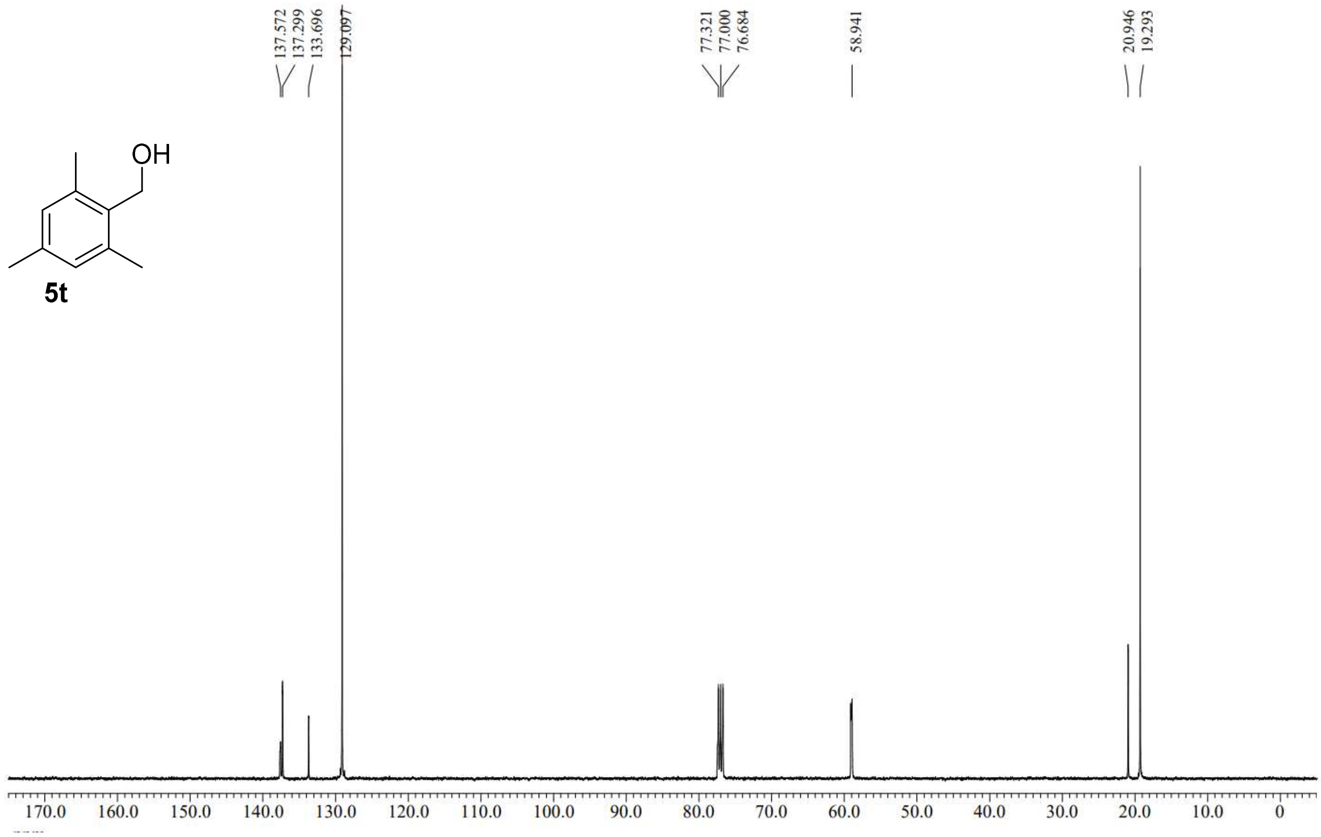

^{1}H NMR ($CDCl_3$, 400 MHz) spectrum of compound **7a**

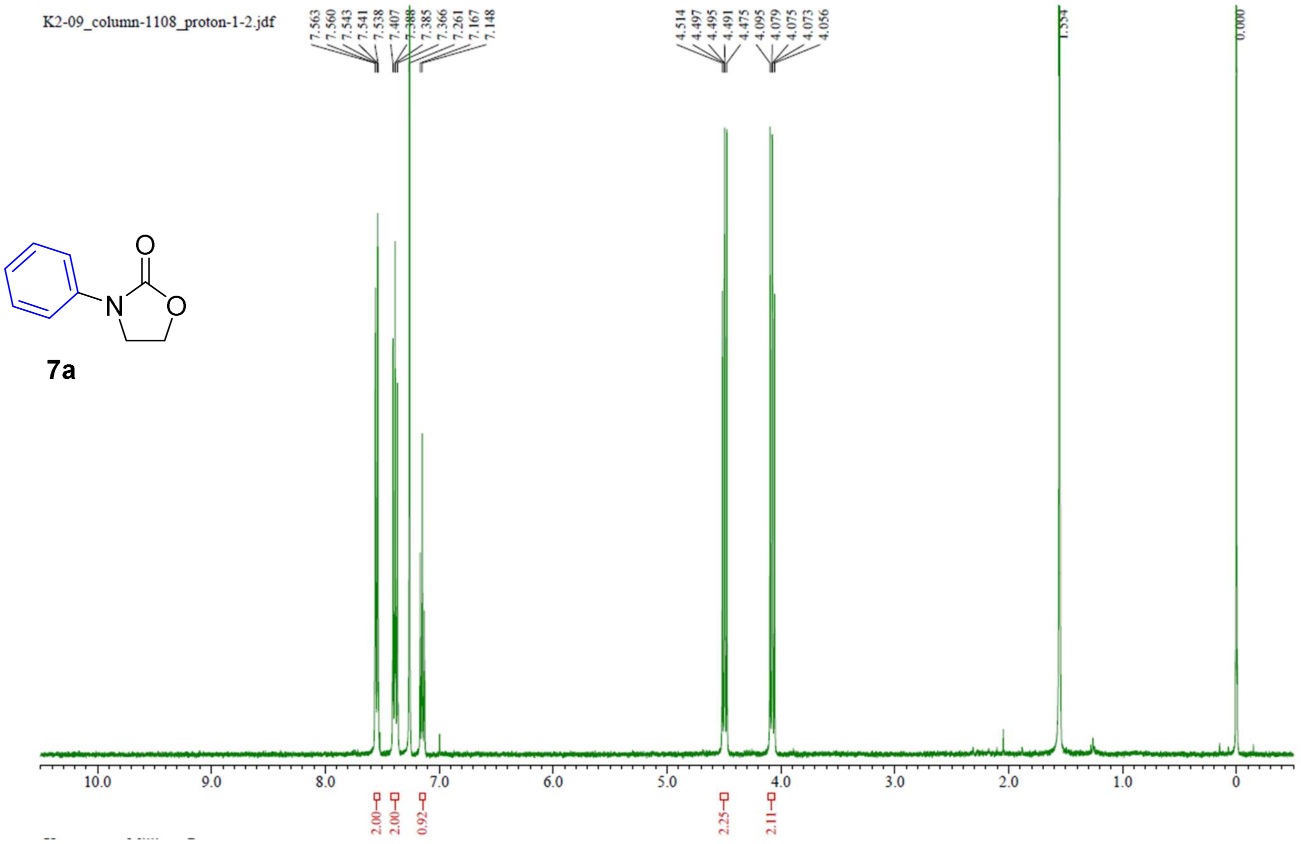

$^{13}C\{^{1}H\}$ NMR ($CDCl_3$, 100 MHz) spectrum of compound **7a**

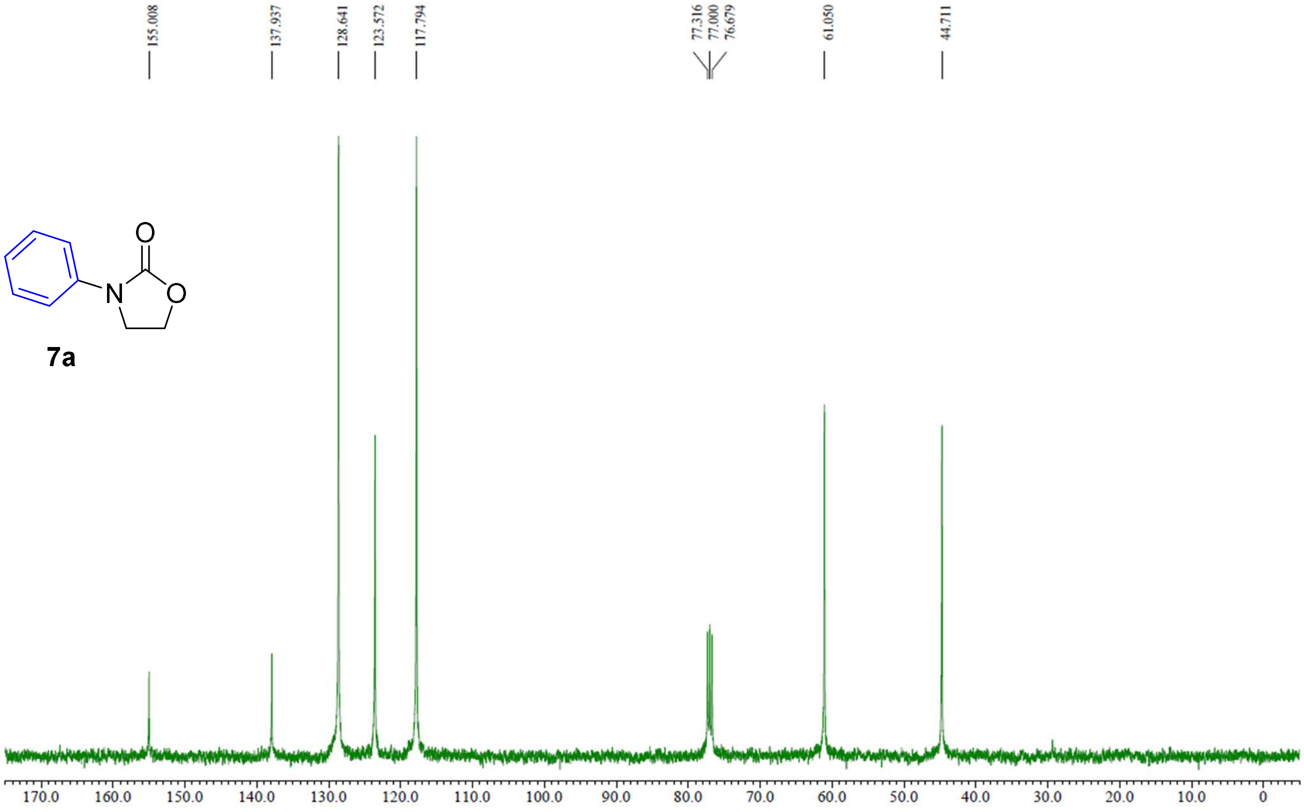

^{1}H NMR ($CDCl_3$, 400 MHz) spectrum of compound **7b**

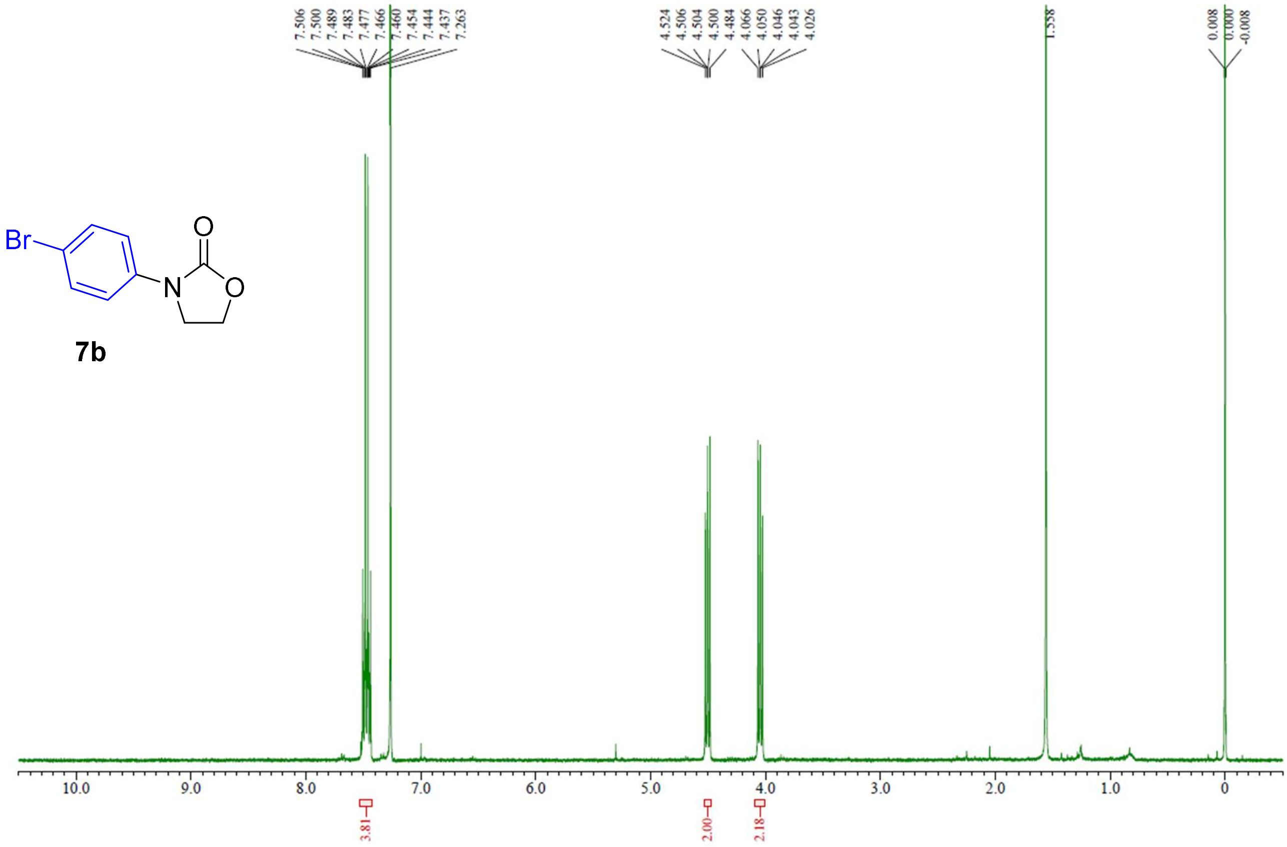

^{13}C{^{1}H} NMR ($CDCl_3$, 100 MHz) spectrum of compound **7b**

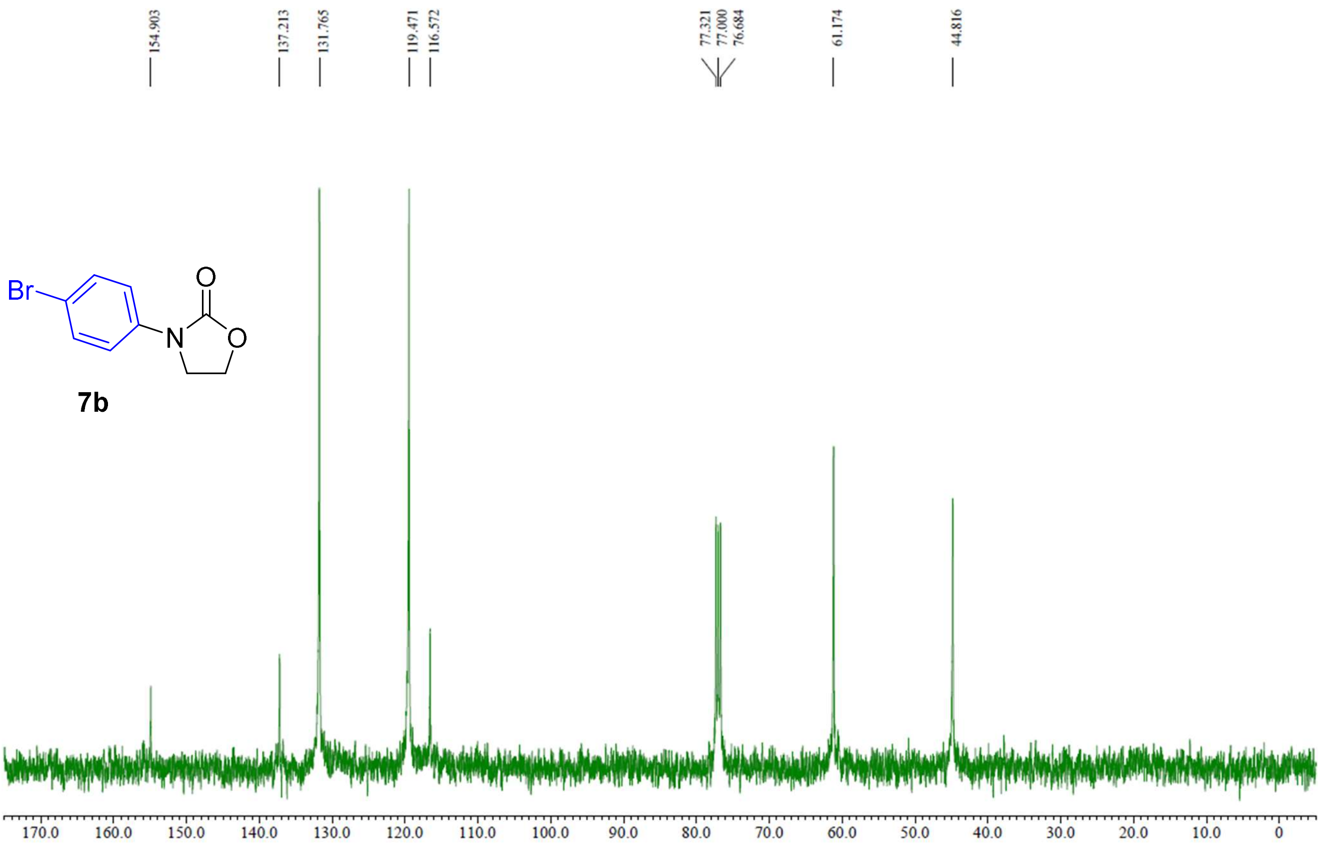

^{1}H NMR ($CDCl_3$, 400 MHz) spectrum of compound **7c**

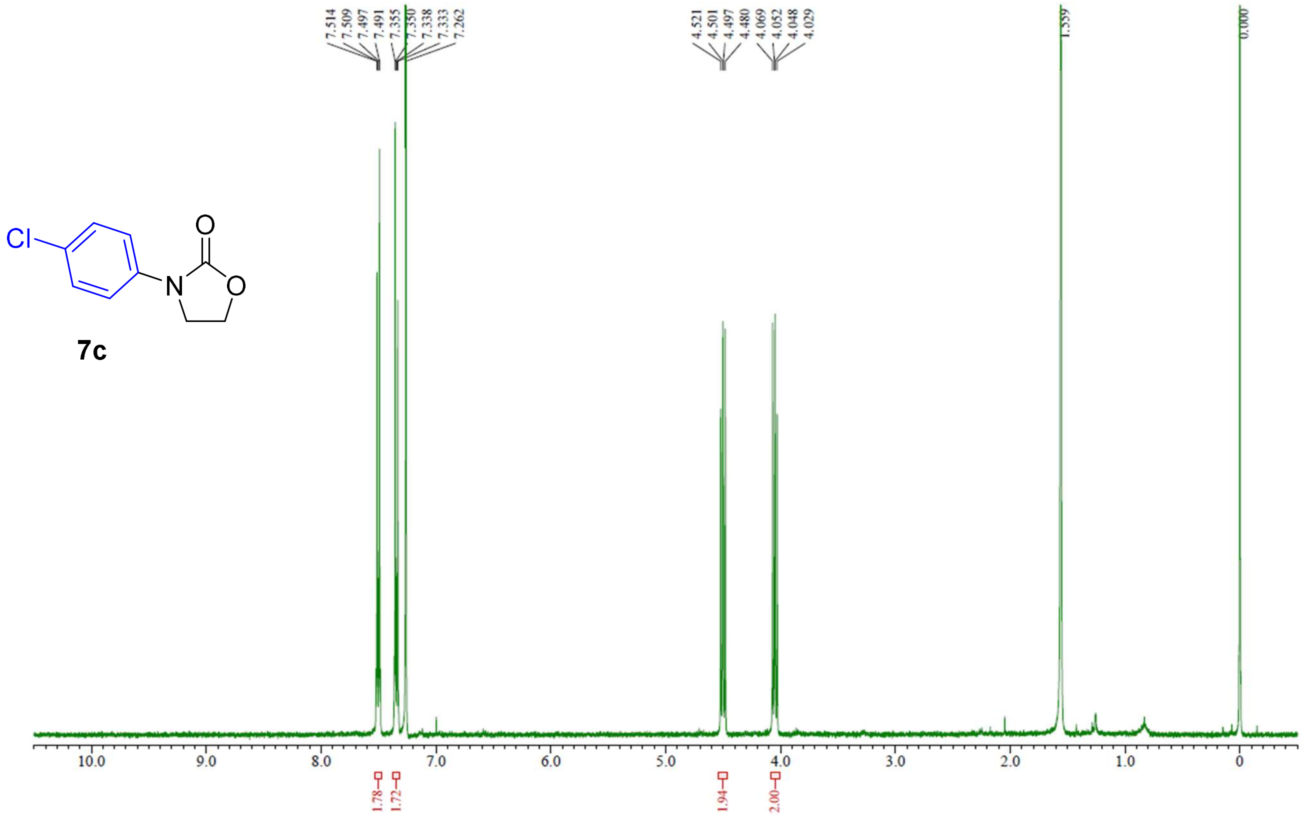

^{13}C{^{1}H} NMR ($CDCl_3$, 100 MHz) spectrum of compound **7c**

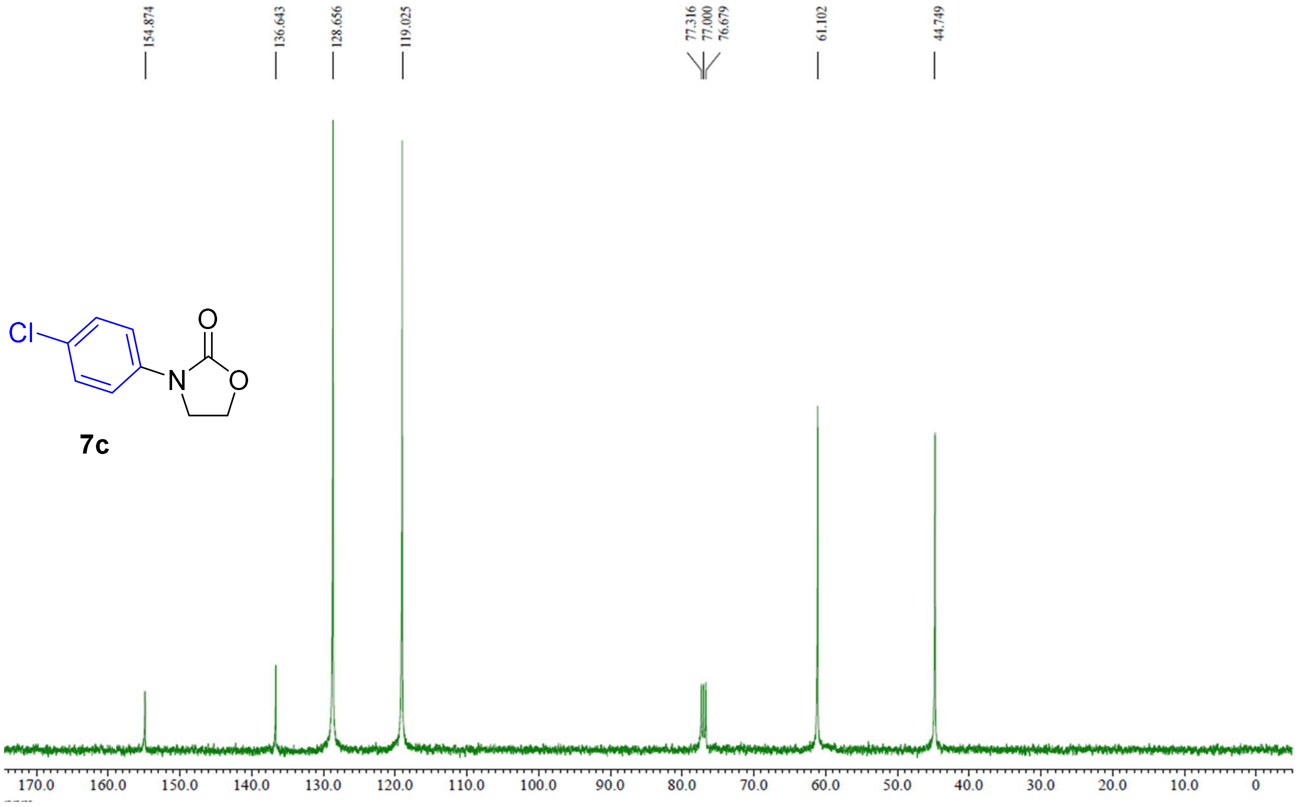

^{1}H NMR ($CDCl_3$, 400 MHz) spectrum of compound **7d**

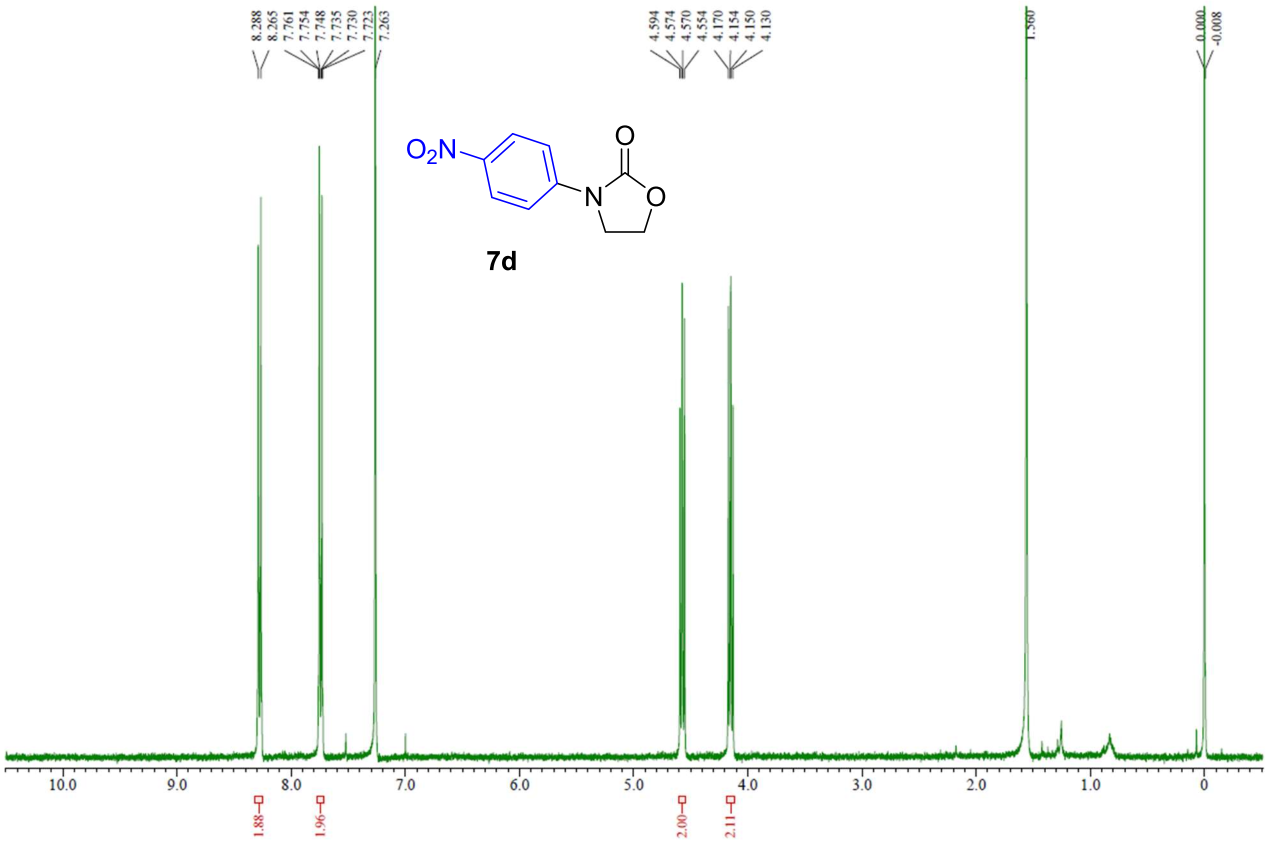

$^{13}C\{^{1}H\}$ NMR ($CDCl_3$, 100 MHz) spectrum of compound **7d**

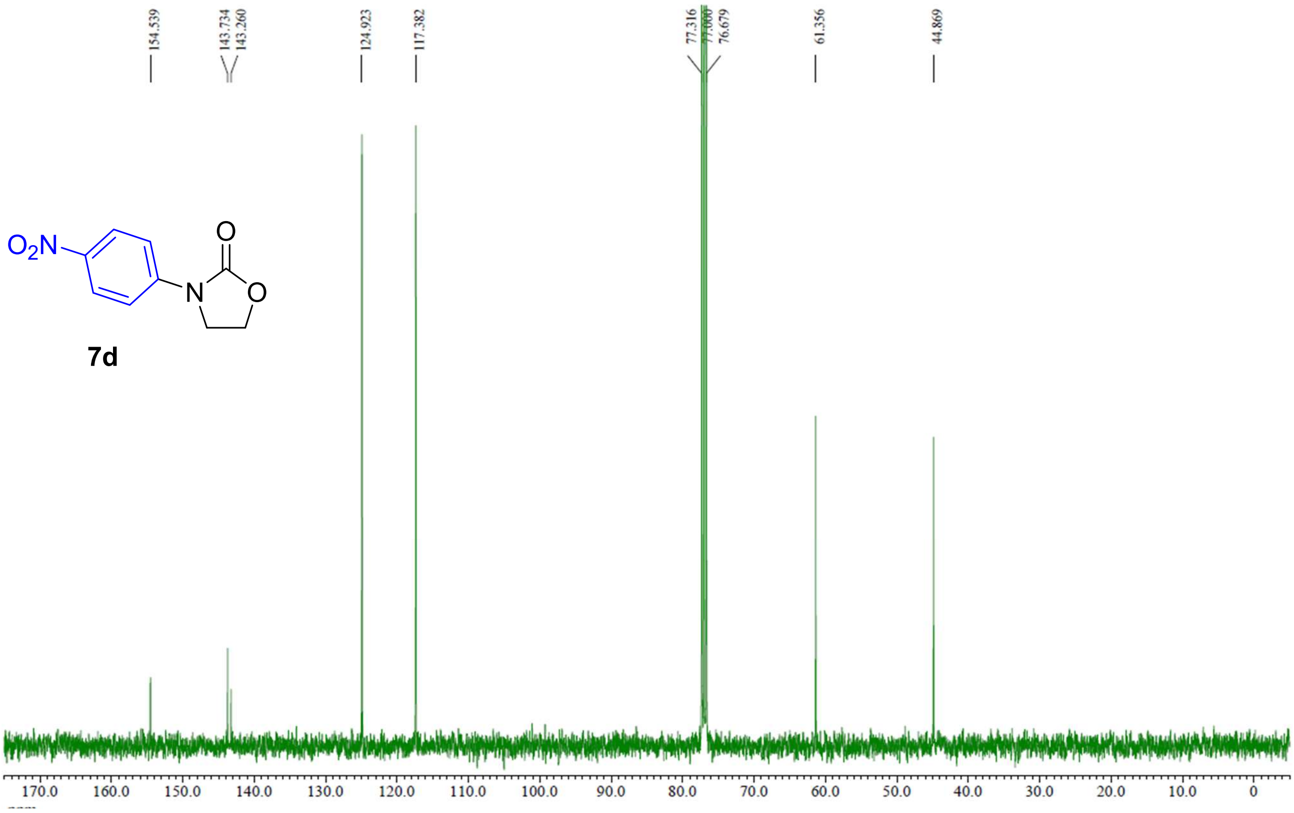

^{1}H NMR ($CDCl_3$, 400 MHz) spectrum of compound **7e**

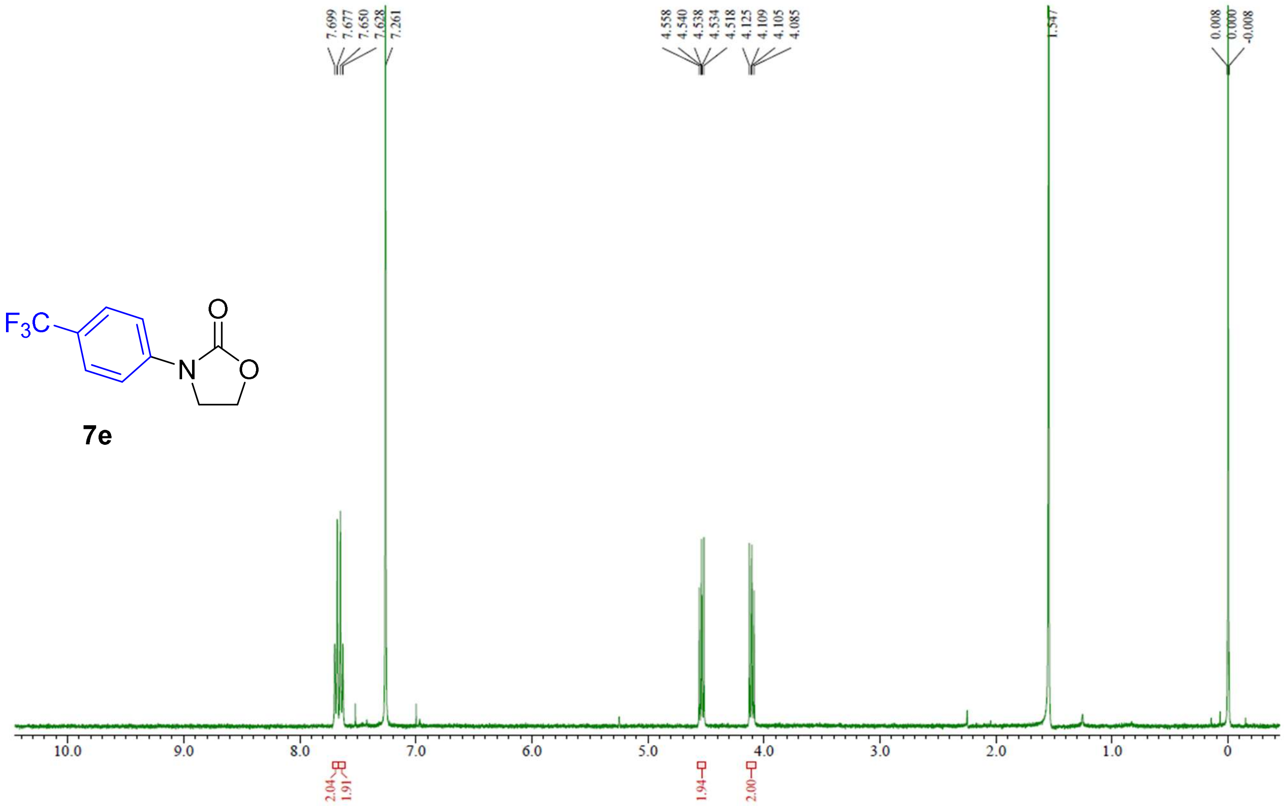

^{13}C{^{1}H} NMR ($CDCl_3$, 100 MHz) spectrum of compound **7e**

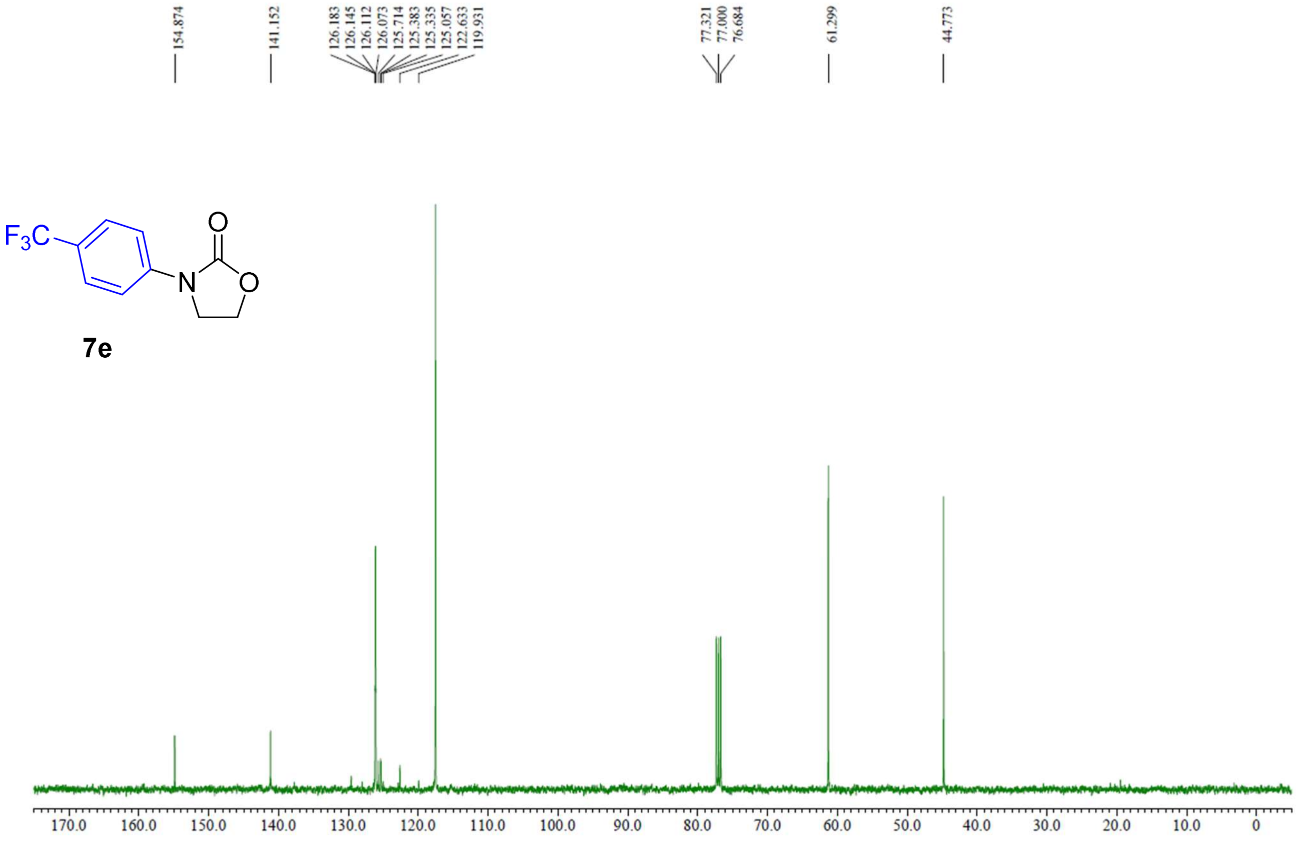

^{19}F NMR ($CDCl_3$, 376 MHz) spectrum of compound **7e**

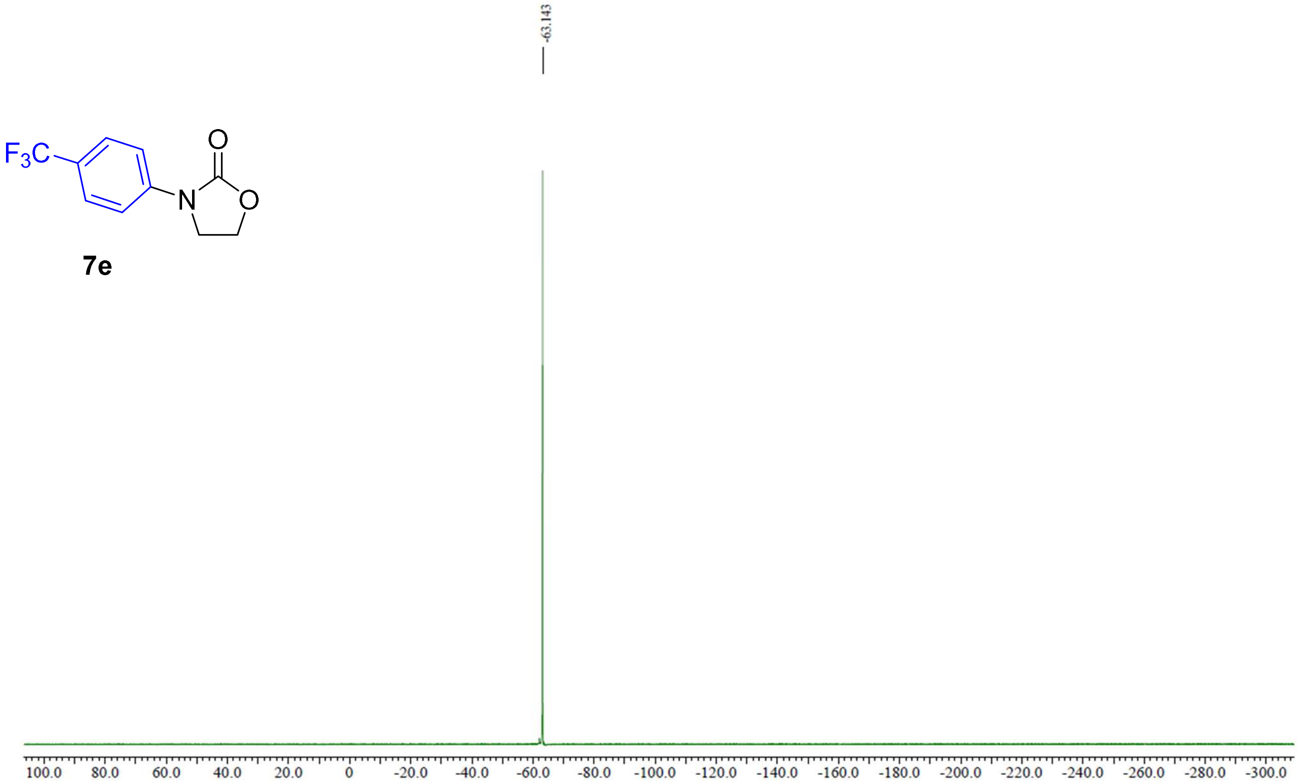

7e

^{1}H NMR ($CDCl_3$, 400 MHz) spectrum of compound **7f**

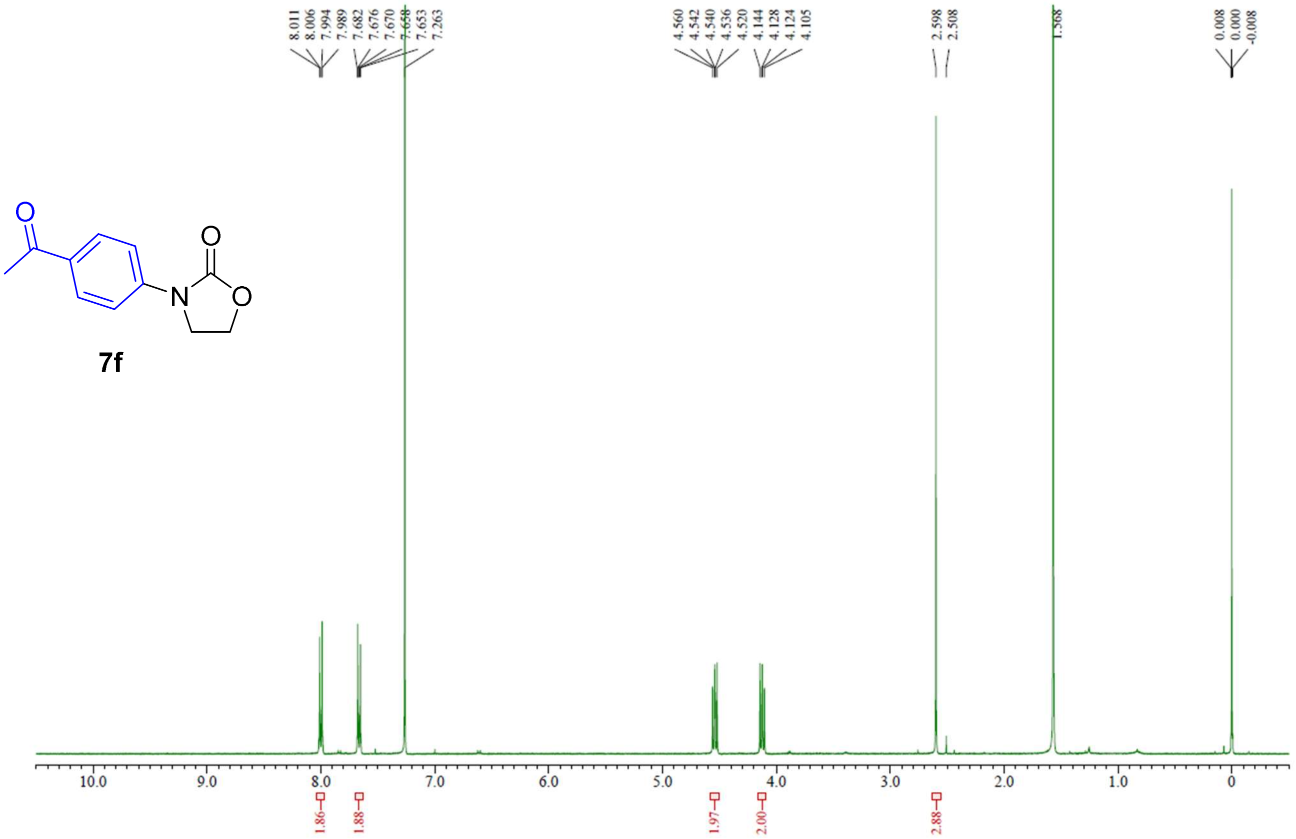

7f

$^{13}C\{^{1}H\}$ NMR ($CDCl_3$, 100 MHz) spectrum of compound **7f**

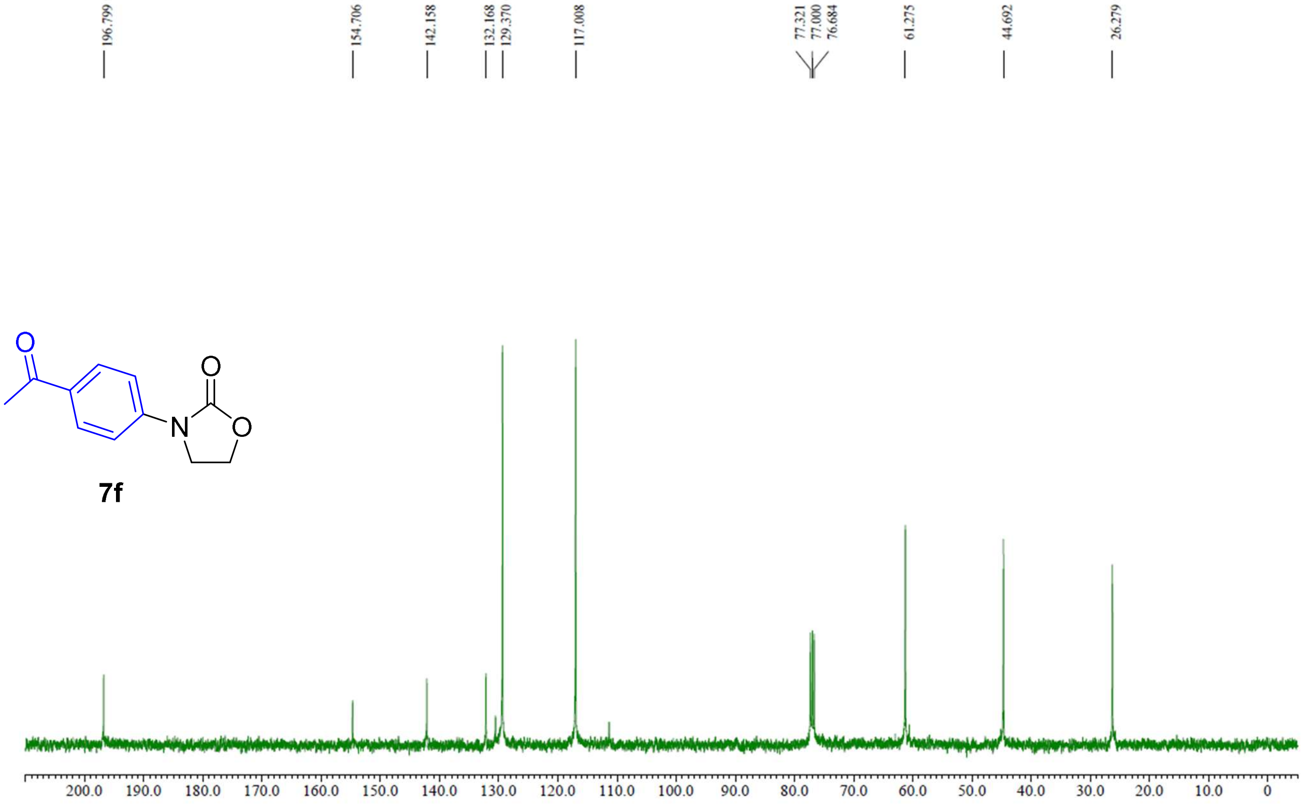

7f

^{1}H NMR ($CDCl_3$, 400 MHz) spectrum of compound **7g**

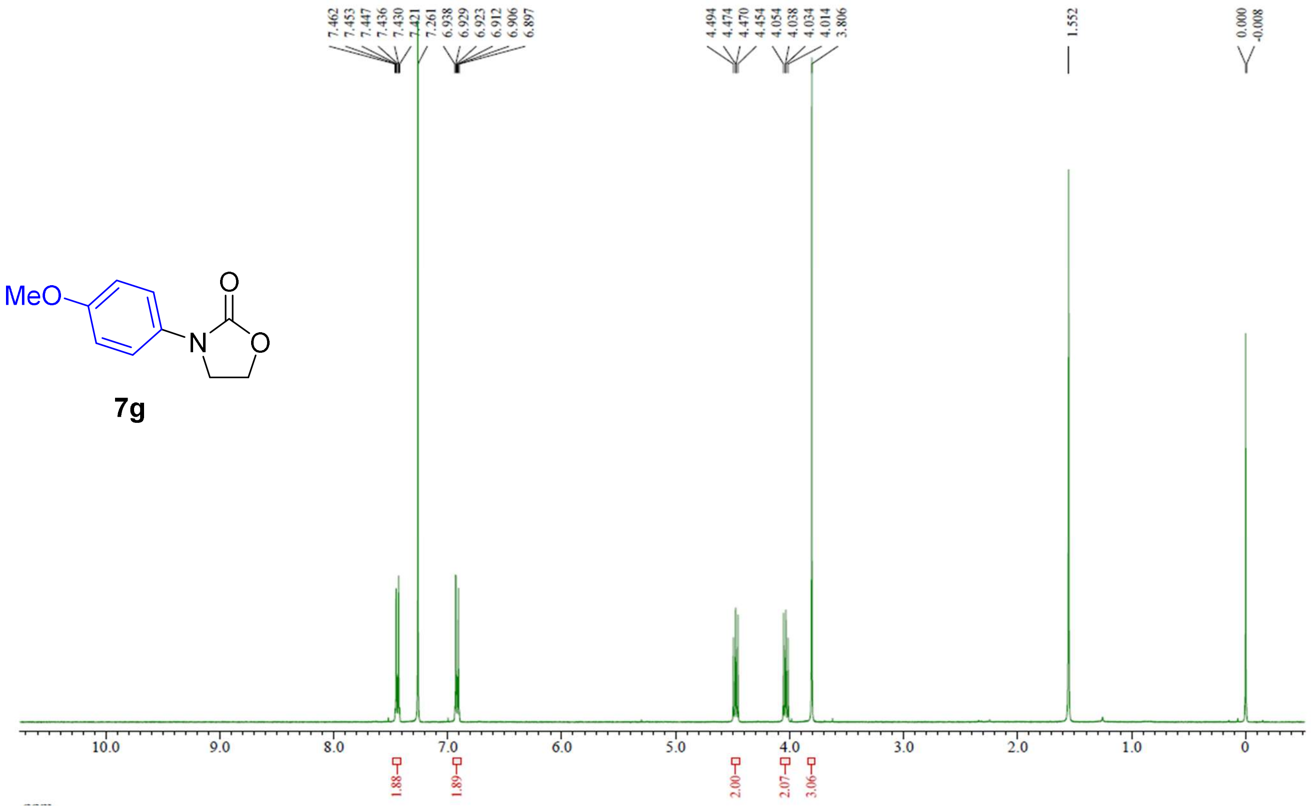

$^{13}C\{^{1}H\}$ NMR ($CDCl_3$, 100 MHz) spectrum of compound **7g**

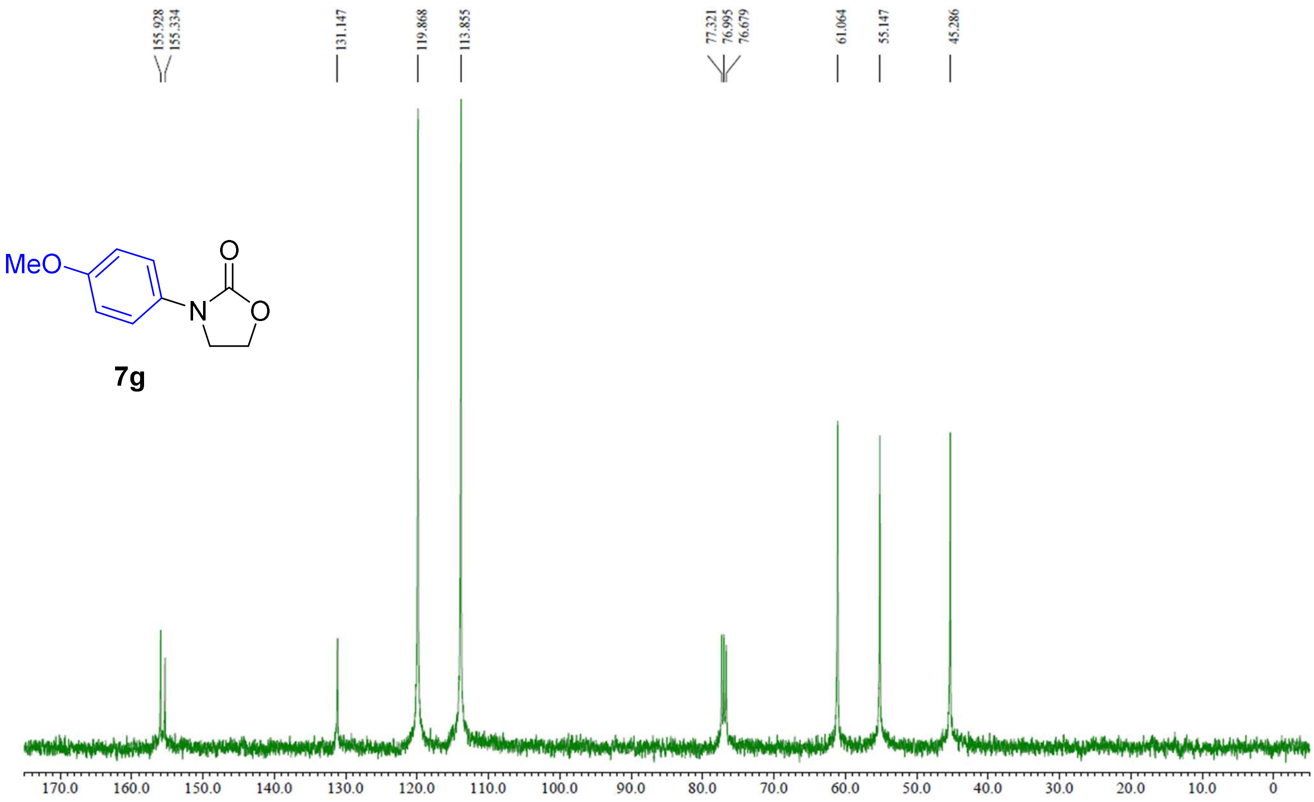

^{1}H NMR ($CDCl_3$, 400 MHz) spectrum of compound **7h**

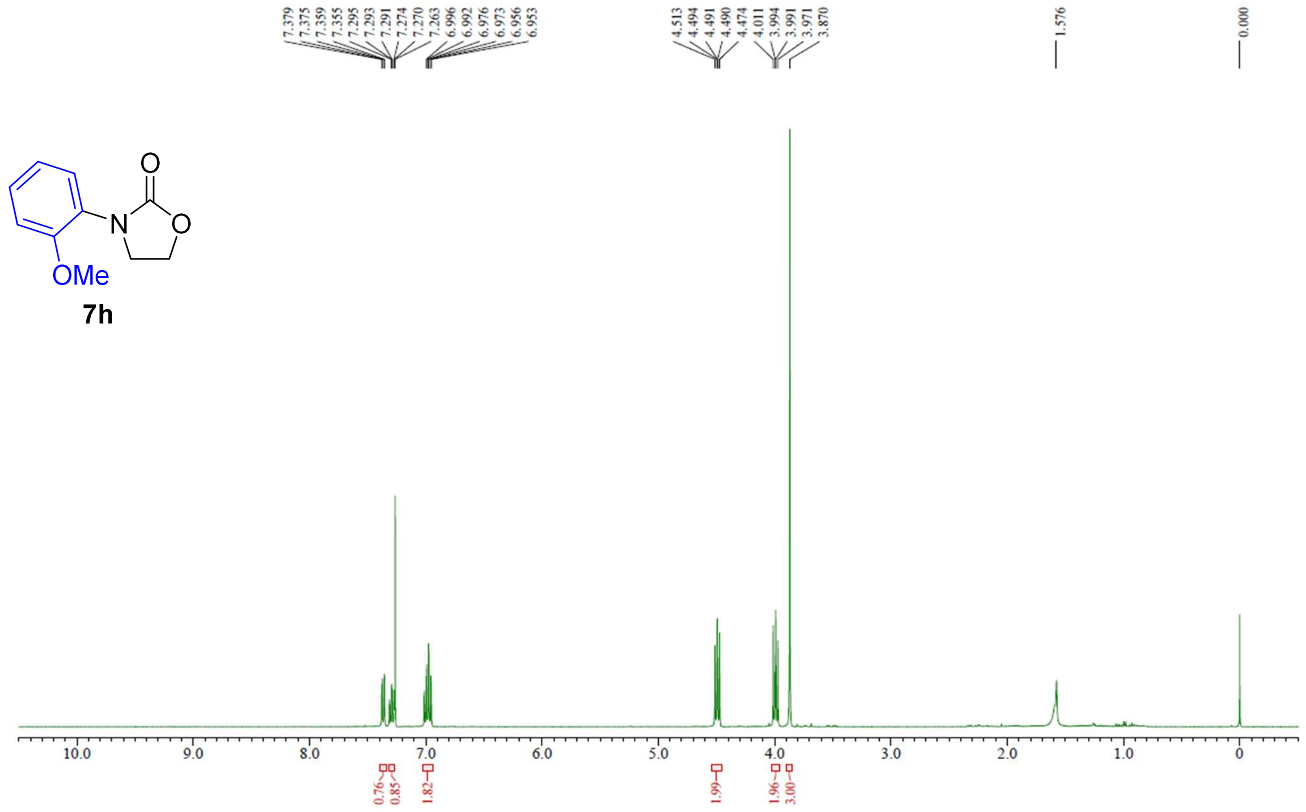

7h

^{13}C{^{1}H} NMR ($CDCl_3$, 100 MHz) spectrum of compound **7h**

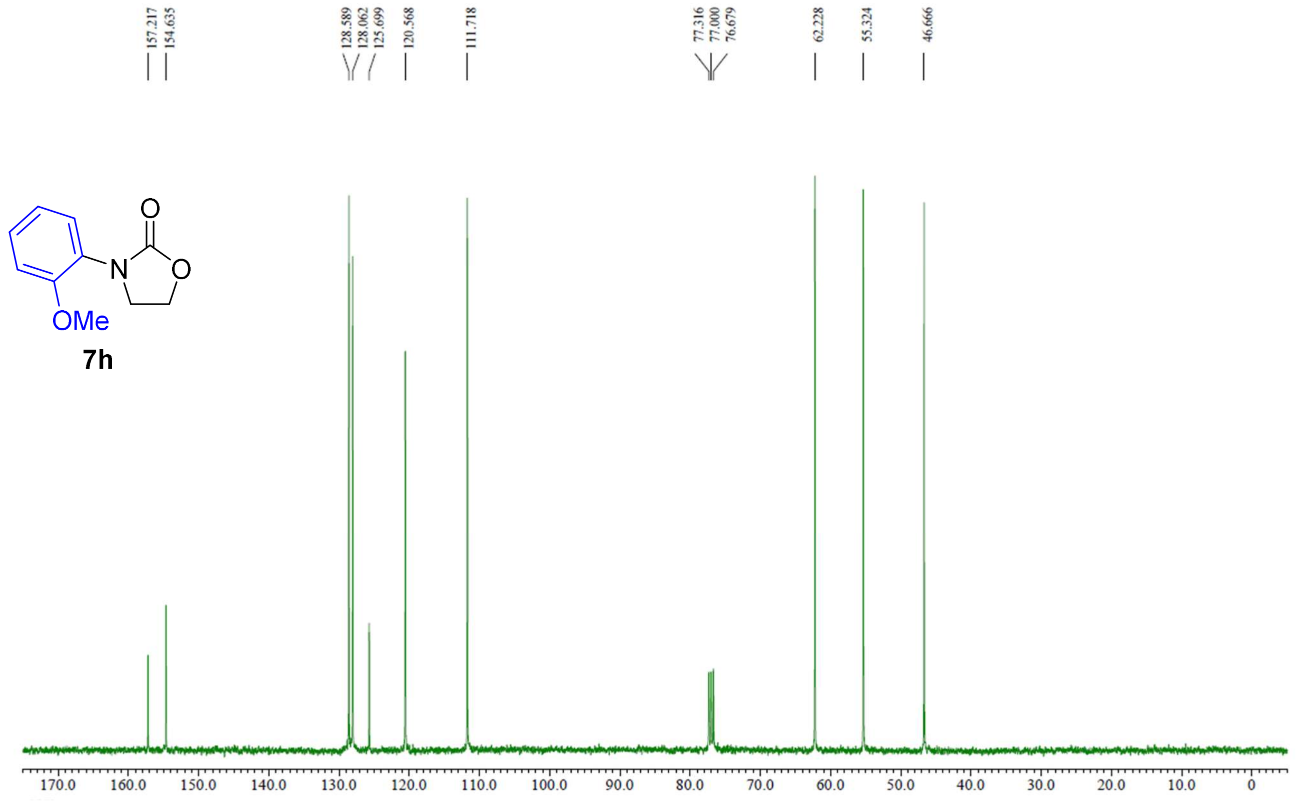

7h

^{1}H NMR ($CDCl_3$, 400 MHz) spectrum of compound **7i**

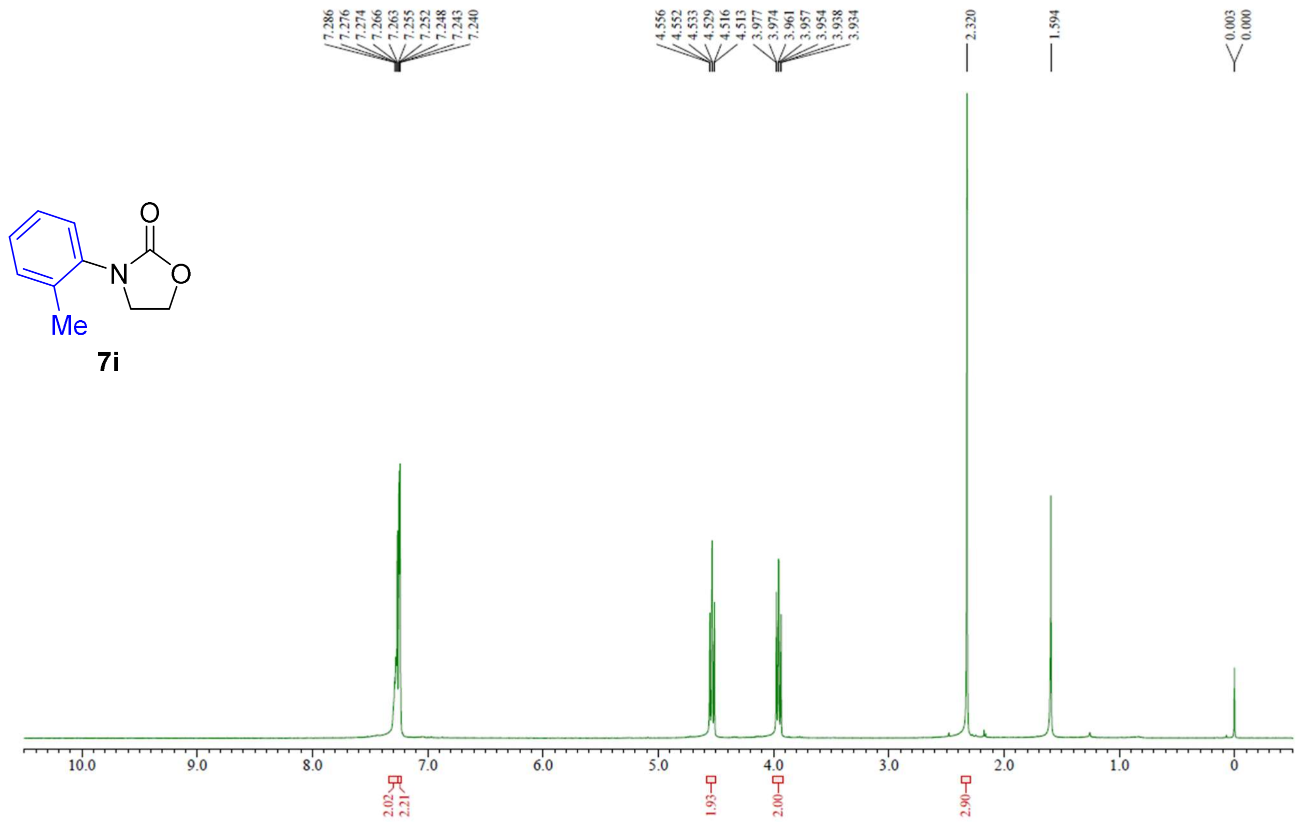

^{13}C{^{1}H} NMR ($CDCl_3$, 100 MHz) spectrum of compound **7i**

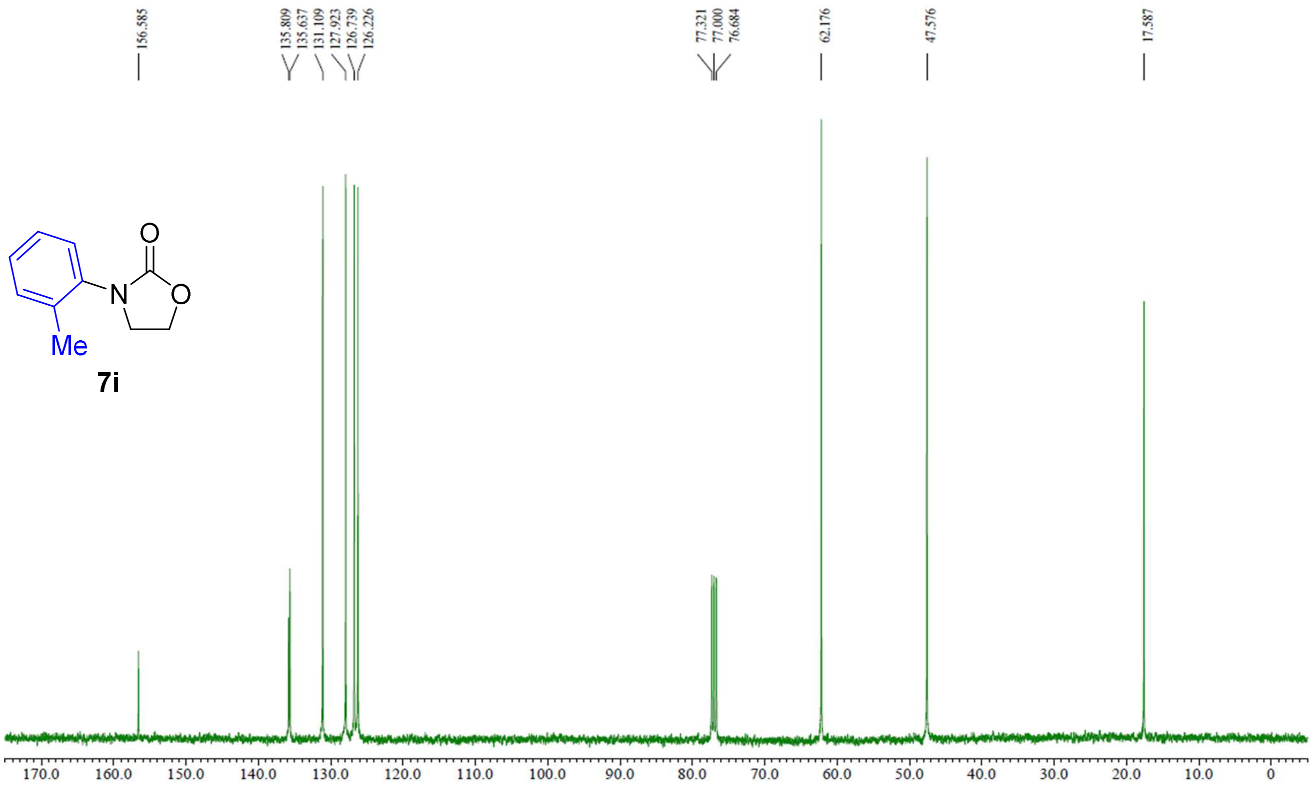

^{1}H NMR ($CDCl_3$, 400 MHz) spectrum of compound **7j**

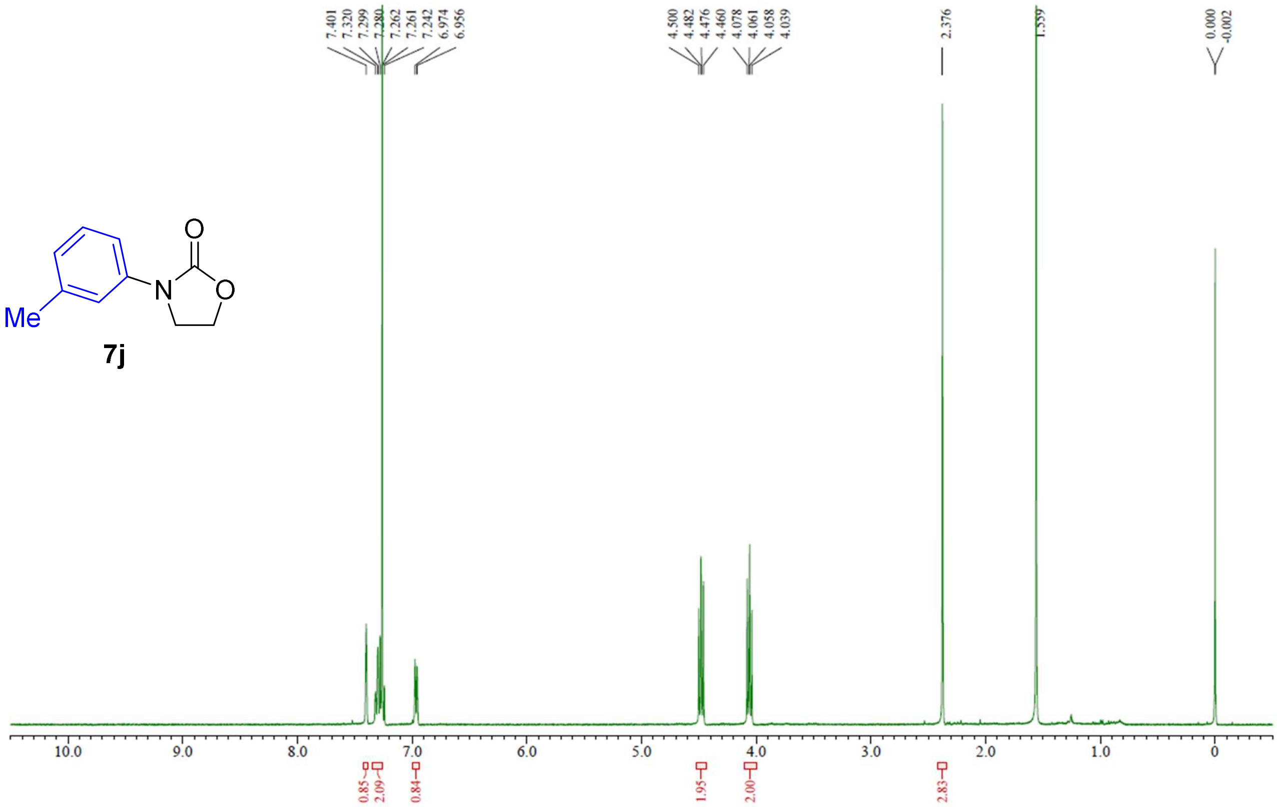

$^{13}C\{^{1}H\}$ NMR ($CDCl_3$, 100 MHz) spectrum of compound **7j**

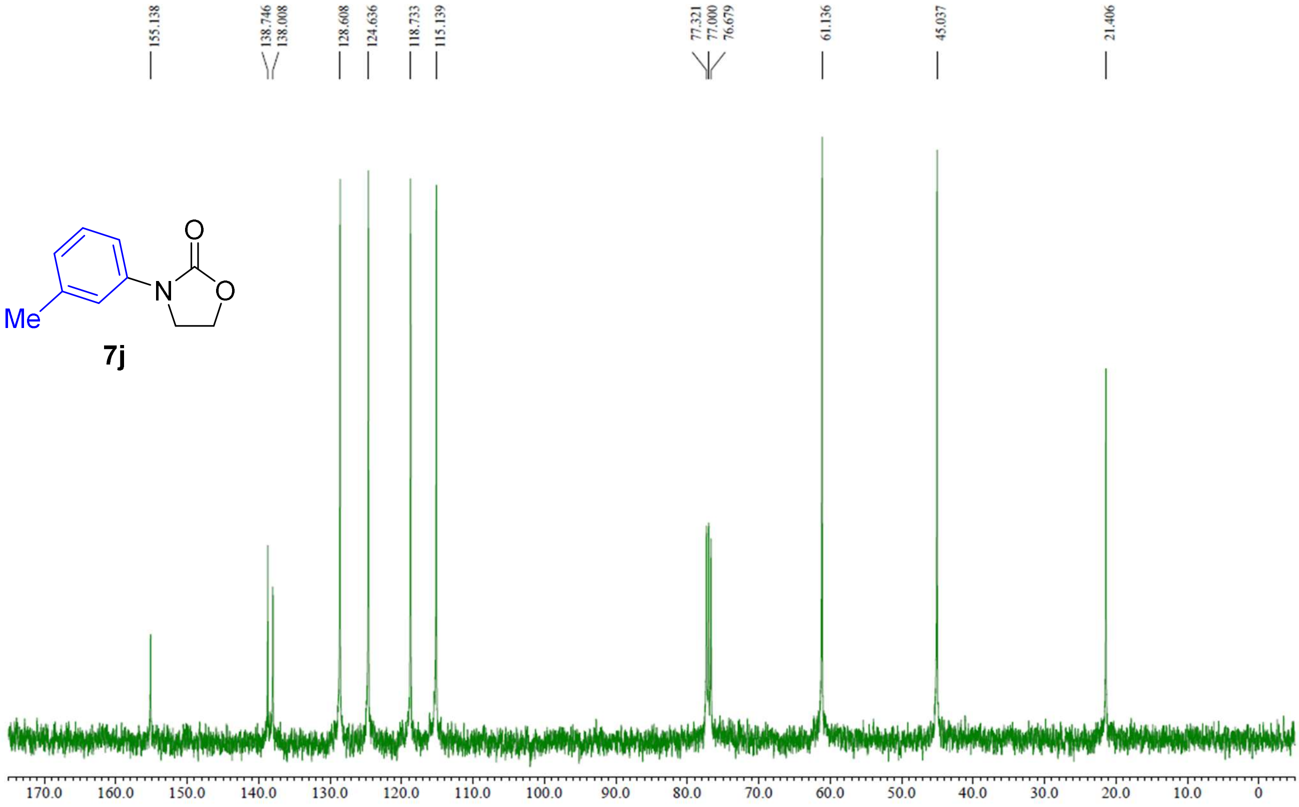

^{1}H NMR ($CDCl_3$, 400 MHz) spectrum of compound **7k**

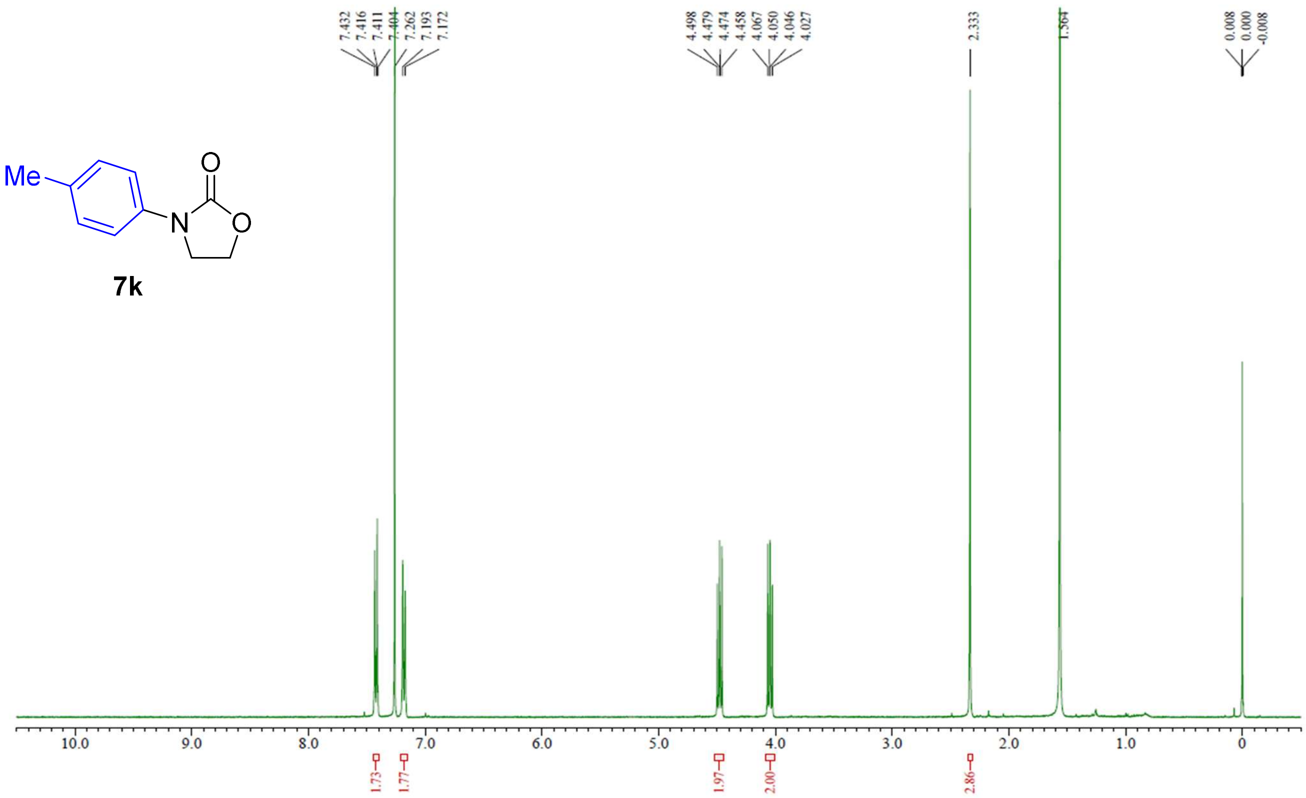

^{13}C{^{1}H} NMR ($CDCl_3$, 100 MHz) spectrum of compound **7k**

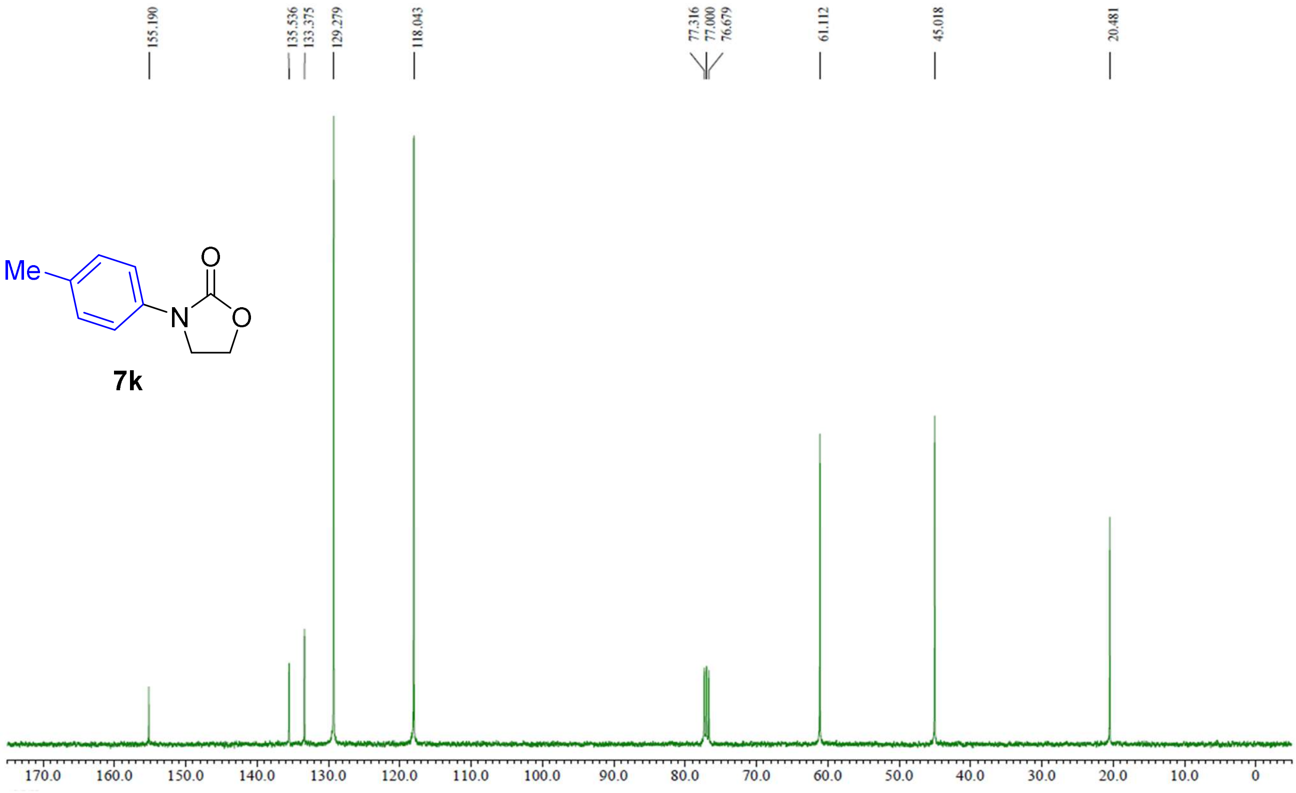

^{1}H NMR ($CDCl_3$, 400 MHz) spectrum of compound **7l**

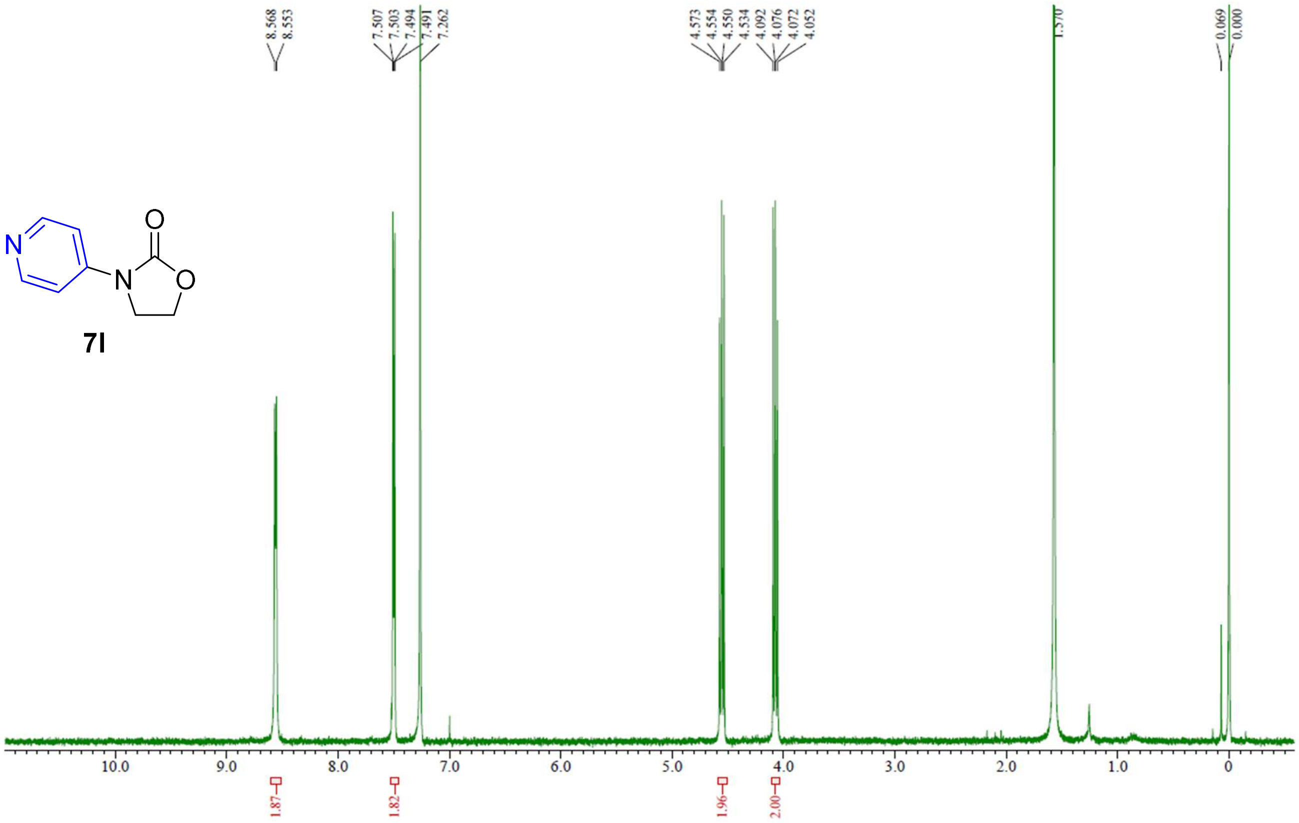

$^{13}C\{^{1}H\}$ NMR ($CDCl_3$, 100 MHz) spectrum of compound **7l**

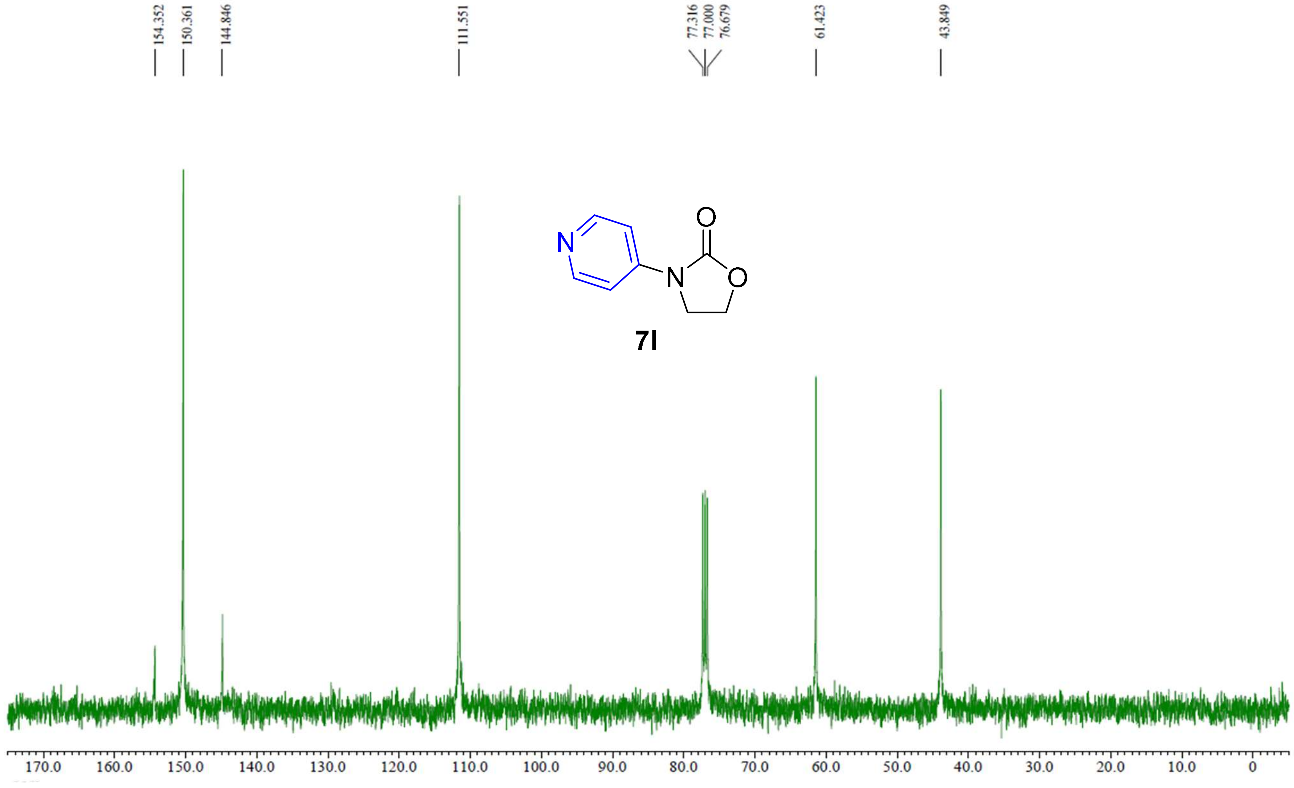

^{1}H NMR ($CDCl_3$, 400 MHz) spectrum of compound **7m**

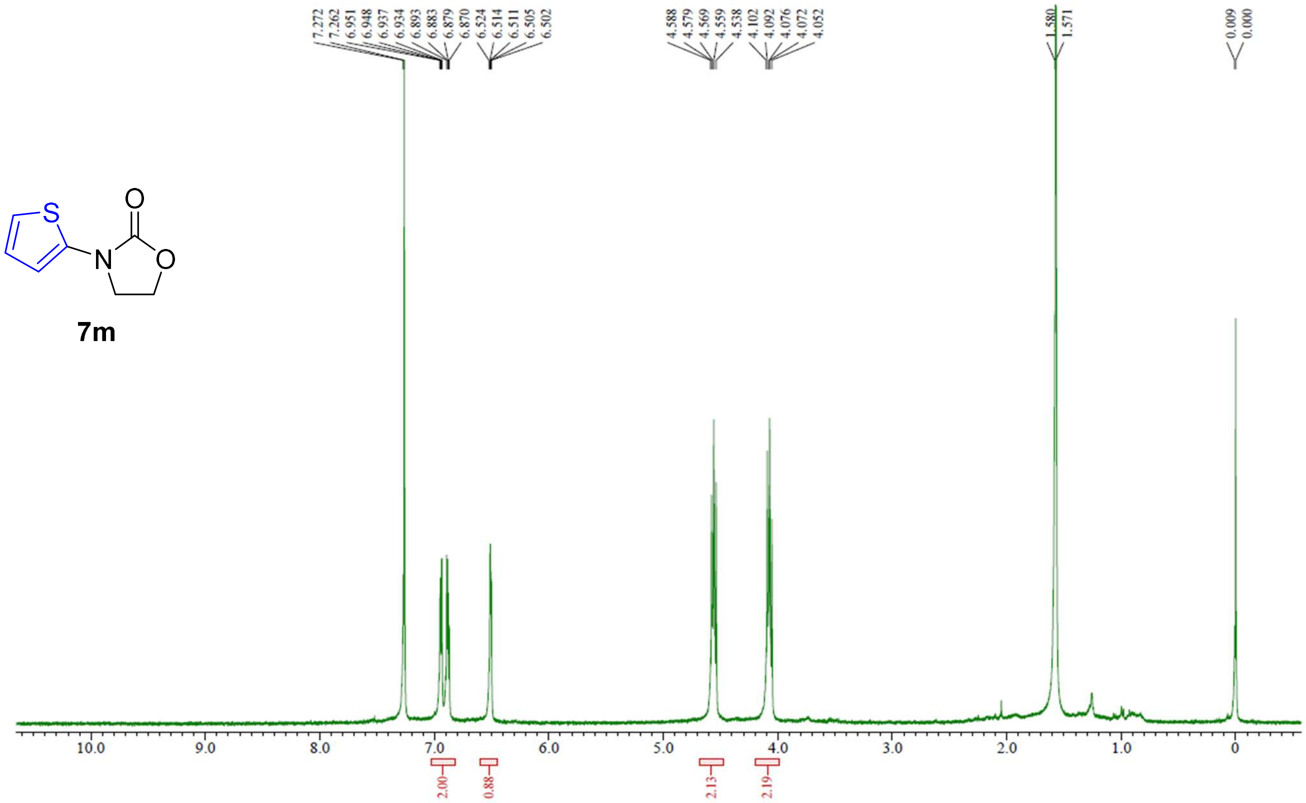

$^{13}C\{^1H\}$ NMR ($CDCl_3$, 100 MHz) spectrum of compound **7m**

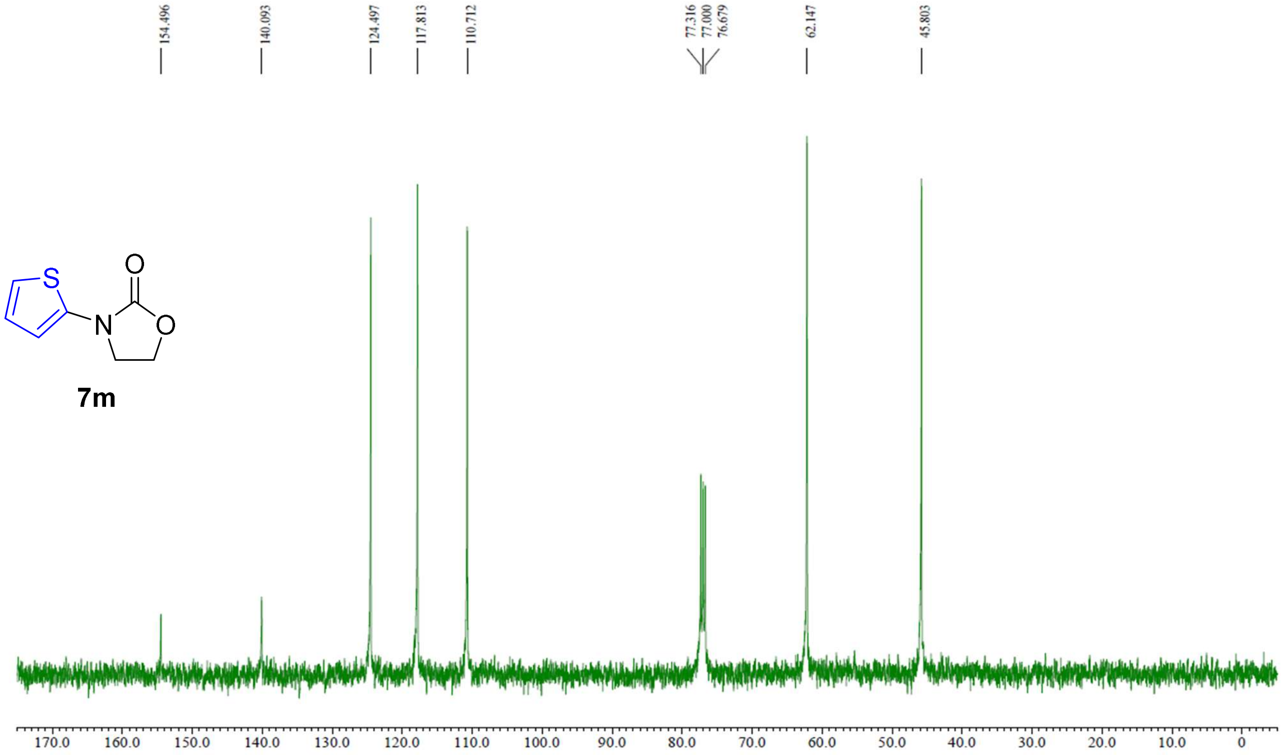

^{1}H NMR ($CDCl_3$, 400 MHz) spectrum of compound **7n**

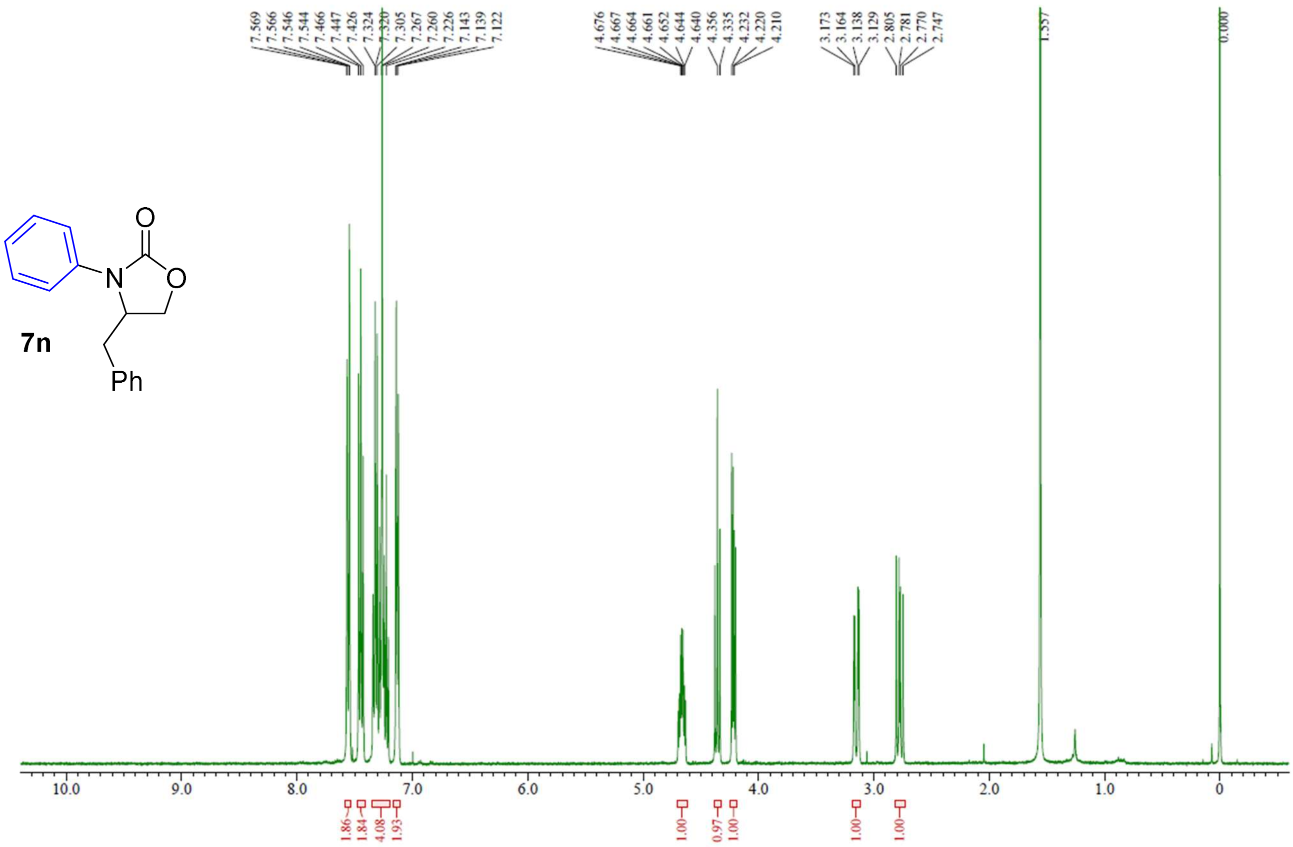

^{13}C{^{1}H} NMR ($CDCl_3$, 100 MHz) spectrum of compound **7n**

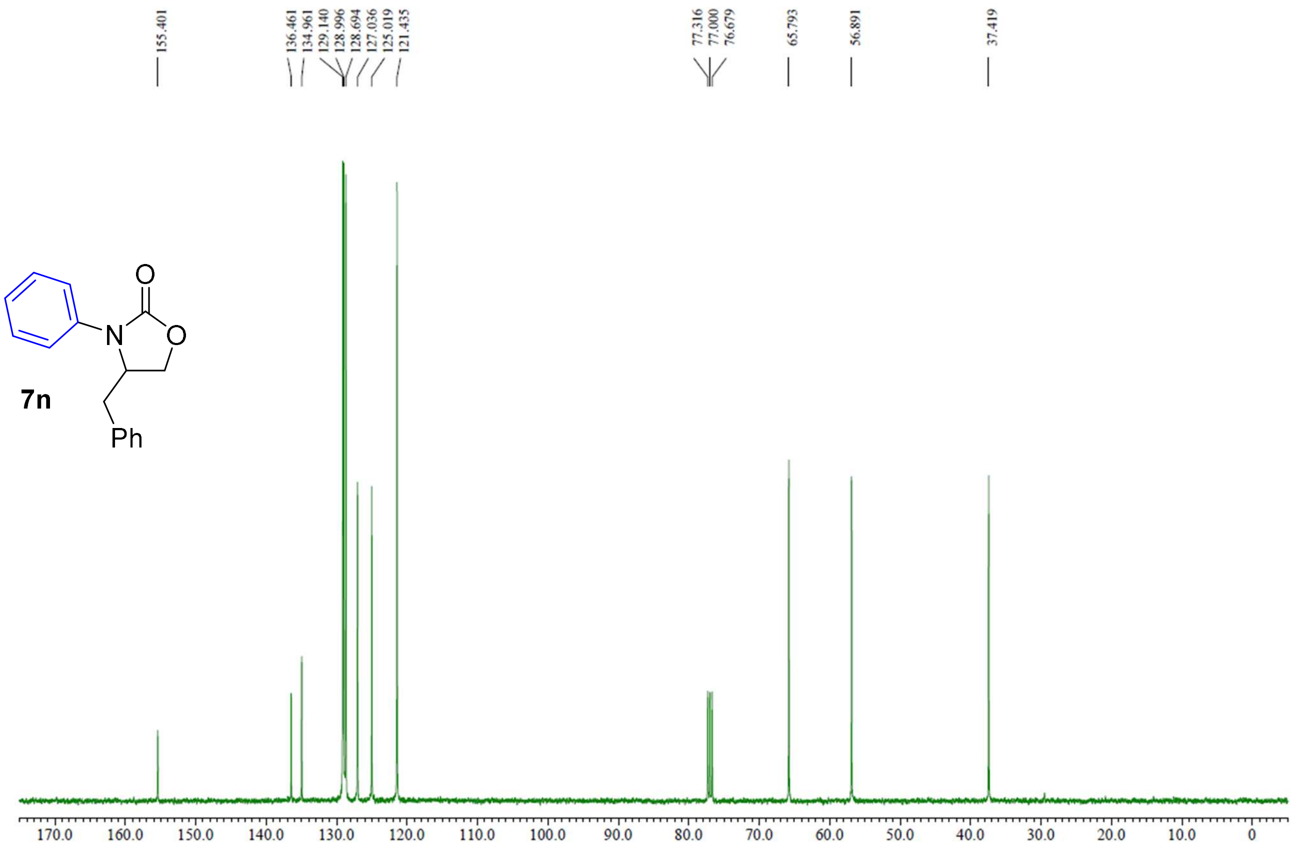

^{1}H NMR ($CDCl_3$, 400 MHz) spectrum of compound **9a**

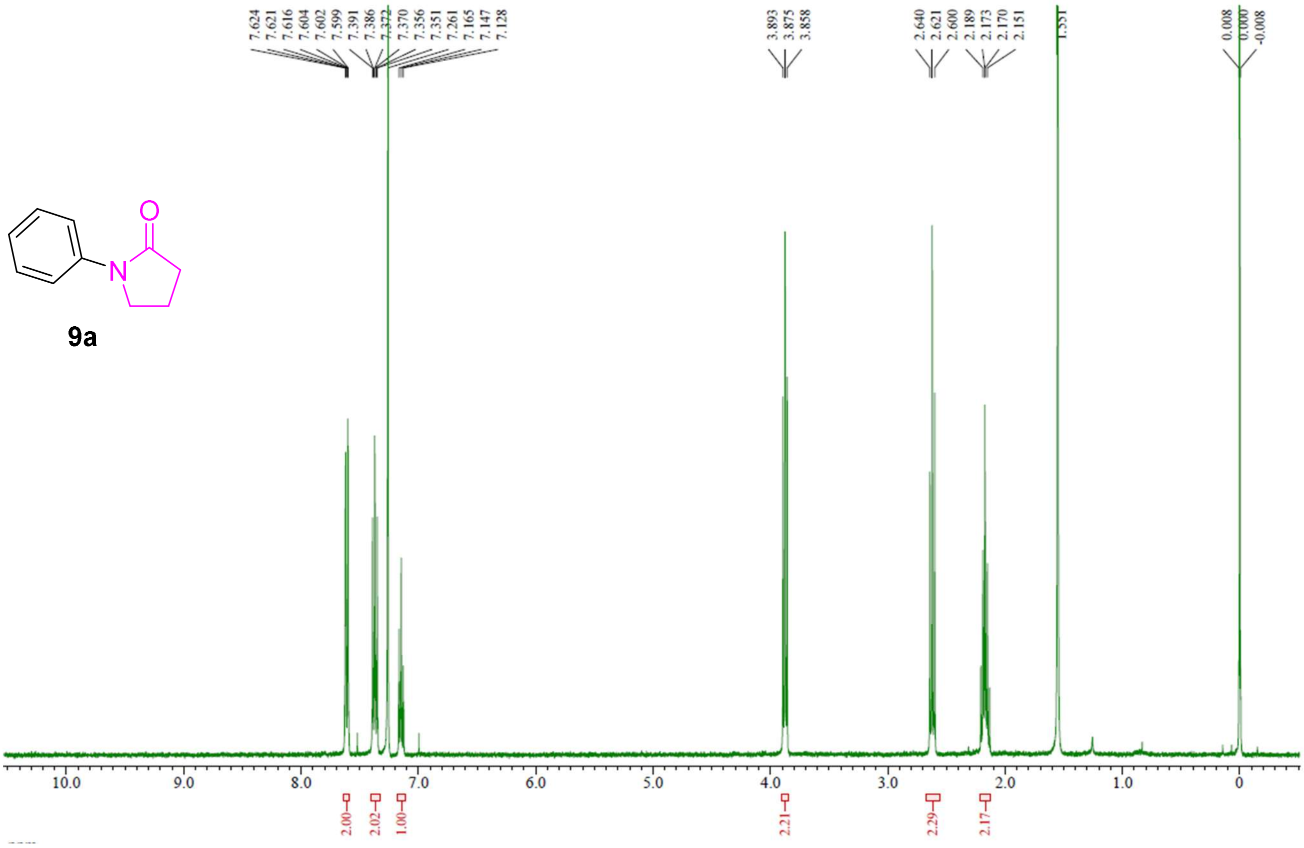

$^{13}C\{^{1}H\}$ NMR ($CDCl_3$, 100 MHz) spectrum of compound **9a**

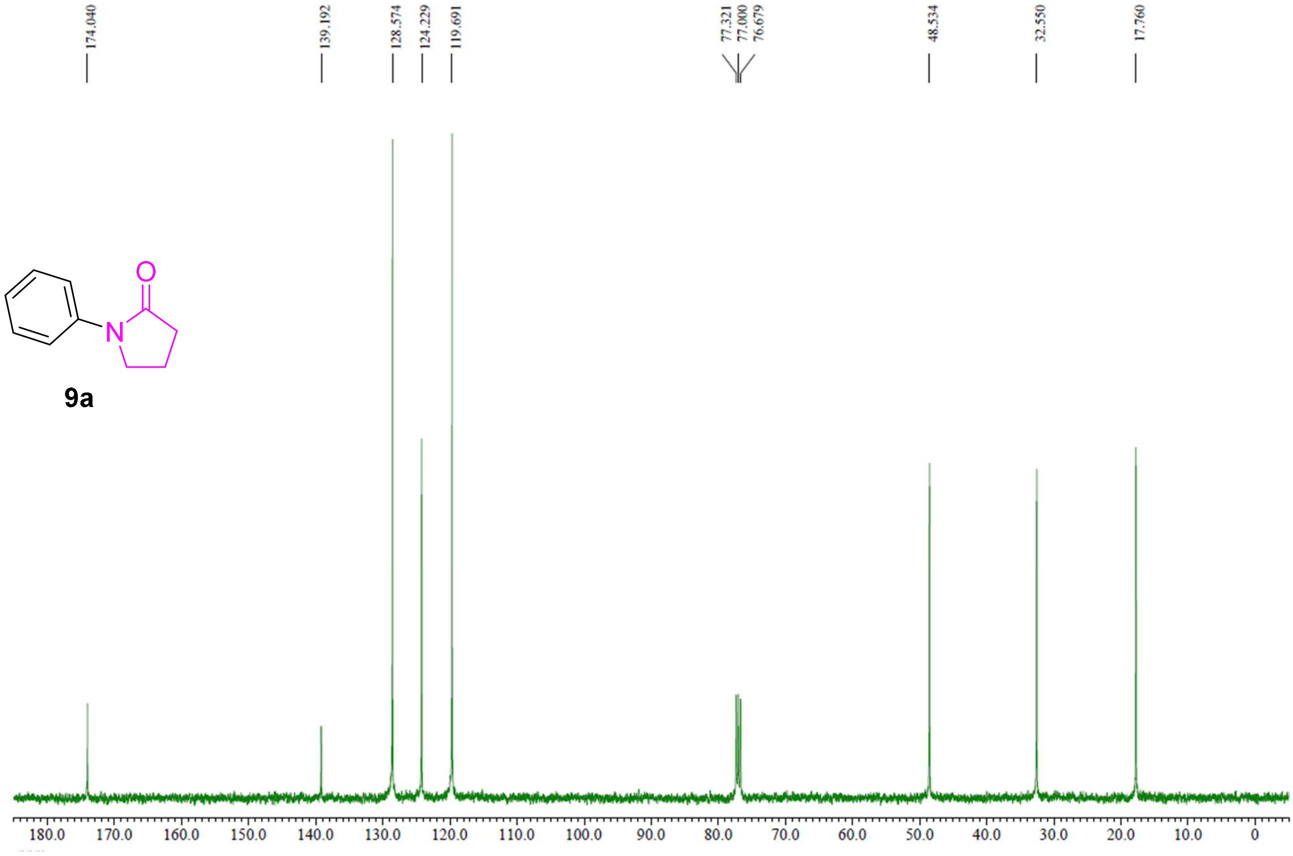

^{1}H NMR ($CDCl_3$, 400 MHz) spectrum of compound **9b**

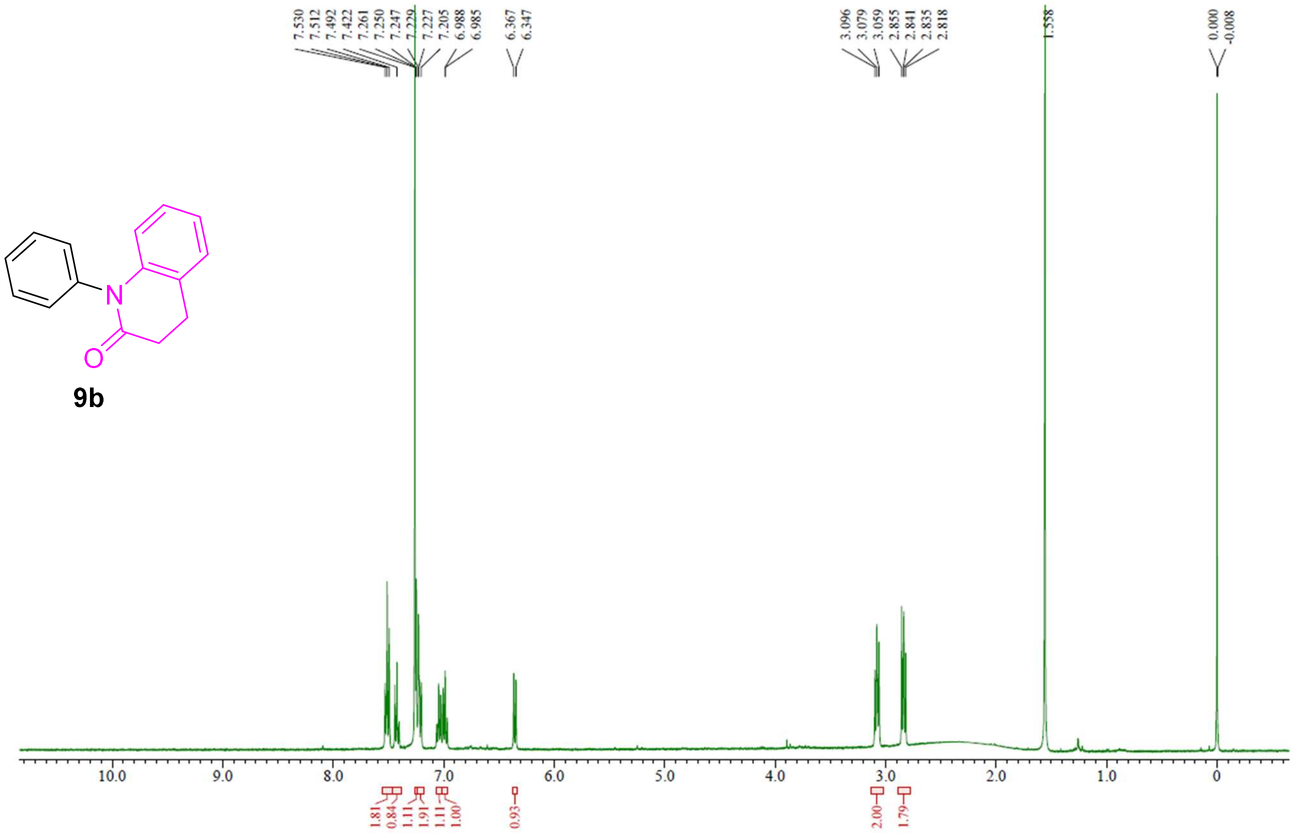

$^{13}C\{^{1}H\}$ NMR ($CDCl_3$, 100 MHz) spectrum of compound **9b**

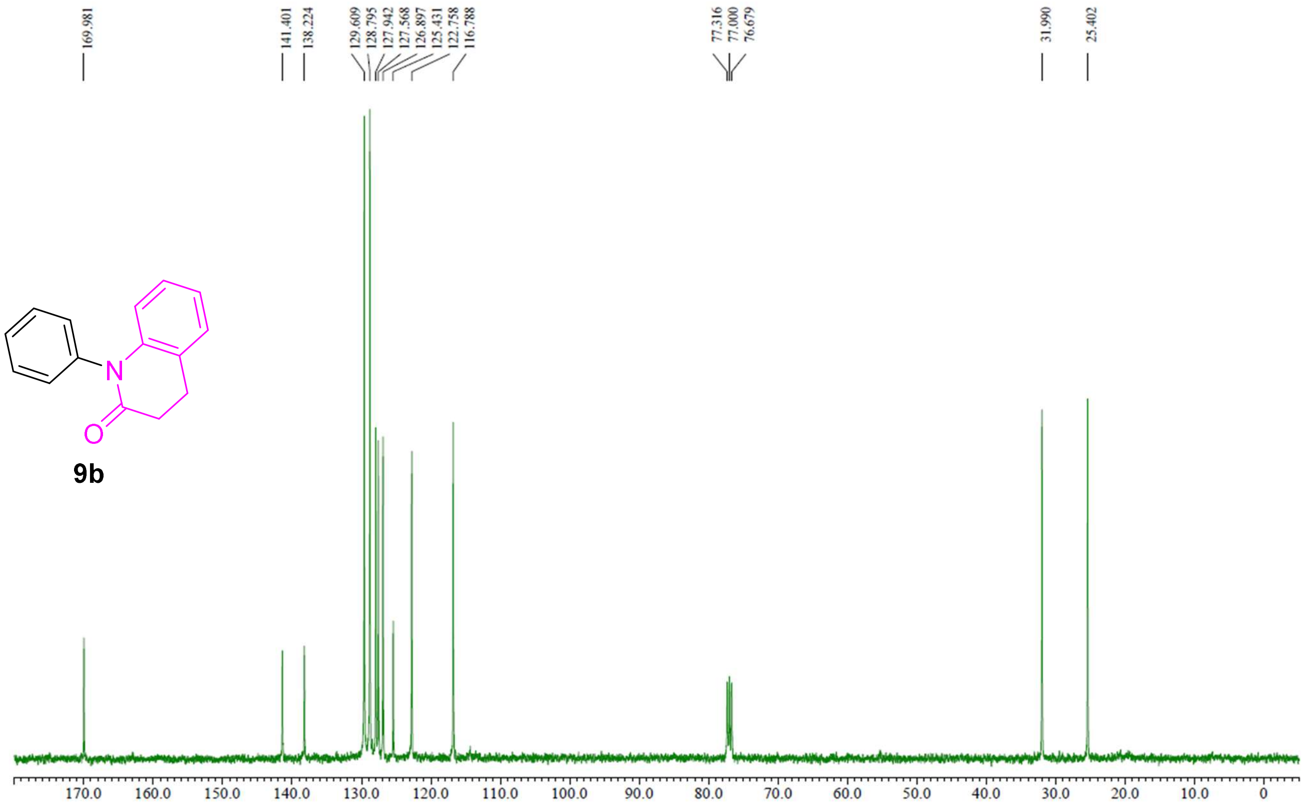

^{1}H NMR (CDCl₃, 400 MHz) spectrum of compound **9c**

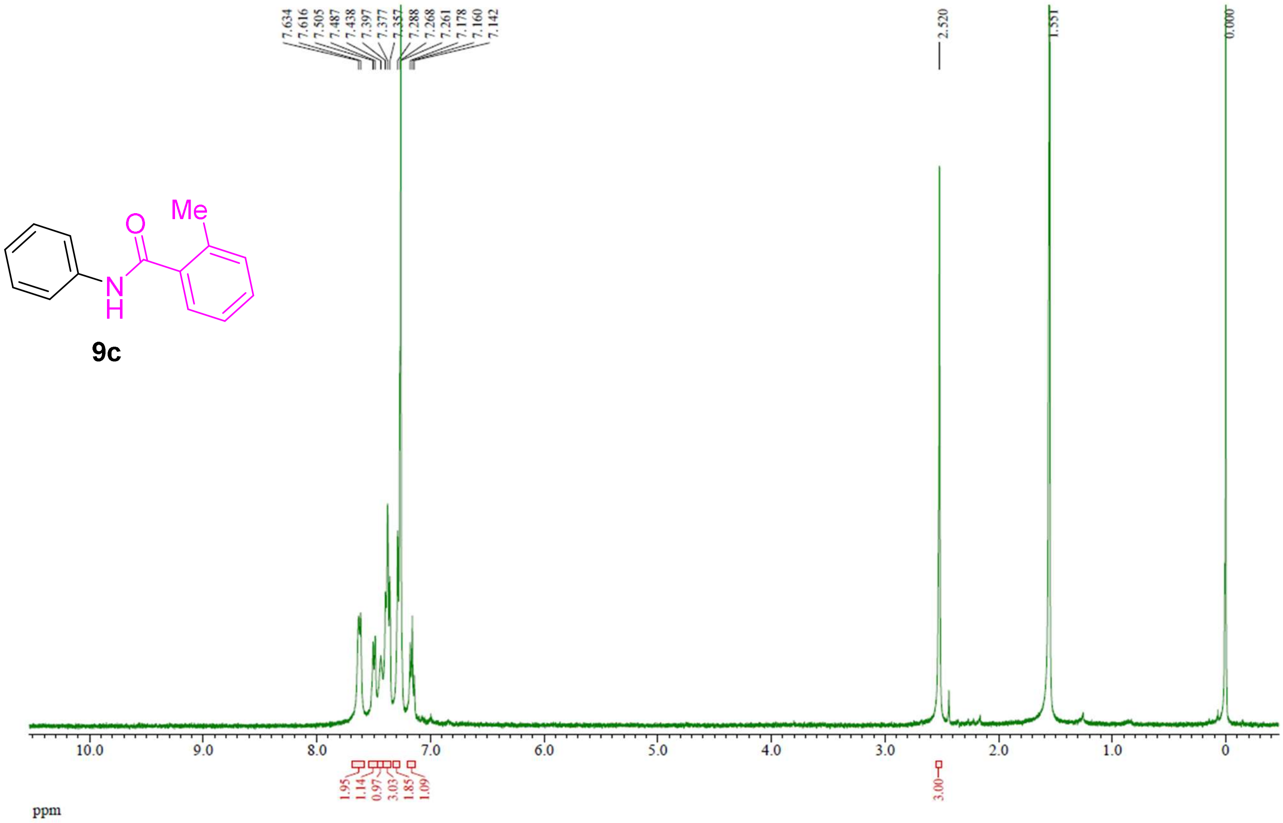

9c

^{13}C{^{1}H} NMR (CDCl₃, 100 MHz) spectrum of compound **9c**

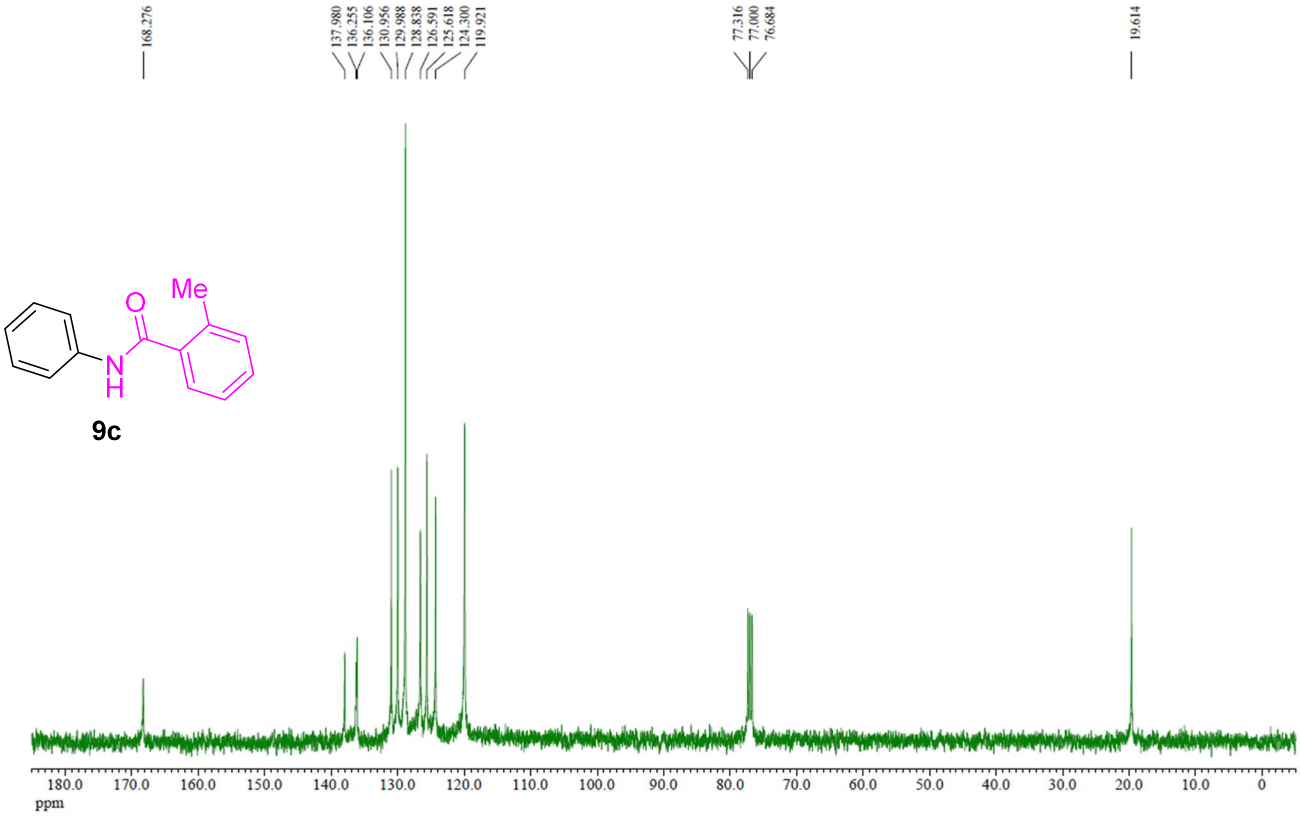

9c

^{1}H NMR ($CDCl_3$, 400 MHz) spectrum of compound **9d**

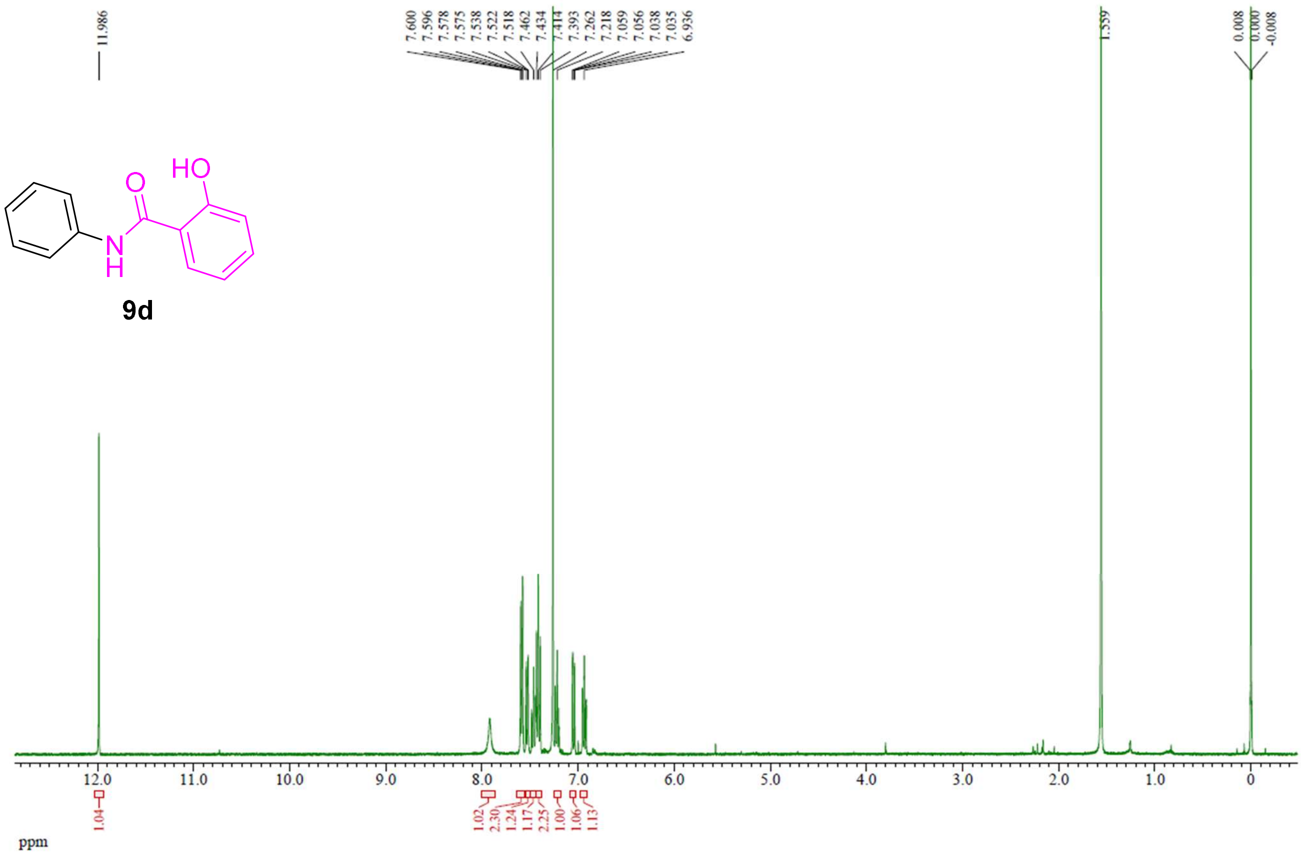

^{13}C{^{1}H} NMR ($CDCl_3$, 100 MHz) spectrum of compound **9d**

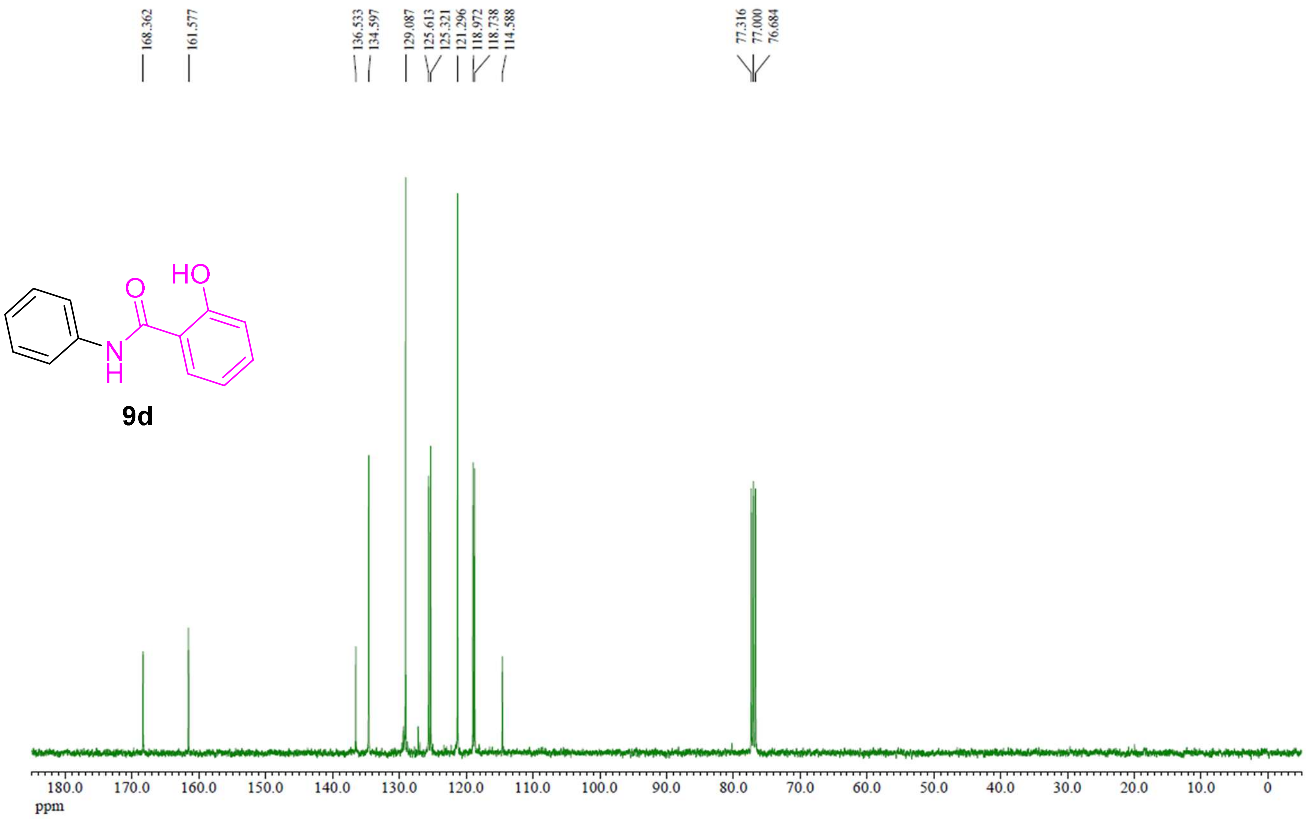

^{1}H NMR ($CDCl_3$, 400 MHz) spectrum of compound **9e**

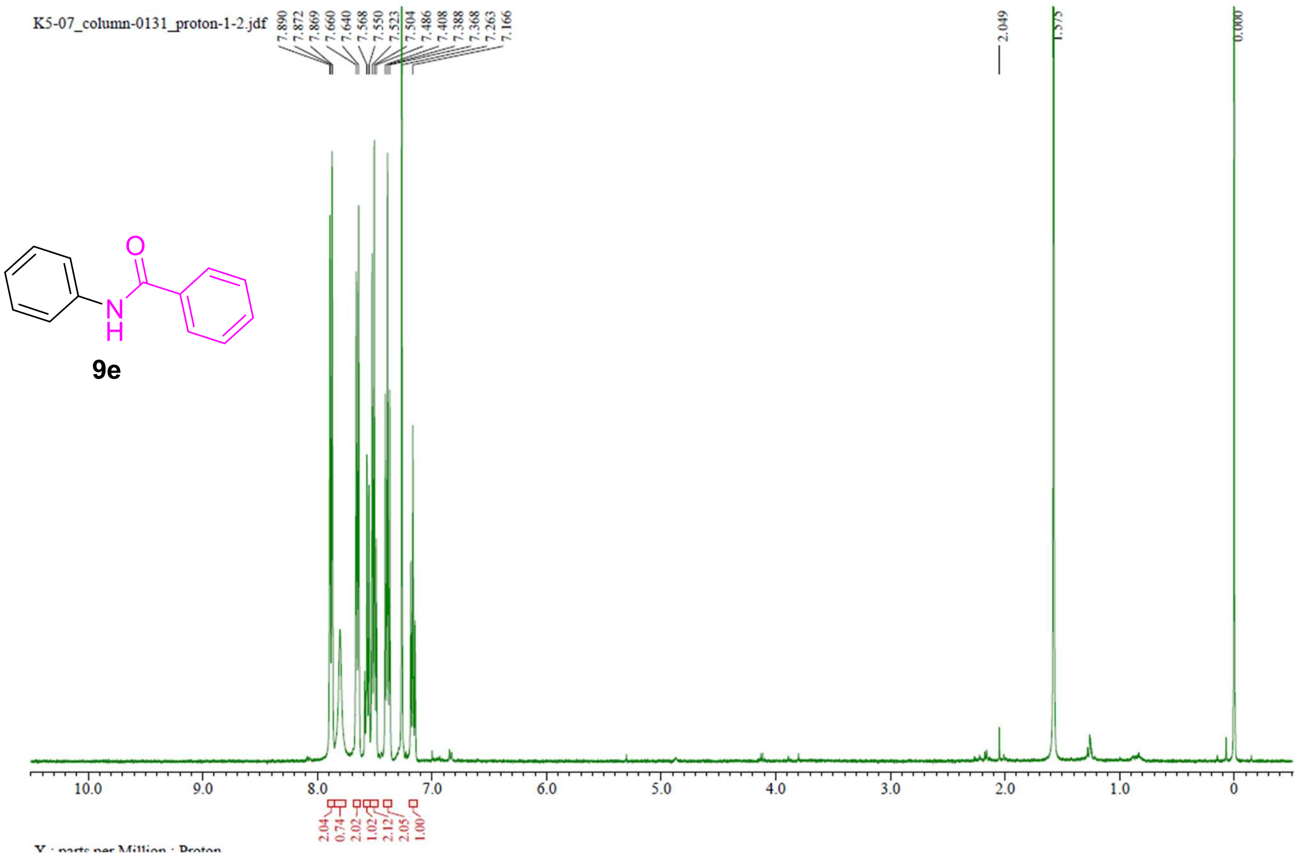

$^{13}C\{^{1}H\}$ NMR ($CDCl_3$, 100 MHz) spectrum of compound **9e**

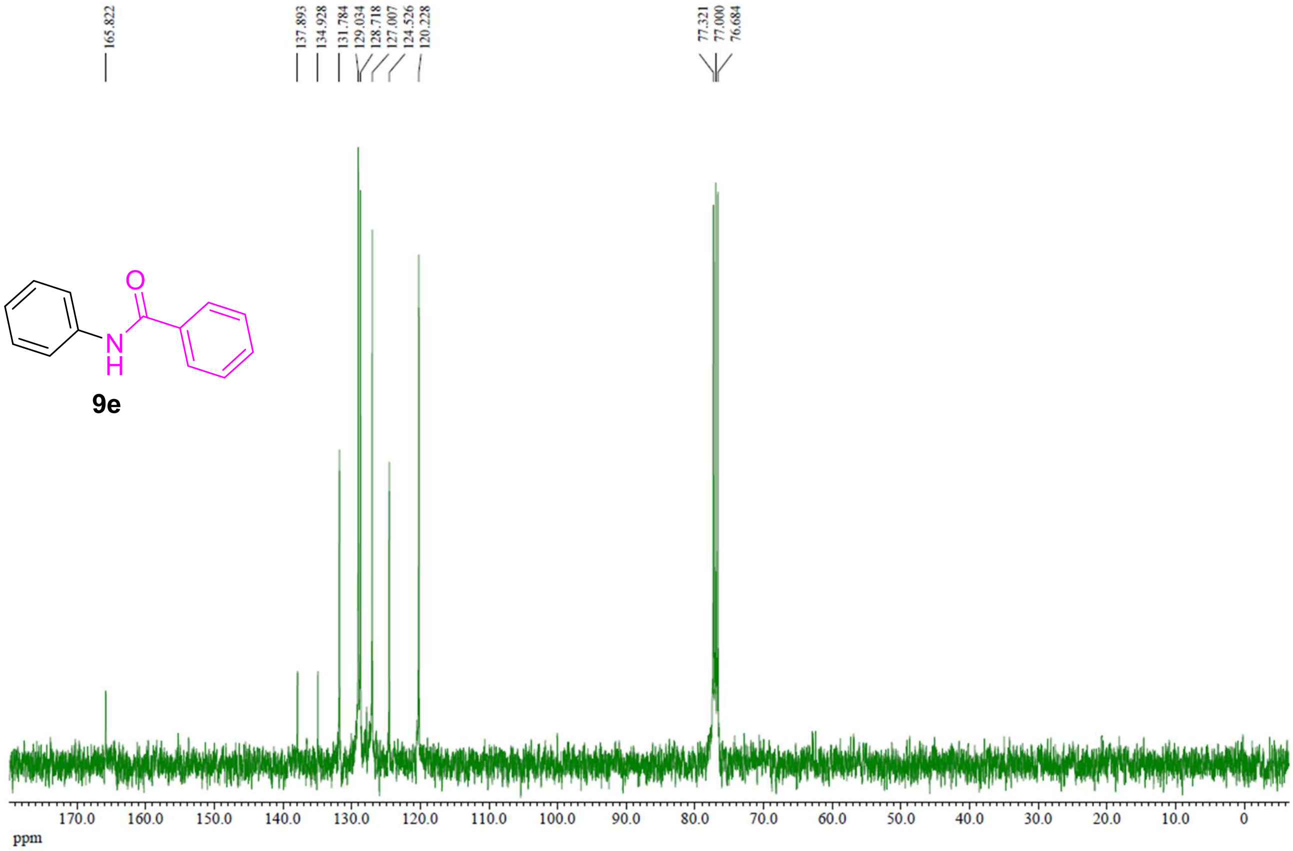

^{1}H NMR ($CDCl_3$, 400 MHz) spectrum of compound **9f**

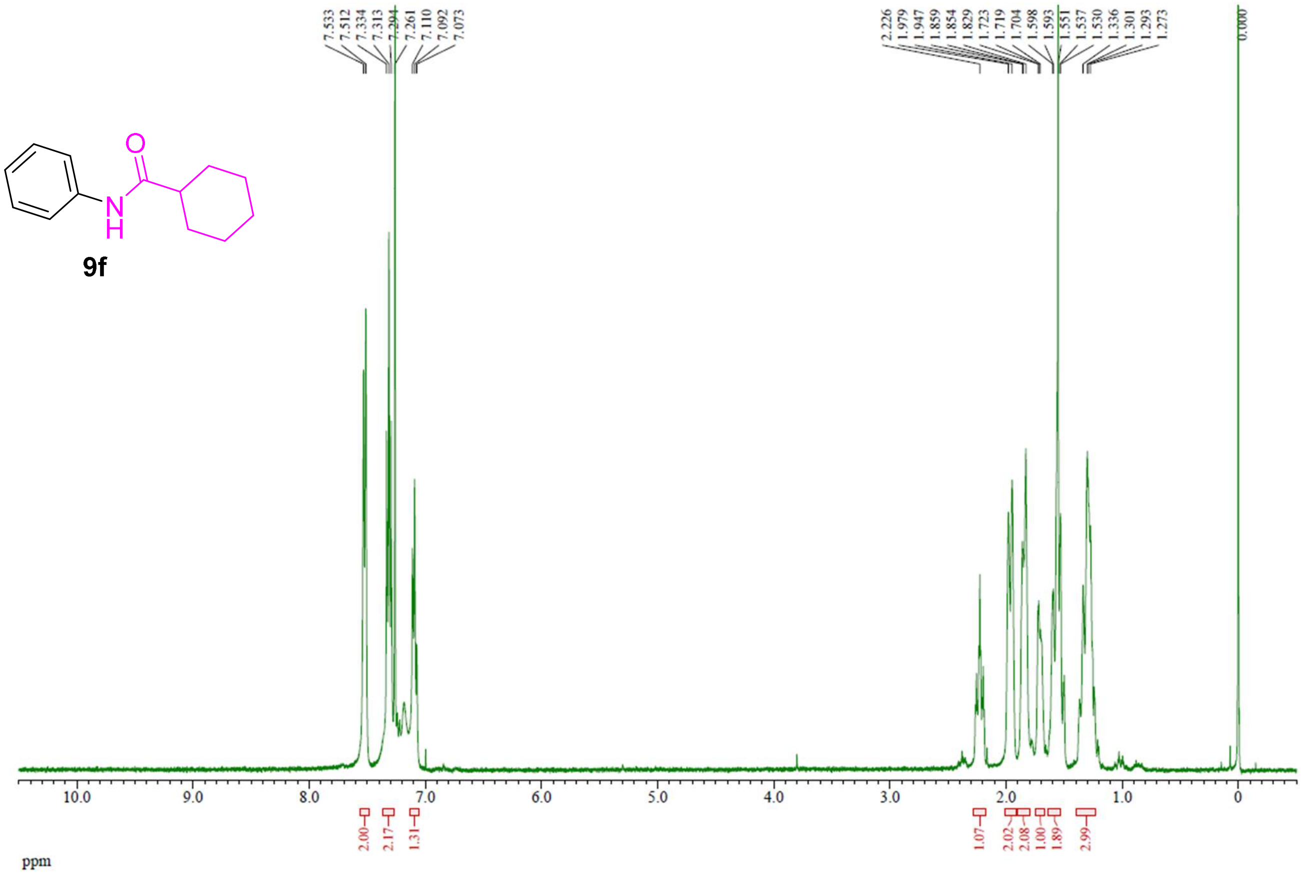

^{13}C{^{1}H} NMR ($CDCl_3$, 100 MHz) spectrum of compound **9f**

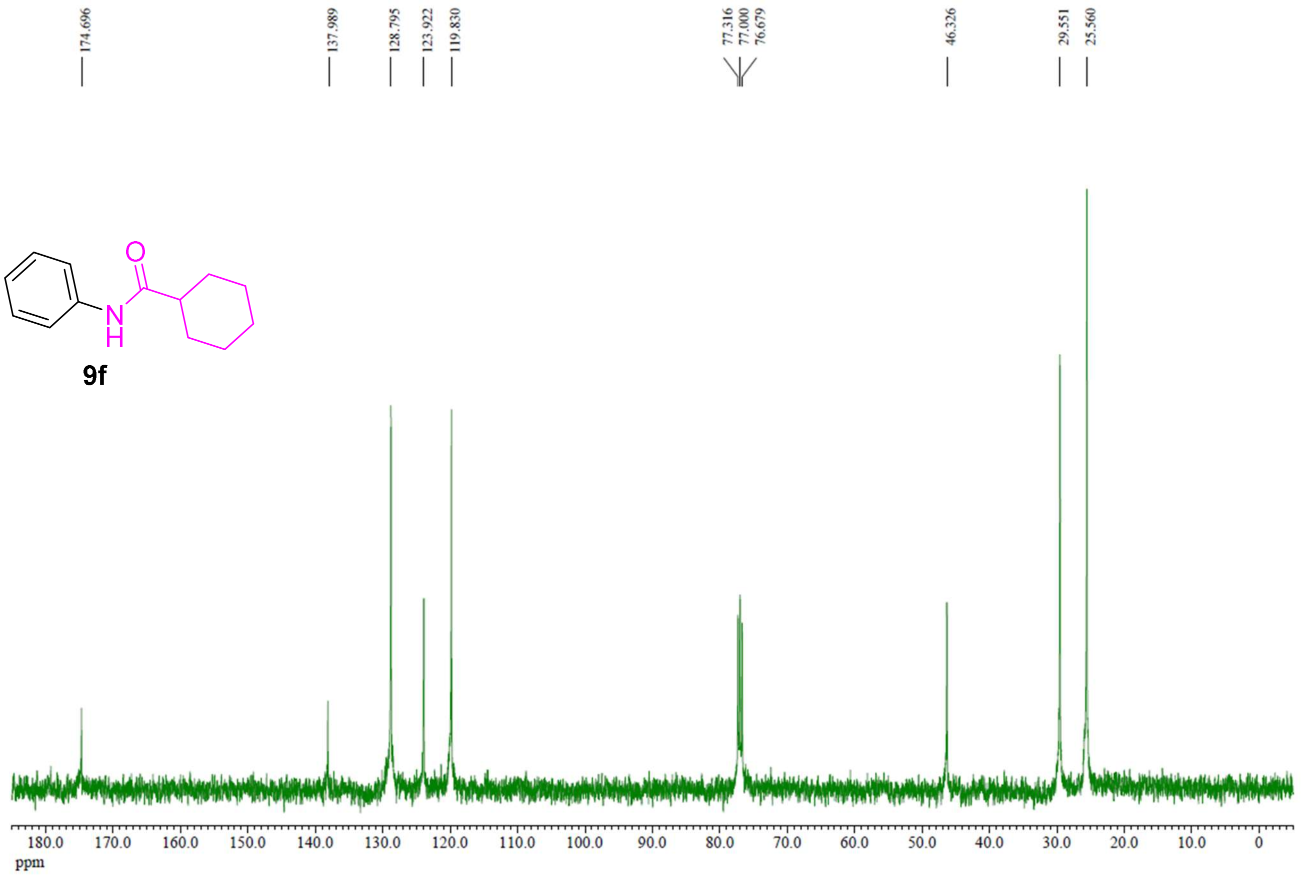

^{1}H NMR ($CDCl_3$, 400 MHz) spectrum of compound **9g**

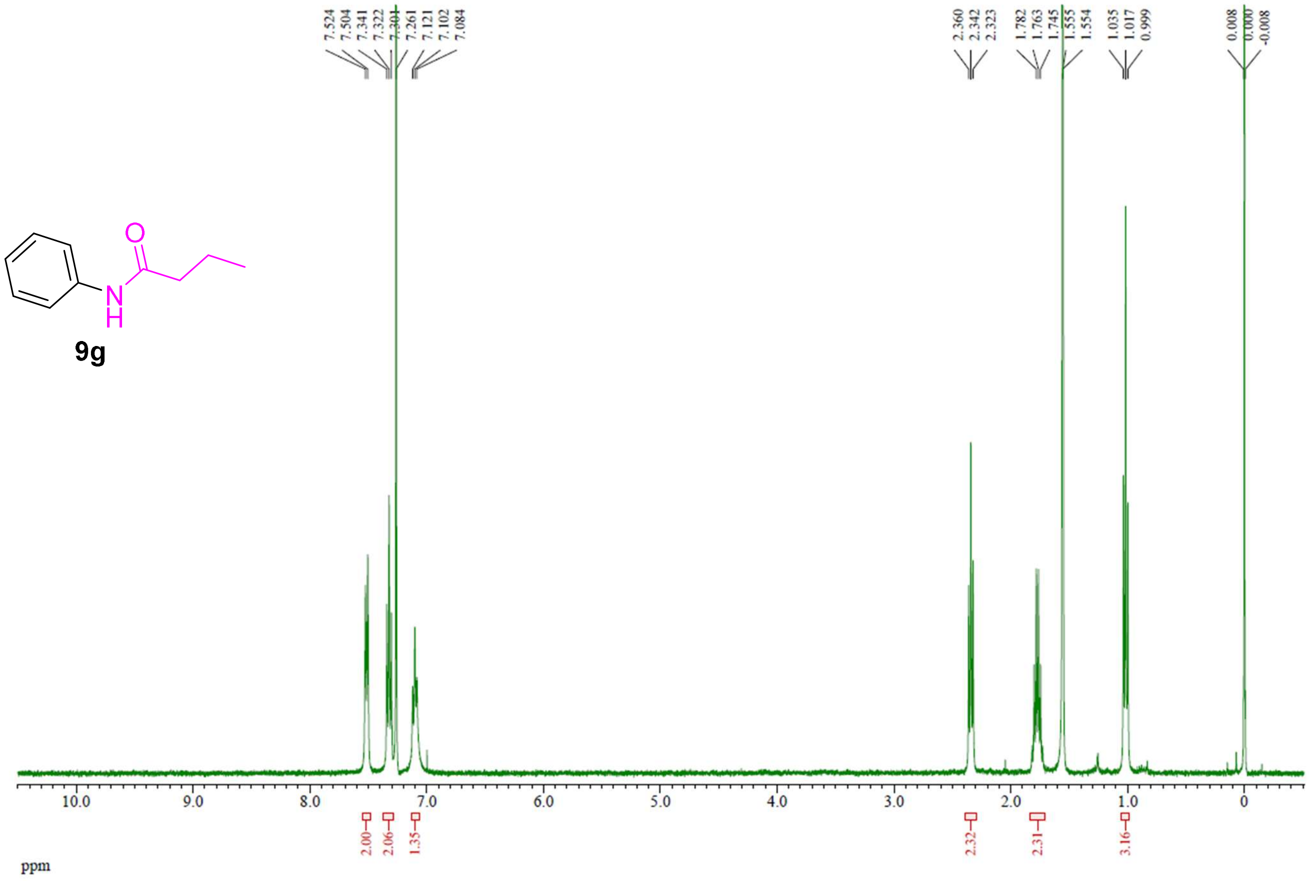

$^{13}C\{^{1}H\}$ NMR ($CDCl_3$, 100 MHz) spectrum of compound **9g**

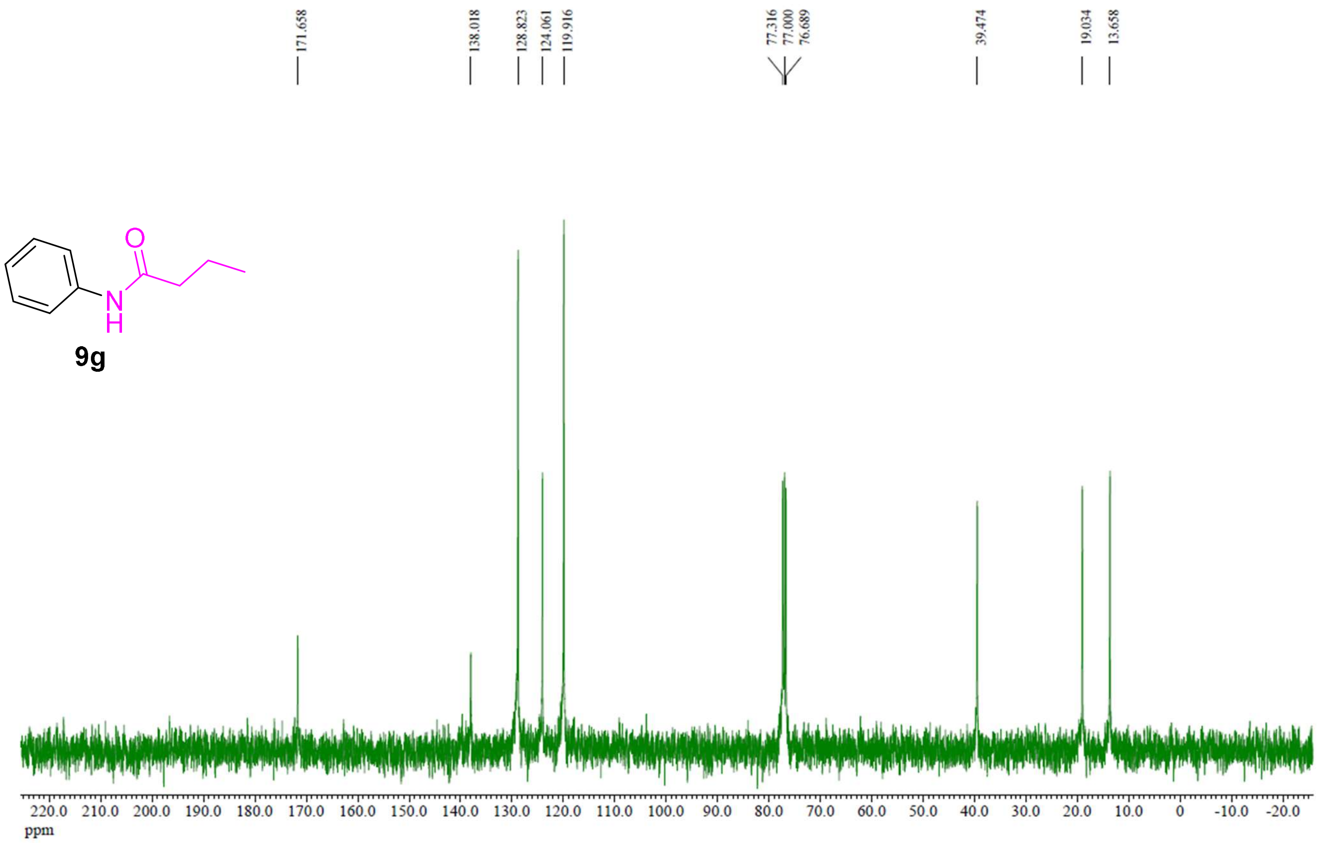

^{1}H NMR ($CDCl_3$, 400 MHz) spectrum of compound **9h**

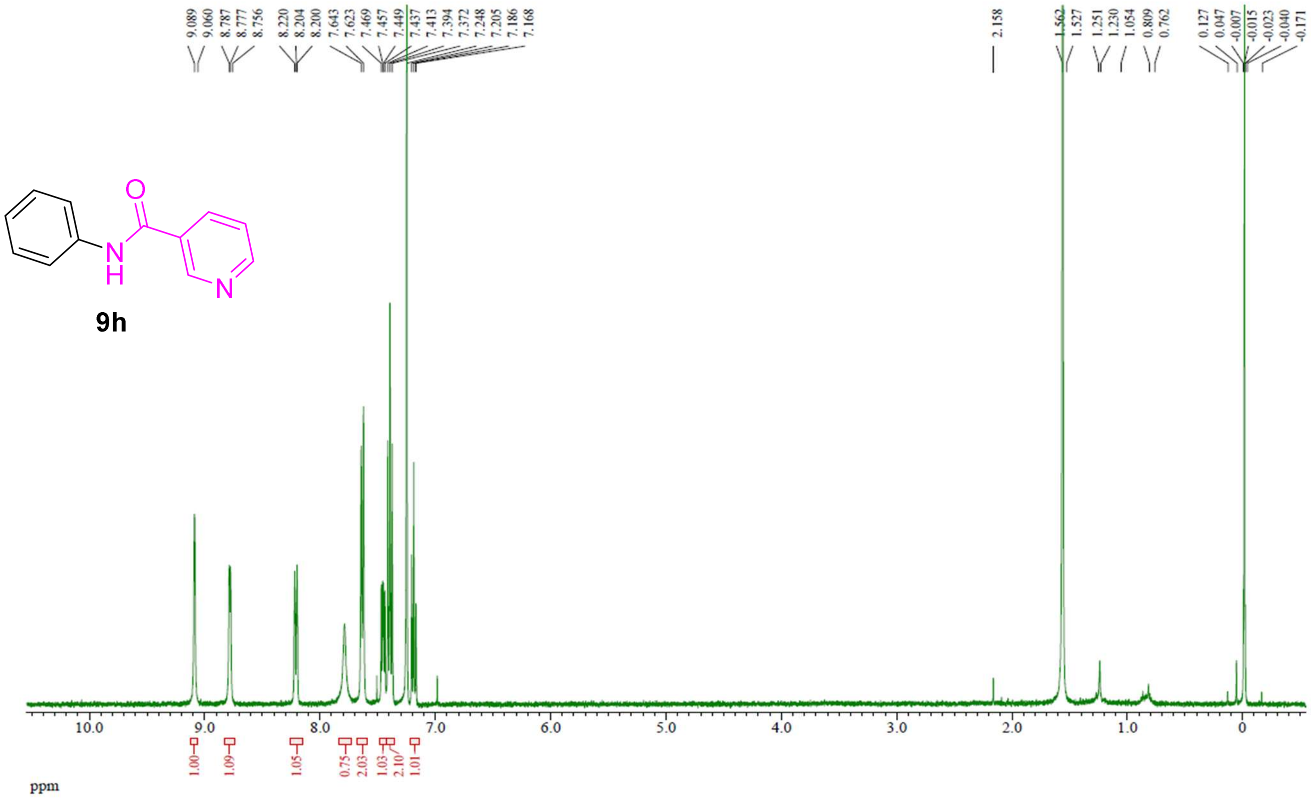

$^{13}C\{^{1}H\}$ NMR ($CDCl_3$, 100 MHz) spectrum of compound **9h**

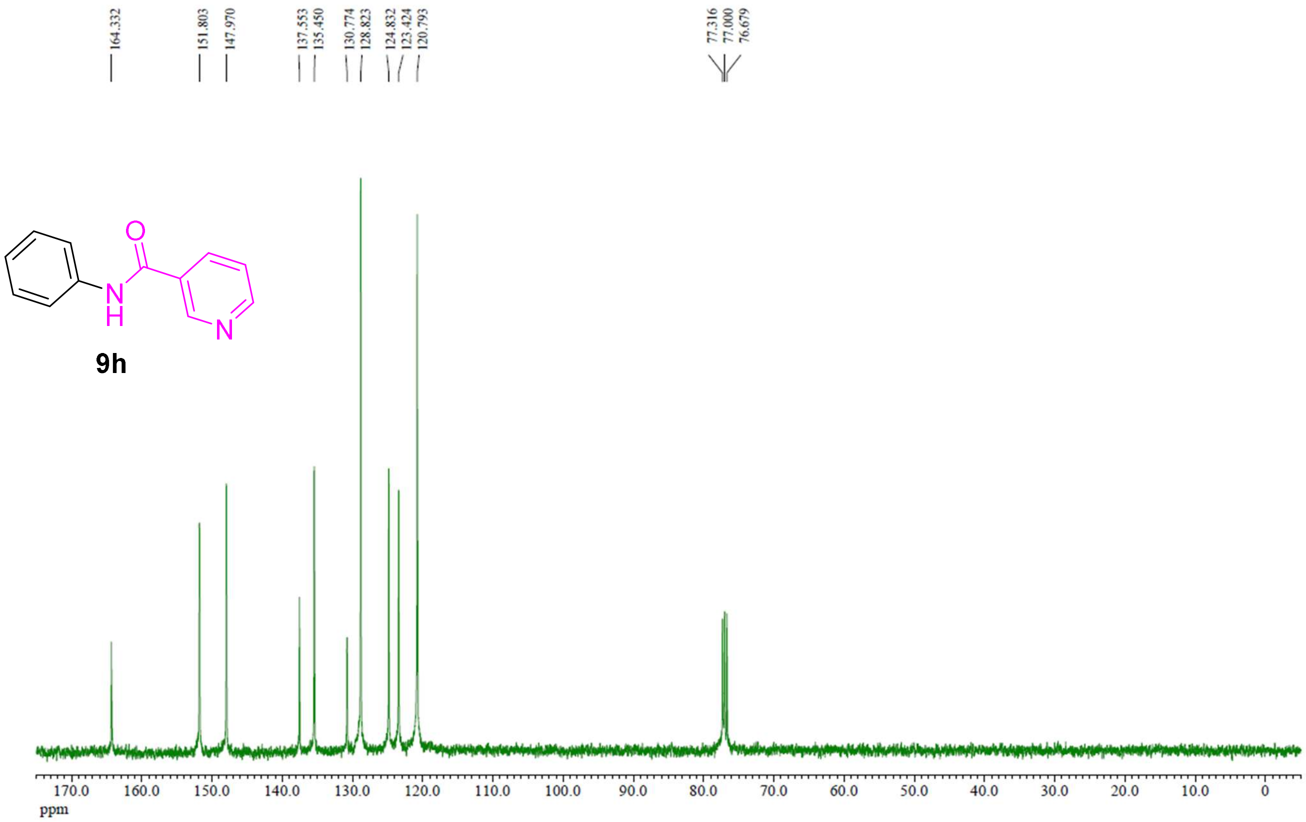

^{1}H NMR ($CDCl_3$, 400 MHz) spectrum of compound **9i**

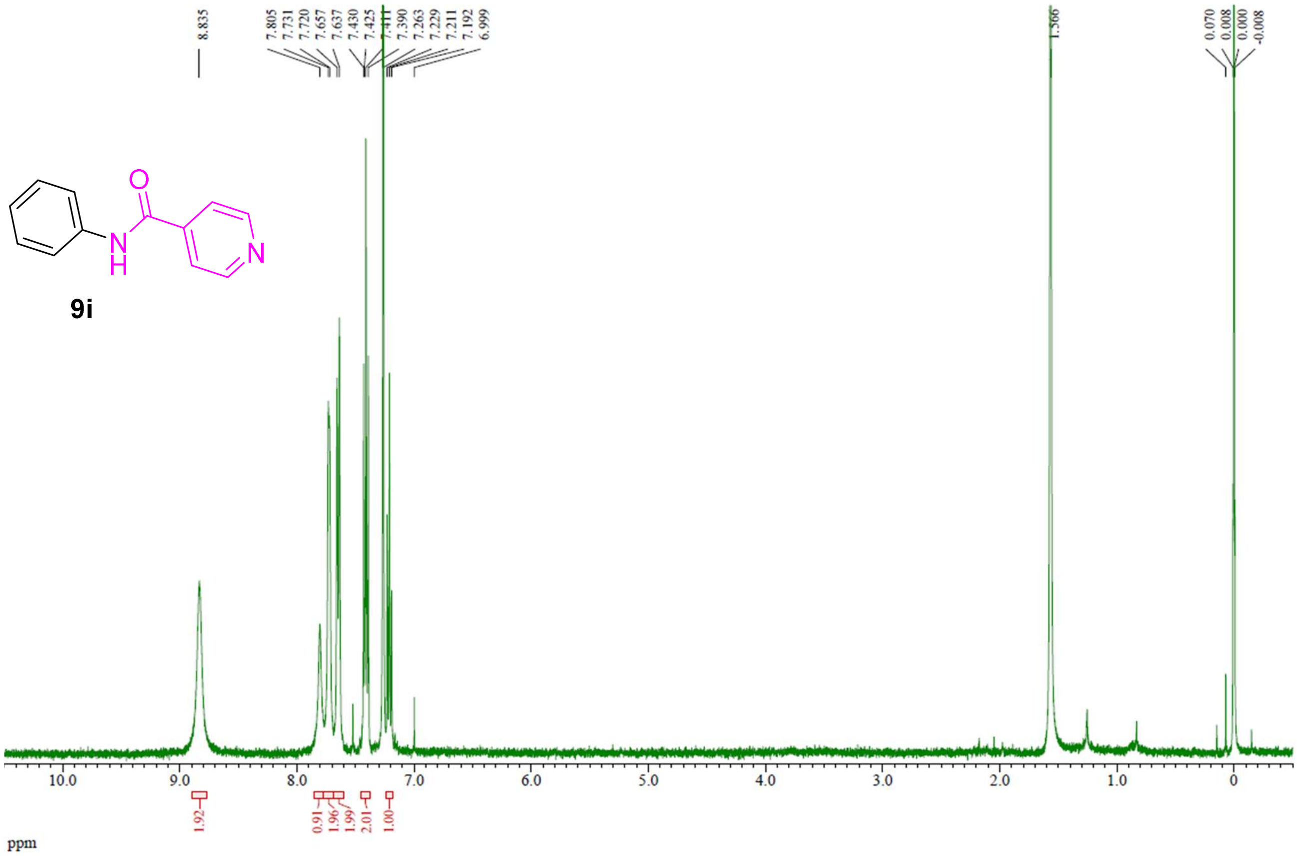

$^{13}C\{^{1}H\}$ NMR ($CDCl_3$, 100 MHz) spectrum of compound **9i**

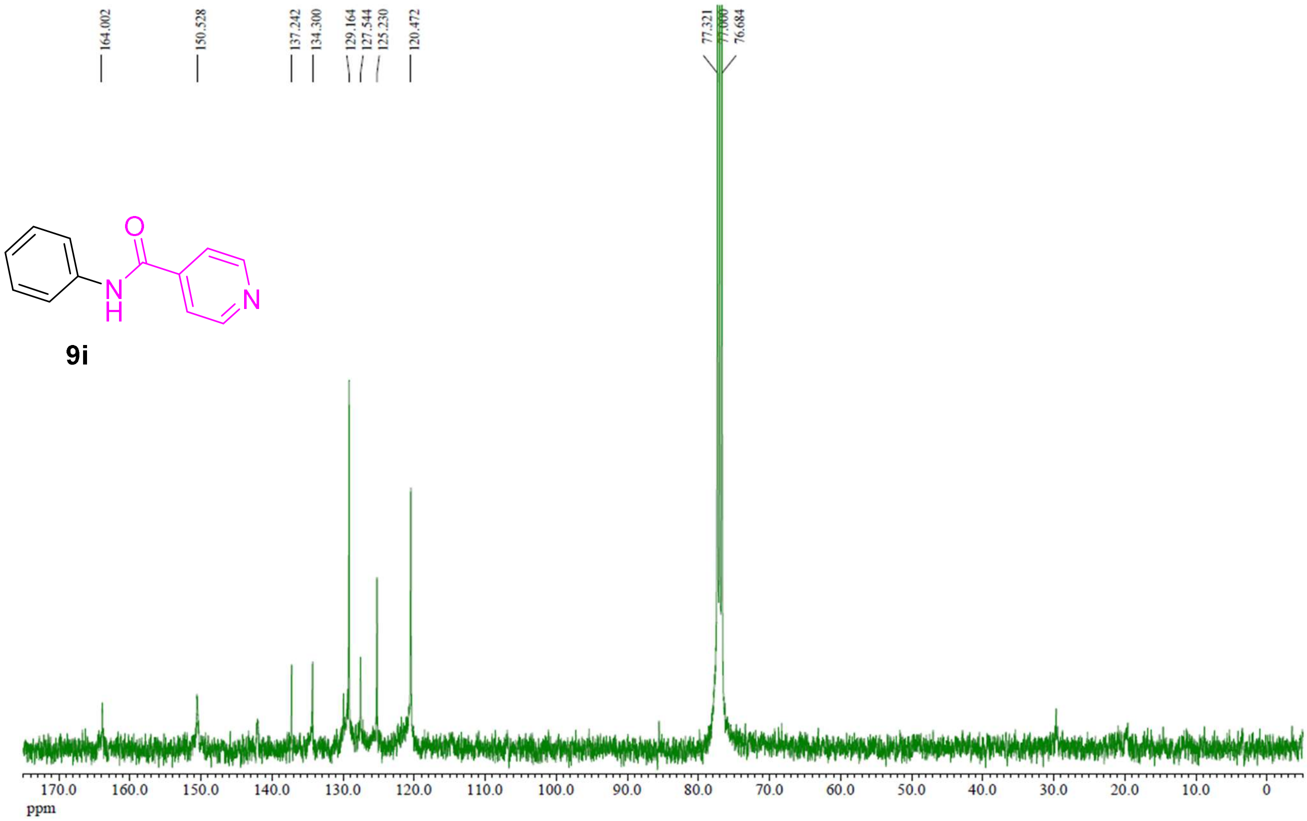

4. X-ray crystallographic analysis

Table 1. Crystal data and structure refinement for **2a**. (CCDC2402285)

Identification code	**2a**	
Empirical formula	C33 H31 Br Cu N3 O	
Formula weight	629.06	
Temperature	150(2) K	
Wavelength	0.71073 Å	
Crystal system	Triclinic	
Space group	P-1	
Unit cell dimensions	a = 9.9951(6) Å	α= 99.719(3)°.
	b = 11.3145(8) Å	β= 99.027(2)°.
	c = 14.5799(10) Å	γ = 112.269(2)°.
Volume	1459.20(17) $Å^3$	
Z	2	
Density (calculated)	1.432 Mg/m^3	
Absorption coefficient	2.148 mm^{-1}	
F(000)	644	
Crystal size	0.250 x 0.160 x 0.070 mm^3	
Theta range for data collection	2.717 to 27.522°.	
Index ranges	-12<=h<=12, -14<=k<=14, -18<=l<=18	
Reflections collected	53448	
Independent reflections	6685 [R(int) = 0.0425]	
Completeness to theta = 25.242°	99.8 %	
Absorption correction	Semi-empirical from equivalents	
Max. and min. transmission	0.7456 and 0.6245	
Refinement method	Full-matrix least-squares on F^2	
Data / restraints / parameters	6685 / 1 / 359	
Goodness-of-fit on F^2	1.021	
Final R indices [I>2sigma(I)]	R1 = 0.0647, wR2 = 0.2119	
R indices (all data)	R1 = 0.0755, wR2 = 0.2246	
Extinction coefficient	n/a	
Largest diff. peak and hole	1.307 and -2.546 e.$Å^{-3}$	

Table 2. Atomic coordinates (x 10^4) and equivalent isotropic displacement parameters ($Å^2$x 10^3)
for E2. U(eq) is defined as one third of the trace of the orthogonalized U^{ij} tensor.

	x	y	z	U(eq)
Cu	4077(1)	5780(1)	2472(1)	23(1)
Br	5637(1)	5133(1)	1851(1)	56(1)
O	5722(4)	10507(4)	1538(3)	39(1)
N(1)	5704(3)	8286(3)	4666(2)	16(1)
N(2)	3106(4)	6754(3)	4084(2)	18(1)
N(3)	1436(4)	6218(4)	2769(3)	23(1)
C(1)	6855(4)	8565(4)	5447(3)	16(1)
C(2)	8250(4)	9628(4)	5772(3)	21(1)
C(3)	9122(5)	9723(4)	6635(3)	26(1)
C(4)	8656(5)	8768(5)	7158(3)	27(1)
C(5)	7291(5)	7712(4)	6837(3)	24(1)
C(6)	6343(4)	7606(4)	5970(3)	18(1)
C(7)	4839(5)	6725(4)	5492(3)	21(1)
C(8)	4496(4)	7172(4)	4720(3)	16(1)
C(9)	5743(4)	8940(4)	3908(3)	17(1)
C(10)	6787(5)	9015(4)	3368(3)	23(1)
C(11)	6761(5)	9572(5)	2595(3)	28(1)
C(12)	5679(5)	10033(4)	2341(3)	25(1)
C(13)	4647(5)	9970(4)	2890(3)	22(1)
C(14)	4688(4)	9429(4)	3678(3)	19(1)
C(15)	4600(30)	10950(20)	1210(30)	56(5)
C(15')	4790(60)	11180(50)	1380(70)	56(5)
C(16)	3814(5)	5563(4)	5767(4)	31(1)
C(17)	1916(4)	6966(4)	4353(3)	22(1)
C(18)	1708(5)	7408(4)	5243(3)	27(1)
C(19)	343(6)	7474(4)	5242(4)	34(1)
C(20)	-731(5)	7127(4)	4391(4)	36(1)
C(21)	-513(5)	6703(4)	3512(4)	32(1)
C(22)	851(4)	6626(4)	3511(3)	23(1)
C(23)	2815(4)	6294(4)	3108(3)	20(1)
C(24)	698(6)	5881(5)	1749(4)	34(1)
C(25)	974(5)	4818(5)	1152(3)	28(1)
C(26)	210(6)	3511(5)	1186(4)	36(1)
C(27)	438(8)	2523(6)	610(4)	49(2)
C(28)	1431(8)	2822(7)	23(4)	53(2)

C(29)	2183(7)	4113(7)	10(4)	48(2)
C(30)	1979(6)	5126(6)	570(3)	36(1)
C(31)	-841(7)	3158(7)	1827(5)	52(2)
C(32)	1910(40)	1950(30)	-640(20)	63(5)
C(32')	1500(40)	1530(30)	-530(20)	63(5)
C(33)	2866(7)	6519(7)	524(5)	53(2)

Table 3. Bond lengths [Å] and angles [°] for E2.

Cu-C(23)	1.878(4)
Cu-Br	2.2076(8)
O-C(12)	1.367(5)
O-C(15')	1.420(2)
O-C(15)	1.438(12)
N(1)-C(1)	1.382(5)
N(1)-C(8)	1.400(5)
N(1)-C(9)	1.428(5)
N(2)-C(23)	1.373(5)
N(2)-C(17)	1.399(5)
N(2)-C(8)	1.402(5)
N(3)-C(23)	1.354(5)
N(3)-C(22)	1.400(6)
N(3)-C(24)	1.466(6)
C(1)-C(2)	1.395(5)
C(1)-C(6)	1.415(5)
C(2)-C(3)	1.376(6)
C(2)-H(2A)	0.9500
C(3)-C(4)	1.404(7)
C(3)-H(3A)	0.9500
C(4)-C(5)	1.373(6)
C(4)-H(4A)	0.9500
C(5)-C(6)	1.413(6)
C(5)-H(5A)	0.9500
C(6)-C(7)	1.429(5)
C(7)-C(8)	1.358(6)
C(7)-C(16)	1.492(5)
C(9)-C(14)	1.387(5)
C(9)-C(10)	1.389(5)
C(10)-C(11)	1.382(6)
C(10)-H(10A)	0.9500
C(11)-C(12)	1.396(6)
C(11)-H(11A)	0.9500
C(12)-C(13)	1.390(6)
C(13)-C(14)	1.392(6)
C(13)-H(13A)	0.9500
C(14)-H(14A)	0.9500
C(15)-H(15A)	0.9800
C(15)-H(15B)	0.9800

C(15)-H(15C)	0.9800
C(15')-H(15D)	0.9800
C(15')-H(15E)	0.9800
C(15')-H(15F)	0.9800
C(16)-H(16A)	0.9800
C(16)-H(16B)	0.9800
C(16)-H(16C)	0.9800
C(17)-C(22)	1.382(6)
C(17)-C(18)	1.387(6)
C(18)-C(19)	1.394(7)
C(18)-H(18A)	0.9500
C(19)-C(20)	1.394(8)
C(19)-H(19A)	0.9500
C(20)-C(21)	1.371(8)
C(20)-H(20A)	0.9500
C(21)-C(22)	1.399(6)
C(21)-H(21A)	0.9500
C(24)-C(25)	1.501(7)
C(24)-H(24A)	0.9900
C(24)-H(24B)	0.9900
C(25)-C(30)	1.397(7)
C(25)-C(26)	1.398(7)
C(26)-C(27)	1.394(8)
C(26)-C(31)	1.501(9)
C(27)-C(28)	1.394(11)
C(27)-H(27A)	0.9500
C(28)-C(29)	1.373(10)
C(28)-C(32)	1.52(4)
C(28)-C(32')	1.58(3)
C(29)-C(30)	1.391(9)
C(29)-H(29A)	0.9500
C(30)-C(33)	1.507(8)
C(31)-H(31A)	0.9800
C(31)-H(31B)	0.9800
C(31)-H(31C)	0.9800
C(32)-H(32A)	0.9800
C(32)-H(32B)	0.9800
C(32)-H(32C)	0.9800
C(32')-H(32D)	0.9800
C(32')-H(32E)	0.9800
C(32')-H(32F)	0.9800

C(33)-H(33A)	0.9800
C(33)-H(33B)	0.9800
C(33)-H(33C)	0.9800

C(23)-Cu-Br	174.61(12)
C(12)-O-C(15')	115(4)
C(12)-O-C(15)	118.6(17)
C(1)-N(1)-C(8)	106.9(3)
C(1)-N(1)-C(9)	127.4(3)
C(8)-N(1)-C(9)	125.6(3)
C(23)-N(2)-C(17)	111.4(3)
C(23)-N(2)-C(8)	124.7(3)
C(17)-N(2)-C(8)	123.1(3)
C(23)-N(3)-C(22)	111.6(4)
C(23)-N(3)-C(24)	124.7(4)
C(22)-N(3)-C(24)	123.5(4)
N(1)-C(1)-C(2)	130.0(4)
N(1)-C(1)-C(6)	107.7(3)
C(2)-C(1)-C(6)	122.1(4)
C(3)-C(2)-C(1)	117.4(4)
C(3)-C(2)-H(2A)	121.3
C(1)-C(2)-H(2A)	121.3
C(2)-C(3)-C(4)	121.7(4)
C(2)-C(3)-H(3A)	119.1
C(4)-C(3)-H(3A)	119.1
C(5)-C(4)-C(3)	121.2(4)
C(5)-C(4)-H(4A)	119.4
C(3)-C(4)-H(4A)	119.4
C(4)-C(5)-C(6)	118.7(4)
C(4)-C(5)-H(5A)	120.7
C(6)-C(5)-H(5A)	120.7
C(5)-C(6)-C(1)	118.9(4)
C(5)-C(6)-C(7)	132.7(4)
C(1)-C(6)-C(7)	108.2(3)
C(8)-C(7)-C(6)	105.6(3)
C(8)-C(7)-C(16)	125.8(4)
C(6)-C(7)-C(16)	128.6(4)
C(7)-C(8)-N(1)	111.6(3)
C(7)-C(8)-N(2)	128.3(4)
N(1)-C(8)-N(2)	119.7(3)
C(14)-C(9)-C(10)	120.3(4)

C(14)-C(9)-N(1)	120.0(3)
C(10)-C(9)-N(1)	119.6(3)
C(11)-C(10)-C(9)	119.6(4)
C(11)-C(10)-H(10A)	120.2
C(9)-C(10)-H(10A)	120.2
C(10)-C(11)-C(12)	120.7(4)
C(10)-C(11)-H(11A)	119.7
C(12)-C(11)-H(11A)	119.7
O-C(12)-C(13)	125.1(4)
O-C(12)-C(11)	115.4(4)
C(13)-C(12)-C(11)	119.5(4)
C(12)-C(13)-C(14)	119.8(4)
C(12)-C(13)-H(13A)	120.1
C(14)-C(13)-H(13A)	120.1
C(9)-C(14)-C(13)	120.1(4)
C(9)-C(14)-H(14A)	119.9
C(13)-C(14)-H(14A)	119.9
O-C(15)-H(15A)	109.4
O-C(15)-H(15B)	109.4
H(15A)-C(15)-H(15B)	109.5
O-C(15)-H(15C)	109.6
H(15A)-C(15)-H(15C)	109.5
H(15B)-C(15)-H(15C)	109.5
O-C(15')-H(15D)	109.6
O-C(15')-H(15E)	109.6
H(15D)-C(15')-H(15E)	109.5
O-C(15')-H(15F)	109.2
H(15D)-C(15')-H(15F)	109.5
H(15E)-C(15')-H(15F)	109.5
C(7)-C(16)-H(16A)	109.5
C(7)-C(16)-H(16B)	109.5
H(16A)-C(16)-H(16B)	109.5
C(7)-C(16)-H(16C)	109.5
H(16A)-C(16)-H(16C)	109.5
H(16B)-C(16)-H(16C)	109.5
C(22)-C(17)-C(18)	122.5(4)
C(22)-C(17)-N(2)	105.8(4)
C(18)-C(17)-N(2)	131.7(4)
C(17)-C(18)-C(19)	115.9(5)
C(17)-C(18)-H(18A)	122.1
C(19)-C(18)-H(18A)	122.1

C(20)-C(19)-C(18)	121.5(5)
C(20)-C(19)-H(19A)	119.2
C(18)-C(19)-H(19A)	119.2
C(21)-C(20)-C(19)	122.3(4)
C(21)-C(20)-H(20A)	118.8
C(19)-C(20)-H(20A)	118.8
C(20)-C(21)-C(22)	116.3(5)
C(20)-C(21)-H(21A)	121.8
C(22)-C(21)-H(21A)	121.8
C(17)-C(22)-C(21)	121.4(4)
C(17)-C(22)-N(3)	106.4(4)
C(21)-C(22)-N(3)	132.1(4)
N(3)-C(23)-N(2)	104.7(3)
N(3)-C(23)-Cu	130.7(3)
N(2)-C(23)-Cu	124.4(3)
N(3)-C(24)-C(25)	112.9(4)
N(3)-C(24)-H(24A)	109.0
C(25)-C(24)-H(24A)	109.0
N(3)-C(24)-H(24B)	109.0
C(25)-C(24)-H(24B)	109.0
H(24A)-C(24)-H(24B)	107.8
C(30)-C(25)-C(26)	120.2(5)
C(30)-C(25)-C(24)	120.7(5)
C(26)-C(25)-C(24)	119.0(5)
C(27)-C(26)-C(25)	118.9(5)
C(27)-C(26)-C(31)	119.8(5)
C(25)-C(26)-C(31)	121.3(5)
C(28)-C(27)-C(26)	121.2(6)
C(28)-C(27)-H(27A)	119.4
C(26)-C(27)-H(27A)	119.4
C(29)-C(28)-C(27)	119.0(6)
C(29)-C(28)-C(32)	109.4(14)
C(27)-C(28)-C(32)	131.6(14)
C(29)-C(28)-C(32')	130.2(14)
C(27)-C(28)-C(32')	110.8(14)
C(28)-C(29)-C(30)	121.5(6)
C(28)-C(29)-H(29A)	119.3
C(30)-C(29)-H(29A)	119.3
C(29)-C(30)-C(25)	119.2(5)
C(29)-C(30)-C(33)	118.1(6)
C(25)-C(30)-C(33)	122.7(5)

C(26)-C(31)-H(31A)	109.5
C(26)-C(31)-H(31B)	109.5
H(31A)-C(31)-H(31B)	109.5
C(26)-C(31)-H(31C)	109.5
H(31A)-C(31)-H(31C)	109.5
H(31B)-C(31)-H(31C)	109.5
C(28)-C(32)-H(32A)	109.5
C(28)-C(32)-H(32B)	109.5
H(32A)-C(32)-H(32B)	109.5
C(28)-C(32)-H(32C)	109.5
H(32A)-C(32)-H(32C)	109.5
H(32B)-C(32)-H(32C)	109.5
C(28)-C(32')-H(32D)	109.5
C(28)-C(32')-H(32E)	109.5
H(32D)-C(32')-H(32E)	109.5
C(28)-C(32')-H(32F)	109.5
H(32D)-C(32')-H(32F)	109.5
H(32E)-C(32')-H(32F)	109.5
C(30)-C(33)-H(33A)	109.5
C(30)-C(33)-H(33B)	109.5
H(33A)-C(33)-H(33B)	109.5
C(30)-C(33)-H(33C)	109.5
H(33A)-C(33)-H(33C)	109.5
H(33B)-C(33)-H(33C)	109.5

Symmetry transformations used to generate equivalent atoms:

Table 4. Anisotropic displacement parameters ($Å^2$x 10^3) for E2. The anisotropic displacement factor exponent takes the form: $-2\pi^2[h^2 a^{*2}U^{11} + ... + 2 h k a^* b^* U^{12}]$

	U^{11}	U^{22}	U^{33}	U^{23}	U^{13}	U^{12}
Cu	24(1)	32(1)	16(1)	6(1)	5(1)	15(1)
Br	51(1)	92(1)	38(1)	16(1)	13(1)	44(1)
O	44(2)	53(2)	34(2)	30(2)	16(2)	25(2)
N(1)	12(1)	18(2)	17(2)	7(1)	3(1)	6(1)
N(2)	15(2)	19(2)	17(2)	3(1)	4(1)	6(1)
N(3)	18(2)	25(2)	24(2)	4(1)	-2(1)	9(1)
C(1)	13(2)	19(2)	17(2)	3(1)	2(1)	8(1)
C(2)	14(2)	23(2)	26(2)	8(2)	6(2)	9(2)
C(3)	15(2)	30(2)	29(2)	4(2)	0(2)	8(2)
C(4)	22(2)	38(2)	23(2)	8(2)	0(2)	16(2)
C(5)	26(2)	29(2)	21(2)	9(2)	4(2)	14(2)
C(6)	19(2)	18(2)	19(2)	5(1)	4(1)	9(1)
C(7)	21(2)	18(2)	21(2)	6(2)	6(2)	6(2)
C(8)	13(2)	16(2)	16(2)	2(1)	2(1)	4(1)
C(9)	16(2)	18(2)	17(2)	6(1)	5(1)	7(1)
C(10)	20(2)	30(2)	29(2)	14(2)	12(2)	15(2)
C(11)	27(2)	38(2)	29(2)	18(2)	18(2)	17(2)
C(12)	27(2)	27(2)	22(2)	12(2)	7(2)	10(2)
C(13)	23(2)	23(2)	24(2)	8(2)	4(2)	13(2)
C(14)	17(2)	19(2)	22(2)	5(2)	4(1)	9(1)
C(15)	62(7)	88(9)	48(15)	49(10)	21(7)	47(8)
C(15')	62(7)	88(9)	48(15)	49(10)	21(7)	47(8)
C(16)	32(2)	25(2)	32(2)	16(2)	9(2)	5(2)
C(17)	16(2)	18(2)	29(2)	5(2)	7(2)	4(1)
C(18)	26(2)	23(2)	29(2)	5(2)	13(2)	6(2)
C(19)	32(2)	22(2)	53(3)	13(2)	28(2)	10(2)
C(20)	21(2)	21(2)	68(4)	12(2)	20(2)	7(2)
C(21)	17(2)	21(2)	55(3)	10(2)	7(2)	7(2)
C(22)	14(2)	19(2)	32(2)	4(2)	3(2)	4(1)
C(23)	17(2)	21(2)	17(2)	4(1)	0(1)	6(1)
C(24)	33(2)	37(2)	29(2)	6(2)	-8(2)	18(2)
C(25)	26(2)	31(2)	20(2)	4(2)	-9(2)	9(2)
C(26)	34(2)	33(2)	27(2)	6(2)	-8(2)	5(2)
C(27)	61(4)	32(3)	36(3)	-1(2)	-18(3)	16(3)
C(28)	67(4)	65(4)	24(3)	-8(3)	-10(3)	39(3)
C(29)	47(3)	78(4)	18(2)	6(2)	-1(2)	31(3)

C(30)	32(2)	48(3)	19(2)	11(2)	-6(2)	12(2)
C(31)	43(3)	51(3)	48(3)	17(3)	4(3)	3(3)
C(32)	89(17)	47(14)	46(8)	-5(9)	14(8)	28(11)
C(32')	89(17)	47(14)	46(8)	-5(9)	14(8)	28(11)
C(33)	43(3)	58(4)	43(3)	28(3)	-3(3)	5(3)

Table 5. Hydrogen coordinates (x 10^4) and isotropic displacement parameters ($Å^2$x 10^3) for E2.

	x	y	z	U(eq)
H(2A)	8586	10261	5411	25
H(3A)	10064	10453	6883	32
H(4A)	9298	8856	7744	33
H(5A)	6989	7065	7191	29
H(10A)	7514	8686	3529	28
H(11A)	7487	9640	2233	33
H(13A)	3916	10294	2728	27
H(14A)	3993	9396	4060	23
H(15A)	4772	11257	631	84
H(15B)	3614	10213	1058	84
H(15C)	4654	11668	1711	84
H(15D)	4889	11485	798	84
H(15E)	3755	10577	1316	84
H(15F)	5096	11937	1931	84
H(16A)	4334	5452	6353	46
H(16B)	2939	5704	5883	46
H(16C)	3495	4769	5248	46
H(18A)	2450	7650	5819	32
H(19A)	141	7763	5834	41
H(20A)	-1647	7186	4422	43
H(21A)	-1249	6474	2935	38
H(24A)	1059	6682	1504	41
H(24B)	-388	5586	1680	41
H(27A)	-93	1629	618	59
H(29A)	2857	4321	-392	58
H(31A)	-873	3961	2180	79
H(31B)	-1840	2555	1438	79
H(31C)	-495	2729	2282	79
H(32A)	1375	1030	-629	95
H(32B)	1682	2048	-1293	95
H(32C)	2985	2219	-420	95
H(32D)	819	763	-360	95
H(32E)	1209	1429	-1225	95
H(32F)	2519	1597	-358	95
H(33A)	2614	7127	951	79

H(33B)	3932	6739	726	79
H(33C)	2627	6596	-135	79
